# Steric and Geometric Tuning of π‐Conjugated Antennae in Europium(III) Complexes for Selective ADP Recognition

**DOI:** 10.1002/chem.202502251

**Published:** 2025-08-22

**Authors:** Samantha E. Bodman, Stephen J. Butler

**Affiliations:** ^1^ Department of Chemistry Loughborough University Epinal Way Loughborough UK

**Keywords:** anion receptor, europium, host‐guest recognition, lanthanide probe, luminescent probe

## Abstract

The selective recognition of adenosine diphosphate (ADP) in water presents a significant challenge for synthetic supramolecular chemistry, driven by its biological importance in cellular energy transfer and enzymatic signaling pathways. Discriminating ADP from structurally similar anions such as ATP requires a high degree of host–guest complementarity. We recently developed [**Eu.ADPGlow**]^−^, a luminescent Eu(III) complex bearing two 6‐substituted quinolyl‐phenoxyacetate arms, which create a binding site at the central Eu(III) ion that accommodates ADP. Binding induces a tight interaction in water, involving both metal coordination and π–π stacking, switching the emission on with a 33‐fold enhancement. Here, we examine how systematic changes in ligand geometry influence anion selectivity, by synthesizing four new Eu(III) complexes with pendant arms at the 4‐ or 7‐positions of the quinoline scaffold. The 4‐substituted systems provide a more accessible binding site and bind ADP, ATP, and AMP with limited selectivity between them, while the 7‐substituted analogues impose steric hindrance at the Eu(III) center, resulting in minimal response to all tested anions. Only the 6‐substituted complex [**Eu.ADPGlow**]^−^ achieves optimal geometrical complementarity for ADP binding. These findings reinforce the importance of steric and geometric control in the design of selective lanthanide probes for biological anions in water.

## Graphical abstract

We present four new macrocyclic Eu(III) complexes and demonstrate how ligand structure and positional tuning of π‐conjugated antennae modulate steric demand and anion binding site geometry. This enables size and shape complementarity for selective ADP recognition in water over competing phosphoanions.

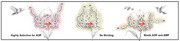

## 1 Introduction

Luminescent lanthanide probes are established as useful tools for the selective recognition of anions in water and in biological media, owing to their unique photophysical properties.^[^ ^1^, ^2^, ^3^, ^4^ ^]^ These include: (i) long luminescence lifetimes (milliseconds), enabling time‐resolved and time‐gated detection to eliminate short‐lived autofluorescence from biomolecules; (ii) line‐like emission spectra suitable for ratiometric analysis; and (iii) fast, sensitive luminescence responses compatible with biological sensing and imaging.^[^ ^5^, ^6^, ^7^, ^8^, ^9^, ^10^, ^11^ ^]^ Direct coordination of certain oxyanions to lanthanide centers provides sufficient free energy for complexation in competitive aqueous media, while careful ligand design enables discrimination between structurally similar biological anions, using multiple noncovalent interactions operating together.^[^ ^12^, ^13^, ^14^, ^15^, ^16^, ^17^, ^18^ ^]^

Nucleoside phosphates such as adenosine diphosphate (ADP) and adenosine triphosphate (ATP) are key targets for supramolecular detection due to their critical roles in cellular energy metabolism and signaling.^[^ ^19^, ^20^, ^21^, ^22^ ^]^ The creation of synthetic receptors that selectively bind ADP could enable enzymatic processes to be visualized in complex biological environments such as cells, but it poses distinct challenges, since it differs from the more abundant anion ATP by just one less phosphate group, and other highly charged polyphosphate anions such as pyrophosphate (PPi) often interfere. The reduced charge of ADP means that electrostatic attraction alone is insufficient to drive selective recognition; instead, receptors must incorporate additional complementary interactions, such as hydrogen bonding or π–π stacking with the adenosine moiety. These binding motifs must be suitably positioned to achieve optimal steric and geometric complementarity.^[^ ^20^ ^]^

A range of organic receptors and metal‐ion‐based coordination complexes have been explored for ADP recognition, with only a few demonstrating useful levels of selectivity in aqueous solution. Duan et al. developed an aminonaphthalimide‐based imidazolium turn‐on probe for selective ADP detection.^[^ ^23^ ^]^ The probe displayed a 1.5‐fold increase in fluorescence in the presence of ADP, attributed to the PET effect through hydrogen bonding interactions with the phosphate and π‐π stacking of the adenosine with the 1,8‐naphthalimide group. Anthracene‐containing Zn(II) probes that showed selectivity for ADP over ATP and AMP have been developed by Feng and coworkers.^[^ ^24^ ^]^ The anion binds through electrostatic interactions between the Zn(II) and diphosphate, with an additional π‐π interaction between the acene and adenosine units displaying a unique fluorescence response. The authors furthered their design for cellular applications, with the addition of four amino groups to aid in solubility.^[^ ^25^ ^]^ This probe displayed a 133‐fold fluorescence enhancement for ADP, compared with a significant 56‐fold increase with ATP.

We previously reported a Eu(III) complex, [**Eu.1**]^+^, capable of reversibly binding ADP in water, enabling real‐time monitoring of kinase‐catalyzed ATP‐to‐ADP conversion.^[^ ^26^, ^27^ ^]^ However, [**Eu.1**]^+^ was incompatible with cellular environments due to appreciable binding to bicarbonate^[^ ^28^ ^]^ and human serum albumin (HSA), both of which elicit an increase in luminescence.^[^ ^29^ ^]^ We recently addressed these limitations with [**Eu.ADPGlow**]^−^, a Eu(III) complex featuring a C_2_‐symmetric macrocyclic core and two 6‐substituted quinoline‐phenoxyacetate antennae.^[^ ^30^ ^]^ The π‐conjugated arms play a crucial role in selectively recognizing ADP over other nucleotide polyphosphate anions, including ATP and AMP. This selectivity is achieved through metal–phosphate coordination and π–π stacking interactions with the adenosine moiety, as determined through emission spectroscopy and DFT calculations. The probe design produces a 33‐fold luminescence enhancement upon ADP binding in aqueous solution, with almost no interference from ATP, AMP, PPi, bicarbonate, or HSA protein.

**Figure 1:**
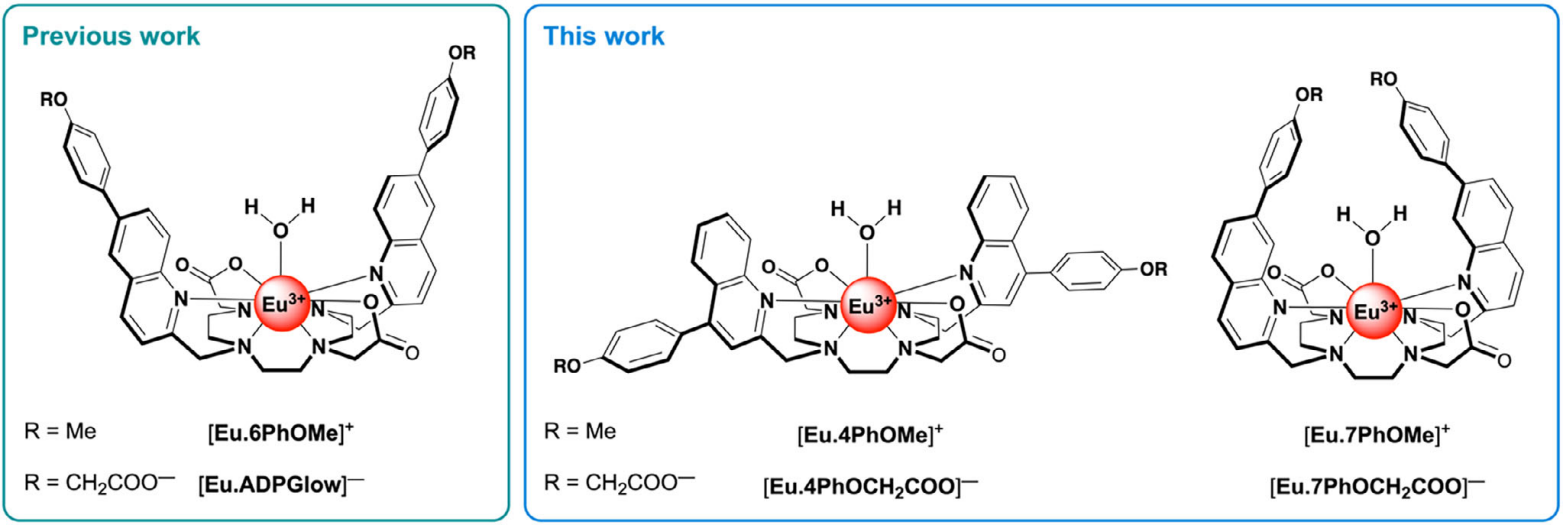
(left) Structures of the previously reported probes [**Eu.6PhOMe**]^+^ and [**Eu.ADPGlow**]^−^; (right) Structures of the novel regioisomeric probes presented in this work, designed to identify the optimal steric and geometric features for selective ADP binding.

In this work, we investigate how the position, geometry, and steric demand of the π‐conjugated arms modulate the shape and depth of the anion binding site around the Eu(III) center, thereby influencing anion selectivity. We anticipated that steric hindrance and binding site geometry play a central role in controlling access to the Eu(III) center and complementarity with the ADP structure. By varying the substitution pattern of the π‐conjugated quinoline arms, we aimed to identify structural features that enhance ADP selectivity by optimizing both steric accessibility and engaging both the adenosine and phosphate moieties. To this end, we synthesized four new Eu(III) complexes, [**Eu.4PhOMe**]⁺, [**Eu.4PhOCH_2_COO**]^−^, [**Eu.7PhOMe**]⁺, and [**Eu.7PhOCH_2_COO**]^−^ (Figure 1), in which the phenoxyacetate or methoxyphenyl groups are positioned either at the 4‐ or 7‐position of the coordinated quinoline arms. The 4‐substituted complexes were expected to generate a more open and accessible binding site relative to [**Eu.ADPGlow**]^−^, potentially accommodating a wider range of anions. In contrast, the 7‐substituted complexes were designed to create a more sterically encumbered binding cavity, which could restrict access but possibly still offer favorable ADP binding through multiple‐point interactions. The anion binding behavior of these complexes was examined using NMR spectroscopy, single‐crystal X‐ray diffraction, mass spectrometry, and photophysical analysis.

## 2 Results and Discussion

### 2.1 Synthesis

**Scheme 1:**
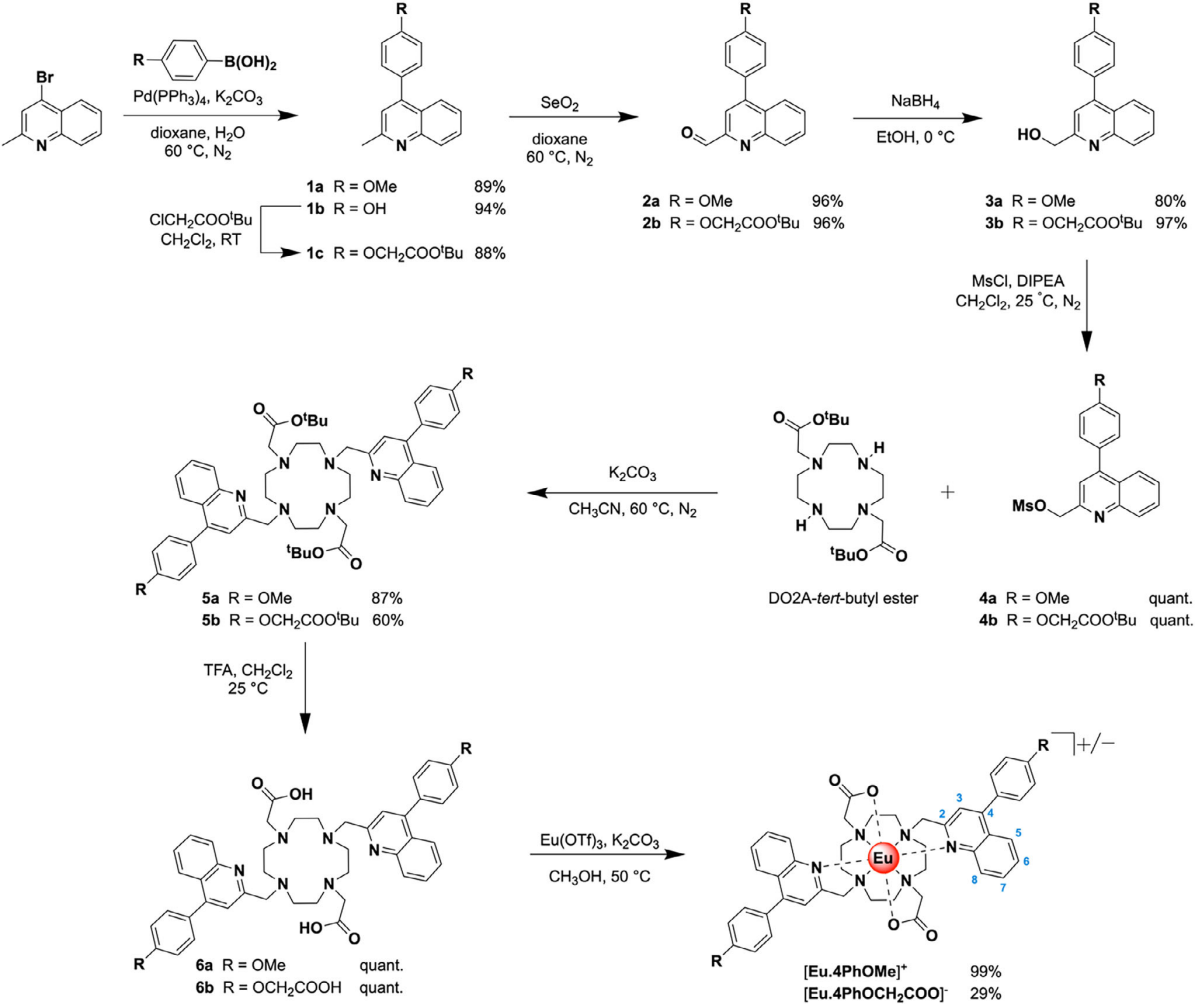
Synthesis of complexes [**Eu.4PhOMe**]^+^ and [**Eu.4PhOCH_2_COO**]^−^.

A representative synthesis of the target complexes [**Eu.4PhOMe**]^+^ and [**Eu.4PhOCH_2_COO**]^−^ is given in Scheme 1, and follows similar methodology described previously for [**Eu.ADPGlow**]^−^.^[^ ^30^ ^]^ Full details of the synthesis and characterization of all complexes are provided in the Supporting Information. Briefly, the π‐conjugated moieties were introduced through Suzuki cross‐coupling reactions of 4‐bromoquinaldine and the appropriately functionalized boronic acid derivative, to give compounds **1a** and **1b**. *O*‐alkylation of **1b** gave the *tert*‐butyl protected carboxylate functionality **1c**. Subsequent oxidation with selenium dioxide gave aldehydes **2a** and **2b**, followed by reduction to the corresponding alcohols **3a** and **3b** using sodium borohydride. Mesylation of the alcohols resulted in mesylate esters **4a** and **4b,** which were *N*‐alkylated with DO2A‐tert‐butyl ester to give the protected macrocycles **5a** and **5b**. Deprotection of the *tert*‐butyl groups with trifluoroacetic acid gave the free ligands **6a** and **6b**. These were then treated with Eu(OTf)_3_ in methanol to afford the target complexes [**Eu.4PhOMe**]^+^ and [**Eu.4PhOCH_2_COO**]^−^, which were purified by normal‐phase and reverse‐phase chromatography, respectively. The complexation reactions showed complete consumption of the free ligands in each case, however significant losses in yield were observed during reverse‐phase HPLC purification.

### 2.2 X‐ray Crystallography

We investigated the structure of the Eu(III) complexes by single‐crystal X‐ray diffraction to give insight into the conformation and binding site configuration at the Eu(III) center. Attempts to grow crystals of the water‐soluble complexes [**Eu.4PhOCH_2_COO**]^−^ and [**Eu.7PhOCH_2_COO**]^−^ were unsuccessful; however, crystals of the corresponding organic‐soluble complexes [**Eu.4PhOMe**]^+^ and [**Eu.7PhOMe**]^+^ were obtained after slow evaporation of each complex from methanol and water (9:1 v/v).^1^

**Figure 2:**
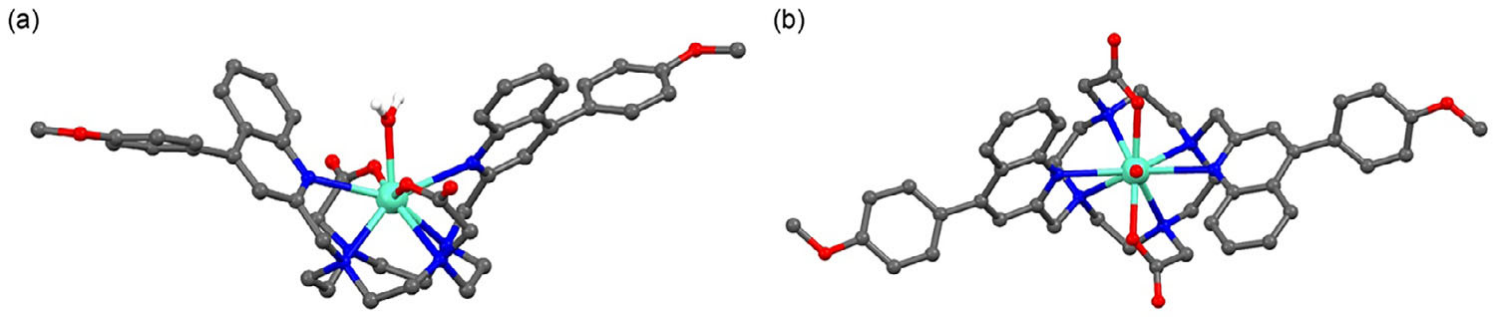
Single crystal X‐ray structure of [**Eu.4PhOMe**]^+^ viewed a) perpendicular to the b axis and b) along the main pseudo‐C2 axis. The hydrogen atoms of the coordinating water molecule are shown, all other hydrogen atoms, non‐coordinating water molecules, and the triflate counter ion have been omitted for clarity. Only the major macrocycle conformation is shown; the lowest occupancy disorder component has been omitted. Atom colors: Eu green, C gray, N blue, O red, H white.

The complexes solved in the monoclinic space groups, *P*2_1_/*n* and *P*2_1_/*c*, respectively. Complex [**Eu.4PhOMe**]^+^ contains one molecule in the asymmetric unit, with one coordinated water molecule in the axial site to the Eu(III) center (Figure 2). The diffraction of the crystal was streaky, indicative of underlying disorder, which complicated the structural refinement. A solvent mask was applied to account for the noncoordinating solvent molecules (details are given in the Supporting Information). Despite these challenges, the desired complex structure was modeled, revealing positional disorder of both the macrocycle and the triflate counter ion over two sites. The coordination of the ligand to the Eu(III) displayed the pendant arms on the same face of the macrocycle, with bond lengths in the expected range, (Eu‐N_cyclen_ 2.673(10)–2.740(11) Å, Eu‐N_quinoline_ 2.755(5) Å and Eu‐O_acetate_ 2.364(5) Å. The 9^th^ coordination site is occupied by a water molecule, which displays a slightly longer bond length (Eu‐O_H2O_ 2.392(6) Å) than the carboxylate oxygen atoms. With two conformations of the macrocyclic unit apparent (the largest occupancy being 67%), the coordination geometry around the metal center of this complex was determined to be twisted square antiprismatic (TSAP) for the major component (calculated torsion angles of ∼31° and ∼28°) and square antiprismatic (SAP) for the minor component (calculated torsion angles of ∼45° and ∼41°). The square is defined by the cyclen ring nitrogen atoms, and the square coordinates the oxygen atoms of the two carboxylate arms and the two coordinated nitrogen atoms of the quinoline rings.

The methoxy‐phenyl rings are twisted out of the plane of the quinoline ring by 56.2(2)° and 59.0(2)°, which limits π‐π stacking interactions between complexes in the packing. As shown in Figure 2a, the π‐conjugated groups at the 4‐position create a relatively open binding site at the Eu(III) center, with minimal steric hindrance and clear accessibility of the coordinated water molecule at the axial position. This open geometry is expected to facilitate the approach of anions and displacement of the bound water. In contrast, the previously reported X‐ray crystal structure of the related gadolinium(III) complex [**Gd.6PhOMe**]^+^, featuring π‐conjugation at the 6‐position, revealed a deeper binding site at the metal center.^[^ ^30^ ^]^ This arises from the two 6‐substituted quinoline‐methoxyphenyl antennae that project above the macrocycle. In that case, the Gd(III) center exclusively adopts a TSAP coordination geometry, which could also contribute to a more sterically enclosed coordination environment.^[^ ^31^ ^]^

**Figure 3:**
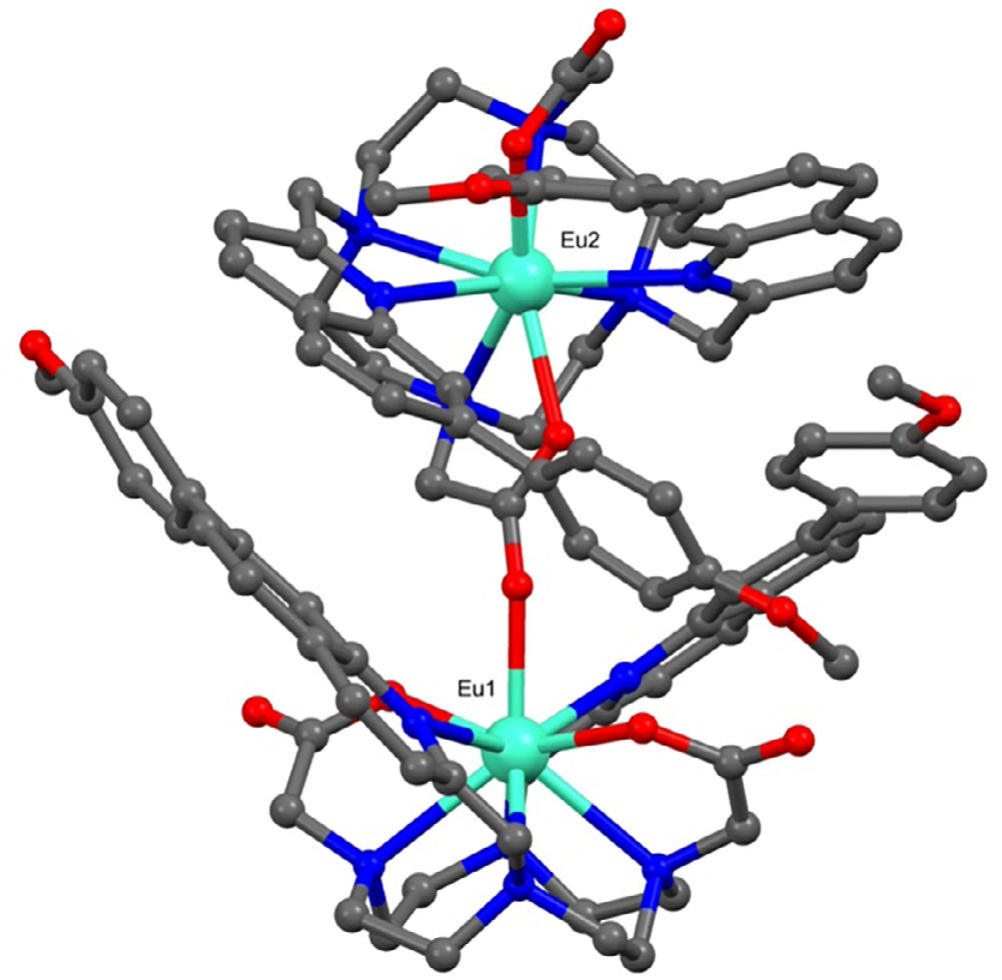
a) Single crystal X‐ray crystal structure of [**Eu.7PhOMe**]^+^, displaying the carboxylate coordination to Eu1 and the twisted geometry of Eu2 along the main pseudo‐C2 axis. The hydrogen atoms, noncoordinating water molecules and counter ions have been omitted for clarity. The lowest occupancy disorder component has also been omitted. Atom colors: Eu green, C gray, N blue, O red.

The regioisomeric complex [**Eu.7PhOMe**]^+^ showed a distinctive structure and crystallized with two Eu(III) complexes within the asymmetric unit, linked together by the coordination of one of the cyclen‐carbonyl oxygen atoms to the Eu(III) metal of the neighboring complex (Figure 3). Due to the presence of disorder in the crystallographic data, only one triflate counter ion could be modeled, while the other counter ion and solvent molecules (methanol and water) in the asymmetric unit were identified through electron density peaks and could not be reliably modeled because of the disorder. To address this, a solvent mask was applied, details are provided in the Supporting Information.

For [**Eu.7PhOMe**]^+^, the ligand coordinates to the Eu(III) ion through the four nitrogen arms of the cyclen ring and the four pendant arms, including the quinoline N‐atoms and the acetate O‐atoms (Figure 3). The coordinating atom bond lengths from the ligand are in the expected range (Eu‐O 2.3–2.4 Å, Eu‐N 2.6–2.8 Å); with the longer bond lengths associated with the quinoline nitrogen atoms. Notably, the Eu(III) ions within the two distinct complexes, referred to as Eu1 and Eu2 (Figure 3), have different coordination numbers, of 9 and 8, respectively. The axial coordination of the acetate oxygen of Eu2 to the metal center of Eu1 creates the 9‐coordinate geometry with a relatively long bond length of 2.388(3) Å. The 9‐coordinate Eu1 adopts a SAP geometry with the octadentate ligand, with angles of ∼40° defined by the square defined by the cyclen ring N‐atoms and the square coordinating the pendant arms.

Interestingly, the second Eu(III) center in the X‐ray structure of [**Eu.7PhOMe**]^+^ adopts an 8‐coordinate geometry with no coordinated solvent. In this case, the complex adopts a more compact structure due to the closer coordination of the quinoline nitrogen atoms, as reflected in the shorter Eu2‐N_quinoline_ bond lengths of 2.595(4) and 2.663(5) Å. The quinoline arms are positioned on the same face of the complex but oriented in opposite directions, creating a TSAP conformation in the Eu2 unit. The more enclosed and twisted structure of [**Eu.7PhOMe**]^+^ appears to be stabilized by face‐to‐face π–π interactions within the dimeric assembly, specifically between the quinoline rings of Eu1 and Eu2, and between a phenyl ring of Eu1 and a quinoline ring of Eu2 (Figure 3). These interactions are enabled by the out‐of‐plane twisting of the phenyl rings relative to the quinoline units, with the closest centroid‐to‐centroid distance measured at 3.751(9) Å. During the synthesis of [**Eu.7PhOMe**]^+^, we obtained crystals of compound **3c** (Figures S2, S3) which displayed a similar twist of the phenyl ring out of the plane of the quinoline ring (30.29(3)°) to that observed for complex [**Eu.7PhOMe**]^+^. Overall, the X‐ray structure of [**Eu.7PhOMe**]^+^ reveals a more sterically encumbered binding site around the Eu(III) center, consistent with stronger and shorter bonds to the quinoline arms. The absence of a coordinated water molecule in Eu2, coupled with the preference for intermolecular dimerization with Eu1, could potentially hinder anion access and reduce the binding affinity and rate of anion/water exchange.

### 2.3 NMR Studies

**Figure 4:**
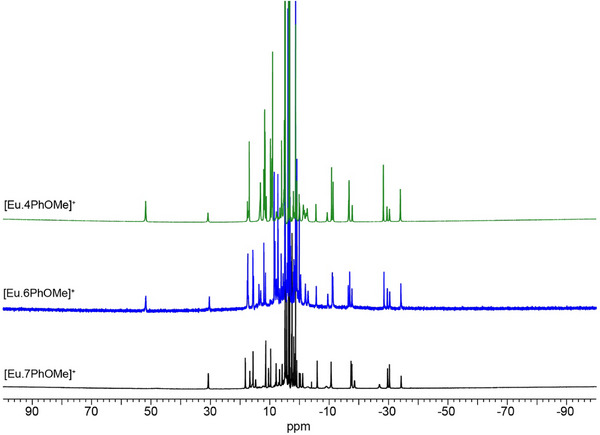
^1^H NMR spectra (500 MHz, CD_3_OD) of [**Eu.4PhOMe**]^+^ (green), [**Eu.6PhOMe**]^+^ (blue), and [**Eu.7PhOMe**]^+^ (black) recorded at 298 K. The spectrum of complex [**Eu.6PhOMe**]^+^ was reproduced from previous work.^[^ ^30^ ^]^

The ^1^H NMR spectra of the methoxyphenyl‐substituted Eu(III) complexes were compared in CD_3_OD (Figure 4). Each complex displayed two sets of proton resonances, consistent with the presence of two diastereomers in solution. These are attributed to the SAP and TSAP coordination geometries, with each species present in different proportions.^[^ ^32^ ^]^ The ^1^H NMR spectra of [**Eu.4PhOMe**]^+^ and the previously reported [**Eu.6PhOMe**]^+^ are similar, with the major isomer corresponding to the SAP geometry. This assignment is supported by the highly shifted proton signals at +50 ppm and −28.5 ppm, tentatively attributed to an axial cyclen proton and an acetate arm proton, respectively.^[^ ^33^, ^34^ ^]^ For [**Eu.4PhOMe**]^+^, integration of the axial proton signals at +50 ppm and +30 ppm revealed a SAP:TSAP ratio of 2:1. Notably, the X‐ray crystal structure of [**Eu.4PhOMe**]^+^ shows predominantly a TSAP geometry, highlighting that the solid‐state conformation does not necessarily reflect the dominant species in solution.

Upon moving the methoxyphenyl substituents from the 4‐ to the 6‐position in [**Eu.6PhOMe**]^+^, the proportion of the TSAP isomer increases, giving a SAP:TSAP ratio of 5:4. This trend continues in [**Eu.7PhOMe**]^+^, where the TSAP isomer becomes dominant. In this case, the signals corresponding to the SAP geometry, particularly the axial proton at +50 ppm, are notably broadened, suggesting increased conformational flexibility or reversible intermolecular association at the Eu(III) center on the NMR timescale. This observation is consistent with the X‐ray crystal structure of [**Eu.7PhOMe**]^+^, which shows dimerization and the lack of a coordinated water molecule at one of the Eu(III) centers (Figure 3). Integration of the four signals in the region from −25 to −35 ppm, indicated a SAP:TSAP ratio of 3:5 for [**Eu.7PhOMe**]^+^. Overall, these data indicate a clear trend: TSAP populations increase in solution as the methoxyphenyl substituents are moved around the quinoline ring from the 4‐ to the 6‐ and 7‐positions. This shift in population may be reasonably attributed to the increasing steric demand imposed by the π‐conjugated substituents in these positions, which increasingly favor the TSAP conformation.

The ^1^H NMR spectra of the water‐soluble Eu(III) complexes bearing phenoxyacetate substituents, recorded in D_2_O, were more complex than those of the methoxyphenyl‐substituted analogues (Figure S5). Each complex displayed more than two sets of signals, accompanied by pronounced exchange broadening. This suggests the presence of multiple conformations in aqueous solution, potentially involving reversible intermolecular association between the peripheral carboxylate groups and Eu(III) centers, particularly at the millimolar concentrations used for NMR analysis. However, due to the extent of exchange broadening and the lower signal‐to‐noise ratio (a consequence of limited sample availability), further interpretation of the solution speciation was not possible.

### 2.4 Photophysical Analysis in Methanol

**Figure 5:**
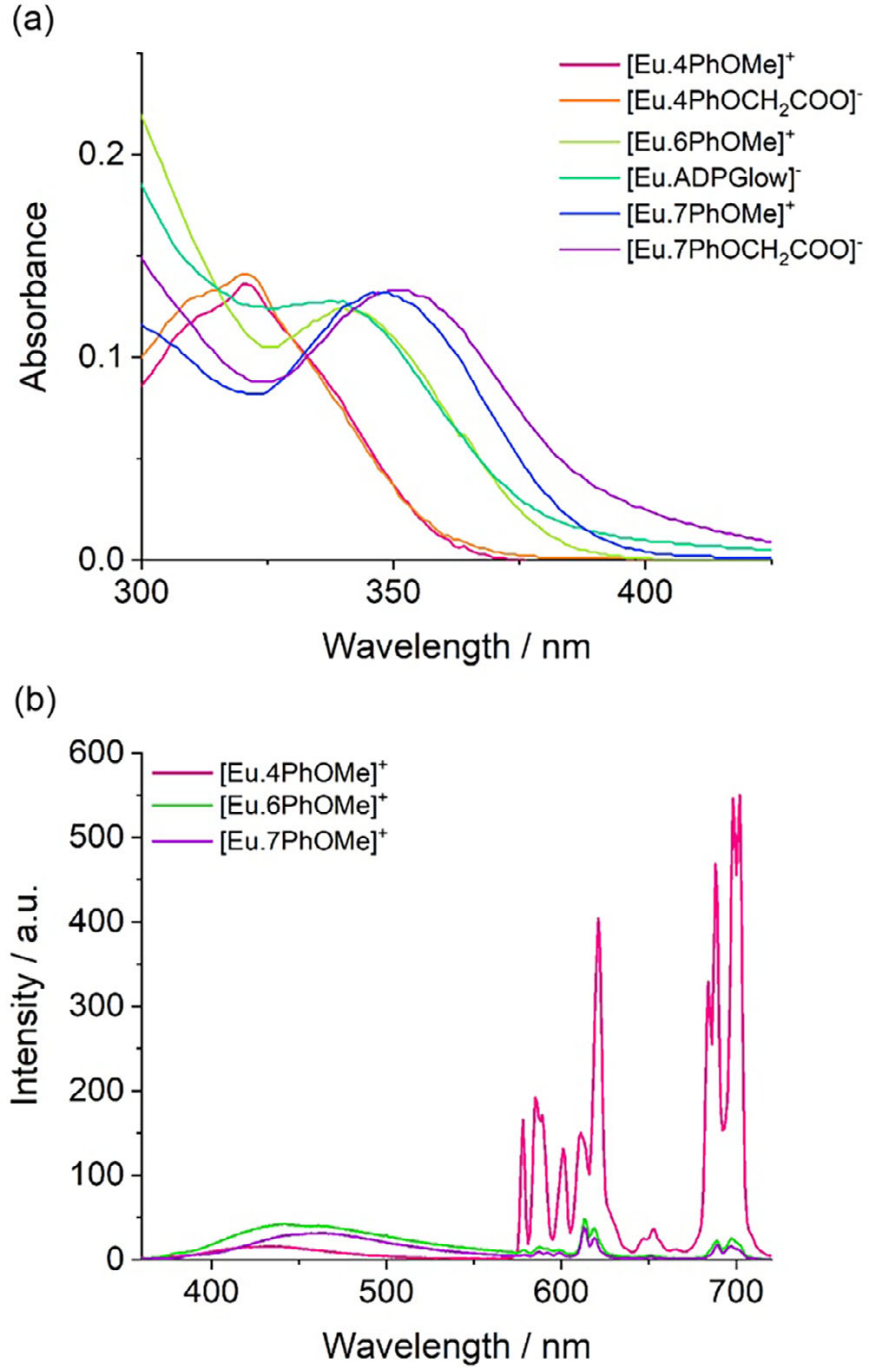
a) Absorption spectra of Eu(III) complexes in methanol at 295 K, showing a progressive red‐shift in absorption maxima as the conjugated substituents move from the 4‐ to 6‐ to 7‐position of the quinoline ring. b) Emission spectra of methoxyphenyl‐substituted Eu(III) complexes, measured in methanol at 0.1 Abs and 295 K.

The photophysical properties of the four novel Eu(III) complexes were initially assessed in methanol, alongside the previously published complexes [**Eu.6PhOMe**]^+^ and [**Eu.ADPGlow**]^−^.^[^ ^30^ ^]^ Each Eu(III) complex displayed two distinct peaks in the UV‐Vis absorption spectra (Figures S6,S7). For the 4‐substituted complexes [**Eu.4PhOMe**]^+^ and [**Eu.4PhOCH_2_COO**]^−^, the lowest energy absorption band occurred at 321 nm. This band red‐shifted to approximately 340 nm for the 6‐substituted complexes and further to 350 nm for the 7‐substituted complexes [**Eu.7PhOMe**]^+^ and [**Eu.7PhOCH_2_COO**]^−^ (Figure 5a). A similar trend was observed in 10 mM HEPES buffer for the three water‐soluble complexes (Figure S7). The highest molar extinction coefficients were recorded for [**Eu.4PhOMe**]^+^ and [**Eu.4PhOCH_2_COO**]^−^ (22400 and 26700 M^−^^1^ cm^−^^1^, respectively), more than twice the values measured for the 6‐substituted analogues (Table 1). Among the water‐soluble complexes, [**Eu.7PhOCH_2_COO**]^−^ offered the best combination of high molar absorptivity (18000 M^−^^1^ cm^−^^1^) and a red‐shifted absorption maximum at 350 nm, making it potentially well‐suited for live‐cell imaging applications with standard fluorescence microscopes, using 355 or 405 nm excitation.^[^ ^35^, ^36^ ^]^

**Table 1:** Photophysical data for Eu(III) complexes.

Complex	Solvent	*λ*_max_ / nm	*ε* / M^−1^ cm^−1^	*τ*_H_ / ms^[^ ^a^ ^]^	*τ*_D_ / ms^[^ ^a^ ^]^	m_CH3OH_	Φ_360–720_ / %^[^ ^b^ ^]^	Φ_550–720_ / %^[^ ^c^ ^]^
[**Eu.4PhOMe**]^+^	Methanol	321	22 400	0.86 ± 0.01	1.34 ± 0.01	0.9	5.9 ± 0.09	5.5 ± 0.04
[**Eu.6PhOMe**]^+^	Methanol	340	8000	0.88 ± 0.09	1.16 ± 0.02	0.7	2.2 ± 0.06	0.6 ± 0.01
[**Eu.7PhOMe**]^+^	Methanol	348	6400	0.99 ± 0.01	1.31 ± 0.01	0.5	1.5 ± 0.02	0.4 ± 0.01
[**Eu.4PhOCH_2_COO**]^−^	HEPES	321	26 700	0.47 ± 0.01	1.00 ± 0.02	1.1	6.7 ± 0.19	2.4 ± 0.09
[**Eu.ADPGlow**]^−^	HEPES	337	9300	0.021 ± 0.001	0.036 ± 0.001	‐	1.1 ± 0.08	0.3 ± 0.01
[**Eu.7PhOCH_2_COO**]^−^	HEPES	350	18 000	0.016 ± 0.001	0.013 ± 0.001	‐	2.7 ± 0.14	0.2 ± 0.01

^[a]^ *τ*_H_ refers to nondeuterated solvent, whereas *τ*_D_ refers to the deuterated solvent. Emission lifetime measurements were conducted in duplicate with errors determined through the averages ± standard deviation. Quantum yields were measured using quinine sulfate in 0.05 M H_2_SO_4_ as a standard (Φ_em_ = 60%).^[^ ^40^ ^]^ Quantum yields were conducted in duplicate with errors determined through the averages ± standard deviation.

^[b]^ Φ**_360–720_** values include the residual ligand fluorescence and the europium‐centered emission (360–720 nm).

^[c]^ Φ**_550–720_** values represent the europium‐centered emission only (550–720 nm).

**Figure 6:**
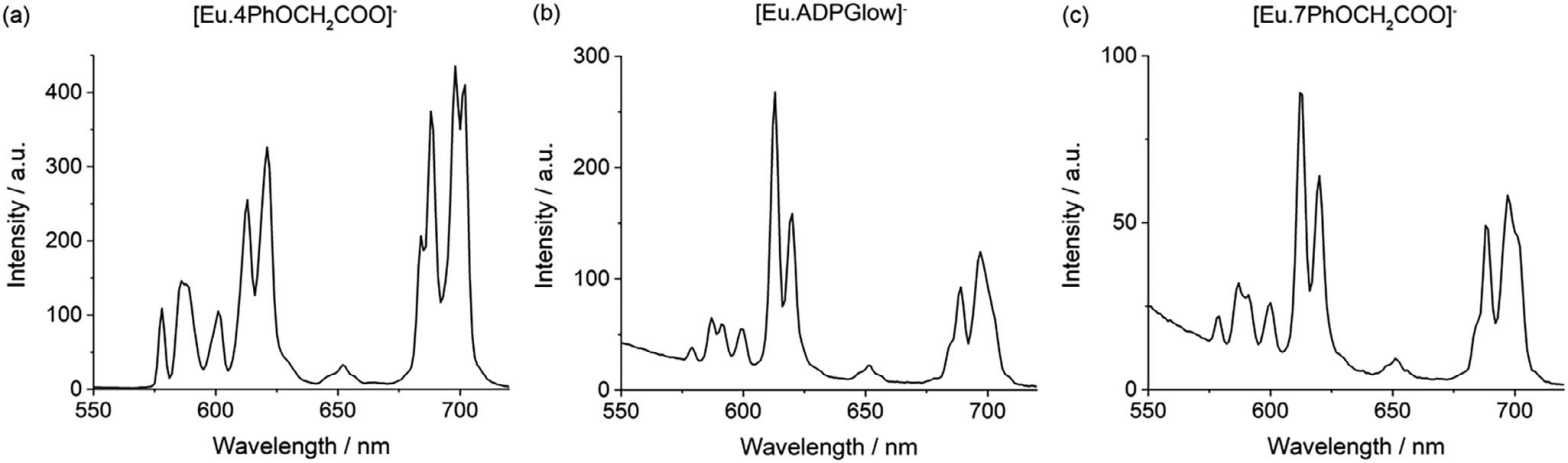
Emission spectra of a) [**Eu.4PhOCH_2_COO**]^−^ (*λ*_exc_ 321 nm), b) [**Eu.ADPGlow**]^−^ (*λ*_exc_ 337 nm), and c) [**Eu.7PhOCH_2_COO**]^−^ (*λ*_exc_ 350 nm), measured in methanol.

All six complexes exhibited characteristic red europium emission between 550–720 nm upon indirect excitation of their quinoline antennae in methanol (Figures 5,6,S8 and S9). The 4‐substituted complexes, [**Eu.4PhOMe**]^+^ and [**Eu.4PhOCH_2_COO**]^−^, displayed nearly identical emission profiles with a strong Δ*J *= 4 band (680–705 nm) comprising four distinct peaks, and relatively low Δ*J* = 2 (605–630 nm) / Δ*J* = 1 (585–605 nm) intensity ratios around 2.3. In contrast, the 6‐ and 7‐substituted analogues exhibited a more prominent Δ*J* = 2 band with two well‐resolved components and higher Δ*J* = 2 / Δ*J *= 1 ratios (approx. 3.0). The Δ*J* = 2 transition is electric‐dipole allowed and hypersensitive to ligand field asymmetry, whereas the Δ*J* = 1 transition is primarily magnetic‐dipole in nature and much less affected by coordination environment.^[^ ^37^ ^]^ The stronger Δ*J* = 2 transitions in the 6‐ and 7‐substituted complexes indicate increased ligand field asymmetry, likely arising from a combination of electronic and steric/geometrical effects. Considering electronic effects, both 4‐ and 7‐substituents are directly conjugated with the quinoline nitrogen donor atoms, potentially increasing the polarizability of the donor site (the ease with which its electron density can be distorted), and thereby, in principle, promoting greater intensity in the hypersensitive ΔJ = 2 electric dipole transition. However, the 6‐substituted complex, which lacks such direct conjugation, also exhibits a pronounced Δ*J* = 2 band. Thus, donor atom polarizability alone cannot account for the observed trend, and steric distortion, particularly in the 6‐substituted system, clearly plays a significant role in increasing the asymmetry of the coordination environment and shaping the overall emission spectrum.^[^ ^34^, ^38^ ^]^ Given that both the 6‐ and 7‐substituted complexes exhibit greater TSAP character in the solution NMR, this conformation can reasonably be attributed to more distorted donor arrangements and reduced symmetry at the metal center. This interpretation is further supported by emission lifetime data (Table 1), which indicate a decrease in the number of coordinated methanol molecules from 0.9 for the 4‐substituted complex to 0.7 and 0.5 for the 6‐ and 7‐substituted analogues, respectively. This trend reflects a shift in the equilibrium toward less solvated species, consistent with a TSAP‐dominated conformation and the presence of a longer and weaker Ln–solvent interaction.^[^ ^39^ ^]^

Among the methoxyphenyl‐substituted Eu(III) complexes, the most emissive complex is [**Eu.4PhOMe**]^+^ (Φ_em _= 5.5%), approximately 10 times more emissive than [**Eu.6PhOMe**]^+^ and [**Eu.7PhOMe**]^+^, both of which also show a higher degree of ligand fluorescence (Figure 5). The higher quantum yield of [**Eu.4PhOMe**]^+^ is attributed primarily to more efficient intramolecular energy transfer, η_ET_, from the quinoline antennae to the metal center (Table S6).^[^ ^41^ ^]^ This is consistent with the blue‐shifted absorption maximum of [**Eu.4PhOMe**]^+^ which potentially offers better overlap with the Eu(III) acceptor levels. Moving to the three phenoxyacetate functionalized Eu(III) complexes, [**Eu.4PhOCH_2_COO**]^−^ displayed the strongest emission (Φ_em_ = 4.5%) in methanol, higher than [**Eu.ADPGlow**]^−^ (Φ_em_ = 2.1%), and [**Eu.7PhOCH_2_COO**]^−^ (Φ_em_ = 1.1%). All three complexes also exhibited a larger proportion of ligand fluorescence compared with their methoxyphenyl analogues (Figure S10).

### 2.5 Photophysical Analysis in HEPES Buffer

All three phenoxyacetate complexes were sufficiently water‐soluble, owing to their peripheral anionic groups, to enable photophysical measurements in 10 mM HEPES buffer (pH 7.0). In buffer, their quantum yields decreased significantly (Table 1), consistent with greater nonradiative decay due to energy transfer to O‐H vibrational modes. The emission spectral shape of [**Eu.4PhOCH_2_COO**]^−^ remained largely unchanged between HEPES buffer and methanol (Figures 6 and S11), although its Eu(III)‐centered quantum yield dropped from 4.5 to 2.4%. Its emission lifetime also decreased to 0.47 ms in HEPES buffer, approximately half the value in methanol, and the number of coordinated water molecules (*q*) was determined to be 1 (Table 1).^[^ ^39^ ^]^ In contrast, [**Eu.7PhOCH_2_COO**]^−^ and [**Eu.ADPGlow**]^−^ exhibited negligible Eu(III) emission and very short lifetimes (∼0.02 ms) in buffer, approximately 50‐fold shorter than in methanol, preventing reliable determination of *q* values. The near‐complete quenching of Eu(III) emission (Figures S11,S12) and very short lifetimes for these two complexes in water can be attributed to a combination of inefficient energy transfer (η_ET_ between 1–3%) and significantly increased nonradiative decay pathways for these two complexes (with *k*_nr_ values over 55 times larger than in methanol; see Table S7). The much higher *k*_nr_ values likely arise from a combination of photoinduced electron transfer (PET) from the electron‐rich quinoline units to the Eu(III) center, and energy transfer to O–H vibrational modes. The higher polarity of water may stabilize the charge‐separated excited state, exacerbating PET quenching compared to in methanol.

### 2.6 Buffer pH Analysis of Water‐Soluble Eu(III) Complexes

**Figure 7:**
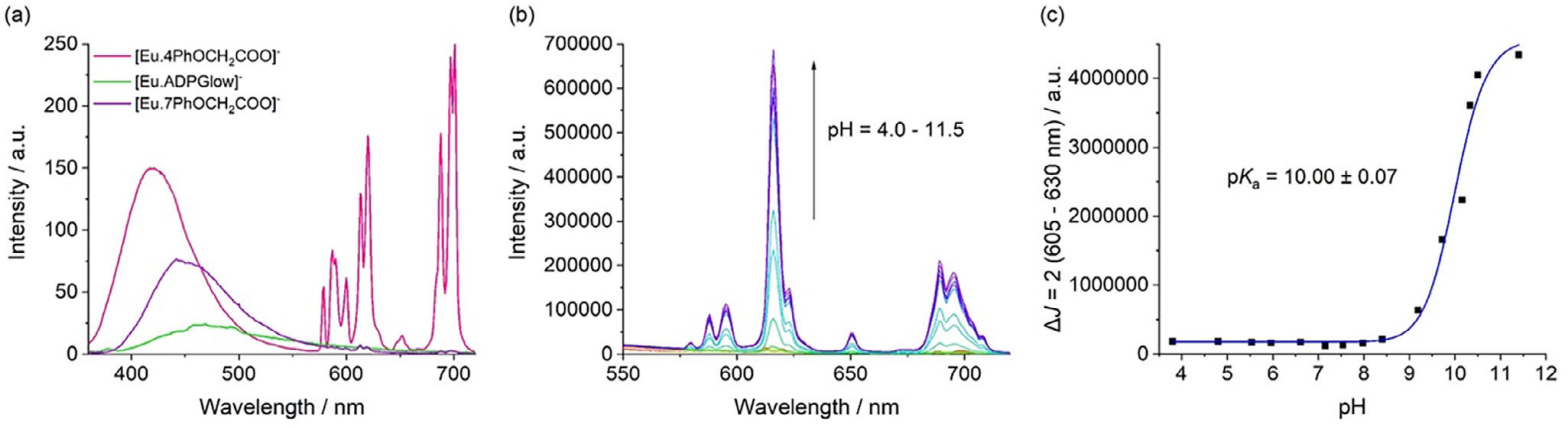
a) Emission spectra of the water‐soluble Eu(III) complexes (0.1 Abs) [**Eu.4PhOCH_2_COO**]^−^ (*λ*_exc_ 321 nm), [**Eu.ADPGlow**]^−^ (*λ*_exc_ 337 nm), and [**Eu.7PhOCH_2_COO**]^−^ (*λ*_exc_ 350 nm), measured in 10 mM HEPES buffer at pH 7.0, 295 K. b) pH titration of [**Eu.7PhOCH_2_COO**]^−^ showing the increase in emission intensity upon incremental addition of NaOH, where the pH was adjusted ∼0.5 pH unit. c) Plot of intensity of the Δ*J *= 2 emission band as a function of pH, showing the fit to the experimental data for [**Eu.7PhOCH_2_COO**]^−^. Measured in water, 295 K, 0.1 Abs, *λ*_exc_ 350 nm.

Next, pH titrations were carried out for [**Eu.4PhOCH_2_COO**]^−^ and [**Eu.7PhOCH_2_COO**]^−^ in water at 295 K. Complex [**Eu.4PhOCH_2_COO**]^−^ showed minimal change in emission intensity between pH 4 and 7.5 (Figure S13), with a marked increase between pH 8 and 10, giving an estimated pK_a_ of 8.55 ± 0.02. This pH profile closely matches that of the previously reported [**Eu.ADPGlow**]^−^ and is consistent with hydroxide binding to the Eu(III) center at higher pH. In contrast, [**Eu.7PhOCH_2_COO**]^−^ showed no change in emission from pH 4 to 9, with a significant increase only above pH 9, and a higher pK_a_ of 10.00 ± 0.07 (Figure 7). The elevated pK_a_ suggests that the coordinated water is less accessible or completely absent, due to a more compact and rigid ligand environment imposed by the 7‐substituted π‐conjugated groups. This interpretation is supported by the X‐ray crystal structure of the analogous [**Eu.7PhOMe**]⁺, which reveals dimerization and the absence of a coordinated water molecule in the TSAP isomer (Figure 3), as well as solution NMR data showing that the TSAP form predominates in solution, attributed to increased steric bulk at the 7‐position (Figure 4). Together, these findings indicate reduced accessibility of the Eu(III) center of [**Eu.7PhOCH_2_COO**]^−^, disfavoring OH^−^ (and water) coordination and increasing the pK_a_.

### 2.7 Anion Binding Studies

**Figure 8:**
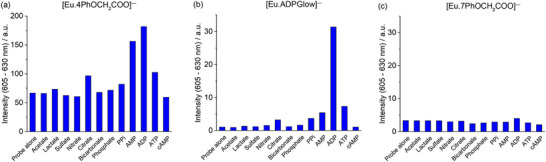
Selective emission enhancement of the Δ*J* = 2 (605 – 630 nm) of a) [**Eu.4PhOCH_2_COO**]^−^ (0.1 Abs, *λ*_exc_ 321 nm), b) [**Eu.ADPGlow**]^−^ (0.1 Abs, *λ*_exc_ 337 nm), and c) [**Eu.7PhOCH_2_COO**]^−^ (0.1 Abs, *λ*_exc_ 350 nm), with acetate, lactate, sulfate, nitrate, citrate, bicarbonate, phosphate, pyrophosphate (PPi), adenosine monophosphate (AMP), ADP, ATP, and cyclic adenosine monophosphate (cAMP) (1 mM each).

The anion binding behavior of the three phenoxyacetate‐substituted Eu(III) complexes was initially assessed by recording their emission spectra in the presence of biologically relevant anions (1 mM) in 10 mM HEPES at pH 7.0 (Figures S15–S17). The most pronounced emission response from [**Eu.4PhOCH_2_COO**]^−^ was observed with added ADP (Figure S15), which induced a threefold overall enhancement accompanied by changes in the spectral profiles of the Δ*J*  =  2 and Δ*J*  =  1 emission bands, consistent with ADP binding at the metal center and displacement of the coordinated water molecule. AMP also induced a twofold emission enhancement with two distinctive features emerging within the Δ*J*  =  2 band, while ATP and citrate produced much smaller changes. Almost no response was observed for pyrophosphate, phosphate, bicarbonate, acetate, lactate, sulfate, nitrate, and cyclic‐AMP (Figure 8). The presence of the adenosine moiety in ADP and AMP is clearly necessary for binding to [**Eu.4PhOCH_2_COO**]^−^, as neither pyrophosphate nor phosphate alone induces a significant emission change. The weak response to ATP further indicates that electrostatic attraction to the Eu(III) center is not the principal factor governing the observed quantum yield enhancements in the host–guest complexes with ADP or AMP.

Emission lifetime measurements in the absence and presence of 1 mM ADP confirmed that both [**Eu.4PhOCH_2_COO**]^−^ and [**Eu.ADPGlow**]^−^ bind ADP with displacement of the coordinated water molecule (Table S8). For [**Eu.7PhOCH_2_COO**]^−^, the very short lifetime in the absence of ADP precluded accurate determination of the hydration number. However, in the presence of 1 mM ADP, a *q* value of 0.4 was obtained, indicating weak binding, with only partial water displacement and the formation of an equilibrium mixture of species in solution.

Comparison of [**Eu.4PhOCH_2_COO**]^−^ and [**Eu.ADPGlow**]^−^ revealed two notable differences in anion response. First, ADP induced a significantly stronger and more selective emission enhancement with [**Eu.ADPGlow**]^−^, showing a 33‐fold increase in intensity, compared to the threefold enhancement observed with [**Eu.4PhOCH_2_COO**]^−^ (Figure 8). Second, the response toward AMP was small for [**Eu.ADPGlow**]^−^, whereas [**Eu.4PhOCH_2_COO**]^−^ exhibited a much greater response, suggesting more favorable binding. These observations provided an early indication that positioning the conjugated phenoxyacetate arms at the quinoline 4‐position facilitates AMP binding, likely through formation of a tighter host–guest complex and improved exclusion of coordinated and second‐sphere water molecules.

To verify this, anion titration experiments were conducted under identical conditions to those used for [**Eu.ADPGlow**]^−^. Apparent binding constants were determined by plotting the Δ*J* = 2/Δ*J* = 1 emission band intensity ratio as a function of anion concentration, followed by curve fitting using a 1:1 binding model (Table 2; Figures S18–S21). Of the four anions that induced an emission response with [**Eu.4PhOCH_2_COO**]^−^, the strongest binding was observed for ADP (log *K*_a_ = 3.38), while ATP and citrate showed weaker affinities (log *K*_a_ = 2.70–2.80). AMP bound with intermediate strength (log *K*_a_ = 3.09), comparable to ATP.

**Table 2:** Apparent binding constants (log *K*_a_) for Eu(III) complexes in the presence of selected anions, measured in 10 mM HEPES at pH 7.0 and 295 K.

Anion	[Eu.4PhOCH_2_COO]^−^	[Eu.ADPGlow]^−^
AMP	3.09 ± 0.02	2.68 ± 0.07
ADP	3.38 ± 0.03	3.77 ± 0.02
ATP	2.79 ± 0.06	3.33 ± 0.04
Citrate	2.71 ± 0.09	3.12 ± 0.07

Compared to the lead probe [**Eu.ADPGlow**]^−^, [**Eu.4PhOCH_2_COO**]^−^ binds ADP less strongly but exhibits notably stronger binding to AMP. These data suggest that the 4‐substituted regioisomer [**Eu.4PhOCH_2_COO**]^−^ presents a more open binding cavity, allowing the nucleobase and phosphate group of AMP to approach the Eu(III) center more closely. In contrast, the extended conjugation at the 6‐position in [**Eu.ADPGlow**]^−^ creates a more sterically constrained binding site that disfavors AMP binding but enables selective engagement of the longer ADP molecule, as previously supported by DFT and NMR analysis. The 6‐substituted framework of [**Eu.ADPGlow**]^−^ achieves geometric complementarity for ADP through a combination of metal–ligand coordination and π–π stacking interactions with the extended aromatic arms. ADP binding likely suppresses multiple quenching pathways, including water displacement and reduced PET via π–π stacking of the electron‐rich quinoline and the adenine ring. This dual effect results in a strong luminescence “switch‐on” response (Figure 8). In contrast, the more flexible, open geometry of [**Eu.4PhOCH_2_COO**]^−^ accommodates both AMP and ADP, with each displacing water and producing comparatively smaller emission enhancements.

In contrast to [**Eu.4PhOCH_2_COO**]^−^, the 7‐substituted analogue [**Eu.7PhOCH_2_COO**]^−^ did not exhibit any significant increase in emission intensity upon addition of the tested anions (Figure 8). A minor enhancement was observed with ADP, accompanied by only a slight change in spectral shape (Figure S17), but the overall luminescence was too weak to reliably determine a binding constant. The absence of meaningful anion binding is consistent with the increased steric hindrance introduced by the 7‐position phenoxyacetate substituents, which evidently restrict access to the Eu(III) center. This interpretation is supported by the higher p*K*_a_ observed in the pH titration profile, the distinctive X‐ray crystal structure, and the predominantly TSAP coordination geometry observed in the NMR spectrum. Together with the contrasting behavior of [**Eu.4PhOCH_2_COO**]^−^ and [**Eu.ADPGlow**]^−^, these results underscore the influence of substitution pattern on the steric and geometric properties of the appended arms, which in turn modulate host–guest recognition. Whereas 4‐substitution permits a more open and accessible coordination environment, enabling binding of both AMP and ADP, 7‐substitution imposes steric constraints that suppress binding altogether.

## 3 Conclusion

We have synthesized four new Eu(III) complexes to investigate how varying the position of the π‐conjugated antennae influences the steric and geometric features of the phosphoanion binding site, thereby modulating selectivity. The 4‐substituted complex [**Eu.4PhOCH_2_COO**]^−^ generated a more open binding site, accommodating a wider range of phosphoanions, while the 7‐substituted complex [**Eu.7PhOCH_2_COO**]^−^ induced too much steric hindrance, effectively closing the binding cavity and preventing access to all anions, including ADP.

Anion titration experiments revealed that [**Eu.4PhOCH_2_COO**]^−^ exhibited preferential ADP binding (log *K*_a_ = 3.38), resulting in a threefold increase in Eu(III) emission, with minimal interference from bicarbonate and inorganic phosphate. However, significant AMP binding (log *K*_a_ = 3.09) resulted in a twofold emission increase, causing interference with ADP recognition. ATP and citrate induced smaller emission increases, with binding affinities less than one order of magnitude lower than for ADP. In contrast, the 7‐substituted [**Eu.7PhOCH_2_COO**]^−^ complex displayed no response to any anions, including ADP, attributed to increased steric demand around the Eu(III) center, which effectively blocked anion binding.

These findings further explain why the previously reported 6‐substituted complex, [**Eu.ADPGlow**]^−^, achieves optimal geometric complementarity for ADP. [**Eu.ADPGlow**]^−^ provides a suitably open binding site that engages the guest through both metal–ligand coordination and π–π stacking with the elongated arms. This design achieves a preferential balance of steric constraint and binding site accessibility, enabling highly selective ADP recognition in aqueous media. More broadly, these results reinforce the importance of tailoring host geometry to match the size, shape, and binding mode of the target anion; a central design principle in the development of selective lanthanide probes, and indeed any synthetic receptor, for biological applications.

## Supporting Information

The authors have cited additional references within the Supporting Information.^[^ ^42^, ^43^, ^44^, ^45^ ^]^

## Conflict of Interest

The authors declare no conflict of interest.

## Supporting information



Supporting Information

Supporting Information

## Data Availability

The data that support the findings of this study are available in the supplementary material of this article.

## Footnotes

1 Deposition Number(s) 2464248 (for [**Eu.4PhOMe**]^+^), 2464162 (for compound 3a), and 2464160 (for [**Eu.4PhOMe**]^+^), contain the supplementary crystallographic data for this paper. These data are provided free of charge by the joint Cambridge Crystallographic Data Center and Fachinformationszentrum Karlsruhe Access Structures service.
