# Supplementary material for: Steric and Geometric Tuning of π‐Conjugated Antennae in Europium(III) Complexes for Selective ADP Recognition
Source: Chemistry. 2025 Aug 22;31(53):e02251. doi: 10.1002/chem.202502251 (PMC12451422; doi:10.1002/chem.202502251)
Supplement: Supplementary file 1 — Supporting Information [file CHEM-31-e02251-s002.pdf]

*Electronic Supplementary Information (ESI)*

# Steric and geometric tuning of π-conjugated antennae in europium(III) complexes for selective ADP recognition

Samantha E. Bodman and Stephen J. Butler

*Department of Chemistry, Loughborough University, Epinal Way, Loughborough, LE11 3TU, UK*

**Contents**

1. Materials and Methods S2
2. Synthesis and Characterisation of Compounds and Europium(III) Complexes S5
3. X-ray Crystallography S20
4. $^{1}$H NMR Studies of Eu(III) Complexes S29
5. Photophysical Measurements of Eu(III) Complexes. S30
6. References S36

Appendix 1: $^{1}$H NMR and $^{13}$C NMR of Compounds and Complexes S37
Appendix 2: High-resolution and Low-resolution Mass Spectra S67
Appendix 3: X-ray Crystallography Data Tables S83

## 1. Materials and Methods

*General Considerations*

Reagent grade chemicals, including the anhydrous solvents, were purchased from Sigma Aldrich and Fluorochem and used without further purification.

*Nuclear Magnetic Resonance*

$^{1}H$, $^{13}C$, COSY, HMQC and HMBC NMR spectra were recorded in the stated deuterated solvent on a JEOL ECS-400 or 500 spectrometers ($^{1}H$ at 400 MHz, $^{13}C$ at 101 MHz; $^{1}H$ at 500 MHz, $^{13}C$ at 126 MHz, respectively), at 298 K. Chemical shifts are expressed in ppm and are adjusted to the chemical shift of the residual NMR solvent resonances ($CDCl_3$: $^{1}H$ δ = 7.26 ppm, $^{13}C$ δ = 77.16 ppm, $CD_3OD$: $^{1}H$ δ = 3.31 ppm, $^{13}C$ δ = 49.00 ppm or DMSO-$d_6$: $^{1}H$ δ = 2.50 ppm, $^{13}C$ δ = 39.52 ppm). The coupling constants are expressed in Hz.

*Liquid Chromatography Mass Spectrometry*

Liquid Chromatography Electrospray Mass spectra were recorded on a Shimadzu Prominence LC system with a Shimadzu SPDM20A Photodiode Array Detector, a Shimazu CTO-20A column oven, Shimadzu SIL-20A autosampler and a Shimadzu LCMS 20 mass spectrometer controlled using LabSolutions software. The system operates in positive ion mode, with acetonitrile as the carrier solvent. The flow rate was maintained at 0.7 mL/min over a gradient of 5 to 95% acetonitrile in water (0.1% formic acid) for 10 minutes. High resolution mass spectra were recorded using a Thermofisher Q-Exactive orbitrap mass spectrometer.

*Column Chromatography*

Column chromatography was performed using flash silica gel 60 (particle size 40–63 microns) purchased from Apollo Scientific. Thin layer chromatography (TLC) was performed on aluminium sheet silica gel plates with 0.2 mm thick silica gel 60 F254 using the stated mobile phase.

*High Performance Liquid Chromatography*

Preparative RP-HPLC was performed using a Waters 2489 UV/Visible detector performed at 254 nm, a Waters 1525 Binary HPLC pump controlled by the Waters Breeze 2 HPLC system software. Separation was achieved using a semi-preparative XBridge C18 (5 μm OBD 19 × 100 mm) column at a flow rate maintained at 17 mL/min). A solvent system composed of either water (0.1% formic acid)/methanol (0.1% formic acid) or water (50 mM $NH_4HCO_3$)/acetonitrile was used over the stated linear gradient (usually 0 to 100% organic solvent over 17 - 25 mins). Analytical RP-HPLC was performed using a XBridge C18 5 μm 4.6 × 100 mm at a flow rate maintained at 2.0 mL/min using the same gradients and solvents.

*Luminescence Experiments*

Luminescence spectra were recorded on a Camlin Photonics luminescence spectrometer with FluoroSENS version 3.4.7.2024 software. Emission spectra were obtained using a 40 μL or 100 μL Hellma Analytics quartz cuvettes. Excitation light was set at the absorption maxima and emission recorded in the range 400 – 720 nm using an integration time of 0.5 seconds, increment of 1.0 nm, excitation slit of 0.2 nm and emission slit of 0.5 nm. Quantum yields were measured using quinine sulfate in 0.05 M $H_2SO_4$ as a standard ($\Phi_{em}$= 0.60, $\lambda_{ex}$ = 350 nm).[1] Emission lifetime measurements were performed on the FluoroSENS instrument. Measurements

were taken of 1 mL of 0.1 absorbance samples of Eu(III) complexes in 10 mM HEPES at pH 7.0, unless stated otherwise. Measurements were obtained by indirect excitation of the Eu(III) ion via the quinoline antennae using a short pulse of light at 321 nm ([**Eu.4PhOMe**]$^+$ and [**Eu.4PhOCH$_2$COO**]$^-$), 348 nm ([**Eu.7PhOMe**]$^+$) and 350 nm ([**Eu.7PhOCH$_2$COO**]$^-$) followed by monitoring the integrated intensity of the light emitted at 615 nm, with 500 data points collected over a 10 millisecond time period. The decay curves were plotted in Origin Labs 2019 version 9.6.0.172, and fitted to the equation:

$$\mathrm{I} = \mathrm{A0} + \mathrm{A1}\, e^{-kt}$$

where I is the intensity at time, t, following excitation, $A_0$ is the intensity when decay has ceased, $A_1$ is the pre-exponential factor and k is the rate constant for the depopulation of the excited state.

The hydration state, q, of the Eu(III) complexes was determined using the modified Horrocks equation:[1]

$$\mathrm{q\,(Eu)} = 1.2\,(\frac{1}{\tau\mathrm{H_2O}} - \frac{1}{\tau\mathrm{D_2O}} - 0.25 - 0.075\mathrm{n})$$

where $\tau_{H2O}$ and $\tau_{D2O}$ are the emission lifetime times in water and $D_2O$, respectively, and n is the number of carbonyl-bound amide NH groups.

The methanol hydration state, *m*, of the Eu(III) complexes was determined using:

$$m\,\mathrm{(Eu)} = 2.1\,(\frac{1}{\tau\mathrm{CH_3OH}} - \frac{1}{\tau\mathrm{CD_3OD}})$$

where $\tau_{CH3OH}$ and $\tau_{CD3OD}$ are the emission lifetime times in methanol and methanol-*d$_4$*, respectively.

*Determination of photophysical parameters*

The overall quantum yield of sensitised Eu(III) emission ($\Phi_{em}$) reflects the efficiency of three sequential processes: light absorption by the antenna, energy transfer to the Eu(III) excited state, and Eu(III)-centred emission. This can be described as the product of two key terms:

$$\Phi_{\mathrm{em}} = \Phi_{\mathrm{Ln}} \times \eta_{\mathrm{ET}}$$

where $\Phi_{Ln}$ is the intrinsic quantum yield of Eu(III) luminescence, i.e. the efficiency with which the excited $^5D_0$ state decays via photon emission, and $\eta_{ET}$ is the efficiency of energy transfer from the antenna to the Eu(III) ion. $\Phi_{Ln}$ is obtained from the ratio of the observed emission lifetime ($\tau_{obs}$) to the radiative lifetime ($\tau_{rad}$):

$$\Phi_{\mathrm{Ln}} = \frac{\tau_{\mathrm{obs}}}{\tau_{\mathrm{rad}}}$$

The radiative lifetime, $\tau_{rad}$, can be calculated from the corrected Eu(III) emission spectrum using the following equation:

$$\frac{1}{\tau_{\mathrm{rad}}} = \mathrm{A_{MD,0}} \times n^3 \times (\frac{\mathrm{I_{tot}}}{\mathrm{I_{MD}}})$$

where, $A_{MD,0}$ is the spontaneous emission probability of the $^5D_0 \rightarrow {}^7F_1$ magnetic dipole (MD) transition (14.65 $s^{-1}$ in vacuo), *n* is the refractive index of the medium (1.333 for water, 1.328 for methanol), $I_{tot}$ is the total integrated Eu(III) emission (550–720 nm), and $I_{MD}$ is the integrated intensity of the MD $^5D_0 \rightarrow {}^7F_1$ transition (580–600 nm). This method assumes the $^5D_0 \rightarrow {}^7F_1$ transition is independent of the ligand field and complex symmetry and can be used as an internal reference to estimate $\tau_{rad}$.[2,3]

With $\tau_{obs}$ determined experimentally, and $\tau_{rad}$ calculated, the radiative ($k_r$) and sum of non-radiative ($\Sigma k_{nr}$) rate constants can also be determined:

$$k\mathrm{r} = \frac{1}{\tau_{\mathrm{rad}}}$$

$$\Sigma k_{\mathrm{nr}} = \left(\frac{1}{\tau_{\mathrm{obs}}}\right) - k_{\mathrm{r}}$$

These parameters provide insight into the competition between radiative decay and non-radiative pathways in each coordination environment. For instance, a high $k_{nr}$ suggests significant quenching processes, such as energy transfer to vibrational modes (e.g. O–H or N–H oscillators), photoinduced electron transfer from the ligand to the metal, or other non-radiative pathways.

In the present study, application of equations above allowed all relevant parameters including $\tau_{rad}$, $\Phi_{Ln}$, $\eta_{ET}$, $k_r$ and $\Sigma k_{nr}$ to be extracted from experimental data.

*Anion Binding Titrations*

Anion binding titrations were carried out in duplicate in degassed 10 mM HEPES buffer at pH 7.0 for complexes $[Eu.4PhOCH_2COO]^-$ and $[Eu.7PhOCH_2COO]^-$. Stock solutions of anions (e.g. inorganic phosphate, ADP) containing Eu(III) complex (0.1 Abs) were made up at 0.4, 4 and 40 mM anion. The appropriate anion stock solution was added incrementally to 100 µL of Eu(III) complex (0.1 Abs) and the emission spectrum was recorded after each addition. The ratio of emission bands 605–630 nm/585–600 nm ($\Delta J = 2$ / $\Delta J = 1$) was plotted as a function of anion concentration. The data was analysed using a nonlinear least-squares curve fitting procedure, based on a 1:1 binding model described by the equation:

$$\mathrm{FB} = \frac{\frac{1}{\mathrm{K_a}} + [\mathrm{A}] + [\mathrm{Eu}] - \sqrt{(\frac{1}{\mathrm{K_a}} + [\mathrm{A}] + [\mathrm{Eu}])^2 + 4[\mathrm{A}][\mathrm{Eu}]}}{2[\mathrm{Eu}]}$$

where FB is the fraction bound, calculated by $(I-I_0)/(I_1-I_0)$ where I is the emission intensity at [A], $I_0$ is the initial emission intensity, and $I_1$ is the final emission intensity. [A] is the total concentration of anion in solution, [Eu] is the total concentration of Eu(III) complex, $K_a$ is the apparent binding constant.

*pH Titrations*

A solution of Eu(III) complex (0.1 Abs) in water was adjusted to pH 11.0 by the addition of 1 M NaOH and an emission spectrum recorded. The pH was decreased slowly by 0.2 – 0.5 units by the addition of 1 M or 0.1 M HCl solution and an emission spectrum recorded at each pH. The ratio of emission bands 605–630 nm/ 585–600 nm ($\Delta J = 2$ / $\Delta J = 1$) was plotted as a function of pH and fitted to a sigmoidal curve using OriginLab 2019 to determine the p$K_a$ value.

*X-ray Crystallography*

Single crystal X-ray diffraction experiments were performed by the UK National Crystallography Service on a Rigaku FRE+ diffractometer with HF Varimax confocal mirrors, and a UG2 goniometer and HyPix 6000HE detector. The crystals were collected at 100(2) K. The structure was solved by direct methods using ShelXT[4] and refined with ShelXL[5] using a least squares method. Olex2 software[6] was used as the solution, refinement and analysis program.

## 2. Synthesis and Characterisation of Europium(III) Complexes

Compound 7-bromoquinaldine and complexes [**Eu.6PhOMe**]$^+$ and [**Eu.ADPGlow**]$^-$ were synthesised using literature procedures.[7,8] All other chemicals were from commercial sources (Sigma Aldrich, Fluorochem, or Alfa Aesar) and used as received.

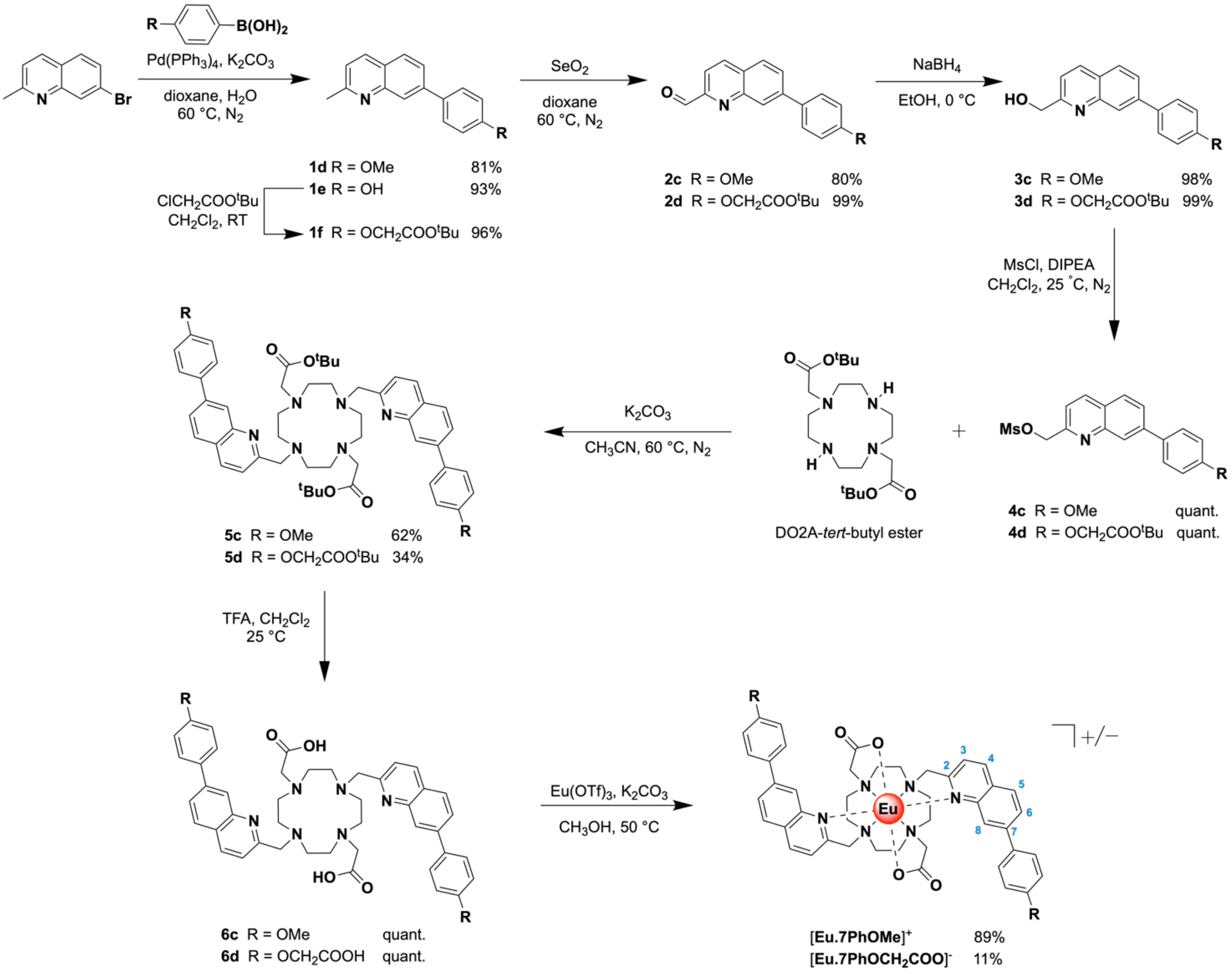


**Scheme S1:** Synthesis of complexes [**Eu.7PhOMe**]$^+$ and [**Eu.7PhOCH$_2$COO**]$^-$.

<u>4-(4-Methoxyphenyl)-2-methylquinoline (**1a**)</u>

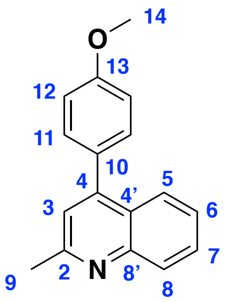


4-Bromoquinaldine (0.60 g, 2.70 mmol), 4-methoxyphenylboronic acid (0.49 g, 3.24 mmol) and potassium carbonate (1.12 g, 8.11 mmol) ere added to an oven-dried Schlenk with a condenser attached. Anhydrous dioxane (24 mL) and oxygen-free water (6 mL) were added, followed by palladium-tetrakis(triphenylphosphine) (0.31 g, 0.27 mmol) and the reaction was heated to 60 °C for 16 hours. The reaction was cooled to room

temperature, filtered through celite and the filtrate was removed under reduced pressure. The residue was dissolved in dichloromethane (50 mL) then washed with water (50 mL). The organic layer was separated, and the aqueous layer washed with $CH_2Cl_2$ (3 x 50 mL). The organic layers combined, washed with brine (100 mL), dried with $MgSO_4$ and solvent evaporated under reduced pressure. The product was obtained after column chromatography (silica gel; neat dichloromethane to 1:9 ethyl acetate/dichloromethane) to give the product as a white solid (0.60 g, 89%). $^1$H NMR (500 MHz, $CDCl_3$): δ 8.07 (1H, d, *J* = 8.2 Hz, $H^8$), 7.90 (1H, d, *J* = 8.5 Hz, $H^5$), 7.67 (1H, dtd, *J* = 8.2 Hz, 6.6 Hz, 1.3 Hz, $H^7$), 7.44-7.41 (3H, m, $H^{11}$, $H^6$), 7.21 (1H, s, $H^3$), 7.04 (2H, dd, *J* = 6.6 Hz, 2.1 Hz, $H^{12}$), 3.89 (3H, s, $H^{14}$), 2.76 (3H, s, $H^9$). $^{13}$C NMR (126 MHz, $CDCl_3$): δ 159.9 ($C^{13}$), 158.6 ($C^2$), 148.6 ($C^{8'}$), 148.3 ($C^4$), 130.8 ($C^{11}$), 130.5 ($C^{10}$), 129.3 ($C^7$), 129.1 ($C^8$), 125.8 ($C^5$), 125.7 ($C^6$), 125.4 ($C^{4'}$), 122.2 ($C^3$), 114.1 ($C^{12}$), 55.5 ($C^{14}$), 25.4 ($C^9$). ESI-MS (*m/z)*: Found [M + H]$^+$ 250.1226, calc [$C_{17}H_{15}NO$ + H]$^+$ 250.1226.

4-(4-Methoxyphenyl)-2-quinolinecarboxaldehyde (**2a**)

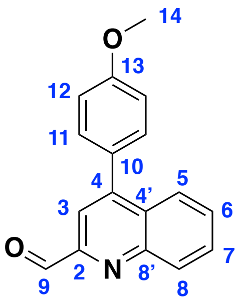


4-(4-Methoxyphenyl)-2-methylquinoline (0.49 g, 1.98 mmol) and anhydrous dioxane (20 mL) were added to an oven-dried glassware under a nitrogen atmosphere. Selenium dioxide (0.45 g, 4.07 mmol) was added as one solid portion, and the reaction was heated to 80 °C overnight. The reaction was cooled to room temperature then brine (20 mL) and ethyl acetate (30 mL) were added. The biphasic mixture was passed through celite then the organic layer was separated. The aqueous layer was extracted with ethyl acetate (3 x 30 mL), organic layers combined, dried ($MgSO_4$) and the solvent removed under reduced pressure to give the pure product as a pale-yellow solid (0.501 g, 96%). $^1$H NMR (500 MHz, $CDCl_3$): δ 10.27 (1H, s, $H^9$), 8.31 (1H, d, *J* = 8.2 Hz, $H^8$), 8.06 (1H, d, *J* = 7.9 Hz, $H^5$), 7.96 (1H, s, $H^3$), 7.82 (1H, t, *J* = 7.1 Hz, $H^7$), 7.64 (1H, *J* = 7.1 Hz, $H^6$), 7.48 (2H, d, *J* = 8.8 Hz, $H^{11}$), 7.08 (2H, d, *J* = 8.8 Hz, $H^{12}$), 3.91 (3H, s, $H^{14}$). $^{13}$C NMR (126 MHz, $CDCl_3$): δ 194.1 ($C^9$), 160.3 ($C^{13}$), 152.2 ($C^2$), 149.8 ($C^4$), 148.7 ($C^{8'}$), 131.0 ($C^{11}$), 130.9 ($C^8$), 130.3 ($C^7$), 129.8 ($C^{10}$), 129.2 ($C^6$), 128.8 ($C^{4'}$), 126.2 ($C^5$), 117.5 ($C^3$), 114.3 ($C^{12}$), 55.5 ($C^{14}$). ESI-MS (*m/z)*: Found [M + H]$^+$ 264.1020, calc [$C_{17}H_{13}NO_2$ + H]$^+$ 264.1019.

4-(4-Methoxyphenyl)-2-quinolinemethanol (**3a**)

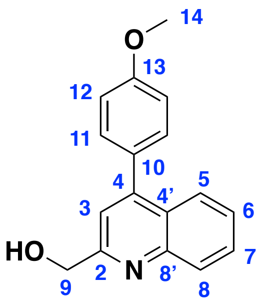

4-(4-Methoxyphenyl)-2-quinolinecarboxaldehyde (0.50 g, 1.90 mmol) was dissolved in anhydrous methanol (30 mL) under a nitrogen atmosphere and cooled to 0 °C. Sodium borohydride (0.09 g, 2.28 mmol) was carefully added as one solid portion, then the reaction was allowed to warm to room temperature. The reaction was quenched with $NH_4Cl$ solution (30 mL) then the solvent evaporated under reduced pressure. The aqueous solution was extracted with chloroform (3 x 50 mL), organics combined, washed with brine (100 mL), dried ($MgSO_4$) and the solvent evaporated under reduced pressure to yield the product was a yellow solid (0.40 g, 80%). $^1$H NMR (500 MHz, $CDCl_3$): δ 8.13 (1H, d, *J* = 8.2 Hz, $H^8$), 7.94 (1H, d, *J* = 8.2 Hz, $H^5$), 7.72 (1H, t, *J* = 6.9 Hz, $H^7$), 7.49 (1H, t, *J* = 7.6 Hz, $H^6$), 7.44 (2H, dt, *J* = 9.3 Hz, 2.5 Hz, $H^{11}$), 7.21 (1H, s, $H^3$), 7.06 (2H, dt, *J* = 9.2 Hz, 2.4 Hz, $H^{12}$), 4.94 (2H, s, $H^9$), 3.90 (3H, s, $H^{14}$). $^{13}$C NMR (126 MHz, $CDCl_3$): δ 160.1 ($C^{13}$), 158.6 ($C^2$), 149.2 ($C^4$), 147.4 ($C^{8'}$), 130.8 ($C^{11}$), 130.2 ($C^{10}$), 129.7 ($C^7$), 129.1 ($C^8$), 126.5 ($C^{4'}$), 126.4 ($C^6$), 126.1 ($C^5$), 118.5 ($C^3$), 114.2 ($C^{12}$), 64.2 ($C^9$), 55.5 ($C^{14}$). ESI-MS (*m/z)*: Found $[M + H]^+$ 266.1174, calc $[C_{17}H_{15}NO_2 + H]^+$ 266.1176.

4-(4-Methoxyphenyl)- 2-methanesulfonate-2-quinolinemethanol (**4a**)

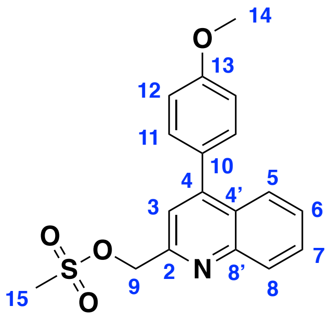


4-(4-Methoxyphenyl)-2-quinolinemethanol (0.35 g, 1.32 mmol) was dissolved in anhydrous dichloromethane (8 mL) then diisopropylethylamine (0.35 mL, 1.98 mmol) and methanesulfonyl chloride (0.20 mL, 1.29 mmol) was added. The mixture was stirred at room temperature under a nitrogen atmosphere for four hours. Water (20 mL) was added, and the organic layer separated. The aqueous solution was extracted with chloroform (3 x 20 mL), organic layers combined, washed with brine (100 mL), dried ($MgSO_4$) and concentrated under reduced pressure to give the product as a dark orange solid, which was used immediately in the next step (0.44 g, quant.). $^1$H NMR (500 MHz, $CDCl_3$): δ 8.11 (1H, d, *J* = 8.5 Hz, $H^8$), 7.97 (1H, d, *J* = 8.4 Hz, $H^5$), 7.74 (1H, t, *J* = 8.2 Hz, $H^7$), 7.54 – 7.45 (5H, m, $H^6$, $H^3$, $H^{11}$), 7.06 (2H, d, *J* = 8.5 Hz, $H^{12}$), 5.52 (2H, s, $H^9$), 3.86 (3H, s, $H^{14}$), 3.13 (3H, s, $H^{15}$). $^{13}$C NMR (126 MHz, $CDCl_3$): δ 160. 2 ($C^{13}$), 153.4 ($C^2$), 149.8 ($C^4$), 148.3 ($C^{8'}$), 130.9 ($C^{11}$), 130.0 ($C^7$), 129.8 ($C^{10}$), 129.7 ($C^8$), 127.2 ($C^6$), 126.6 ($C^{4'}$), 126.0 ($C^5$), 119.8 ($C^3$), 114.3 ($C^{12}$), 72.3 ($C^9$), 56. 5 ($C^{14}$), 38.2 ($C^{15}$). LRMS-ESI (*m/z)*: Found $[M + H]^+$ 344.1, calc $[C_{18}H_{17}NO_4S + H]^+$ 344.1.

4,10-*Bis*((4-(4-methoxyphenyl)-quinolin-2-yl)-methyl)-1,4,7,10-tetraazacyclododecane-1,7-diyl)-diacetate (**5a**)

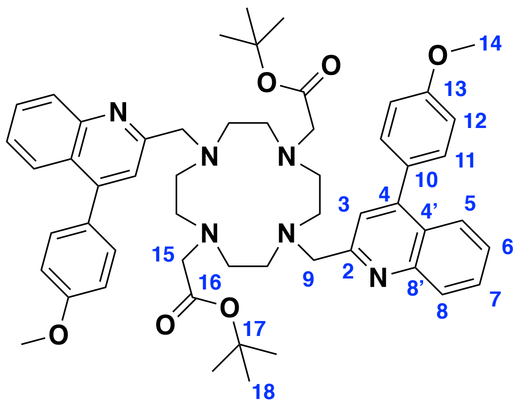


To a solution of DO2A-*tert*-butyl ester (0.17 g, 0.43 mmol) and potassium carbonate (0.06 mg, 0.63 mmol) in anhydrous acetonitrile (10 mL), was added 4-(4-methoxyphenyl)-2-methanesulfonate-2-quinolinemethanol (0.40 mg, 0.63 mmol). The reaction mixture was stirred at 60 °C for 18 hours. The reaction was cooled to room temperature, salts removed through centrifugation (1500 rpm for 5 minutes). The organic layer was removed, and the salts washed with acetonitrile (2 x 10 mL). The organic layers combined, and the solvent removed under reduced pressure. The crude material was purified by column chromatography (silica gel; neat dichloromethane to 95:5 dichloromethane/methanol) to give the desired protected ligand, as a yellow solid (0.112 g, 69%). $^{1}$H NMR (500 MHz, $CD_3OD$): δ 8.08 (2H, d, *J* = 8.5 Hz, $H^8$), 7.95 (2H, d, *J* = 7.6 Hz, $H^5$), 7.50 (4H, d, *J* = 8.8 Hz, $H^{11}$), 7.44–7.42 (4H, m, $H^6$, $H^3$), 7.33 (2H, t, *J* = 7.1 Hz, $H^7$), 7.12 (4H, d, *J* = 8.5 Hz, $H^{12}$), 4.84 (4H, s, $H^9$), 3.89 (6H, s, $H^{14}$), 3.21–2.57 (20H, m, $H^{15}$, $H^{cyclen}$), 1.18 (18H, s, $H^{18}$). $^{13}$C NMR (126 MHz, $CD_3OD$): δ 172.6 ($C^{16}$), 160.4 ($C^{13}$), 159.0 ($C^2$), 149.3 ($C^4$), 148.5 ($C^{8'}$), 130.7 ($C^{11}$), 129.8 ($C^8$), 129.4 ($C^{10}$), 129.3 ($C^7$), 126.0 ($C^6$, $C^{4'}$), 125.5 ($C^5$), 121.6 ($C^3$), 114.1 ($C^{12}$), 81.9 ($C^{17}$), 60.0 ($C^9$), 57.5 ($C^{15}$), 54.6 ($C^{14}$), 50.9 ($C^{cyclen}$), 48.6 ($C^{cyclen}$), 27.1 ($C^{18}$). ESI-MS (*m/z)*: Found $[M + 2H]^{2+}$ 448.2593, calc $[C_{54}H_{66}N_6O_6 + 2H]^{2+}$ 448.2595; Found $[M + H]^+$ 895.5115, calc $[C_{54}H_{66}N_6O_6 + H]^+$ 895.5117; Found $[M + Na]^+$ 917.4937, calc $[C_{54}H_{66}N_6O_6 + Na]^+$ 917.4936.

4,10-*Bis*((4-(4-methoxyphenyl)-quinolin-2-yl)-methyl)-1,4,7,10-tetraazacyclododecane-1,7-diyl)-diacetic acid (**6a**)

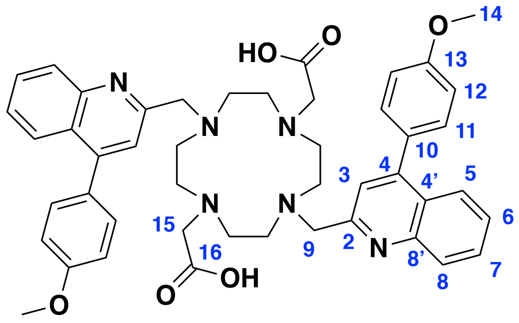


To a solution of 4,10-*bis*((4-(4-methoxyphenyl)-quinolin-2-yl)-methyl)-1,4,7,10-tetraazacyclododecane-1,7-diyl)-diacetate (73 mg, 81 μmol) in dichloromethane (2 mL) was added trifluoroacetic acid (2 mL). The reaction mixture was stirred at room temperature for 4 hours and the trifluoroacetic acid was co-evaporated with dichloromethane (5 x 25 mL) to give the deprotected ligand as a yellow solid (82 mg, 99%). $^{1}$H NMR (500 MHz, $CD_3OD$): δ 8.21 (2H, d, *J* = 8.5 Hz, $H^8$), 8.02 (2H, d, *J* = 8.5 Hz, $H^5$), 7.55–7.53 (6H, m, $H^6$, $H^{11}$), 7.48 (2H, s, $H^3$), 7.34 (2H, t, *J* = 7.3 Hz, $H^7$), 7.16 (4H, d, *J* = 8.5 Hz, $H^{12}$), 5.06 (4H, s, $H^9$), 3.92–3.90 (6H, m, $H^{14}$), 3.86–

3.78 (12H, br m, $H^{15}$, $H^{cyclen}$), 3.37–3.31 (8H, m, $H^{cyclen}$). $^{13}C$ NMR (101 MHz, $CD_3OD$): δ 172.6 ($C^{16}$), 160 7 ($C^{13}$), 150.8 ($C^{2}$), 150.6 ($C^{4}$), 147.5 ($C^{8'}$), 130.6 ($C^{11}$), 130.0 ($C^{7}$), 129.3 ($C^{8}$), 129.2 ($C^{10}$), 127.1 ($C^{6}$), 126.4 ($C^{4'}$), 125.7 ($C^{5}$), 119.7 ($C^{3}$), 114.0 ($C^{12}$), 58.4 ($C^{9}$), 54.6 ($C^{14}$), 52.8 ($C^{15}$), 51.8 ($C^{cyclen}$), 48.2 ($C^{cyclen}$). ESI-MS (*m/z)*: Found $[M + H]^+$ 783.3864, calc $[C_{46}H_{50}N_6O_6 + H]^+$ 783.3865; Found $[M + 2H]^{2+}$ 392.1967, calc $[C_{46}H_{50}N_6O_6 + 2H]^{2+}$ 392.1969.

**[Eu.4PhOMe]$^+$**

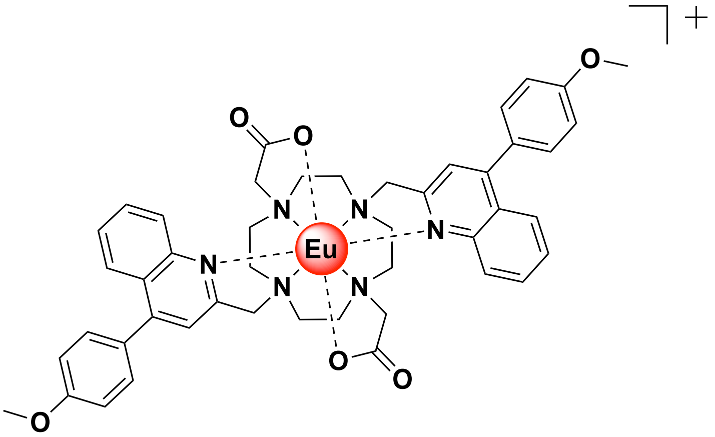

The deprotected ligand, **6a** (82 mg, 0.08 mmol) was dissolved in methanol (2 mL). Potassium carbonate (18 mg, 0.13 mmol) and europium(**III**) trifluoromethanesulfonate (156 mg, 0.261 mmol) were added and the reaction heated to 50 °C for 18 hours. The reaction was cooled to room temperature, filtered and the solvent evaporated under reduced pressure. The crude material was purified by column chromatography (silica gel; neat dichloromethane to 4:1 dichloromethane/methanol) to give the desired complex as a pale-yellow solid (87 mg, 99%). $^1H$ NMR (500 MHz, $CD_3OD$): δ 51.8, 30.8, 17.5, 17.1, 16.9, 13.1, 12.0, 11.7, 11.6, 11.2, 9.8, 9.3, 9.0, 7.8, 6.6, 6.0, 5.9, 5.2, 5.1, 4.9, 3.6, 2.1, 2.0, 1.6, 1.3, 1.1, 0.9, 0.8, 0.1, 0.0, -1.3, -1.7, -2.2, -2.6, -5.6, -9.4, -10.8, -11.3, -16.6, -16.7, -17.8, -28.3, -29.5, -30.3, -34.0. ESI-MS (*m/z*): Found $[M]^+$ 933.2844, calc $[C_{46}H_{48}EuN_6O_6]^+$ 933.2842. Photophysical data measured in methanol: $\lambda_{max}$ = 321 nm, $\varepsilon$ = 22400 $M^{-1}$ $cm^{-1}$, $\Phi_{em}$ = 5.5%, $\tau_{CH3OH}$ = 0.859 ms, $\tau_{CD3OD}$ = 1.341 ms, $m$ = 0.9.

4-(2-Methyl-4-quinolinyl)-phenol (**1b**)

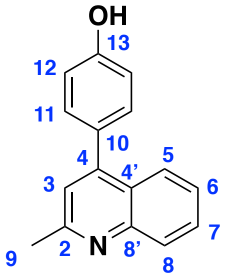


4-Bromoquinaldine (0.50 g, 2.24 mmol), 4-hydroxyphenyl boronic acid (0.46 g, 3.36 mmol) and potassium carbonate (0.62 g, 4.48 mmol) were added to an oven-dried Schlenk with a condenser attached. Anhydrous dioxane (20 mL) and oxygen-free water (5 mL) were added, followed by palladium-tetrakis(triphenylphosphine) (0.26 g, 0.22 mmol) and the reaction was heated to 60 °C for 18 hours. The reaction as cooled to room temperature, filtered through celite and the filtrate was removed under reduced pressure. The residue was taken up in ethyl acetate (100 mL) and solid collected through vacuum filtration and allowed to dry to give the product as an off-white solid (0.50 g, 94%). $^1H$ NMR (500 MHz, DMSO-$d_6$): δ 9.79 (1H, s, O-H), 7.97 (1H, d, *J*

= 8.5 Hz, $H^8$), 7.87 (1H, d, *J* = 8.5 Hz, $H^5$), 7.70 (1H, t, *J* = 7.6 Hz, $H^7$), 7.49 (1H, t, *J* = 7.6 Hz, $H^6$), 7.36 (2H, d, *J* = 8.5 Hz, $H^{11}$), 7.29 (1H, s, $H^3$), 6.95 (2H, d, *J* = 8.5 Hz, $H^{12}$), 2.66 (3H, s, $H^9$). $^{13}$C NMR (126 MHz, DMSO-$d_6$): δ 158.8 ($C^2$), 158.4 ($C^{13}$), 148.5 ($C^{8'}$), 148.1 ($C^4$), 131.3 ($C^{11}$), 129.7 ($C^7$), 129.3 ($C^8$), 128.5 ($C^{10}$), 126.2 ($C^6$), 125.9 ($C^5$), 125.1 ($C^{4'}$), 122.4 ($C^3$), 116.1 ($C^{12}$), 25.3 ($C^9$). ESI-MS (*m/z)*: Found [M + H]$^+$ 236.1070, calc [$C_{16}H_{13}NO$ + H]$^+$ 236.1070.

4-(4-(Phenoxy)*tert*-butyl acetate)-2-methylquinoline or 4-(4-(1,1-dimethylethyl)-phenoxyacetate)-2-methylquinoline (**1c**)

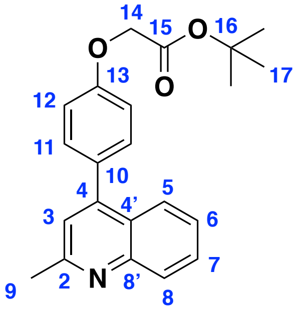


Under a nitrogen atmosphere, 4-(2-methyl-4-quinolinyl)-phenol (0.26 g, 1.11 mmol) and potassium carbonate (0.23 g, 1.66 mmol) were dissolved in anhydrous dimethylformamide (6 mL). Subsequently, *tert*-butyl chloroacetate (0.20 g, 1.33 mmol) was added and reaction stirred at room temperature for 6 hours. Water (50 mL) was added and extracted with diethyl ether (3 x 50 mL). The organic layers were combined, dried ($MgSO_4$) and solvent evaporated under reduced pressure. The residue was dissolved in LiCl (5 % solution, 50 mL) then extracted with diethyl ether (3 x 30 mL). The organic layers were combined, dried ($MgSO_4$) and solvent evaporated under reduced pressure to obtain the product as a yellow solid (0.34 g, 88%). $^1$H NMR (500 MHz, $CDCl_3$): δ 8.07 (1H, d, *J* = 8.2 Hz, $H^8$), 7.88 (1H, d, *J* = 8.2 Hz, $H^5$), 7.68 (1H, m, $H^7$), 7.45–7.41 (3H, m, $H^{11}$, $H^6$), 7.21 (1H, s, $H^3$), 7.04 (2H, dd, *J* = 7.0 Hz, 2.1 Hz, $H^{12}$), 4.61 (2H, s, $H^{14}$), 2.76 (3H, s, $H^9$), 1.52 (9H, s, $H^{17}$). $^{13}$C NMR (126 MHz, $CDCl_3$): δ 168.0 ($C^{15}$), 158.6 ($C^2$), 158.2 ($C^{13}$), 148.5 ($C^{8'}$), 148.1 ($C^4$), 131.4 ($C^{10}$), 130.8 ($C^{11}$), 129.3 ($C^7$), 129.1 ($C^8$), 125.8 ($C^6$), 125.7 ($C^5$), 125.3 ($C^{4'}$), 122.3 ($C^3$), 114.8 ($C^{12}$), 82.7 ($C^{16}$), 65.8 ($C^{14}$), 28.2 ($C^{17}$), 25.4 ($C^9$). ESI-MS (*m/z*): Found [M + H]$^+$ 350.1750, calc [$C_{22}H_{23}NO_3$ + H]$^+$ 350.1751; Found [M + Na]$^+$ 372.1570, calc [$C_{22}H_{23}NO_3$ + Na]$^+$ 372.1570; Found [$M_2$ + Na]$^+$ 721.3247, calc [($C_{22}H_{23}NO_3$)$_2$ + Na]$^+$ 721.3248.

4-(4-(1,1-Dimethylethyl)-phenoxyacetate)-2-quinolinecarboxaldehyde (**2b**)

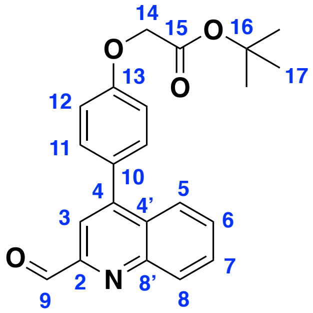


The 4-(4-(1,1-dimethylethyl)-phenoxyacetate)-2-methylquinoline (0.33 g, 0.93 mmol) and anhydrous dioxane (15 mL) were added to an oven-dried glassware under a nitrogen atmosphere. Selenium dioxide (0.21 g, 1.86 mmol) was added as one solid portion and the reaction was heated to 80 °C for 16 hours. The reaction was

cooled to RT then brine (50 mL) and ethyl acetate (50 mL) were added. The biphasic mixture was passed through a celite plug then the organic layer was separated. The aqueous layer was extracted with ethyl acetate (2 x 50 mL), organic layers combined, dried ($MgSO_4$) and the solvent removed under reduced pressure to obtain pure product (0.33 g, 96%). $^1$H NMR (500 MHz, $CDCl_3$): δ 10.26 (1H, s, $H^9$), 8.31 (1H, d, *J* = 8.7 Hz, $H^8$), 8.04 (1H, d, *J* = 8.2 Hz, $H^5$), 7.95 (1H, s, $H^3$), 7.82 (1H, t, *J* = 7.6 Hz, $H^7$), 7.65 (1H, t, *J* = 7.2 Hz, $H^6$), 7.48 (2H, d, *J* = 9.1 Hz, $H^{11}$), 7.07 (2H, d, *J* = 8.7 Hz, $H^{12}$), 4.62 (2H, s, $H^{14}$), 1.53 (9H, s, $H^{17}$). $^{13}$C NMR (126 MHz, $CDCl_3$): δ 194.1 ($C^9$), 167.9 ($C^{15}$), 158.6 ($C^{13}$), 152.2 ($C^2$), 149.6 ($C^4$), 148.7 ($C^{8'}$), 131.0 ($C^{11}$), 130.9 ($C^8$), 130.6 ($C^{10}$), 130.3 ($C^7$), 129.2 ($C^6$), 128.8 ($C^{4'}$), 126.2 ($C^5$), 117.5 ($C^3$), 115.0 ($C^{12}$), 82.7 ($C^{16}$), 65.8 ($C^{14}$), 28.2 ($C^{17}$). ESI-MS (*m/z)*: Found $[M + H]^+$ 364.1545, calc $[C_{22}H_{21}NO_4 + H]^+$ 364.1543.

4-(4-(1,1-Dimethylethyl)-phenoxyacetate)-2-quinolinemethanol (**3b**)

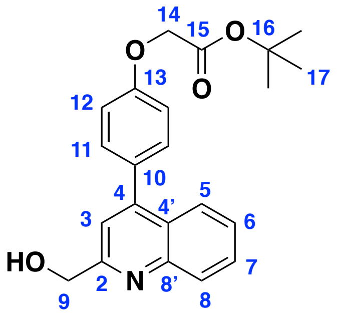


4-(4-(1,1-Dimethylethyl)-phenoxyacetate)-2-quinolinecarboxaldehyde (0.31, 0.85 mmol) was dissolved in anhydrous methanol (15 mL) under a nitrogen atmosphere. The flask was cooled to 0 °C, sodium borohydride (0.04 g, 1.06 mmol) was carefully added as one solid, and the reaction was allowed to warm to room temperature and stirred for 2 hours. The reaction was quenched with saturated $NH_4Cl$ solution (20 mL) then the methanol was evaporated under reduced pressure. The remaining aqueous solution was extracted with chloroform (3 x 50 mL), organics combined, washed with brine (2 x 50 mL), dried ($MgSO_4$) and the solvent evaporated under reduced pressure to yield the product as a yellow solid (0.30 g, 97%). $^1$H NMR (500 MHz, $CDCl_3$): δ 8.13 (1H, d, *J* = 7.8 Hz, $H^8$), 7.92 (1H, d, *J* = 7.8 Hz, $H^5$), 7.72 (1H, t, *J* = 7.2 Hz, $H^7$), 7.49 (1H, t, *J* = 7.4 Hz, $H^6$), 7.44 (2H, d, *J* = 8.7 Hz, $H^{11}$), 7.20 (1H, s, $H^3$), 7.05 (2H, d, *J* = 8.7 Hz, $H^{12}$), 4.94 (2H, s, $H^9$), 4.61 (2H, s, $H^{14}$), 1.52 (9H, s, $H^{17}$). $^{13}$C NMR (126 MHz, $CDCl_3$): δ 167.9 ($C^{15}$), 158.5 ($C^{13}$), 158.4 ($C^2$), 149.0 ($C^4$), 147.4 ($C^{8'}$), 131.1 ($C^{10}$), 130.9 ($C^{11}$), 129.7 ($C^8$), 129.1 ($C^7$), 126.4 ($C^{4'}$), 126.4 ($C^6$), 126.0 ($C^5$), 118.6 ($C^3$), 114.8 ($C^{12}$), 82.7 ($C^{16}$), 65.8 ($C^{14}$), 64.2 ($C^9$), 28.2 ($C^{17}$). ESI-MS (*m/z)*: Found $[M + H]^+$ 366.1700, calc $[C_{22}H_{23}NO_4 + H]^+$ 366.1700.

4-(4-(1,1-Dimethylethyl)-phenoxyacetate)- 2-methanesulfonate-2-quinolinemethanol (**4b**)

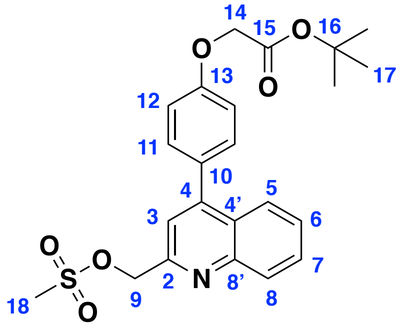

In an oven-dried Schlenk, 4-(4-(1,1-dimethylethyl)-phenoxyacetate)-2-quinolinemethanol (0.30 g, 0.82 mmol) was dissolved in anhydrous dichloromethane (8 mL) under a nitrogen atmosphere. Diisopropylethylamine (0.29 mL, 1.64 mmol) and methanesulfonyl chloride (0.06 mL, 0.81 mmol) were added and the mixture was stirred at room temperature for 2 hours. Water (20 mL) was added, and the organic layer separated. The aqueous layer was extracted with dichloromethane (2 x 20 mL) and the combined organic layers were washed with brine (2 x 50 mL), the organic layer dried ($MgSO_4$) and concentrated under reduced pressure, to give the product as a yellow oil (0.35 g, 96%). $^1$H NMR (500 MHz, $CDCl_3$): δ 8.10 (1H, d, $J$ = 8.7 Hz, $H^8$), 7.95 (1H, d, $J$ = 8.7 Hz, $H^5$), 7.73 (1H, t, $J$ = 7.0 Hz, $H^7$), 7.52 (1H, t, $J$ = 6.8 Hz, $H^6$), 7.47 (1H, s, $H^3$), 7.44 (2H, d, $J$ = 8.7 Hz, $H^{11}$), 7.04 (2H, d, $J$ = 8.7 Hz, $H^{12}$), 5.50 (2H, s, $H^9$), 4.60 (2H, s, $H^{14}$), 3.12 (3H, s, $H^{18}$), 1.50 (9H, s, $H^{17}$). $^{13}$C NMR (126 MHz, $CDCl_3$): δ 167.9 ($C^{15}$), 158.5 ($C^{13}$), 153.4 ($C^2$), 149.6 ($C^4$), 148.3 ($C^{8'}$), 130.9 ($C^{11}$), 130.8 ($C^{10}$), 130.0 ($C^7$), 129.7 ($C^8$), 127.2 ($C^6$), 126.5 ($C^{4'}$), 126.0 ($C^5$), 119.8 ($C^3$), 114.9 ($C^{12}$), 82.7 ($C^{16}$), 72.2 ($C^9$), 65.8 ($C^{14}$), 38.2 ($C^{18}$), 28.2 ($C^{17}$). LR-MS ESI (*m/z*): Found $[M + H]^+$ 444.2, calc $[C_{23}H_{25}NO_6S + H]^+$ 444.1.

4,10-*Bis*((4-(4-(1,1-dimethylethyl)-phenoxyacetate)-quinolin-2-yl)-methyl)-1,4,7,10-tetraazacyclododecane-1,7-diyl)-diacetate (**5b**)

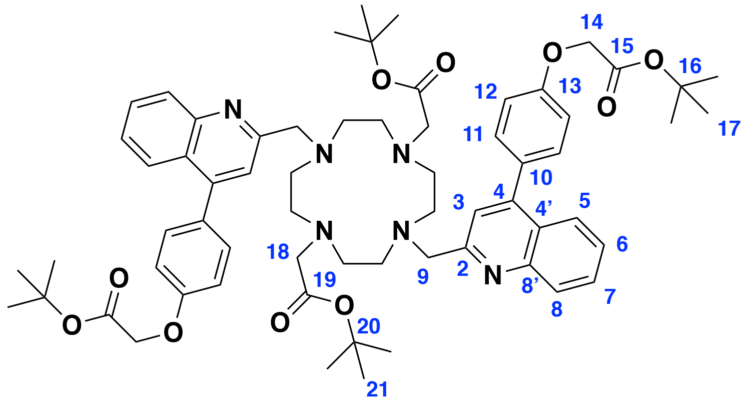


To a solution of DO2A-*tert*-butyl ester (81 mg, 0.23 mmol) and potassium carbonate (93 mg, 0.68 mmol) in anhydrous acetonitrile (10 mL), was added 4-(4-(1,1-dimethylethyl)-phenoxyacetate)- 2-methanesulfonate-2-quinolinemethanol (300 mg, 0.68 mmol). The reaction mixture was stirred at 60°C for 64 hours. The reaction was cooled to room temperature, salts removed through centrifugation (1500 rpm for 5 minutes). The organic layer was removed, and the salts washed with acetonitrile (2 x 10 mL). The organic layers combined, and the solvent removed under reduced pressure. The crude material was purified by column chromatography (silica gel; neat dichloromethane to 9:1 dichloromethane/methanol) to give the desired protected ligand, as a yellow solid (134 mg, 60%). $^1$H NMR (400 MHz, $CDCl_3$): δ 7.96 (2H, d, $J$ = 8.4 Hz, $H^8$), 7.92 (2H, d, $J$ = 7.4 Hz, $H^5$), 7.46 (4H, d, $J$ = 8.7 Hz, $H^{11}$), 7.37 (2H, t, $J$ = 7.6 Hz, $H^6$), 7.25–7.21 (4H, m, $H^3$, $H^7$), 7.06 (4H, d, $J$ = 9.1 Hz, $H^{12}$), 4.61 (4H, s, $H^{14}$), 3.05–1.90 (24H, m, $H^9$, $H^{18}$, $H^{cyclen}$), 1.51 (18H, s, $H^{17}$), 1.19 (18H, s, $H^{21}$). $^{13}$C NMR (101 MHz, $CDCl_3$): δ 172.0 ($C^{19}$), 167.9 ($C^{15}$), 158.7 ($C^2$), 158.5 ($C^{13}$), 149.1 ($C^4$), 148.5 ($C^{8'}$), 130.9 ($C^{11}$), 130.7 ($C^{10}$), 130.0 ($C^8$), 129.5 ($C^7$), 126.3 ($C^6$), 126.0 ($C^{4'}$), 125.8 ($C^5$), 121.7 ($C^3$), 114.9 ($C^{12}$), 82.8 ($C^{16}$), 82.1 ($C^{20}$), 65.8 ($C^{14}$), 60.2 ($C^9$), 58.0 ($C^{18}$), 51.0 ($C^{cyclen}$), 50.6 ($C^{cyclen}$), 28.2 ($C^{17}$), 28.1 ($C^{21}$). ESI-MS (*m/z)*: Found $[M + 2H]^{2+}$ 548.3120, calc $[C_{64}H_{82}N_6O_{10} + 2H]^{2+}$ 548.3119; Found $[M + H]^+$ 1095.6169, calc $[C_{64}H_{82}N_6O_{10} + H]^+$ 1095.6165; Found $[M + Na]^+$ 1117.5989, calc $[C_{64}H_{82}N_6O_{10} + Na]^+$ 1117.5985.

4,10-*Bis*((4-(4-(1,1-dimethylethyl)-phenoxyacetate)-quinolin-2-yl)-methyl)-1,4,7,10-tetraazacyclododecane-1,7-diyl)-diacetic acid (**6b**)

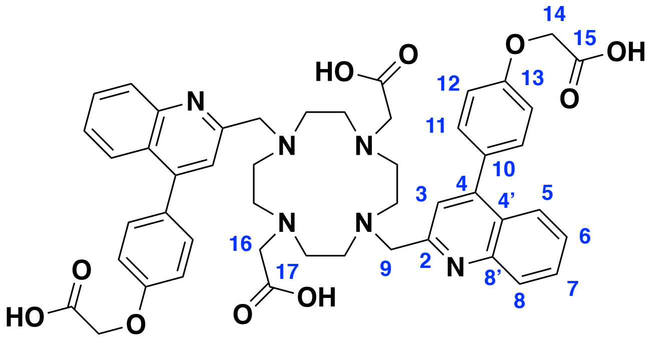

To a solution of 4,10-*bis*((4-(4-(1,1-dimethylethyl)-phenoxyacetate)-quinolin-2-yl)-methyl)-1,4,7,10-tetraazacyclododecane-1,7-diyl)-diacetate (40 mg, 37 μmol) in dichloromethane (1 mL) was added trifluoroacetic acid (1 mL). The reaction mixture was stirred at room temperature for 16 hours and the trifluoroacetic acid was co-evaporated with dichloromethane (10 x 20 mL) to give the deprotected ligand as a pale-yellow solid (40 mg, 100%). $^1$H NMR (500 MHz, $CD_3OD$): δ 8.21 (2H, d, $J$ = 8.7 Hz, $H^8$), 8.01 (2H, d, $J$ = 7.8 Hz, $H^5$), 7.56 – 7.52 (6H, m, $H^{11}$, $H^6$), 7.48 (2H, s, $H^3$), 7.34 (2H, t, $J$ = 7.2 Hz, $H^7$), 7.17 (4H, d, $J$ = 8.7 Hz, $H^{12}$), 5.06 (4H, s, $H^9$), 4.80 (4H, s, $H^{14}$), 3.90 – 3.75 (12H, m, $H^{cyclen}$, $H^{16}$), 3.42 – 3.30 (8H, m, $H^{cyclen}$). $^{13}$C NMR (125 MHz, $CDCl_3$): δ 172.6 ($C^{17}$), 171.1 ($C^{15}$), 158.9 ($C^{13}$), 150.8 ($C^2$), 150.4 ($C^4$), 147.5 ($C^{8'}$), 130.7 ($C^{11}$), 130.2 ($C^{10}$), 130.1 ($C^7$), 129.2 ($C^8$), 127.1 ($C^6$), 126.4 ($C^{4'}$), 125.7 ($C^5$), 119.8 ($C^3$), 114.8 ($C^{12}$), 64.6 ($C^{14}$), 58.4 ($C^9$), 52.8 ($C^{16}$), 51.9 ($C^{cyclen}$), 48.2 ($C^{cyclen}$). ESI-MS (*m/z)*: Found $[M + H]^+$ 871.3668, calc $[C_{48}H_{50}N_6O_{10} + H]^+$ 871.3661; Found $[M + 2H]^{2+}$ 436.1867, calc $[C_{48}H_{50}N_6O_{10} + 2H]^{2+}$ 436.1867;

**$[Eu.4PhOCH_2COO]^-$**

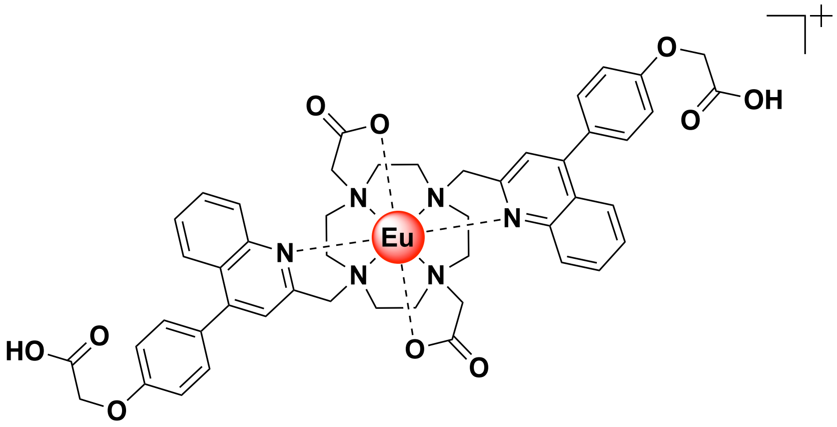

The deprotected ligand (35 mg, 0.040 mmol) was dissolved in methanol (2 mL). Potassium carbonate (2 mg, 0.015 mmol) and europium(III) trifluoromethanesulfonate (48 mg, 0.08 mmol) were added and the reaction heated to 50°C for 72 hours. The reaction was cooled to room temperature, filtered and the solvent evaporated under reduced pressure. The resulting yellow solid was purified by preparative RP-HPLC [gradient: 0 − 100% acetonitrile in 100 mM $NH_4HCO_3$ over 20 minutes, at 17 mL per minute; $t_R$ = 7.08 min] to give the complex $[Eu.suzPhOCH_2COO]^-$ as a pale-yellow solid (12 mg, 29%). $^1$H-NMR (500 MHz, $CD_3OD$): δ 51.8, 47.4, 45.7, 40.2, 30.8, 27.1, 18.3, 17.5, 17.0, 13.4, 13.0, 12.0, 11.7, 11.6, 11.4, 11.2, 10.9, 9.8, 9.3, 9.1, 9.0, 8.8, 8.7, 8.4, 8.0, 8.0, 7.7, 7.6, 7.5, 7.5, 7.4, 7.1, 7.1, 7.1, 6.0, 5.7, 5.5, 5.3, 5.1, 4.9, 4.5, 3.9, 3.6, 3.5, 3.4, 3.1, 3.0, 3.0, 2.8, 2.6, 2.2, 2.2, 2.2, 2.0, 2.0, 1.6, 1.3, 1.3, 1.2, 1.1, 1.1, 1.1, 0.9, 0.1, -1.1, -2.8, -5.6, -9.4, -10.8, -11.3, -13.5, -16.6, -16.7, -17.8, -28.3, -29.5, -30.4, -34.0. ESI-MS (*m/z*): Found $[M]^+$ 1021.2642, calc $[C_{48}H_{48}EuN_6O_{10}]^+$

1021.2639.Photophysical data measured in methanol: $\lambda_{max}$ = 321 nm, $\varepsilon$ = 26700 $M^{-1}$ $cm^{-1}$, $\Phi_{em}$ = 2.4%, $\tau_{H2O}$ = 0.466 ms, $\tau_{D2O}$ = 1.004 ms, $q$ = 0.9.

7-(4-Methoxyphenyl)-2-methylquinoline (**1d**)

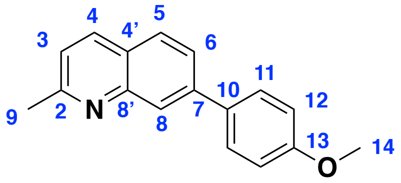


7-Bromoquinaldine (1.01 g, 4.55 mmol), 4-methoxyphenylboronic acid (1.04 g, 6.84 mmol) and potassium carbonate (1.90 g, 13.75 mmol) were added to an oven-dried Schlenk with a condenser attached. Anhydrous dioxane (30 mL) and oxygen-free water (9 mL) were added, followed by palladium-tetrakis(triphenylphosphine) (0.52 g, 0.45 mmol) and the reaction was heated to 60 °C for 16 hours. The reaction as cooled to room temperature, filtered through celite and the filtrate was removed under reduced pressure. The product was obtained after column chromatography (silica gel; 1:9 ethyl acetate/hexane) to give the product as a pale-yellow solid (0.92 g, 81%). $^1$H NMR (400 MHz, $CDCl_3$): δ 8.20 (1H, s, $H^8$), 8.03 (1h, d, $J$ = 8.7 Hz, $H^4$), 7.79 (1H, d, $J$ = 8.2 Hz, $H^5$), 7.73–7.68 (3H, m, $H^6$, $H^{11}$), 7.26–7.24 (1H, m, $H^3$), 7.02 (2H, d, $J$ = 9.1 Hz, $H^{12}$), 3.86 (3H, s, $H^{14}$), 2.73 (3H, s, $H^9$). $^{13}$C NMR (101 MHz, $CDCl_3$): δ 159.7 ($C^{13}$), 159.5 ($C^2$), 148.4 ($C^{8'}$), 141.8 ($C^7$), 135.9 ($C^4$), 133.0 ($C^{10}$), 128.5 ($C^{11}$), 127.9 ($C^5$), 125.6 ($C^8$), 125.3 ($C^{4'}$), 125.2 ($C^6$), 121.7 ($C^3$), 114.5 ($C^{12}$), 55.5 ($C^{14}$), 25.5 ($C^9$). ESI-MS (*m/z)*: Found $[M + H]^+$ 250.1226, calc $[C_{17}H_{15}NO + H]^+$ 250.1226.

7-(4-Methoxyphenyl)-2-quinolinecarboxaldehyde (**2c**)

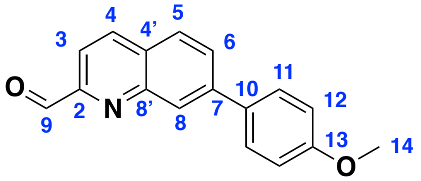


7-(4-Methoxyphenyl)-2-methylquinoline (0.76 g, 3.03 mmol) and anhydrous dioxane (30 mL) were added to an oven-dried glassware under a nitrogen atmosphere. Selenium dioxide (0.67 g, 6.06 mmol) was added as one solid portion and the reaction was heated to 80°C for 18 hours. The reaction was cooled to room temperature then brine (50 mL) and ethyl acetate (50 mL) were added. The biphasic mixture was passed through celite then the organic layer was separated. The aqueous layer was extracted with ethyl acetate (3 x 50 mL), organic layers combined, dried ($MgSO_4$) and the solvent removed under reduced pressure. The crude material was purified by column chromatography (silica gel; dichloromethane) to obtain the product as a pale-yellow solid (0.64 g, 80%). $^1$H NMR (400 MHz, $CDCl_3$): δ 10.24 (1H, d, $J$ = 0.8 Hz, $H^9$), 8.41 (1H, d, $J$ = 0.8 Hz, $H^8$), 8.31 (1H, d, $J$ = 8.0 Hz, $H^4$), 8.00 (1H, d, $J$ = 8.2 Hz, $H^3$), 7.94–7.93 (1H, m, $H^5$, $H^6$), 7.73 (2H, d, $J$ = 8.7 Hz, $H^{11}$), 7.06 (2H, d, $J$ = 8.7 Hz, $H^{12}$), 3.89 (3H, s, $H^{14}$). $^{13}$C NMR (100 MHz, $CDCl_3$): δ 193.8 ($C^9$), 160.1 ($C^{13}$), 153.1 ($C^2$), 148.5 ($C^{8'}$), 143.0 ($C^7$), 137.1 ($C^4$), 132.1 ($C^{10}$), 128.9 ($C^{4'}$), 128.8 ($C^6$), 128.7 ($C^{11}$), 128.2 ($C^5$), 127.0 ($C^8$), 117.1 ($C^3$), 114.7 ($C^{12}$), 55.5 ($C^{14}$). ESI-MS (*m/z)*: Found $[M + H]^+$ 264.1019, calc $[C_{17}H_{13}NO_2 + H]^+$ 264.1019.

7-(4-Methoxyphenyl)-2-quinolinemethanol (**3c**)

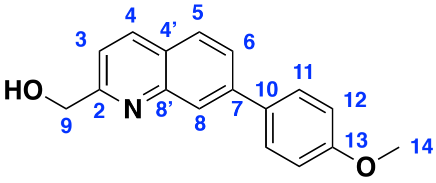


7-(4-Methoxyphenyl)-2-quinolinecarboxaldehyde (0.60 g, 2.28 mmol) was dissolved in anhydrous methanol (30 mL) under a nitrogen atmosphere and cooled to 0 °C. Sodium borohydride (0.10 g, 2.73 mmol) was carefully added as one solid portion, then the reaction was allowed to warm to room temperature and stirred for 2 hours. The reaction was quenched with $NH_4Cl$ solution (30 mL) then methanol evaporated under reduced pressure. The resulting aqueous solution was extracted with chloroform (3 x 50 mL), organics combined, washed with brine (100 mL), dried ($MgSO_4$) and the solvent evaporated under reduced pressure to yield the product was a yellow solid (0.59 g, 98%). $^1$H NMR (400 MHz, $CDCl_3$): δ 8.24 (1H, s, $H^8$), 8.13 (1H, d, $J$ = 8.7 Hz, $H^4$), 7.86 (1H, d, $J$ = 8.2 Hz, $H^5$), 7.78 (1H, dd, $J$ = 8.7 Hz, 1.6 Hz, $H^6$), 7.71 (2H, d, $J$ = 9.1 Hz, $H^{11}$), 7.27–7.25 (1H, m, $H^3$), 7.04 (2H, d, $J$ = 8.7 Hz, $H^{12}$), 4.93 (2H, s, $H^9$), 3.88 (3H, s, H1$^4$). $^{13}$C NMR (101 MHz, $CDCl_3$): δ 159.8 ($C^{13}$), 159.4 ($C^2$), 147.3 ($C^{8'}$), 142.3 ($C^7$), 136.3 ($C^4$), 132.7 ($C^{10}$), 128.6 ($C^{11}$), 128.1 ($C^5$), 126.4 ($C^{4'}$), 125.8 ($C^6$), 125.6 ($C^8$), 118.0 ($C^3$), 114.6 ($C^{12}$), 64.3 ($C^9$), 55.5 ($C^{14}$). ESI-MS (*m/z)*: Found $[M + H]^+$ 266.1175, calc $[C_{17}H_{15}NO_2 + H]^+$ 266.1176; Found $[M + Na]^+$ 288.0994, calc $[C_{17}H_{15}NO_2 + Na]^+$ 288.0995.

7-(4-Methoxyphenyl)- 2-methanesulfonate-2-quinolinemethanol (**4c**)

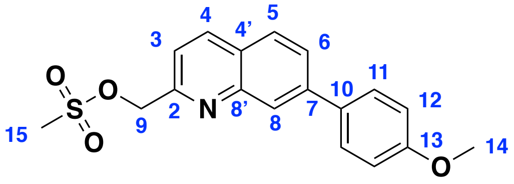


7-(4-Methoxyphenyl)-2-quinolinemethanol (0.50 g, 1.89 mmol) was dissolved in anhydrous dichloromethane (10 mL) then diisopropylethylamine (0.49 mL, 2.83 mmmol) and methanesulfonyl chloride (0.14 mL, 1.85 mmol) was added. The mixture was stirred under a nitrogen atmosphere for 1 hour. Water (20 mL) was added, and the organic layer separated. The aqueous solution was extracted with dichloromethane (3 x 20 mL), organic layers combined, washed with brine (100 mL), dried ($MgSO_4$) and concentrated under reduced pressure to give the product as an orange solid, which was used immediately in the next step (0.63 g, quant.). $^1$H NMR (400 MHz, $CDCl_3$): δ 8.24–8.22 (2H, m, $H^8$, $H^4$), 7.89 (1H, d, $J$ = 8.7 Hz, $H^5$), 7.83 (1H, dd, $J$ = 8.7 Hz, 1.6 Hz, $H^6$), 7.70 (2H, d, $J$ = 8.7 Hz, $H^{11}$), 7.56 (1H, d, $J$ = 8.7 Hz, $H^3$), 7.04 (2H, d, $J$ = 9.1 Hz, $H^{12}$), 5.51 (2H, s, $H^9$), 3.88 (3H, s, $H^{14}$), 3.13 (3H, s, $H^{15}$). $^{13}$C NMR (101 MHz, $CDCl_3$): δ 159.9 ($C^{13}$), 154.3 ($C^2$), 148.1 ($C^{8'}$), 142.6 ($C^7$), 137.2 ($C^4$), 132.5 ($C^{10}$), 128.6 ($C^{11}$), 128.1 ($C^5$), 126.8 ($C^{4'}$), 126.6 ($C^6$), 126.0 ($C^8$), 119.3 ($C^3$), 114.6 ($C^{12}$), 72.3 ($C^9$), 55.5 ($C^{14}$), 38.2 ($C^{15}$). ESI-MS (*m/z*): Found $[M + H]^+$ 344.0951, calc $[C_{18}H_{17}NO_4S + H]^+$ 344.0951; Found $[2M + Na]^+$ 709.1648, calc $[(C_{18}H_{17}NO_4S)_2 + Na]^+$ 709.1649.

4,10-*Bis*((7-(4-methoxyphenyl)-quinolin-2-yl)-methyl)-1,4,7,10-tetraazacyclododecane-1,7-diyl)-diacetate (**5c**)

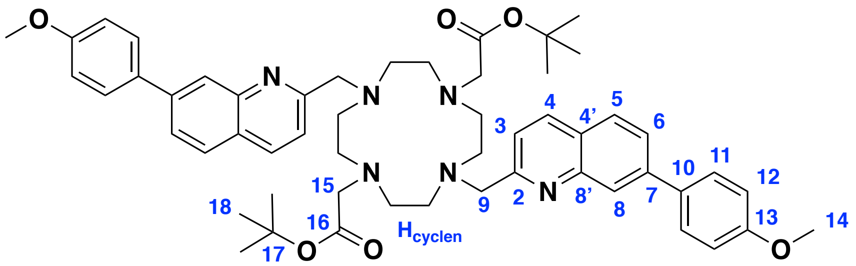


To a solution of DO2A-*tert*-butyl ester (0.25 g, 0.62 mmol) and potassium carbonate (0.26 mg, 1.85 mmol) in anhydrous acetonitrile (15 mL), was added 7-(4-methoxyphenyl)- 2-methanesulfonate-2-quinolinemethanol (0.63 mg, 1.85 mmol). The reaction mixture was stirred at 60 °C for 18 hours. The reaction was cooled to room temperature, salts removed through centrifugation (1500 rpm for 5 minutes). The organic layer was removed, and the salts washed with acetonitrile (2 x 20 mL). The organic layers combined, and the solvent removed under reduced pressure. The crude material was purified by column chromatography (silica gel; neat dichloromethane to 9:1 dichloromethane/methanol) to give the desired protected ligand, as a yellow solid (0.35 g, 62%). $^{1}$H-NMR (400 MHz, $CDCl_3$): δ 8.26 (2H, d, *J* = 8.2 Hz, $H^{4}$), 8.09 (2H, s, $H^{8}$), 7.83 (2H, d, *J* = 8.7 Hz, $H^{5}$), 7.55 (2H, dd, *J* = 8.4 Hz, 1.9 Hz, $H^{6}$), 7.40 (2H, d, *J* = 8.2 Hz, $H^{3}$), 6.71 (4H, d, *J* = 9.1 Hz, $H^{11}$), 6.47 (4H, d, *J* = 8.7 Hz, $H^{12}$), 3.73 (6H, s, $H^{14}$), 2.92–2.14 (24H, m, $H^{15}$, $H^{9}$, $H^{cyclen}$), 0.89 (16H, s, $H^{18}$). $^{13}$C NMR (101 MHz, $CDCl_3$): δ 171.4 ($C^{16}$), 160.0 ($C^{2}$), 159.3 ($C^{13}$), 148.3 ($C^{8'}$), 142.1 ($C^{7}$), 137.2 ($C^{4}$), 132.1 ($C^{10}$), 128.1 ($C^{11}$), 128.0 ($C^{5}$), 126.4 ($C^{4'}$), 126.1 ($C^{6}$), 126.0 ($C^{8}$), 121.4 ($C^{3}$), 113.9 ($C^{12}$), 82.0 ($C^{17}$), 60.1 ($C^{9}$), 58.3 ($C^{15}$), 55.4 ($C^{14}$), 51.1 ($C^{cyclen}$), 57.6 ($C^{18}$). ESI-MS (*m/z)*: Found $[M + H]^{+}$ 895.5113, calc $[C_{54}H_{66}N_6O_6 + H]^{+}$ 895.5117; Found $[M + Na]^{+}$ 917.4934, calc $[C_{54}H_{66}N_6O_6 + Na]^{+}$ 917.4934; Found $[M + 2H]^{2+}$ 448.2594, calc $[C_{54}H_{66}N_6O_6 + 2H]^{2+}$ 448.2595.

4,10-*Bis*((7-(4-methoxyphenyl)-quinolin-2-yl)-methyl)-1,4,7,10-tetraazacyclododecane-1,7-diyl)-diacetic acid (**6c**)

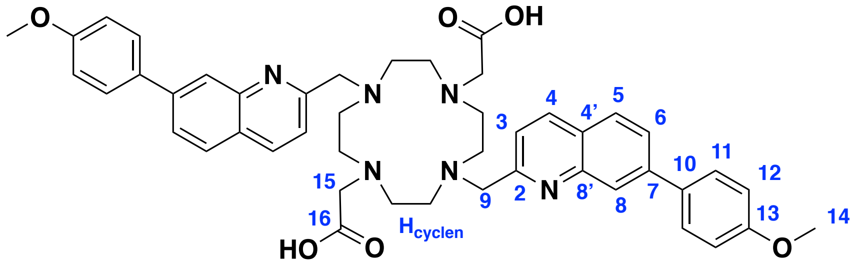


To a solution of 4,10-*bis*((7-(4-methoxyphenyl)-quinolin-2-yl)-methyl)-1,4,7,10-tetraazacyclododecane-1,7-diyl)-diacetate (116 mg, 0.13 mmol) in dichloromethane (2 mL) was added trifluoroacetic acid (2 mL). The reaction mixture was stirred at room temperature for 16 hours, then the trifluoroacetic acid was co-evaporated with dichloromethane (5 x 25 mL) to give the deprotected ligand as a yellow solid (0.13 g, 99%). $^{1}$H NMR (500 MHz, $CD_3OD$): δ 8.38 (2H, d, *J* = 8.7 Hz, $H^{4}$), 8.08 (2H, s, $H^{8}$), 7.80 (2H, d, *J* = 8.2 Hz, $H^{5}$), 7.59 (2H, d, *J* = 8.7 Hz, $H^{6}$), 7.52 (2H, d, *J* = 8.2 Hz, $H^{3}$), 7.11 (4H, d, *J* = 8.7 Hz, $H^{11}$), 6.51 (4H, d, *J* = 8.7 Hz, $H^{12}$), 5.12 (4H, s, $H^{9}$), 3.88–3.75 (18H, m, $H^{15}$, $H^{cyclen}$, $H^{14}$), 3.45–3.35 (8H, m, $H^{cyclen}$). $^{13}$C NMR (126 MHz, $CD_3OD$): δ 172.0 ($C^{16}$), 159.5 ($C^{13}$), 151.1 ($C^{2}$), 147.1 ($C^{8'}$), 142.7 ($C^{7}$), 138.1 ($C^{4}$), 131.4 ($C^{10}$), 127.9 ($C^{11}$, $C^{5}$), 126.8 ($C^{4'}$), 126.3 ($C^{6}$), 124.7 ($C^{8}$), 119.2 ($C^{3}$), 113.4 ($C^{12}$), 58.1 ($C^{9}$), 54.3 ($C^{14}$), 53.3 ($C^{15}$), 51.6 ($C^{cyclen}$), 48.5 ($C^{cyclen}$). ESI-MS (*m/z)*: Found $[M + H]^{+}$ 783.3861, calc $[C_{46}H_{50}N_6O_6 + H]^{+}$ 783.3865; Found $[M + Na]^{+}$ 805.3681, calc $[C_{46}H_{50}N_6O_6 + Na]^{+}$ 805.3684; Found $[M + 2H]^{2+}$ 392.1967, calc $[C_{46}H_{50}N_6O_6 + 2H]^{2+}$ 392.1969.

[Eu.7PhOMe]$^{+}$

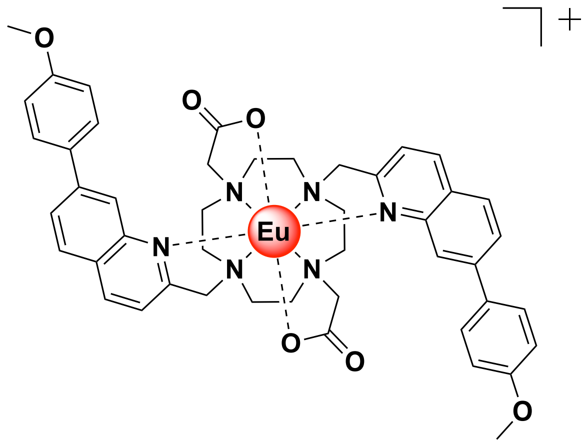


The deprotected ligand (130 mg, 0.13 mmol) was dissolved in methanol (5 mL). Potassium carbonate (53 mg, 0.38 mmol) and europium(III) trifluoromethanesulfonate (230 mg, 0.38 mmol) were added and the reaction heated to 50 °C for 18 hours. The reaction was cooled to room temperature, filtered and the solvent evaporated under reduced pressure. The crude material was purified by column chromatography (silica gel; neat dichloromethane to 4:1 dichloromethane/methanol) to give the desired complex as a pale-yellow solid (124 mg, 89%). $^{1}$H-NMR (500 MHz, $CD_3OD$): δ 47.8, 30.7, 18.2, 16.7, 15.9, 15.6, 14.8, 12.2, 11.4, 11.3, 10.4, 9.7, 7.9, 6.7, 5.8, 4.9, 4.9, 4.2, 3.6, 3.5, 3.4, 3.4, 3.3, 3.2, 3.2, 3.1, 2.8, 2.6, 2.5, 2.0, 1.9, 1.6, 1.5, 1.3, 1.3, 0.9, 0.8, 0.1, -0.3, -1.1, -2.9, -4.1, -6.0, -9.1, -10.7, -17.4, -17.7, -18.6, -27.0, -29.7, -30.3, -34.3. ESI-MS (*m/z*): Found $[M]^+$ 933.2843, calc $[C_{46}H_{48}N_6O_6Eu]^+$ 933.2842. Photophysical data measured in methanol: $\lambda_{max}$ = 348 nm, $\varepsilon$ = 6400 $M^{-1}$ $cm^{-1}$, $\Phi_{em}$ = 0.4%, $\tau_{CH3OH}$ = 0.991 ms, $\tau_{CD3OD}$ = 1.306 ms, *m* = 0.4.

7-(2-Methyl-4-quinolinyl)-phenol (**1e**)

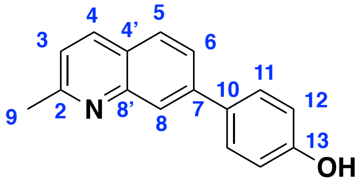


7-Bromoquinaldine (1.00 g, 4.55 mmol), 4-hydroxyphenyl boronic acid (1.19 g, 8.63 mmol) and potassium carbonate (1.30 g, 9.41 mmol) were added to an oven-dried Schlenk with a condenser attached. Anhydrous dioxane (30 mL) and oxygen-free water (9 mL) were added, followed by palladium-tetrakis(triphenylphosphine) (0.52 g, 0.45 mmol) and the reaction was heated to 80 °C for 64 hours. The reaction as cooled to room temperature and the solvent removed under reduced pressure. The residue was purified by column chromatography (silica gel; neat hexanes to 1:1 ethyl acetate/hexanes) to obtain the product as an off-white solid (0.98 g, 93%). $^{1}$H NMR (400 MHz, DMSO-$d_6$): δ 8.20 (1H, d, *J* = 8.2 Hz, $H^4$), 8.07 (1H, s, $H^8$), 7.88 (1H, d, *J* = 8.7 Hz, $H^5$), 7.78 (1H, dd, *J* = 8.2 Hz, 1.6 Hz, $H^6$), 7.62 (2H, d, *J* = 8.7 Hz, $H^{11}$), 7.36 (1H, d, *J* = 8.7 Hz, $H^3$), 6.92 (2H, d, *J* = 8.7 Hz, $H^{12}$), 2.71 (3H, s, $H^9$). $^{13}$C NMR (101 MHz, DMSO-$d_6$): δ 159.4 ($C^2$), 157.7 ($C^{13}$), 147.5 ($C^{8'}$), 142.7 ($C^7$), 136.8 ($C^4$), 131.2 ($C^{10}$), 128.2 ($C^{11}$), 127.9 ($C^5$), 125.4 ($C^{4'}$), 125.1 ($C^6$), 123.3 ($C^8$), 121.5 ($C^3$), 115.6 ($C^{12}$), 23.3 ($C^9$). ESI-MS (*m/z*): Found $[M + H]^+$ 236.1070, calc $[C_{16}H_{13}NO + H]^+$ 236.1070.

7-(4-(Phenoxy)*tert*-butyl acetate)-2-methylquinoline or 4-(4-(1,1-dimethylethyl)-phenoxyacetate)-2-methylquinoline (**1f**)

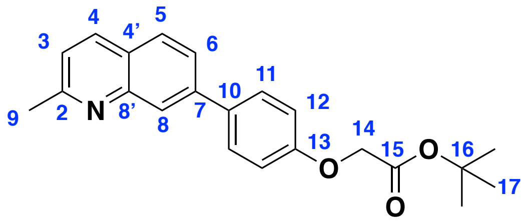


Under a nitrogen atmosphere, 7-(2-methyl-4-quinolinyl)-phenol (0.29 g, 1.22 mmol) and potassium carbonate (0.25 g, 1.83 mmol) were dissolved in anhydrous dimethylformamide (6 mL). Subsequently, *tert*-butyl chloroacetate (0.22 g, 1.46 mmol) was added and reaction stirred at room temperature for 16 hours. Water (50 mL) was added and extracted with diethyl ether (3 x 50 mL). The organic layers were combined, dried ($MgSO_4$) and solvent evaporated under reduced pressure and dried on the high vac to obtain the product as a yellow solid (0.41 g, 96%). $^1$H NMR (400 MHz, $CDCl_3$): δ 8.19 (1H, s, $H^8$), 8.01 (1H, d, *J* = 8.2 Hz, $H^4$), 7.78 (1H, d, *J* = 8.7 Hz, $H^5$), 7.71 – 7.67 (3H, m, $H^6$, $H^{11}$), 7.24 (1H, d, *J* = 8.2 Hz, $H^3$), 7.00 (2H, d, *J* = 8.7 Hz, $H^{12}$), 4.57 (2H, s, $H^{14}$), 2.74 (3H, s, $H^9$), 1.49 (9H, s, $H^{17}$). $^{13}$C NMR (101 MHz, $CDCl_3$): δ 168.0 ($C^{15}$), 159.5 ($C^2$), 158.0 ($C^{13}$), 148.3 ($C^{8'}$), 141.6 ($C^7$), 135.9 ($C^4$), 133.8 ($C^{10}$), 128.6 ($C^{11}$), 127.9 ($C^5$), 125.7 ($C^8$), 125.4 ($C^{4'}$), 125.1 ($C^6$), 121.8 ($C^3$), 115.2 ($C^{12}$), 82.5 ($C^{16}$), 65.9 ($C^{14}$), 28.1 ($C^{17}$), 25.5 ($C^9$). ESI-MS (*m/z)*: Found $[M + H]^+$ 350.1750, calc $[C_{22}H_{23}NO_3 + H]^+$ 350.1751.

7-(4-(1,1-Dimethylethyl)-phenoxyacetate)-2-quinolinecarboxaldehyde (**2d**)

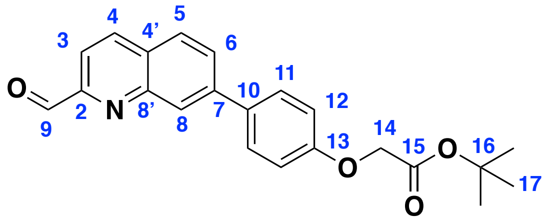


The 7-(4-(1,1-dimethylethyl)-phenoxyacetate)-2-methylquinoline (0.39 g, 1.12 mmol) and anhydrous dioxane (18 mL) were added to an oven-dried glassware under a nitrogen atmosphere. Selenium dioxide (0.27 g, 2.25 mmol) was added as one solid portion and the reaction was heated to 80 °C for 3 hours. The reaction was cooled to RT then brine (30 mL) and ethyl acetate (50 mL) were added. The biphasic mixture was passed through a celite plug then the organic layer was separated. The aqueous layer was extracted with ethyl acetate (2 x 50 mL), organic layers combined, dried ($MgSO_4$) and the solvent removed under reduced pressure to obtain pure product (0.41 g, 99%). $^1$H NMR (400 MHz, $CDCl_3$): δ 10.23 (1H, s, $H^9$), 8.40 (1H, s, $H^8$), 8.30 (1H, d, *J* = 8.7 Hz, $H^4$), 8.00 (1H, d, *J* = 8.7 Hz, $H^3$), 7.94–7.92 (2H, m, $H^5$, $H^6$), 7.72 (2H, d, *J* = 9.1 Hz, $H^{11}$), 7.05 (2H, d, *J* = 8.7 Hz, $H^{12}$), 4.60 (2H, s, $H^{14}$), 1.51 (9H, s, $H^{17}$). $^{13}$C NMR (101 MHz, $CDCl_3$): δ 193.8 ($C^9$), 167.9 ($C^{15}$), 158.4 ($C^{13}$), 153.1 ($C^2$), 148.5 ($C^{8'}$), 142.8 ($C^7$), 137.1 ($C^4$), 133.0 ($C^{10}$), 128.9 ($C^{4'}$), 128.8 ($C^6$), 128.7 ($C^{11}$), 128.3 ($C^5$), 127.1 ($C^8$), 117.2 ($C^3$), 115.3 ($C^{12}$), 82.6 ($C^{16}$), 65.8 ($C^{14}$), 28.1 ($C^{17}$). ESI-MS (*m/z)*: Found $[M + H]^+$ 364.1539, calc $[C_{22}H_{21}NO_4 + H]^+$ 364.1543.

7-(4-(1,1-dimethylethyl)-phenoxyacetate)-2-quinolinemethanol (**3d**)

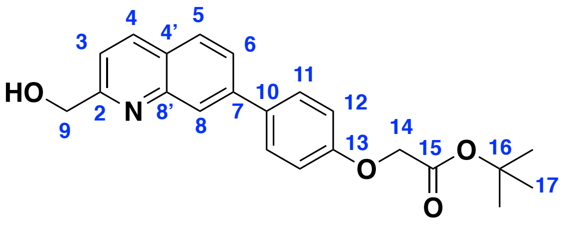

7-(4-(1,1-Dimethylethyl)-phenoxyacetate)-2-quinolinecarboxaldehyde (0.38, 1.05 mmol) was dissolved in anhydrous methanol (20 mL) under a nitrogen atmosphere. The flask was cooled to 0 °C, sodium borohydride (0.05 g, 1.26 mmol) was carefully added as one solid, and the reaction was allowed to warm to room temperature and stirred for 1 hour. The reaction was quenched with saturated $NH_4Cl$ solution (20 mL) then the methanol was evaporated under reduced pressure. The remaining aqueous solution was extracted with chloroform (3 x 50 mL), organics combined, washed with brine (2 x 50 mL), dried ($MgSO_4$) and the solvent evaporated under reduced pressure to yield the product as a yellow solid (0.38 g, 99%). $^1$H NMR (400 MHz, $CDCl_3$): δ 8.23 (1H, s, $H^8$), 8.13 (1H, d, *J* = 8.2 Hz, $H^4$), 7.85 (1H, d, *J* = 8.7 Hz, $H^5$), 7.77 (1H, d, *J* = 8.7 Hz, $H^6$), 7.69 (2H, d, *J* = 8.7 Hz, $H^{11}$), 7.27–7.25 (1H, m, $H^3$), 7.03 (2H, d, *J* = 8.7 Hz, $H^{12}$), 4.93 (2H, s, $H^9$), 4.59 (2H, s, $H^{14}$), 1.51 (9H, s, $H^{17}$). $^{13}$C NMR (101 MHz, $CDCl_3$): δ 168.0 ($C^{15}$), 159.5 ($C^2$), 158.1 ($C^{13}$), 147.1 ($C^{8'}$), 142.2 ($C^7$), 136.6 ($C^4$), 133.6 ($C^{10}$), 128.7 ($C^{11}$), 128.1 ($C^5$), 126.5 ($C^{4'}$), 125.9 ($C^6$), 125.6 ($C^8$), 118.1 ($C^3$), 115.2 ($C^{12}$), 82.6 ($C^{16}$), 65.8 ($C^{14}$), 64.2 ($C^9$), 28.1 ($C^{17}$). ESI-MS (*m/z)*: Found $[M + H]^+$ 366.1700, calc $[C_{22}H_{23}NO_4 + H]^+$ 366.1700.

7-(4-(1,1-Dimethylethyl)-phenoxyacetate)-2-methanesulfonate-2-quinolinemethanol (**4d**)

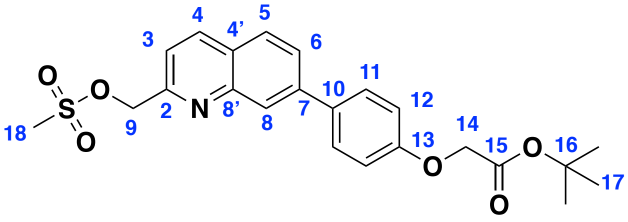

In an oven-dried Schlenk, 7-(4-(1,1-dimethylethyl)-phenoxyacetate)-2-quinolinemethanol (0.38 g, 1.04 mmol) was dissolved in anhydrous dichloromethane(10 mL) under a nitrogen atmosphere. Diisopropylethylamine (0.37 mL, 2.14 mmol) and methanesulfonyl chloride (0.08 mL, 1.02 mmol) were added and the mixture was stirred at room temperature for 2 hours. Water (20 mL) was added, and the organic layer separated. The aqueous layer was extracted with dichloromethane(2 x 30 mL) and the combined organic layers were washed with brine (100 mL), the organic layer dried ($MgSO_4$) and concentrated under reduced pressure, to give the product as a yellow oil (0.44 g, 97%). $^1$H NMR (400 MHz, $CDCl_3$): δ 8.25–8.23 (2H, m, $H^8$, $H^4$), 7.90 – 7.81 (2H, m, $H^5$, $H^6$), 7.69 (2H, d, *J* = 8.7 Hz, $H^{11}$), 7.56 (1H, d, *J* = 8.2 Hz, $H^3$), 7.03 (2H, d, *J* = 8.7 Hz, $H^{12}$), 5.51 (2H, s, $H^9$), 4.59 (2H, s, $H^{14}$), 3.13 (3H, s, $H^{18}$), 1.51 (9H, s, $H^{17}$). $^{13}$C NMR (101 MHz, $CDCl_3$): δ 168.0 ($C^{15}$), 158.2 ($C^{13}$), 154.4 ($C^2$), 148.1 ($C^{8'}$), 142.5 ($C^7$), 137.2 ($C^4$), 133.4 ($C^{10}$), 128.7 ($C^{11}$), 128.1 ($C^5$), 126.8 ($C^6$), 126.6 ($C^{4'}$), 126.2 ($C^8$), 119.4 ($C^3$), 115.2 ($C^{12}$), 82.6 ($C^{16}$), 72.2 ($C^9$), 65.8 ($C^{14}$), 38.2 ($C^{18}$), 28.1 ($C^{17}$). LR-MS ESI (*m/z*): Found $[M + H]^+$ 444.1, calc $[C_{23}H_{25}NO_6S + H]^+$ 444.1.

4,10-*Bis*((7-(4-(1,1-dimethylethyl)-phenoxyacetate)-quinolin-2-yl)-methyl)-1,4,7,10-tetraazacyclododecane-1,7-diyl)-diacetate (**5d**)

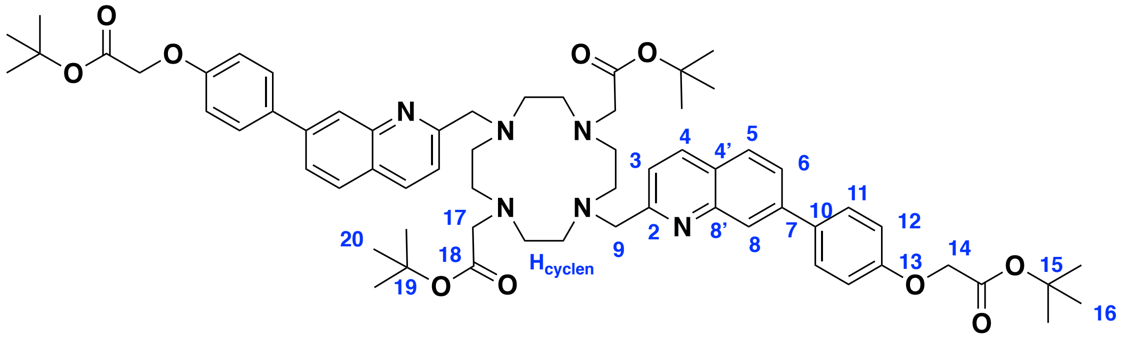

To a solution of DO2A-*tert*-butyl ester (138 mg, 0.35 mmol) and potassium carbonate (143 mg, 1.04 mmol) in anhydrous acetonitrile (10 mL), was added 4-(4-(1,1-dimethylethyl)-phenoxyacetate)- 2-methanesulfonate-2-quinolinemethanol (420 mg, 0.95 mmol). The reaction mixture was stirred at 60 °C for 18 hours. The reaction was cooled to room temperature, salts removed through centrifugation (1500 rpm for 5 minutes). The organic layer was removed, and the salts washed with acetonitrile (2 x 10 mL). The organic layers combined, and the solvent removed under reduced pressure. The crude material was purified by column chromatography (silica gel; neat dichloromethane to 98:2 dichloromethane/methanol) to give the desired protected ligand, as a yellow solid (132 mg, 35%). $^{1}$H-NMR (500 MHz, $CDCl_3$): δ 8.25 (2H, d, *J* = 8.2 Hz, $H^{4}$), 8.07 (2H, s, $H^{8}$), 7.84 (2H, d, *J* = 8.2 Hz $H^{5}$), 7.52 (2H, dd, *J* = 8.4 Hz, 1.4 Hz, $H^{6}$), 7.37 (2H, d, *J* = 8.2 Hz, $H^{3}$), 6.62 (4H, d, *J* = 8.7 Hz, $H^{11}$), 6.43 (4H, d, *J* = 8.7 Hz, $H^{12}$), 4.41 (4H, s, $H^{14}$), 2.92–2.58 (12H, m, $H^{18}$, $H^{cyclen}$), 1.66–1.63 (8H, m, $H^{cyclen}$), 1.48 (18H, s, $H^{17}$), 0.87 (18H, s, $H^{21}$). $^{13}$C NMR (126 MHz, $CDCl_3$): δ 171.4 ($C^{19}$), 167.8 ($C^{15}$), 160.2 ($C^{2}$), 157.6 ($C^{13}$), 148.3 ($C^{8'}$), 141.9 ($C^{7}$), 137.2 ($C^{4}$), 133.1 ($C^{10}$), 128.2 ($C^{11}$), 128.1 ($C^{5}$), 126.4 ($C^{4'}$), 126.2 ($C^{8}$), 126.1 ($C^{6}$), 121.4 ($C^{3}$), 114.5 ($C^{12}$), 82.6 ($C^{16}$), 81.9 ($C^{20}$), 65.6 ($C^{14}$), 60.1 ($C^{9}$), 58.4 ($C^{18}$), 51.4 ($C^{cyclen}$), 50.5 ($C^{cyclen}$), 28.1 ($C^{17}$), 27.6 ($C^{21}$). ESI-MS (*m/z)*: Found $[M + H]^{+}$ 1095.6160, calc $[C_{64}H_{82}N_6O_{10} + H]^{+}$ 1095.6165; Found $[M + Na]^{+}$ 1117.5980, calc $[C_{64}H_{82}N_6O_{10} + Na]^{+}$ 1117.5985; Found $[M + 2H]^{2+}$ 548.3118, calc $[C_{64}H_{82}N_6O_{10} + 2H]^{2+}$ 548.3119.

4,10-*Bis*((7-(4-(1,1-dimethylethyl)-phenoxyacetate)-quinolin-2-yl)-methyl)-1,4,7,10-tetraazacyclododecane-1,7-diyl)-diacetic acid (**6d**)

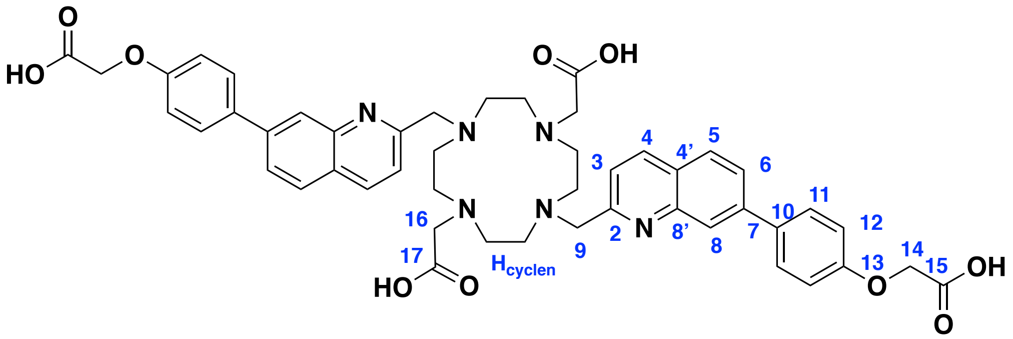

To a solution of 4,10-*bis*((7-(4-(1,1-dimethylethyl)-phenoxyacetate)-quinolin-2-yl)-methyl)-1,4,7,10-tetraazacyclododecane-1,7-diyl)-diacetate (78 mg, 71 μmol) in dichloromethane (2 mL) was added trifluoroacetic acid (2 mL). The reaction mixture was stirred at room temperature for 16 hours and the trifluoroacetic acid was co-evaporated with dichloromethane (10 x 20 mL) to give the deprotected ligand as a yellow residue (77 mg, 99%). $^{1}$H NMR (500 MHz, $CD_3OD$): δ 8.38 (2H, d, *J* = 8.2 Hz, $H^{4}$), 7.99 (2H, s, $H^{8}$), 7.78

(2H, d, $J$ = 8.7 Hz, $H^5$), 7.75 (2H, d, $J$ = 8.2 Hz, $H^6$), 7.50 (2H, d, $J$ = 8.7 Hz, $H^3$), 7.07 (4H, d, $J$ = 8.7 Hz, $H^{11}$), 6.53 (4H, d, $J$ = 8.7 Hz, $H^{12}$), 5.12 (4H, s, $H^9$), 4.61 (4H, s, $H^{14}$), 3.80 – 3.70 (12H, m, $H^{16}$, $H^{cyclen}$), 3.45 – 3.38 (8H, m, $H^{cyclen}$). $^{13}$C NMR (126 MHz, $CD_3OD$): δ 171.9 ($C^{17}$), 171.2 ($C^{15}$), 157.9 ($C^{13}$), 151.1 ($C^2$), 146.9 ($C^{8'}$), 142.3 ($C^7$), 138.3 ($C^4$), 132.2 ($C^{10}$), 128.1 ($C^5$), 127.9 ($C^{11}$), 126.9 ($C^{4'}$), 126.2 ($C^6$), 124.8 ($C^8$), 119.2 ($C^3$), 114.1 ($C^{12}$), 64.5 ($C^{14}$), 58.0 ($C^9$), 53.3 ($C^{16}$), 51.7 ($C^{cyclen}$). ESI-MS (*m/z)*: Found $[M + H]^+$ 871.3659, calc $[C_{48}H_{50}N_6O_{10} + H]^+$ 871.3661; Found $[M + Na]^+$ 893.3480, calc $[C_{48}H_{50}N_6O_{10} + Na]^+$ 893.3481; Found $[M + 2H]^{2+}$ 436.1867, calc $[C_{48}H_{50}N_6O_{10} + 2H]^{2+}$ 436.1867.

**$[Eu.7PhOCH_2COO]^-$**

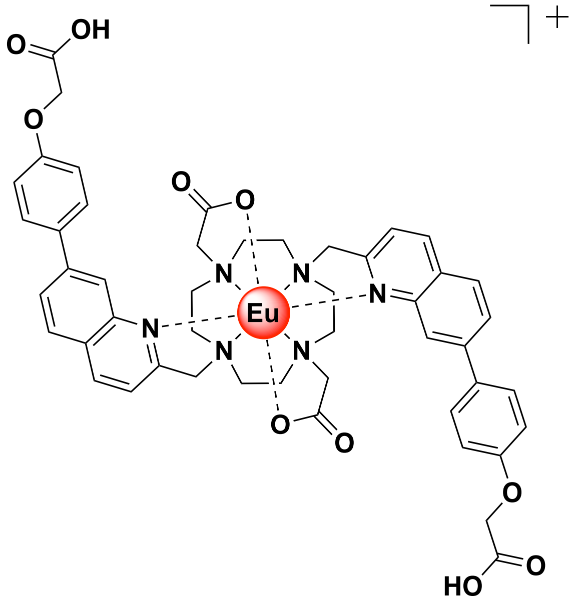

The deprotected ligand (65 mg, 0.060 mmol) was dissolved in deionised water (2 mL) and the pH adjusted to pH 7.5 using 0.1 M NaOH solution. $EuCl_3.6H_2O$ (41 mg, 0.112 mmol) was then added and the pH was readjusted to 7.5, before stirring the solution at 60°C for 72 hours. The reaction was cooled to room temperature, then the solvent volume was reduced by half under reduced pressure. The resulting solution was purified by preparative RP-HPLC [gradient: 0 − 100% acetonitrile in 100 mM $NH_4HCO_3$ over 20 minutes, at 17 mL per minute; $t_R$ = 7.08 min] to give the complex $[Eu\text{-}7\text{-}suzPhOCH_2COO]^-$ as a pale-yellow solid (10 mg, 17%). $^1$H-NMR (500 MHz, $CDCl_3$): δ 48.0, 38.9, 38.2, 37.8, 35.1, 32.3, 20.2, 20.0, 18.5, 18.2, 17.8, 17.5, 16.7, 15.7, 14.7, 14.4, 14.2, 14.1, 13.6, 13.2, 12.5, 12.0, 11.9, 11.7, 11.7, 11.5, 11.3, 10.9, 10.4, 10.0, 9.4, 9.2, 8.9, 8.7, 8.7, 8.5, 8.3, 8.3, 8.2, 8.0, 7.9, 7.9, 7.8, 7.8, 7.7, 7.6, 7.5, 7.5, 7.4, 7.4, 7.2, 7.2, 7.1, 7.1, 6.6, 6.6, 6.5, 6.5, 5.6, 5.4, 5.3, 5.1, 5.1, 4.9, 4.9, 4.5, 4.4, 4.3, 4.2, 4.2, 3.6, 3.6, 3.5, 3.4, 3.4, 3.1, 3.1 , 3.0, 2.6, 2.2, 2.2, 2.2, 2.1, 2.0, 1.9, 1.6, 1.3, 1.3, 1.2, 1.1, 1.1, 1.1, 0.9, 0.9, 0.9, 0.2, 0.1, -1.1, -1.6, -2.0, -3.5, -4.6, -5.0, -5.9, -7.0, -8.5, -9.8, -10.7, -11.0, -13.6, -14.5, -15.2, -15.7, -16.1, -18.6, -18.9, -19.8, -20.9, -22.1, -22.8, -23.5, -24.4, -27.5, -28.5, -29.0. ESI-MS (*m/z*): Found $[M]^+$ 1021.2640, calc $[C_{48}H_{48}N_6O_{10}Eu]^+$ 1021.2639. Photophysical data measured in methanol: $\lambda_{max}$ = 350 nm, $\varepsilon$ = 18000 $M^{-1}$ $cm^{-1}$, $\Phi_{em}$ = 0.2%.

## 3. X-ray Crystallography

**[Eu.4PhOMe]+**

Small colourless block crystals were obtained after slow evaporation of the compound dissolved in methanol-*d₄* and water (9:1). The complex crystallised in the Monoclinic space group, $P2_1/n$ with the asymmetric unit consisting of one structure of the complex with a coordinated water molecule to the Eu(III) in the axial site and a triflate counter ion. All non-hydrogen atoms were refined anisotropically and all hydrogen atoms were geometrically placed and refined using a riding model.

*Disorder and Refinement Special Details*

The crystal was weakly diffracting, and data was collected on I19 at Diamond Light Source. The crystal was found to be twinned, with one major component, one minor component, and many components of *ca* 10% of less of the found reflections. Attempts were made to separate the components and integrate the data as a twin, but these attempts were unsuccessful. There remains a large peak of electron density (*ca* 12 or 19 e/A$^3$), that sits approx. 5.8 Å from the Eu(III) centre in the asymmetric unit, and 5 Å from the nearest neighbouring Eu(III) centre. The spacing between these peaks is the same (10.99 Å) as that between the Eu centres, leading us to believe that there is a very small amount of disorder, thus the Eu(III) has been modelled with disorder over two sites, with the largest component being 93% (located in the cyclen unit). The macrocycle is disordered over two sites, and the parts have been fixed with occupancies of 0.65 and 0.35 for parts 1 and 2, respectively. To aid refinement of the model, the anisotropic displacement paraments of the nitrogen atoms (N3A, N4A, N5A, N6A) and carbon atoms (C39A, C40A, C41A, C42A, C43A, C44A, C45A and C46A) of the cyclen ring were constrained to be identical (EADP), due to the low occupancy, overlapping with the other atoms in close proximity to the Eu atom. The triflate anion has been modelled with disorder over two sites. Firstly, the anion atoms were split and refined, however, due to the two components overlapping and restraints required, this disorder was addressed by using the fragment database in Olex2. This allowed for the appropriate restraints to be applied (DFIX, DANG and SADI) for each component. The occupancies have been fixed at 0.65 and 0.35. Due to the quality of the data, the non-coordinating water and methanol molecules could not be modelled, therefore a solvent mask was used to confirm the presence of 6 $H_2O$ and 1 $CH_3OH$ (volume of 1052 cubic angstroms in 1 void per unit cell, which is consistent with the presence of six water molecules and one methanol molecule per asymmetric unit, which accounts for 312 electrons per unit cell). The anisotropic displacement parameter of the minor triflate anion and the minor Eu(III) atom components is restrained to have more isotropic character (ISOR).

*Crystal Packing*

The methoxy-phenyl rings are twisted out of the plane of the quinoline rings (56.87(19)° and 57.82(19)°). These large twists and the positioning of the triflate counter ions around the complex structure, limits the complex to exhibit any intermolecular π-π interactions. Non-classical hydrogen bonding interactions are observed between the counter anions and the complex structure (C-H•••F and C-H•••O).

Deposited cif number: 2464248

**Table S1**. Crystal data and structure refinement details.

| **Compound** | **[Eu.4PhOMe]$^+$** |
| --- | --- |
| Empirical formula | $C_{48}H_{66}EuF_3N_6O_{17}S$ |
| Formula weight | 1240.08 |
| Temperature/K | 100.15 |
| Crystal system | monoclinic |
| Space group | $P2_1/n$ |
| a/Å | 13.5915(4) |
| b/Å | 25.6668(11) |
| c/Å | 15.2393(5) |
| α/° | 90 |
| β/° | 97.314(3) |
| γ/° | 90 |
| Volume/Å$^3$ | 5273.0(3) |
| Z | 4 |
| $\rho_{calc}$g/cm$^3$ | 1.562 |
| μ/mm$^{-1}$ | 1.217 |
| F(000) | 2552.0 |
| Crystal size/mm$^3$ | 0.31 × 0.06 × 0.05 |
| Radiation | synchrotron (λ = 0.6889) |
| 2Θ range for data collection/° | 3.03 to 48.416 |
| Index ranges | -16 ≤ h ≤ 16, -30 ≤ k ≤ 30, -18 ≤ l ≤ 18 |
| Reflections collected | 56990 |
| Independent reflections | 9299 [$R_{int}$ = 0.1071, $R_{sigma}$ = 0.0872] |
| Data/restraints/parameters | 9299/372/745 |
| Goodness-of-fit on F$^2$ | 1.098 |
| Final R indexes [I>=2σ (I)] | $R_1$ = 0.0724, $wR_2$ = 0.1901 |
| Final R indexes [all data] | $R_1$ = 0.0753, $wR_2$ = 0.1922 |
| Largest diff. peak/hole / e Å$^{-3}$ | 1.67/-1.53 |

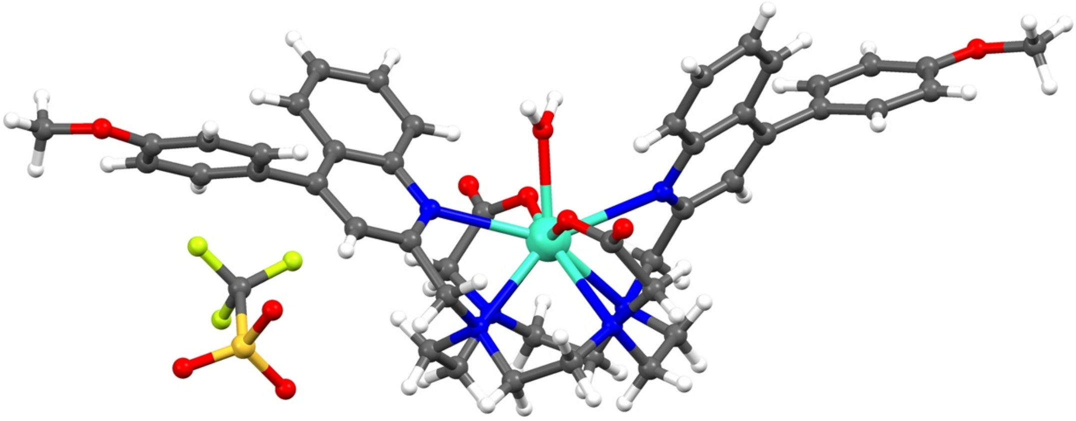

**Figure S1**. Single crystal X-ray structure of [**Eu.4PhOMe**]$^+$, displaying the asymmetric unit. The lowest occupancy disorder of the macrocycle, triflate and Eu(III) atom, have been omitted for clarity. Atom colours: Eu red, C grey, N blue, O red, H white, S yellow, F green.

Compound **3c**

Yellow block-like crystals were obtained after slow evaporation of the compound dissolved in dichloromethane and methanol (1:1).

The structure solved in the Monoclinic space group, $P2_1/c$ with one molecule in the asymmetric unit (Figure S2). All non-hydrogen atoms were refined anisotropically and all hydrogen atoms were geometrically placed and refined using a riding model. The phenyl ring is twisted out of the plane of the quinoline ring by 30.29(3)°. The hydroxyl group is located out of the plane of the quinoline ring, which creates modest hydrogen-bonding, creating a ten-membered ring network between a neighbouring hydroxyl group (Figure S3, Table S3). The molecules stack along the *b* axis, with π-π interactions between the quinoline rings (Table S4).

Deposited cif number: 2464162

**Table S2.** Crystal data and structure refinement details for compound **3c**.

| **Compound** | **3c** |
|---|---|
| Empirical formula | $C_{17}H_{15}NO_2$ |
| Formula weight | 265.30 |
| Temperature/K | 99.9(6) |
| Crystal system | monoclinic |
| Space group | $P2_1/c$ |
| Unit cell dimensions: a / Å | 11.15720(10) |
| b / Å | 7.76420(10) |
| c / Å | 15.45920(10) |
| α / ° | 90 |
| β / ° | 100.3210(10) |
| γ / ° | 90 |
| Volume / $Å^3$ | 1317.51(2) |
| Z | 4 |
| Density (*calc)* / $cm^3$ | 1.337 |
| Absorption coeff. / $mm^{-1}$ | 0.088 |
| *F*(000) | 560.0 |
| Crystal size / $mm^3$ | 0.220 × 0.140 × 0.120 |
| Radiation | Mo $K_{\alpha}$ (λ = 0.71075) |
| 2Θ range for data collection / ° | 3.71 to 63.762 |
| Index ranges | -16 ≤ h ≤ 16, -11 ≤ k ≤ 11, -22 ≤ l ≤ 22 |
| Reflections collected | 66258 |
| Independent reflections | 4414 [$R_{int}$ = 0.0302, $R_{sigma}$ = 0.0124] |
| Data/restraints/parameters | 4414/0/183 |
| Goodness-of-fit on $F^2$ | 1.051 |
| Final R indexes ( $I > 2\sigma(I)$ ) | $R_1$ = 0.0428, $wR_2$ = 0.1198 |
| Final R indexes (all data) | $R_1$ = 0.0501, $wR_2$ = 0.1268 |
| Largest diff. peak/hole / e $Å^{-3}$ | 0.39/-0.20 |

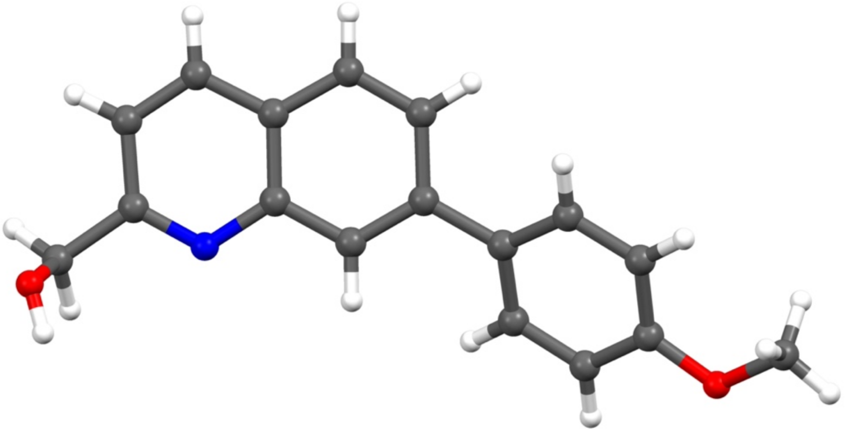

**Figure S2.** Single crystal X-ray structure of compound **3c**. Atom colours: C grey, N blue, O red, H white.

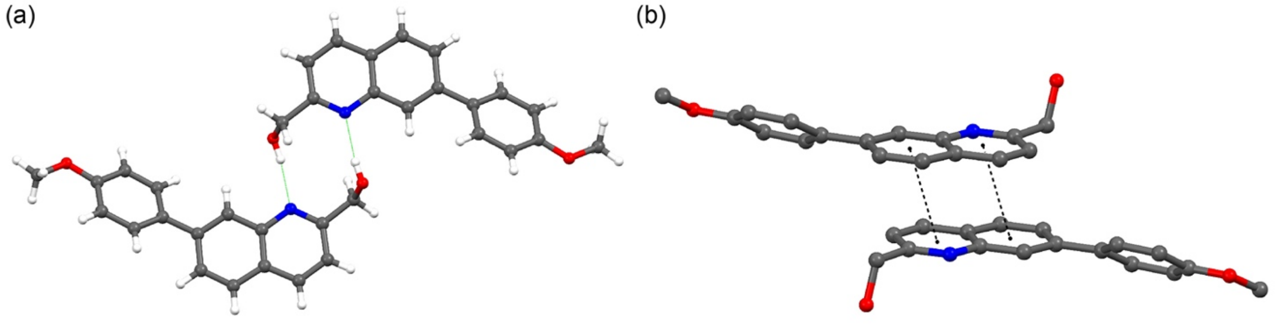


**Figure S3.** Single crystal X-ray structure of compound **3c** displaying intermolecular (a) hydrogen bonding and (b) π-π stacking interactions in the packing of the molecules. The hydrogen atoms in (b) have been omitted for clarity. Atom colours: C grey, N blue, O red, H white.

**Table S3.** Hydrogen bonds in the packing of compound **3c**.

| D | H | A | d(D-H) / Å | d(H-A) / Å | d(D-A) / Å |
|---|---|---|---|---|---|
| O | H | N | 0.8400(7) | 1.9894(6) | 2.8041(9) |

**Table S4.** Face-to-face π-π interactions observed in the packing of compound **3c**.

| Interaction | centroid-to-centroid / Å | plane-to-plane shift / Å | twist angle / ° |
|---|---|---|---|
| quinoline ••• quinoline | 3.7176(7) | 1.4886(13) | 0 |
| quinoline ••• quinoline | 3.8049(6) | 1.6948(13) | 0.34(6) |

**[Eu.7PhOMe]$^+$**

Pale-yellow coloured crystals were obtained after slow evaporation of the compound dissolved in methanol/water (1:1). The complex crystallised in the Monoclinic space group, $P2_1/c$ with the asymmetric unit consisting of two structures of the complex with one of the carboxylate oxygen atoms coordinated to the Eu(III) centre of the other, forming an interwoven type structure, and a non-coordinated triflate counter ion. The other triflate anion was disordered over multiple sites, which could not be modelled due to the close proximity of non-coordinating water and methanol molecules in the asymmetric unit. All non-hydrogen atoms were refined anisotropically and all hydrogen atoms were geometrically placed and refined using a riding model.

*Disorder and Refinement Special Details*

There is disorder within the two complex structures which has been modelled. One of the quinoline-phenyl groups on each Eu(III) complex structure is disordered over two sites. The quinoline and phenyl-methoxy components on the Eu1 complex structure have been fixed with occupancies of 0.55 (part 1) and 0.45 (part 2). However, the occupancies of the disorder on the Eu2 structure were refined as free variables rather than fixed, as attempts to constrain them to specific values resulted in an unstable model (largest occupancy being 51%). To aid refinement of the model the anisotropic displacement parameters of the nitrogen and carbon atoms of the quinoline and phenyl rings were constrained to be identical (EADP), due to overlapping with other atoms in close proximity. The anisotropic displacement parameter of the disordered quinoline-phenyl components have been restrained to have more isotropic character (ISOR). There is also some minor disorder of the carbonyl groups, one on each complex structure. These have been fixed with occupancies of 0.75 (part 1) and 0.25 (part 2) and 0.59 (part 1) and 0.41 (part 2) for complex structures Eu1 and Eu2, respectively. The triflate anion located within the asymmetric unit was identified from electron density, and the Olex2 fragment database was employed to confirm and refine its geometry, ensuring chemically appropriate conformation. Due to the significant disorder around the complex in the asymmetric unit, the non-coordinating water and methanol molecules could not be modelled, as well as the second triflate anion (due to overlapping electron density peaks of these solvent molecules), therefore, a solvent mask was applied and confirmed the presence of 1 $SO_3CF_3^-$, 17 $H_2O$ and 2 $CH_3OH$ (volume of 3036 cubic angstroms in 1 void per unit cell, which is consistent with the presence of one triflate anion, two methanol molecules and seventeen water molecules per asymmetric unit which accounts for 326 electrons per unit cell).

*Crystal Packing*

The methoxy-phenyl rings are twisted out of the plane of the quinoline rings, ranging from 18° to 47°. The reduced twisting of these rings allows the structures to engage in intra- and intermolecular face-to-face π-π interactions. Together with the coordination of the carboxylate oxygen to the axial site of the Eu(III) centre, these interactions promote the association of the two complex molecules within the crystal structure. Non-classical hydrogen bonding is observed within the asymmetric unit between the carboxylate oxygen atoms and the C-H groups of the quinoline arms. There are also weak non-classical hydrogen bonding interactions between the Eu(III) complex C-H groups in the crystal packing with the triflate anion (closest interaction F2•••H74B-C74 146.1(5)°, F2•••C74 3.255(8) Å) and a carboxylate oxygen atom (O8B•••H8B-C8 156.7(6)°, O8B•••C8 2.27(2) Å).

Deposited cif number: 2464160

**Table S5**. Crystal data and structure refinement details.

| **Compound** | **[Eu.7PhOMe]⁺** |
|---|---|
| Empirical formula | $C_{96}H_{138}Eu_2F_6N_{12}O_{37}S_2$ |
| Formula weight | 2534.22 |
| Temperature/K | 100.00(10) |
| Crystal system | monoclinic |
| Space group | $P2_1/c$ |
| Unit cell dimensions: a / Å | 19.3306(3) |
| b / Å | 15.9227(2) |
| c / Å | 34.8428(7) |
| α / ° | 90 |
| β / ° | 92.705(2) |
| γ / ° | 90 |
| Volume / Å$^3$ | 10712.5(3) |
| Z | 4 |
| Density (*calc*) / cm$^3$ | 1.571 |
| Absorption coeff. / mm$^{-1}$ | 9.521 |
| *F*(000) | 5224.0 |
| Crystal size / mm$^3$ | 0.424 × 0.037 × 0.03 |
| Radiation | Cu *K*α (λ = 1.54178) |
| 2Θ range for data collection / ° | 4.576 to 133.186 |
| Index ranges | -22 ≤ h ≤ 23, -14 ≤ k ≤ 18, -41 ≤ l ≤ 41 |
| Reflections collected | 110251 |
| Independent reflections | 18839 [$R_{int}$ = 0.1045, $R_{sigma}$ = 0.0637] |
| Data/restraints/parameters | 18839/747/1494 |
| Goodness-of-fit on $F^2$ | 1.020 |
| Final R indexes ( $I > 2\sigma(I)$ ) | $R_1$ = 0.0501, $wR_2$ = 0.1385 |
| Final R indexes (all data) | $R_1$ = 0.0644, $wR_2$ = 0.1453 |
| Largest diff. peak/hole / e Å$^{-3}$ | 1.06/-0.70 |

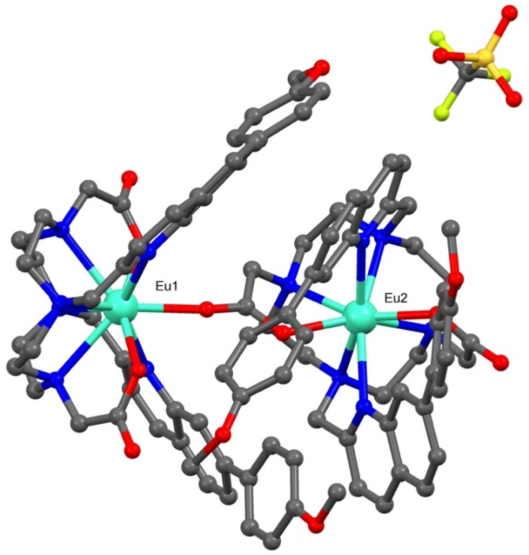


**Figure S4**. Single crystal X-ray structure of [**Eu.7PhOMe**]⁺, displaying the asymmetric unit. The lowest occupancy disorder has been omitted for clarity. Atom colours: Eu red, C grey, N blue, O red, S orange, F lime green.

## 4. $^1$H NMR studies of Eu(III) complexes

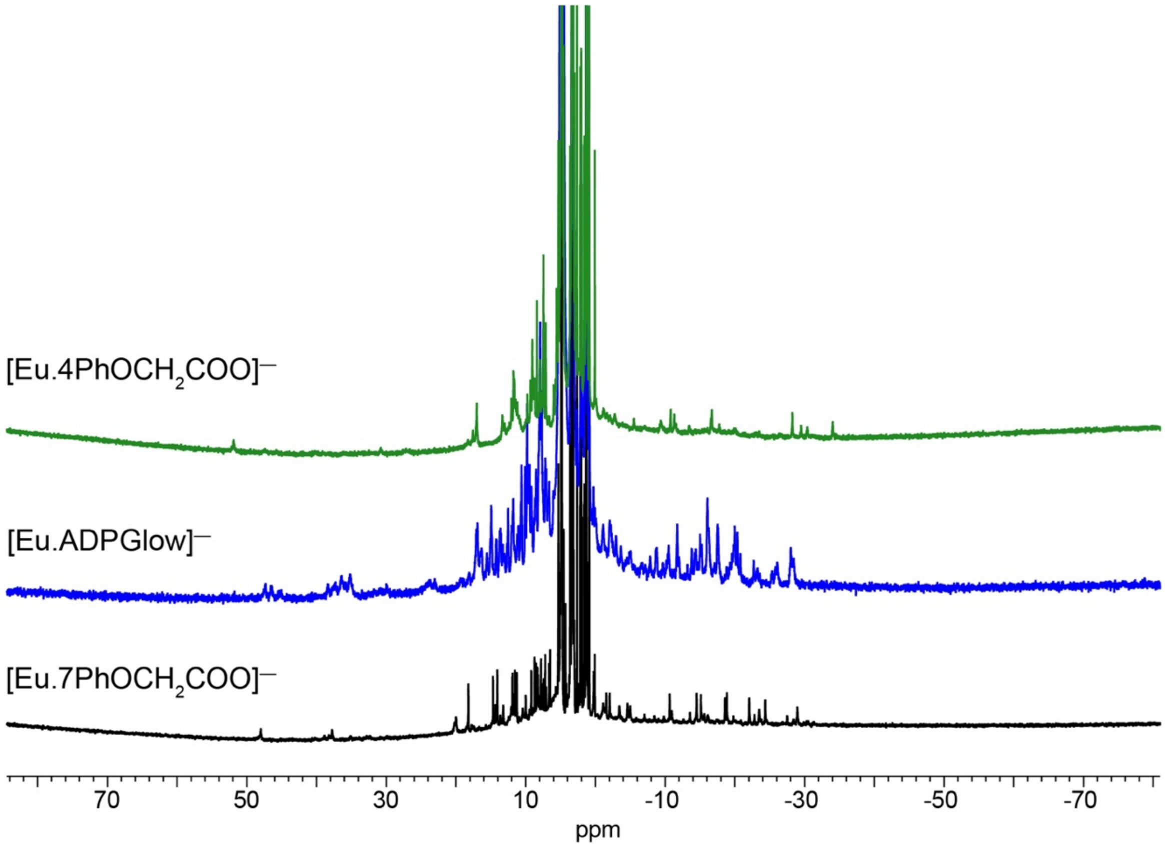


**Figure S5.** $^1$H NMR spectra (500 MHz, $CD_3OD$) of [**Eu.4PhOCH$_2$COO**]$^-$ (green), [**Eu.ADPGlow**]$^-$ (blue) and [**Eu.7PhOCH$_2$COO**]$^-$ (black) recorded at 298 K. Complex [**Eu.ADPGlow**]$^-$ was reproduced from reference 8.

## 5. Photophysical measurements of Eu(III) complexes

**Table S6**. Calculated values of the pure radiative lifetime ($\tau_{rad}$), radiative ($k_r$) and non-radiative ($\Sigma k_{nr}$) rate constants, intrinsic quantum yields ($\Phi_{Ln}$), and energy transfer efficiencies ($\eta_{ET}$) for the methoxyphenyl-substituted Eu(III) complexes, using the experimentally determined values $\tau_{obs}$, $\Phi_{em}$, and $I_{tot}$ / $I_{MD}$, in methanol.

| Complex | $\tau_{obs}$ (ms) [a] | $\Phi_{em}$[b] | $I_{tot}$ / $I_{MD}$[c] | $\tau_{rad}$ (ms) | $k_r$ ($s^{-1}$) | $\Sigma k_{nr}$ ($s^{-1}$) | $\Phi_{Ln}$ | $\eta_{ET}$ (%) |
|---|---|---|---|---|---|---|---|---|
| [**Eu.4PhOMe**]$^+$ | 0.86 | 0.055 | 9.615 | 3.21 | 311.5 | 851.3 | 0.27 | 20.5 |
| [**Eu.6PhOMe**]$^+$ | 0.88 | 0.006 | 8.561 | 3.61 | 277.4 | 859.0 | 0.24 | 2.5 |
| [**Eu.7PhOMe**]$^+$ | 0.99 | 0.004 | 8.690 | 3.55 | 281.6 | 728.5 | 0.28 | 1.4 |

[a] $\tau_{obs}$ determined from duplicate measurements in methanol at 295K. [b] $\Phi_{em}$ measured using quinine sulfate in 0.05 M $H_2SO_4$ as standard ($\Phi_{em}$ = 60%). [c] $I_{tot}$ / $I_{MD}$ is the relative contribution of the $^5D_0 \rightarrow {}^7F_1$ emission band (580–600 nm) to the total integrated emission intensity (550–720 nm).[3]

**Table S7**. Calculated values of the pure radiative lifetime ($\tau_{rad}$), radiative ($k_r$) and non-radiative ($\Sigma k_{nr}$) rate constants, intrinsic quantum yields ($\Phi_{Ln}$), and energy transfer efficiencies ($\eta_{ET}$) for the phenoxyacetate-substituted Eu(III) complexes, using the experimentally determined values $\tau_{obs}$, $\Phi_{em}$, and $I_{tot}$ / $I_{MD}$, in 10 mM HEPES buffer at pH 7.0.

| Complex | $\tau_{obs}$ (ms) [a] | $\Phi_{em}$[b] | $I_{tot}$ / $I_{MD}$[c] | $\tau_{rad}$ (ms) | $k_r$ ($s^{-1}$) | $\Sigma k_{nr}$ ($s^{-1}$) | $\Phi_{Ln}$ | $\eta_{ET}$ (%) |
|---|---|---|---|---|---|---|---|---|
| [**Eu.4PhOCH$_2$COO**]$^-$ | 0.47 | 0.024 | 7.105 | 4.34 | 230.2 | 1897.5 | 0.11 | 22.2 |
| [**Eu.ADPGlow**]$^-$ | 0.021 | 0.003 | 10.860 | 2.84 | 351.9 | 47267.2 | 0.007 | 4.0 |
| [**Eu.7PhOCH$_2$COO**]$^-$ | 0.016 | 0.002 | 9.869 | 3.13 | 319.7 | 62180.2 | 0.005 | 3.9 |

[a] $\tau_{obs}$ determined from duplicate measurements in 10 mM HEPES at pH 7.0 and 295K. [b] $\Phi_{em}$ measured using quinine sulfate in 0.05 M $H_2SO_4$ as standard ($\Phi_{em}$ = 60%). [c] $I_{tot}$ / $I_{MD}$ is the relative contribution of the $^5D_0 \rightarrow {}^7F_1$ emission band (580–600 nm) to the total integrated emission intensity (550–720 nm).[3]

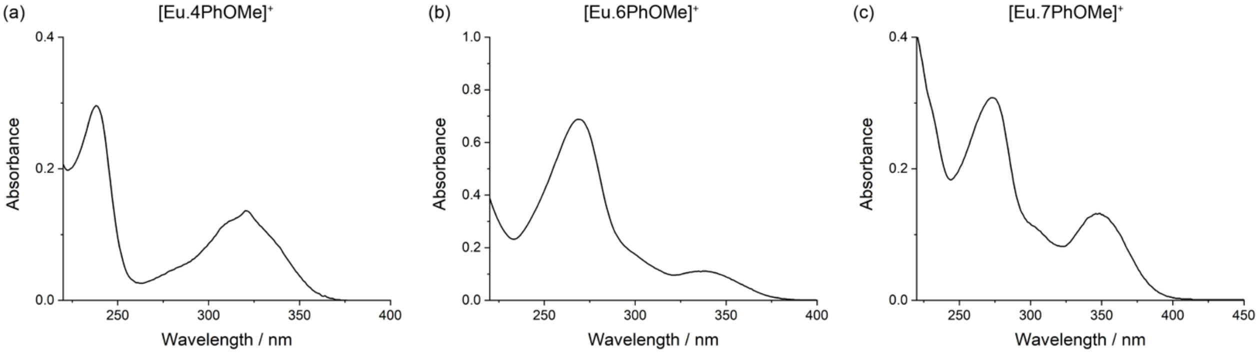


**Figure S6.** Absorption spectra of (a) [**Eu.4PhOMe**]$^+$, (b) [**Eu.6PhOMe**]$^+$ , and (c) [**Eu.7PhOMe**]$^+$ measured in methanol.

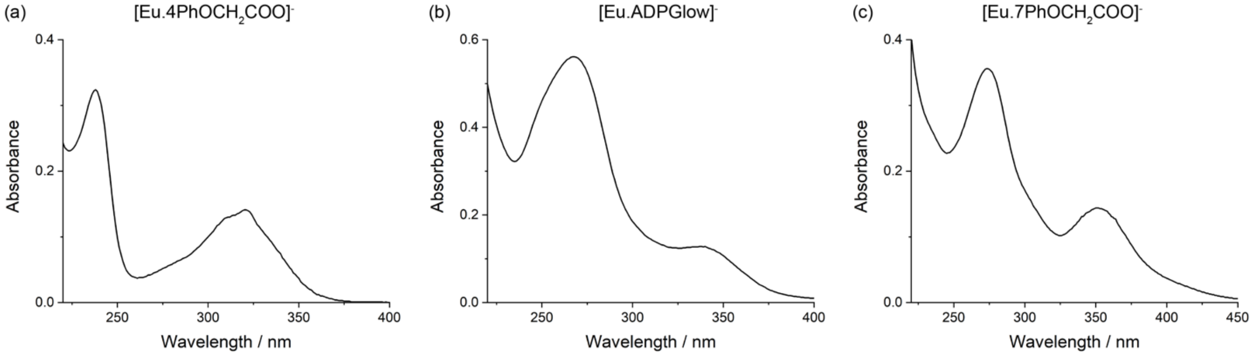


**Figure S7.** Absorption spectra of (a) [**Eu.4PhOCH$_2$COO**]$^-$, (b) [**Eu.ADPGlow**]$^-$, and (c) [**Eu.7PhOCH$_2$COO**]$^-$, measured in 10 mM HEPES at pH 7.0.

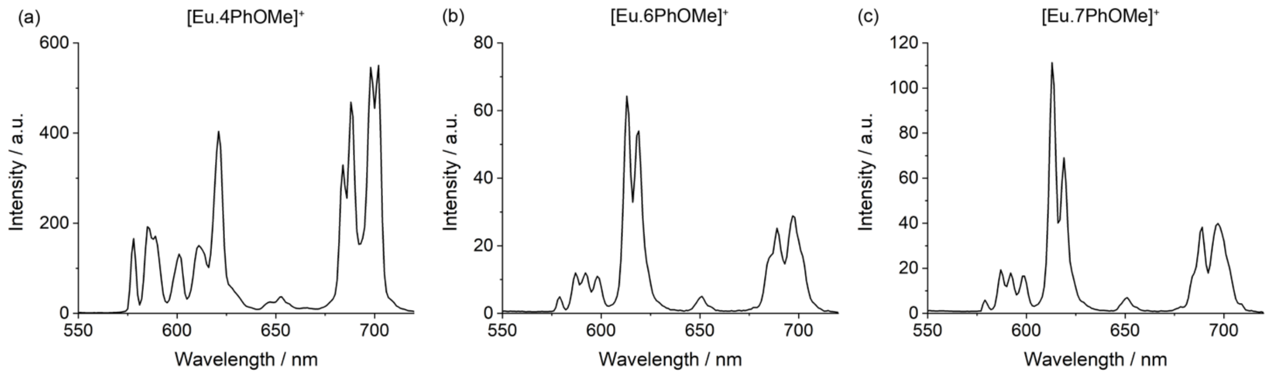


**Figure S8.** Emission spectra of (a) [**Eu.4PhOMe**]$^+$ ($\lambda_{exc}$ 321 nm), (b) [**Eu.6PhOMe**]$^+$ ($\lambda_{exc}$ 340 nm), and (c) [**Eu.7PhOMe**]$^+$ ($\lambda_{exc}$ 348 nm), measured methanol.

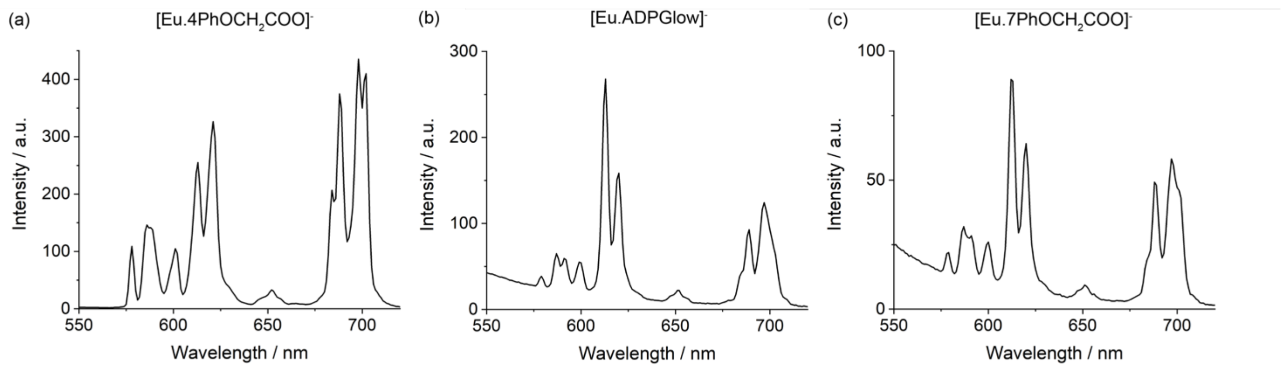


**Figure S9.** Emission spectra of (a) [**Eu.4PhOCH$_2$COO**]$^-$ ($\lambda_{exc}$ 321 nm), (b) [**Eu.ADPGlow**]$^-$ ($\lambda_{exc}$ 337 nm), and (c) [**Eu.7PhOCH$_2$COO**]$^-$ ($\lambda_{exc}$ 350 nm), measured in methanol. Reproduced from Figure 6 of the main manuscript.

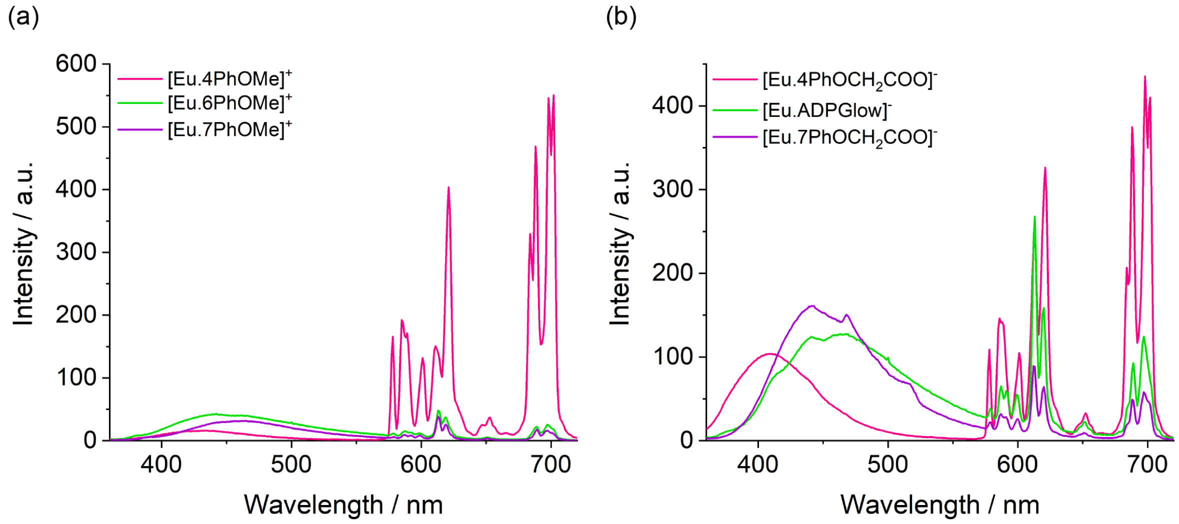


**Figure S10.** Emission spectra of the europium(III) complexes (0.1 Abs), measured in methanol at 295 K. Excitation: [**Eu.4PhOMe**]$^+$ ($\lambda_{exc}$ 321 nm), [**Eu.6PhOMe**]$^+$ ($\lambda_{exc}$ 340 nm), [**Eu.7PhOMe**]$^+$ ($\lambda_{exc}$ 348 nm), [**Eu.4PhOCH$_2$COO**]$^-$ ($\lambda_{exc}$ 321 nm), [**Eu.6PhOCH$_2$COO**]$^-$ ($\lambda_{exc}$ 337 nm), and [**Eu.7PhOCH$_2$COO**]$^-$ ($\lambda_{exc}$ 350 nm).

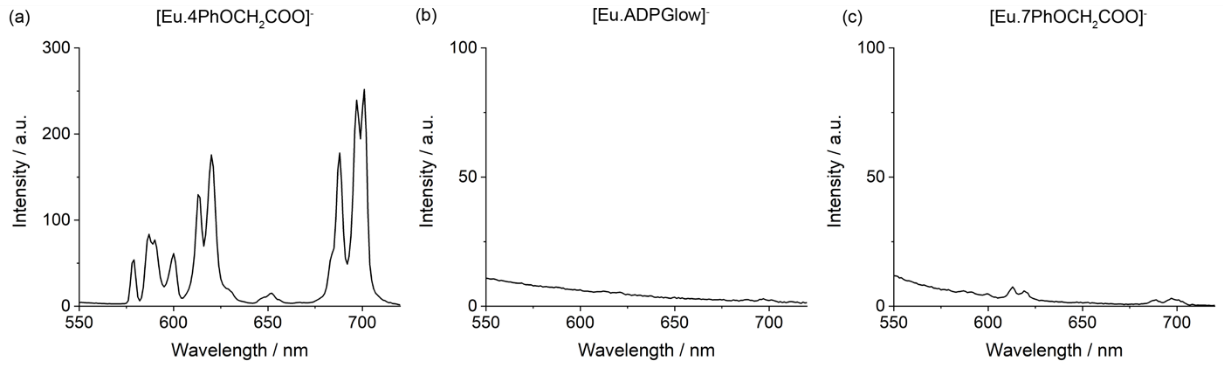


**Figure S11.** Emission spectra of (a) [**Eu.4PhOCH₂COO**]⁻ ($\lambda_{exc}$ 321 nm), (b) [**Eu.ADPGlow**]⁻ ($\lambda_{exc}$ 337 nm), and (c) [**Eu.7PhOCH₂COO**]⁻ ($\lambda_{exc}$ 350 nm), measured in 10 mM HEPES at pH 7.0.

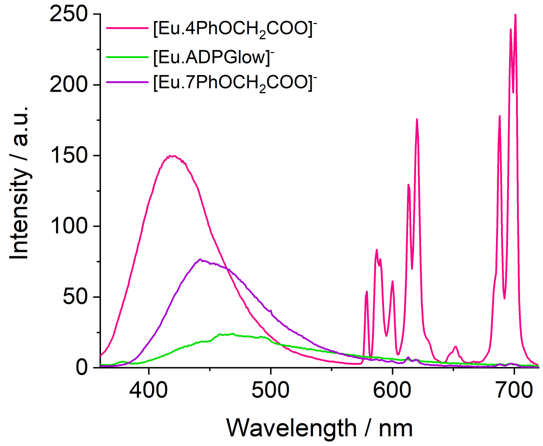


**Figure S12.** Emission spectra of the water-soluble europium(III) complexes (0.1 Abs). [**Eu.4PhOCH₂COO**]⁻ ($\lambda_{exc}$ 321 nm), [**Eu.ADPGlow**]⁻ ($\lambda_{exc}$ 337 nm), and [**Eu.7PhOCH₂COO**]⁻ ($\lambda_{exc}$ 350 nm), measured in 10 mM HEPES buffer at pH 7.0, 295 K.

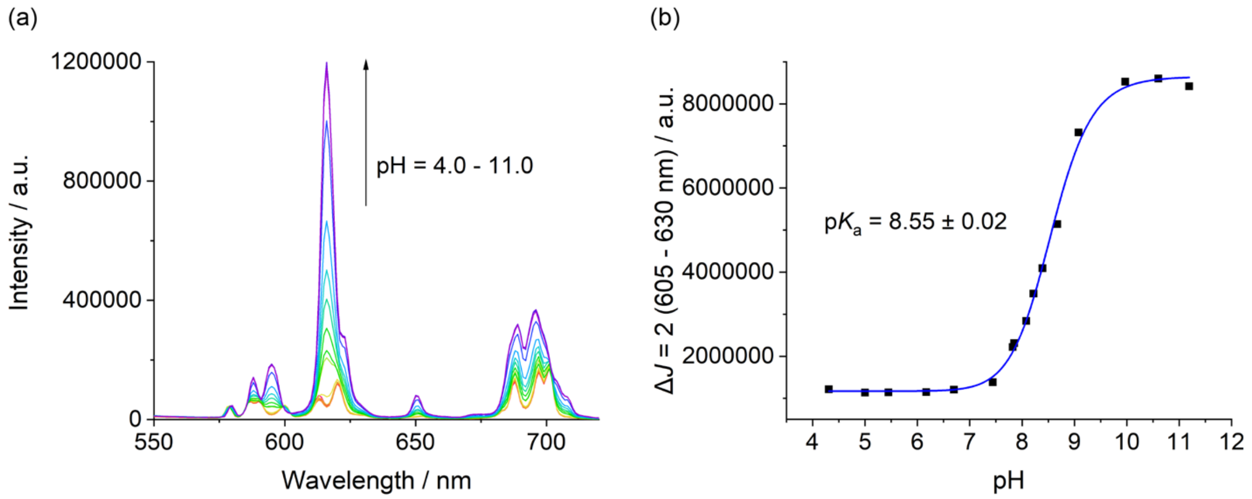


**Figure S13.** pH Titration of [**Eu.4PhOCH₂COO**]⁻. (a) Increase in emission intensity upon addition of NaOH solution, where the pH was adjusted incrementally by ~0.5 pH unit, (b) plot of emission intensity ($\Delta J = 2$) as a function of pH, showing the fit to the observed data. Measured in water, 295 K, 0.1 Abs, $\lambda_{exc}$ 321 nm.

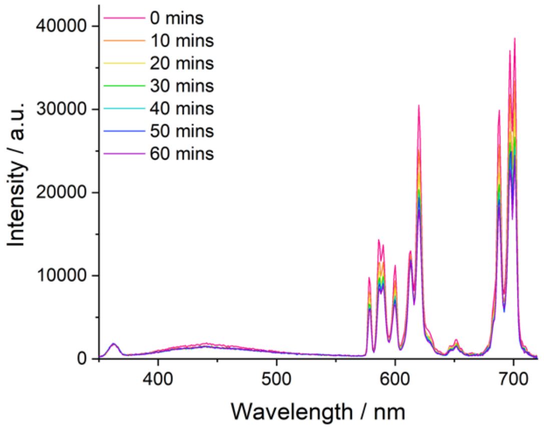


**Figure S14.** Stability of the emission response of (a) [**Eu.4PhOMe**]$^+$, (0.1 Abs) over an hour incubation period, measured in 9:1, 10 mM HEPES buffer at pH 7.0:methanol. $\lambda_{exc}$ 321 nm, 295 K.

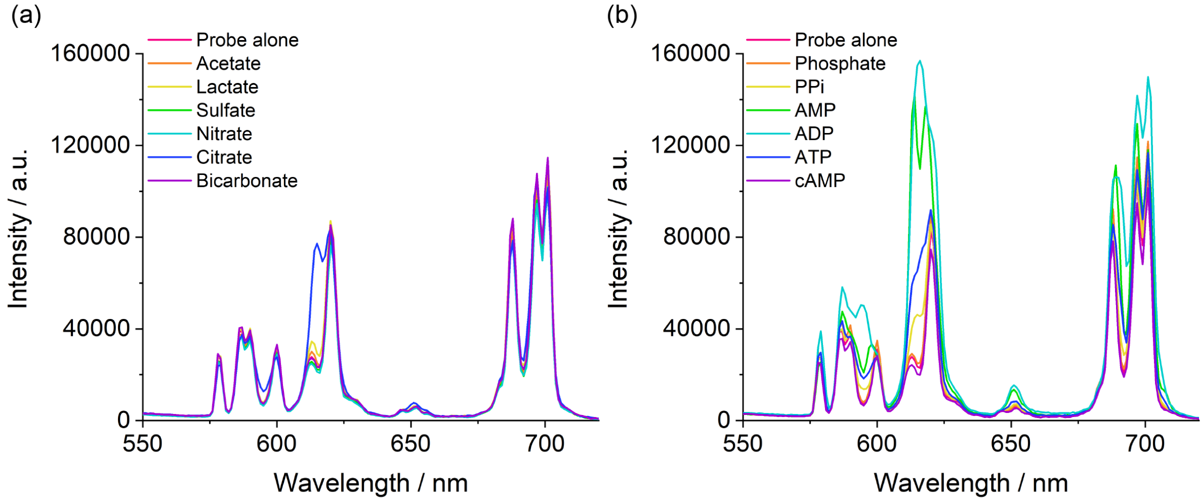


**Figure S15.** Selective emission enhancement of [**Eu.4PhOCH$_2$COO**]$^-$ (0.1 Abs, $\lambda_{exc}$ 321 nm) with (a) oxyanions (1 mM each: acetate, lactate, sulfate, nitrate, citrate, bicarbonate) and (b) phosphate containing anions (1 mM each: phosphate, pyrophosphate (PPi), adenosine monophosphate (AMP), adenosine diphosphate (ADP), adenosine triphosphate (ATP), cyclic adenosine monophosphate (cAMP)). Measured in 10 mM HEPES at pH 7.0 and 295 K.

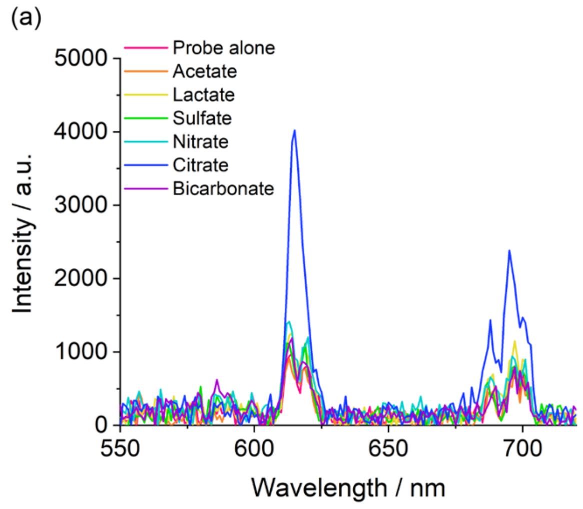


**Figure S16.** Selective emission enhancement of **[Eu.ADPGlow]**$^{-}$ (0.1 Abs, $\lambda_{exc}$ 321 nm) with (a) oxyanions (1 mM each: acetate, lactate, sulfate, nitrate, citrate, bicarbonate) and (b) phosphate containing anions (1 mM each: phosphate, pyrophosphate (PPi), adenosine monophosphate (AMP), adenosine diphosphate (ADP), adenosine triphosphate (ATP), cyclic adenosine monophosphate (cAMP)). Measured in 10 mM HEPES at pH 7.0 and 295 K. Reproduced from reference 8.

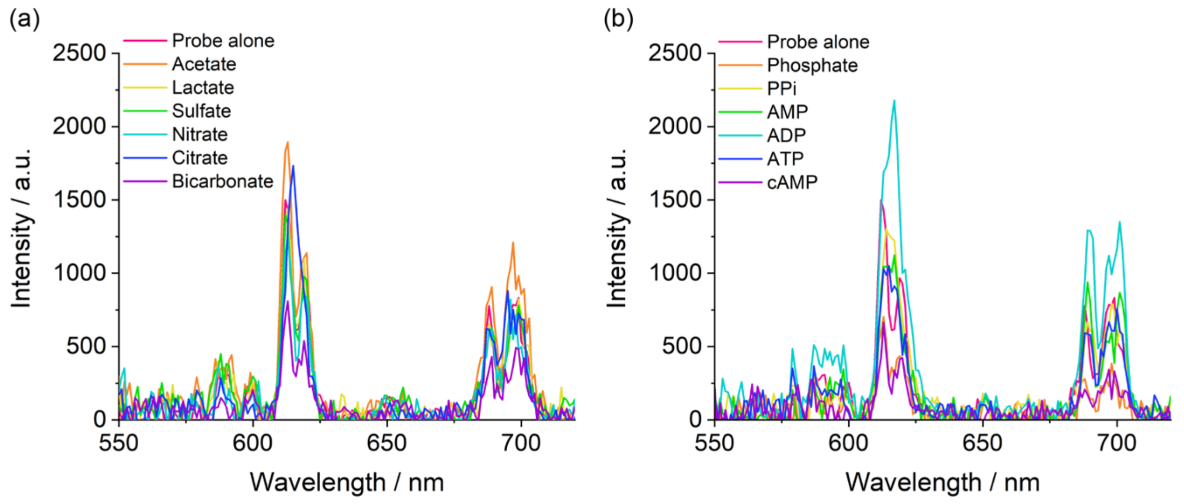


**Figure S17.** Selective emission enhancement of **[Eu.7PhOCH$_2$COO]**$^{-}$ (0.1 Abs, $\lambda_{exc}$ 321 nm) with (a) oxyanions (1 mM each: acetate, lactate, sulfate, nitrate, citrate, bicarbonate) and (b) phosphate containing anions (1 mM each: phosphate, pyrophosphate (PPi), adenosine monophosphate (AMP), adenosine diphosphate (ADP), adenosine triphosphate (ATP), cyclic adenosine monophosphate (cAMP)). Measured in 10 mM HEPES at pH 7.0 and 295 K.

**Table S8.** Lifetime values for Eu(**III**) complexes alone and in the presence of ADP (1 mM), measured in 10 mM HEPES at pH 7.0.

| Complex | Anion | $\tau_{H2O}$ / ms | $\tau_{D2O}$ / ms | q |
|---|---|---|---|---|
| **[Eu.4PhOCH$_2$COO]**$^{-}$ | None | 0.47 ± 0.01 | 1.00 ± 0.02 | 1.1 |
| | ADP | 1.02 ± 0.01 | 1.47 ± 0.01 | 0.1 |
| **[Eu.ADPGlow]**$^{-}$ | None | 0.021 ± 0.001 | 0.036 ± 0.001 | - |
| | ADP | 0.94 ± 0.04 | 1.35 ± 0.04 | 0.1 |
| **[Eu.7PhOCH$_2$COO]**$^{-}$ | None | 0.016 ± 0.001 | 0.013 ± 0.001 | - |
| | ADP | 0.64 ± 0.02 | 1.01 ± 0.03 | 0.4 |

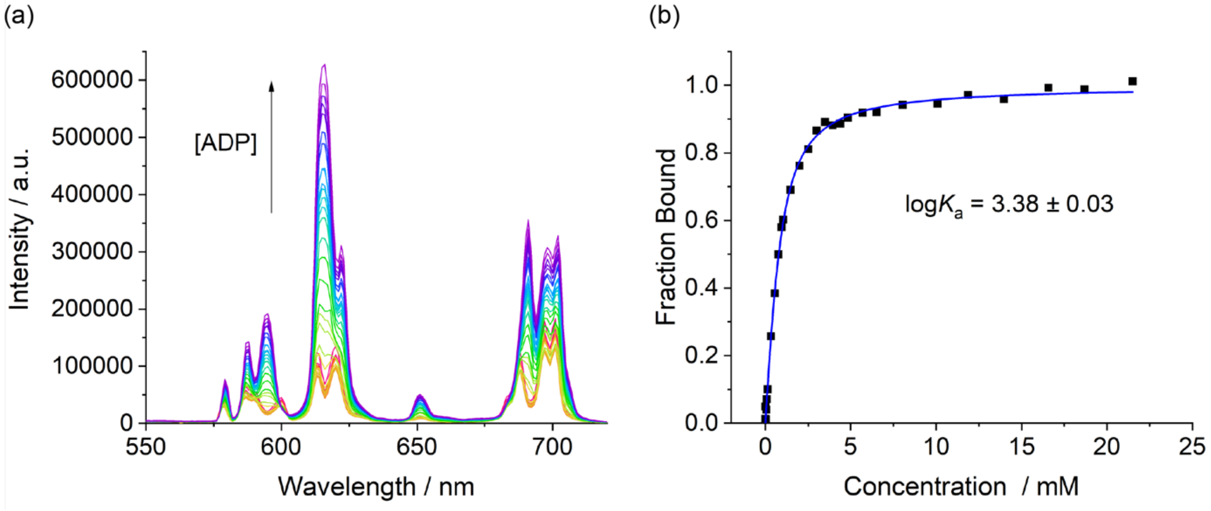


**Figure S18.** (a) Variation in emission spectra of [**Eu.4PhOCH$_2$COO**]$^-$ upon incremental addition of ADP; (d) Plot of fraction bound (determined from Δ$J$ = 2 / Δ$J$ = 1 intensity ratio) versus ADP concentration, showing the fit to a 1:1 binding isotherm. Measured in 10 mM HEPES at pH 7.0 and 295 K, $\lambda_{exc}$ = 321 nm.

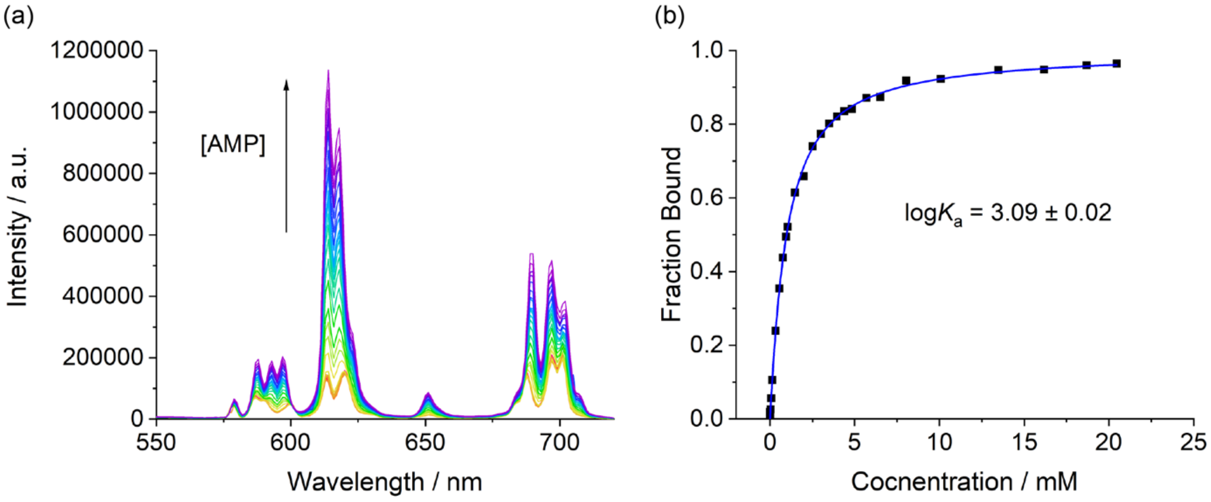


**Figure S19.** (a) Variation in emission spectra of [**Eu.4PhOCH$_2$COO**]$^-$ upon incremental addition of AMP; (d) Plot of fraction bound (determined from Δ$J$ = 2 / Δ$J$ = 1 intensity ratio) versus AMP concentration, showing the fit to a 1:1 binding isotherm. Measured in 10 mM HEPES at pH 7.0 and 295 K, $\lambda_{exc}$ = 321 nm.

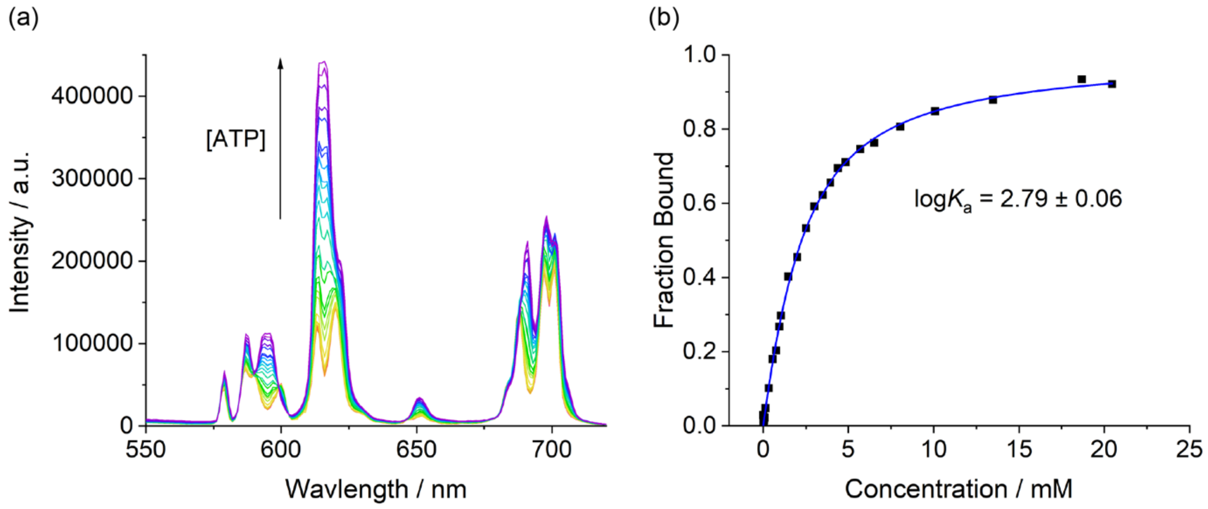


**Figure S20.** (a) Variation in emission spectra of [**Eu.4PhOCH$_2$COO**]$^-$ upon incremental addition of ATP; (d) Plot of fraction bound (determined from Δ$J$ = 2 / Δ$J$ = 1 intensity ratio) versus ATP concentration, showing the fit to a 1:1 binding isotherm. Measured in 10 mM HEPES at pH 7.0 and 295 K, $\lambda_{exc}$ = 321 nm.

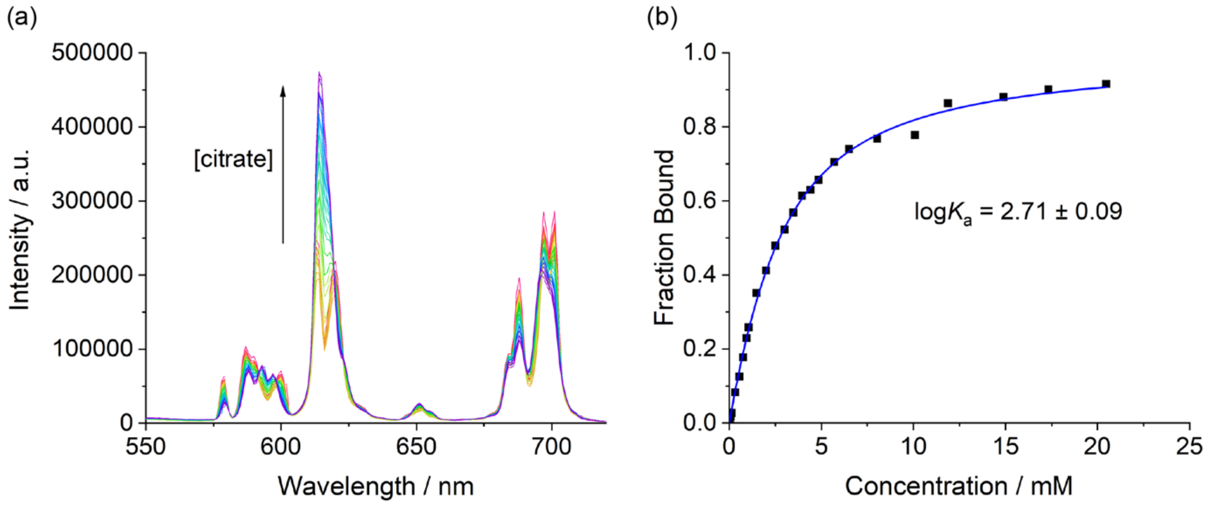


**Figure S21.** (a) Variation in emission spectra of [**Eu.4PhOCH$_2$COO**]$^-$ upon incremental addition of citrate; (d) Plot of fraction bound (determined from Δ$J$ = 2 / Δ$J$ = 1 intensity ratio) versus citrate concentration, showing the fit to a 1:1 binding isotherm. Measured in 10 mM HEPES at pH 7.0 and 295 K, $\lambda_{exc}$ = 321 nm.

## 6. References


1 A. Beeby, I. M. Clarkson, R. S. Dickins, S. Faulkner, D. Parker, L. Royle, A. S. De Sousa, J. A. G. Williams and M. Woods, *J. Chem. Soc., Perkin Trans. 2*, 1999, **2**, 493–503.

2 R. D. Peacock, *Struct. Bonding (Berlin)*, 1975, **22**, 83–122.

3 A. Beeby, L. M. Bushby, D. Maffeo and J. A. Gareth Williams, *J. Chem. Soc. Dalton Trans.*, 2002, **2**, 48–54.

4 G. M. Sheldrick, *Acta Crystallogr. A*, 2015, **71**, 3–8.

5 G. M. Sheldrick, *Acta Crystallogr. C*, 2015, **71**, 3–8.

6 O. V. Dolomanov, L. J. Bourhis, R. J. Gildea, J. A. K. Howard and H. Puschmann, *J. Appl. Crystallogr.*, 2009, **42**, 339–341.

7 S. J. Butler, *Chem. Eur. J.*, 2014, **20**, 15768–15774.

8 S. E. Bodman, P. Stachelek, U. Rehman, F. Plasser, R. Pal and S. J. Butler, *Chem. Sci.*, 2025, **16**, 5602–5612.

## Appendix 1: $^{1}H$ NMR and $^{13}C$ NMR spectra

4-(4-Methoxyphenyl)-2-methylquinoline (**1a**)

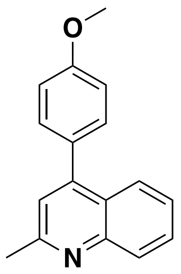


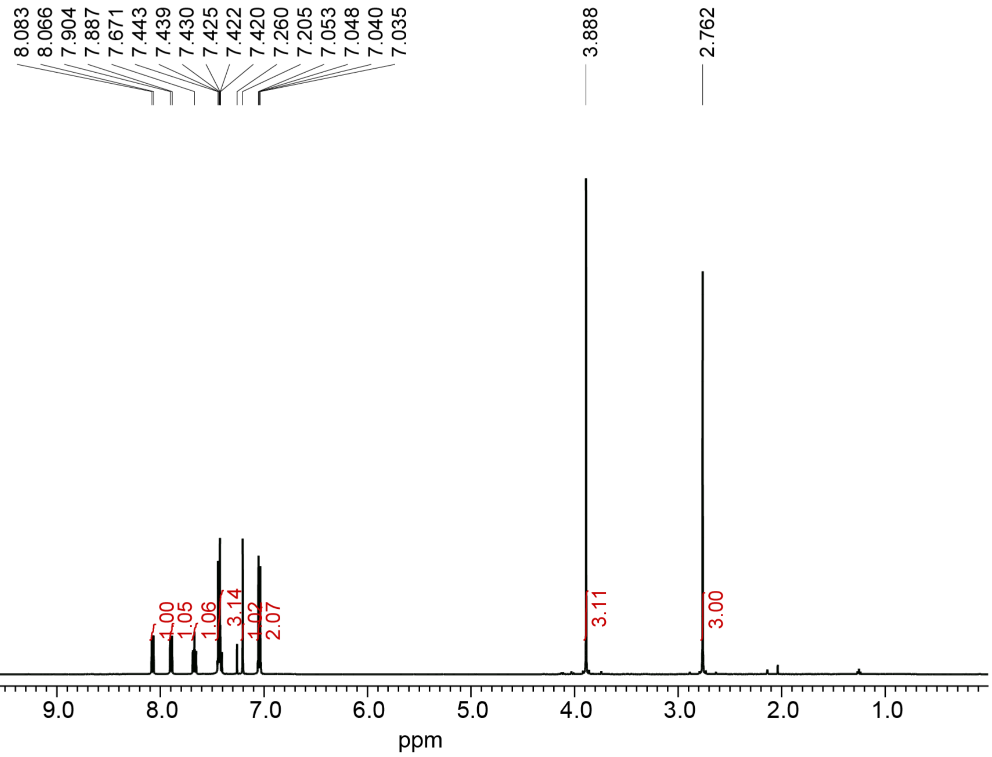


$^{1}H$ NMR (500 MHz, $CDCl_3$, 298 K) of 4-(4-methoxyphenyl)-2-methylquinoline (**1a**).

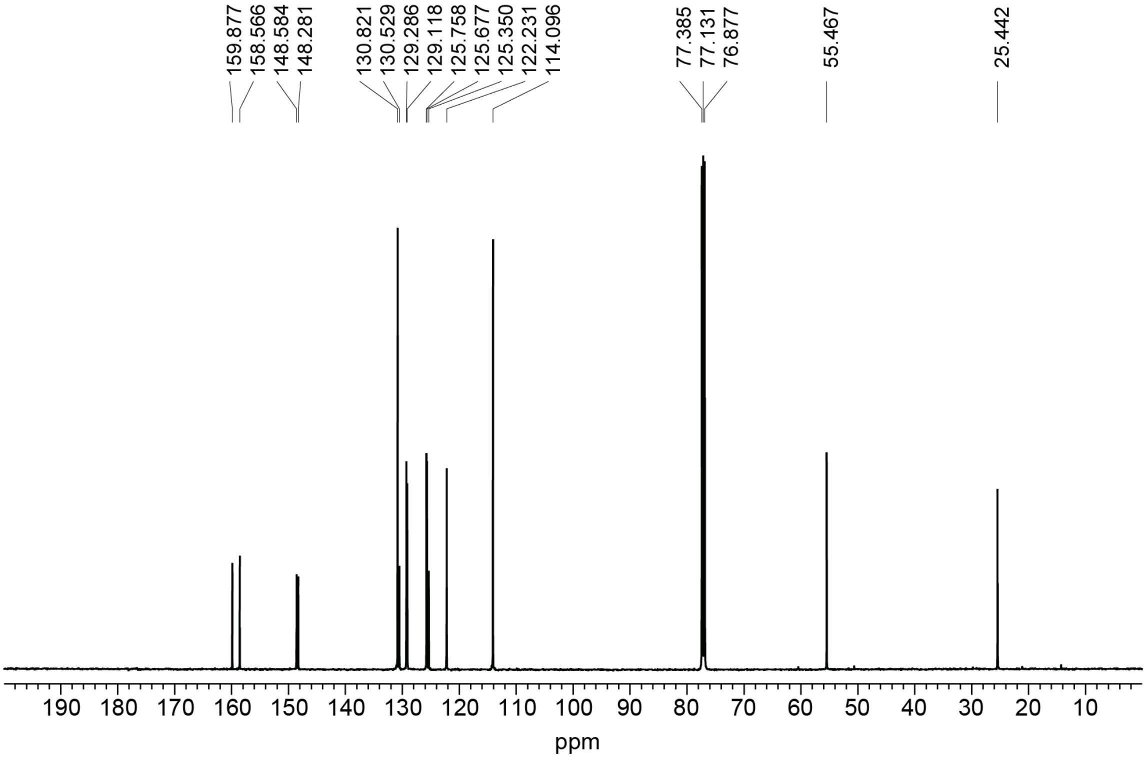


$^{13}C$ NMR (126 MHz, $CDCl_3$, 298 K) of 4-(4-methoxyphenyl)-2-methylquinoline (**1a**).

4-(4-Methoxyphenyl)-2-quinolinecarboxaldehyde (**2a**)

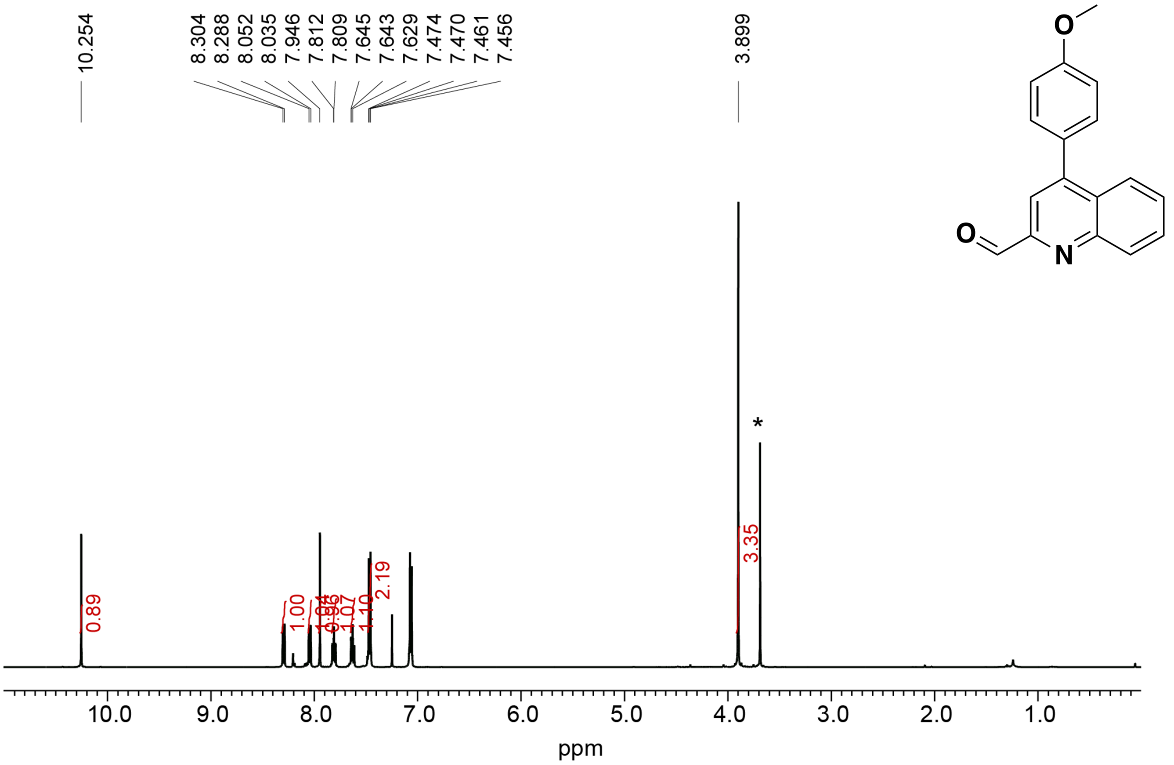


$^{1}$H NMR (500 MHz, $CDCl_3$, 298 K) of 4-(4-methoxyphenyl)-2-quinolinecarboxaldehyde (**2a**).

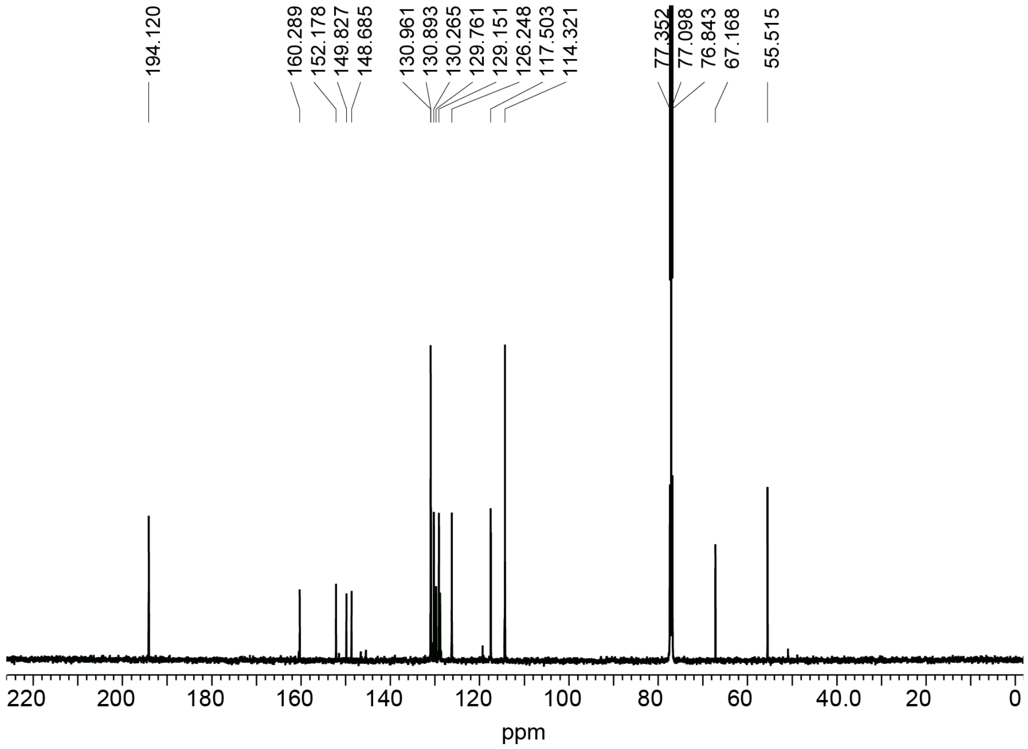


$^{13}$C NMR (126 MHz, $CDCl_3$, 298 K) of 4-(4-methoxyphenyl)-2-quinolinecarboxaldehyde (**2a**).

4-(4-Methoxyphenyl)-2-quinolinemethanol (**3a**)

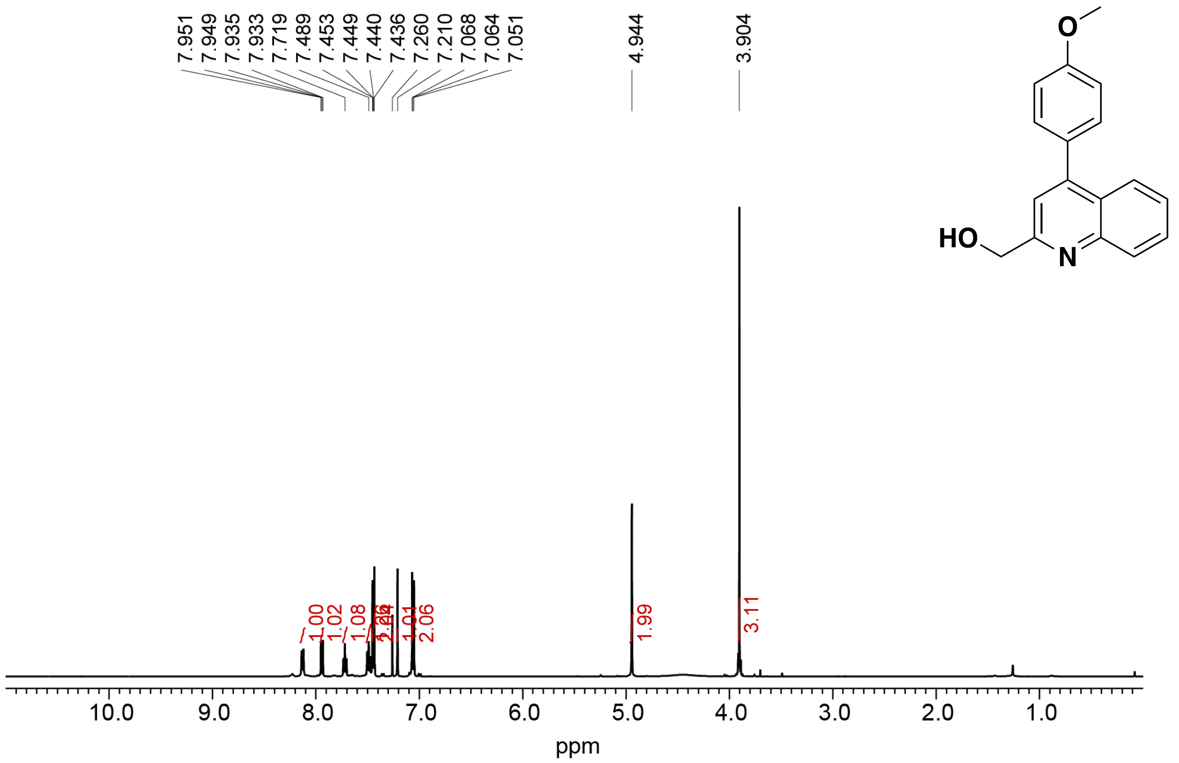


$^{1}$H NMR (500 MHz, $CDCl_3$, 298 K) of 4-(4-methoxyphenyl)-2-quinolinemethanol (**3a**).

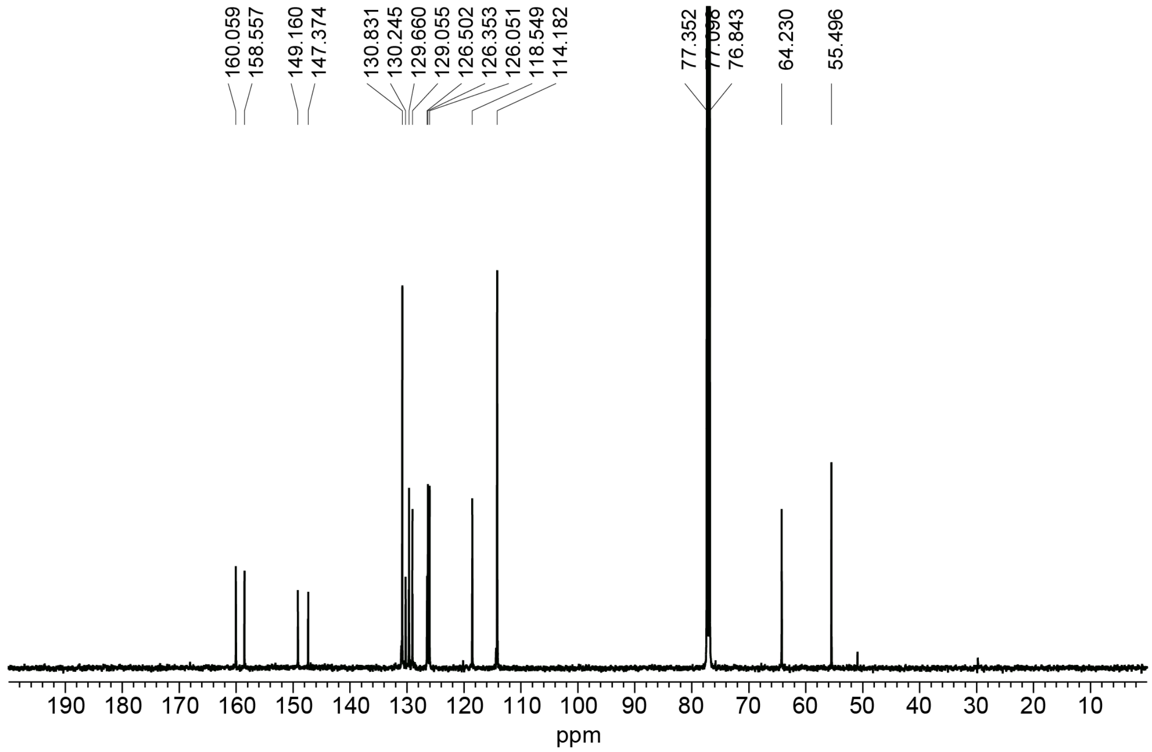


$^{13}$C NMR (126 MHz, $CDCl_3$, 298 K) of 4-(4-methoxyphenyl)-2-quinolinemethanol (**3a**).

4-(4-Methoxyphenyl)- 2-methanesulfonate-2-quinolinemethanol (**4a**)

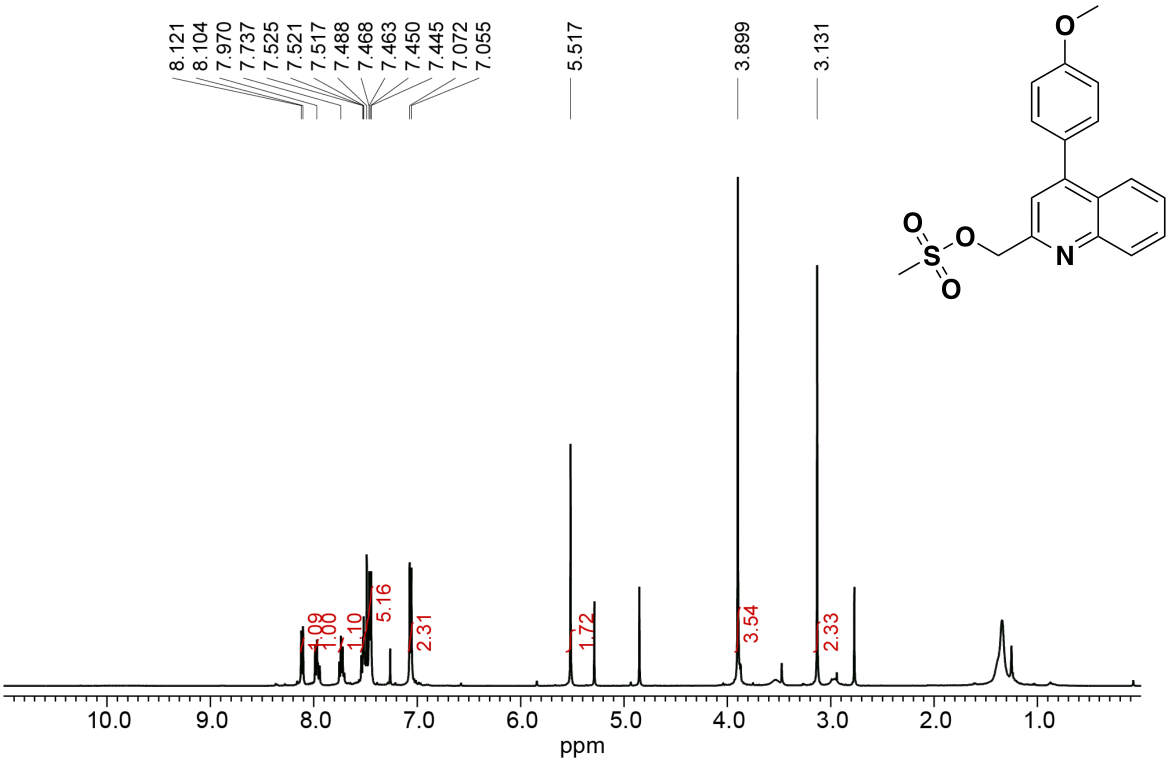


$^{1}$H NMR (500 MHz, $CDCl_3$, 298 K) of 4-(4-methoxyphenyl)- 2-methanesulfonate-2-quinolinemethanol (**4a**).

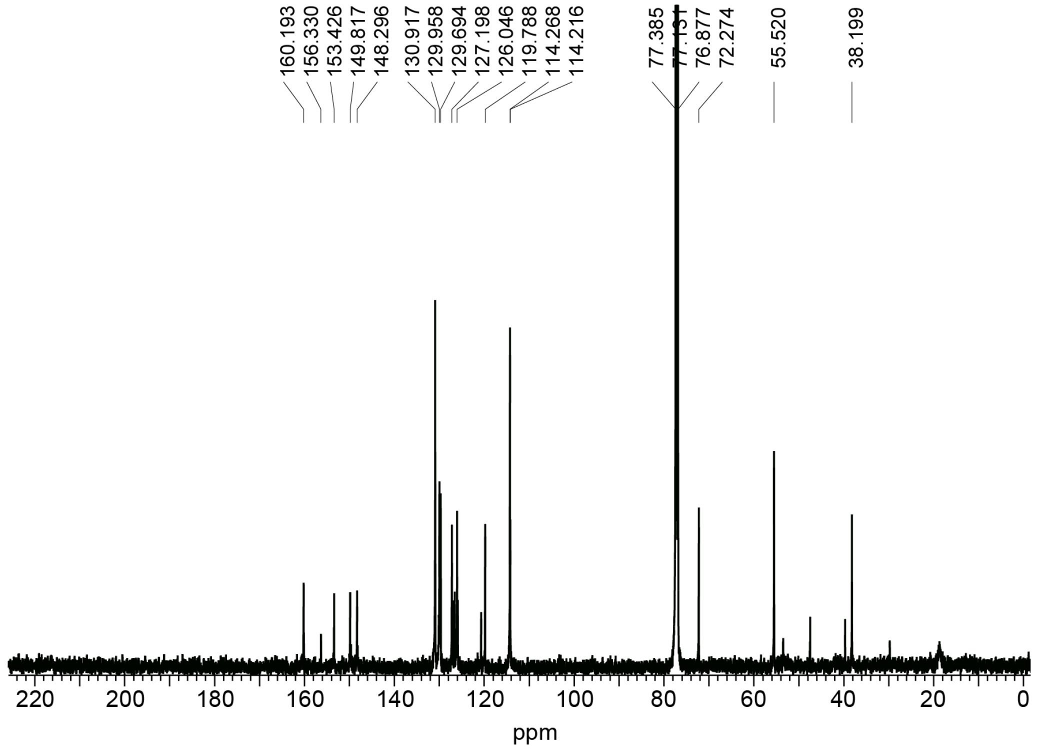


$^{13}$C NMR (126 MHz, $CDCl_3$, 298 K) of 4-(4-methoxyphenyl)- 2-methanesulfonate-2-quinolinemethanol (**4a**).

4,10-*Bis*((4-(4-methoxyphenyl)-quinolin-2-yl)-methyl)-1,4,7,10-tetraazacyclododecane-1,7-diyl)-diacetate (**5a**)

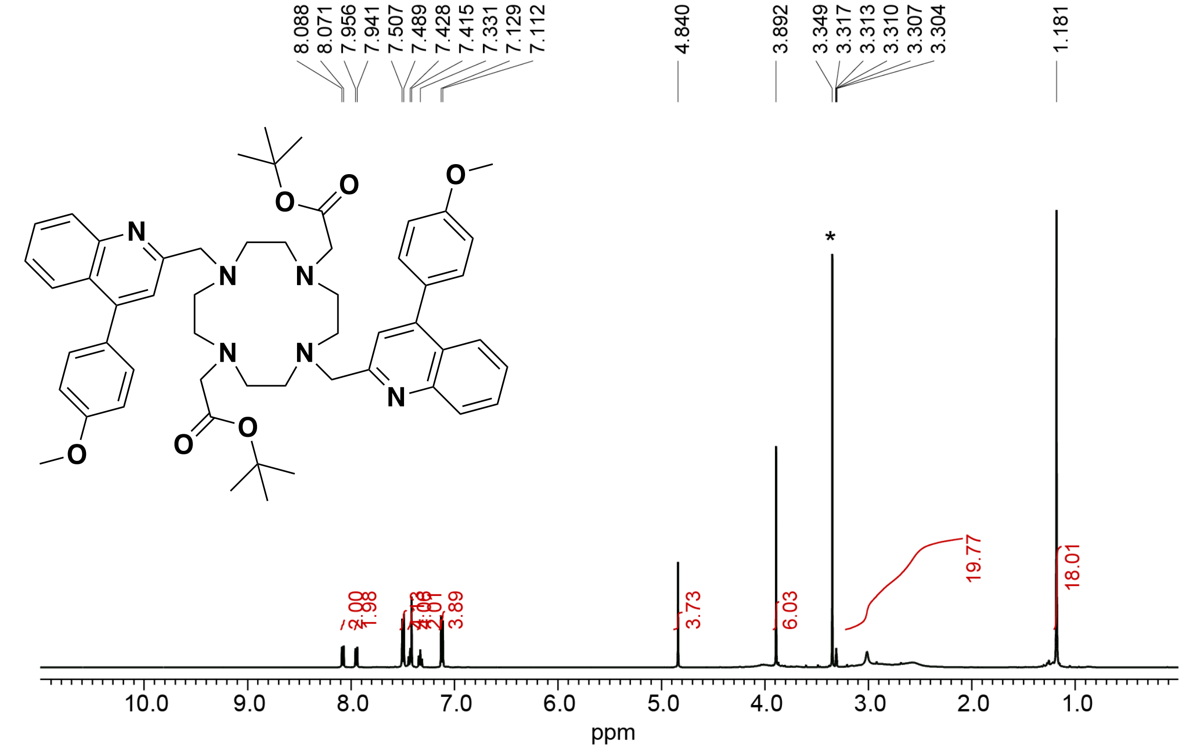


$^{1}$H NMR (500 MHz, $CD_3OD$, 298 K) of 4,10-*bis*((4-(4-methoxyphenyl)-quinolin-2-yl)-methyl)-1,4,7,10-tetraazacyclododecane-1,7-diyl)-diacetate (**5a**).

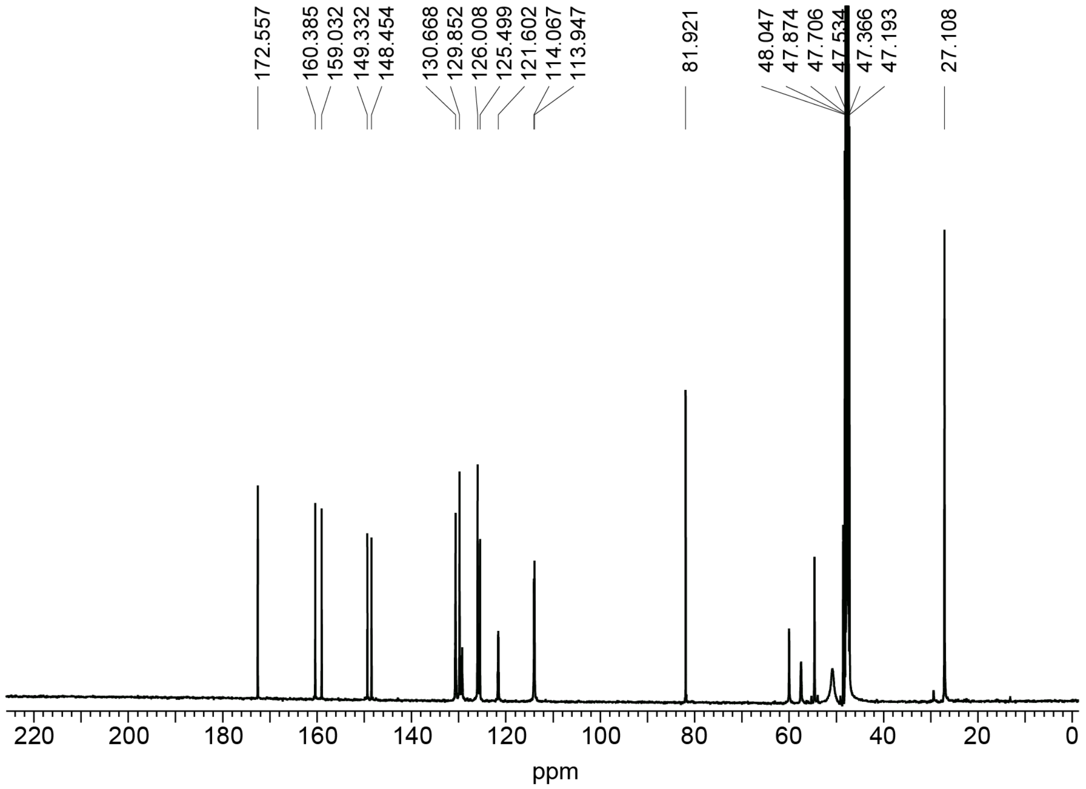


$^{13}$C NMR (126 MHz, $CD_3OD$, 298 K) of 4,10-*bis*((4-(4-methoxyphenyl)-quinolin-2-yl)-methyl)-1,4,7,10-tetraazacyclododecane-1,7-diyl)-diacetate (**5a**).

4,10-*Bis*((4-(4-methoxyphenyl)-quinolin-2-yl)-methyl)-1,4,7,10-tetraazacyclododecane-1,7-diyl)-diacetic acid (**6a**)

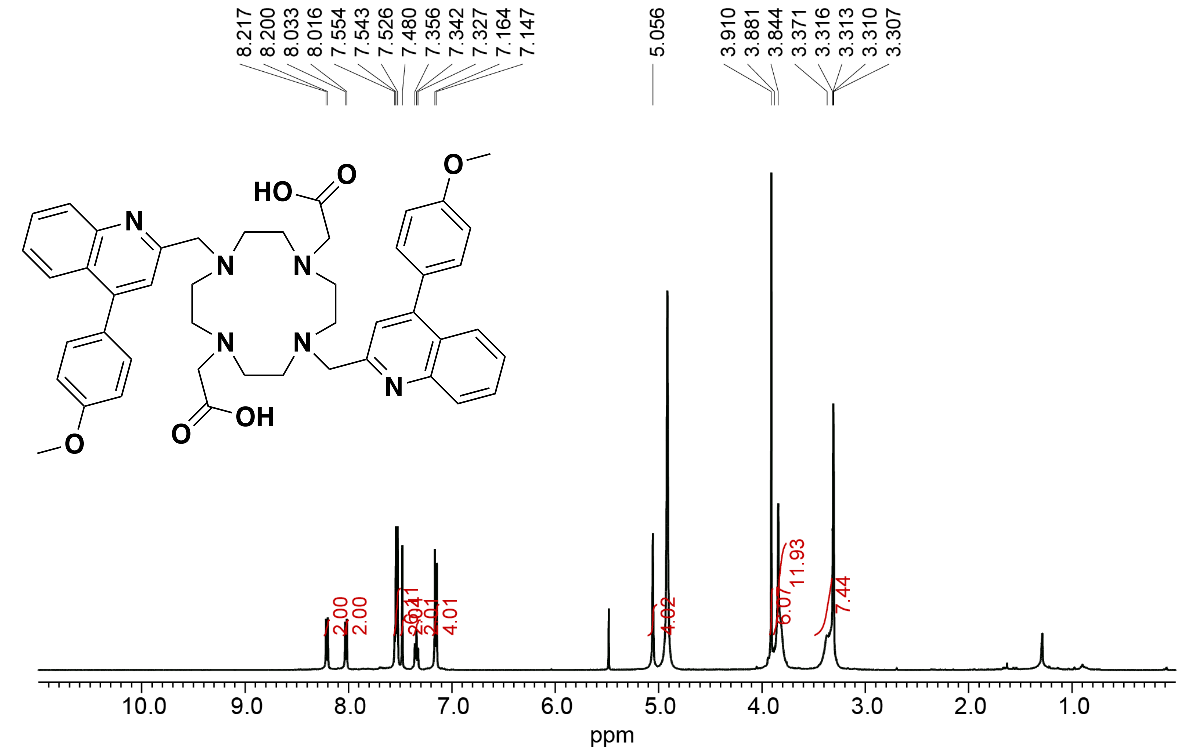


$^{1}$H NMR (500 MHz, $CD_3OD$, 298 K) of 4,10-*bis*((4-(4-methoxyphenyl)-quinolin-2-yl)-methyl)-1,4,7,10-tetraazacyclododecane-1,7-diyl)-diacetic acid (**6a**).

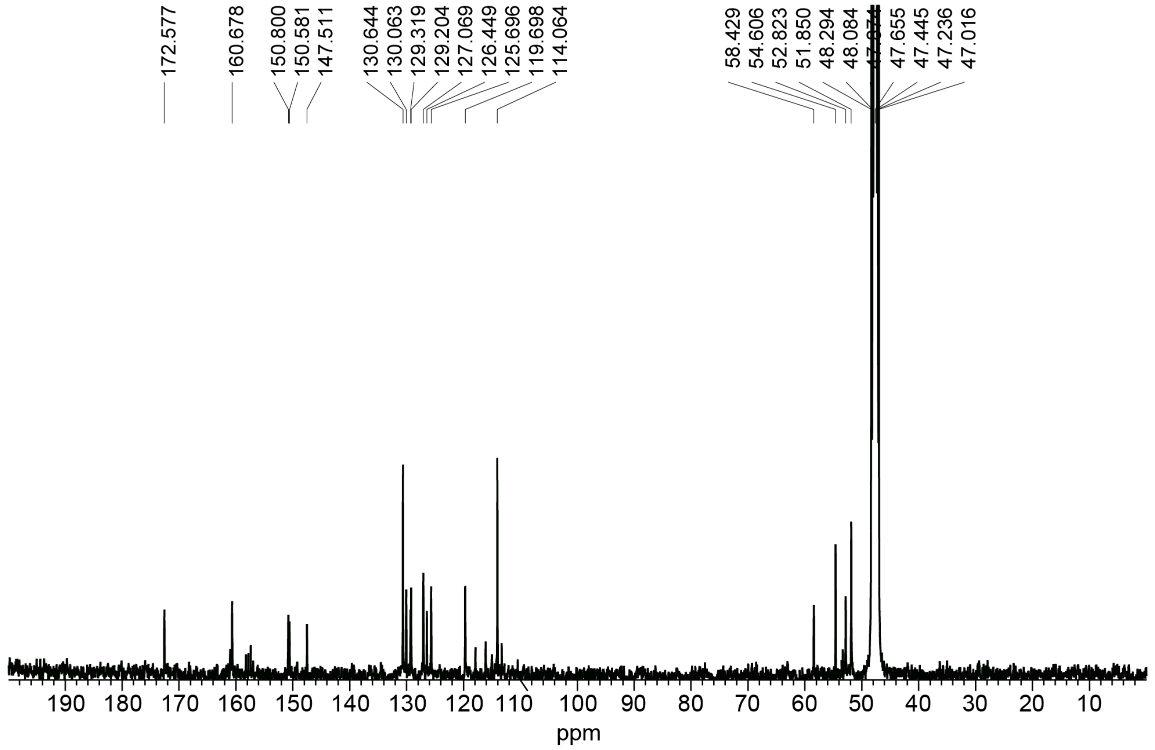


$^{13}$C NMR (126 MHz, $CD_3OD$, 298 K) of 4,10-*bis*((4-(4-methoxyphenyl)-quinolin-2-yl)-methyl)-1,4,7,10-tetraazacyclododecane-1,7-diyl)-diacetic acid (**6a**).

**[Eu.4PhOMe]$^+$**

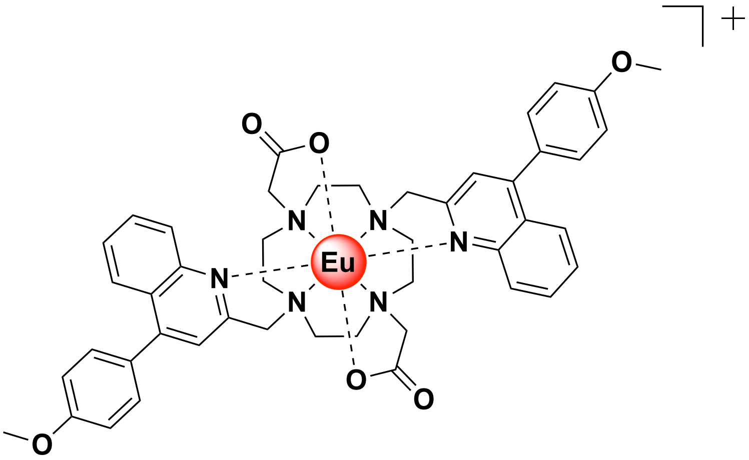


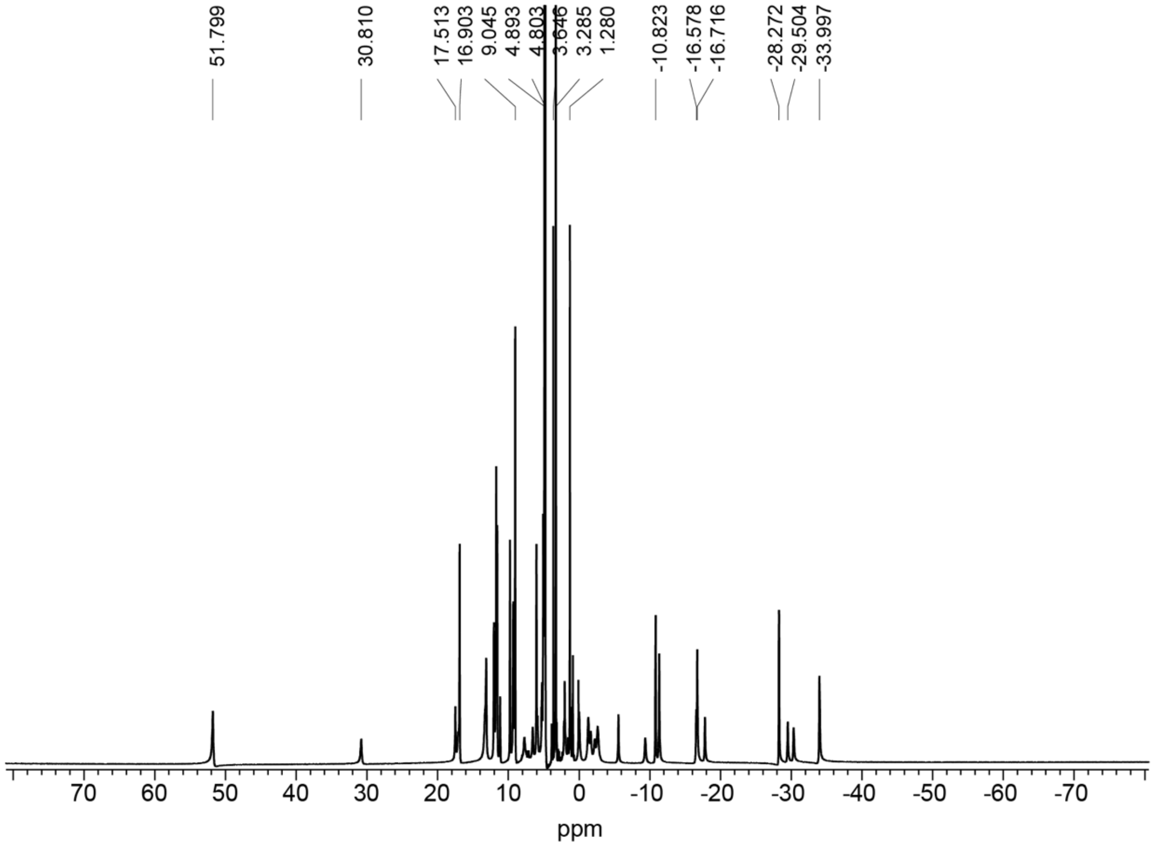


$^1$H NMR (500 MHz, $CD_3OD$, 298 K) of **[Eu.4PhOMe]$^+$**.

4-(2-Methyl-4-quinolinyl)-phenol (**1b**)

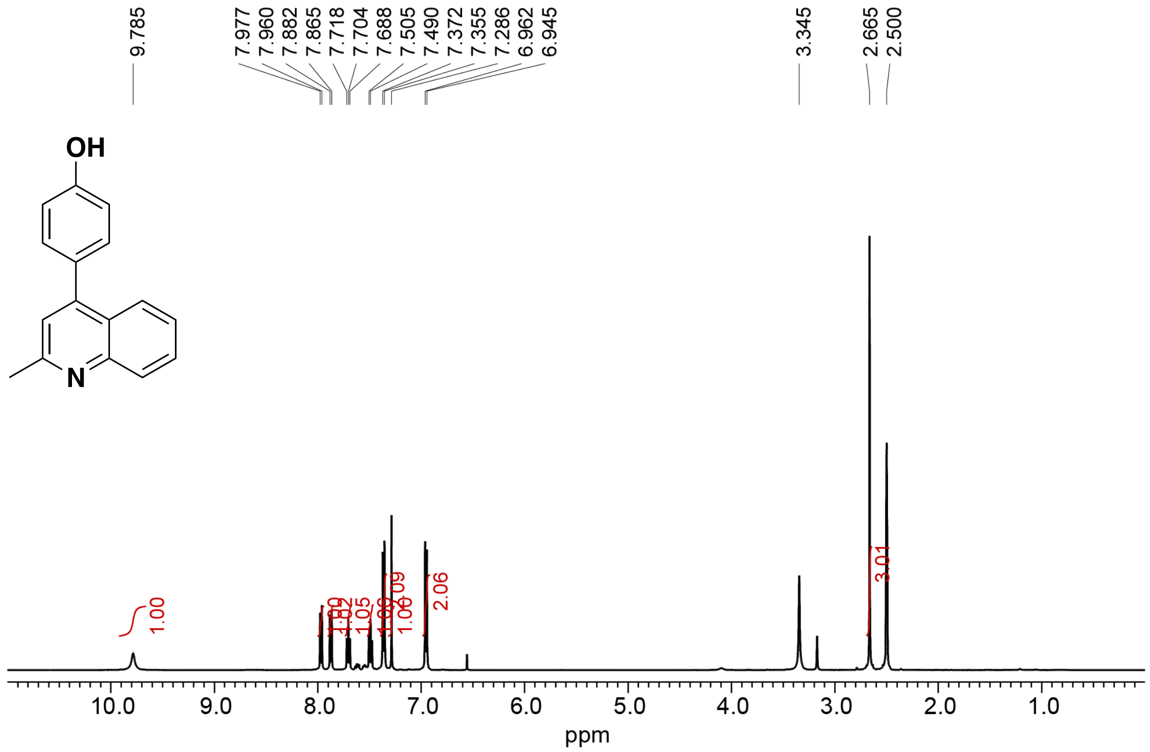


$^{1}H$ NMR (500 MHz, DMSO-$d_6$, 298 K) of 4-(2-methyl-4-quinolinyl)-phenol (**1b**).

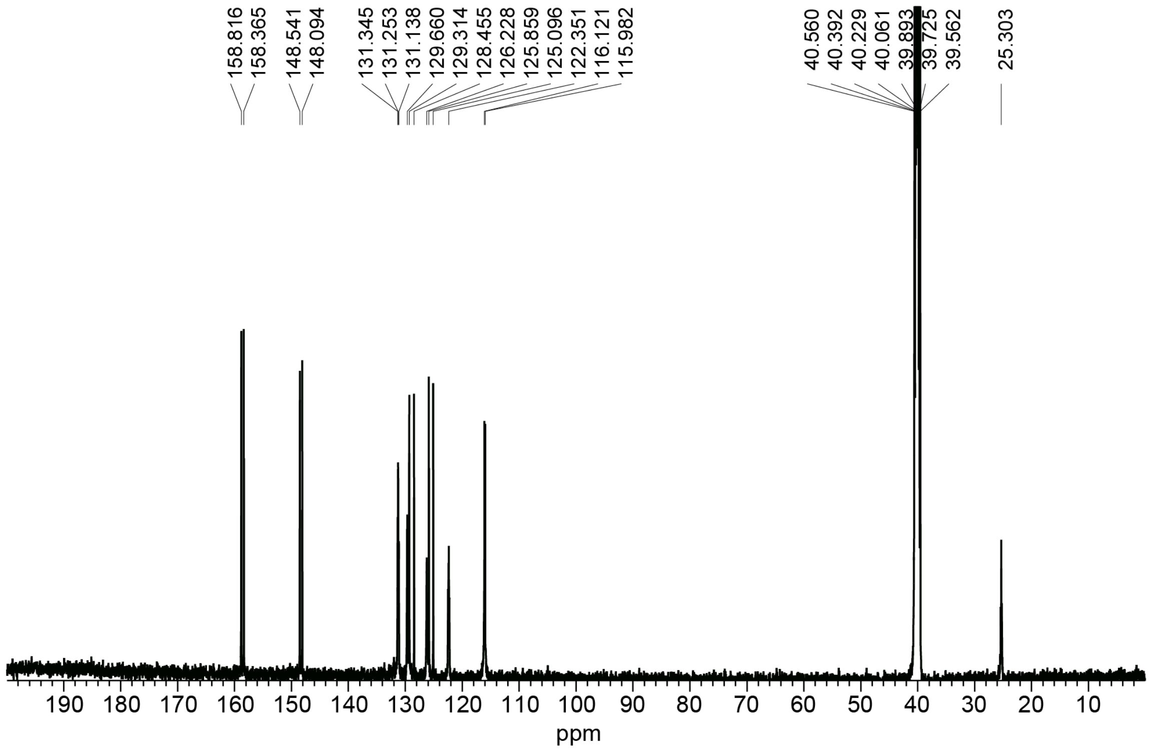


$^{13}C$ NMR (126 MHz, DMSO-$d_6$, 298 K) of 4-(2-methyl-4-quinolinyl)-phenol (**1b**).

4-(4-(Phenoxy)*tert*-butyl acetate)-2-methylquinoline or 4-(4-(1,1-dimethylethyl)-phenoxyacetate)-2-methylquinoline (**1c**)

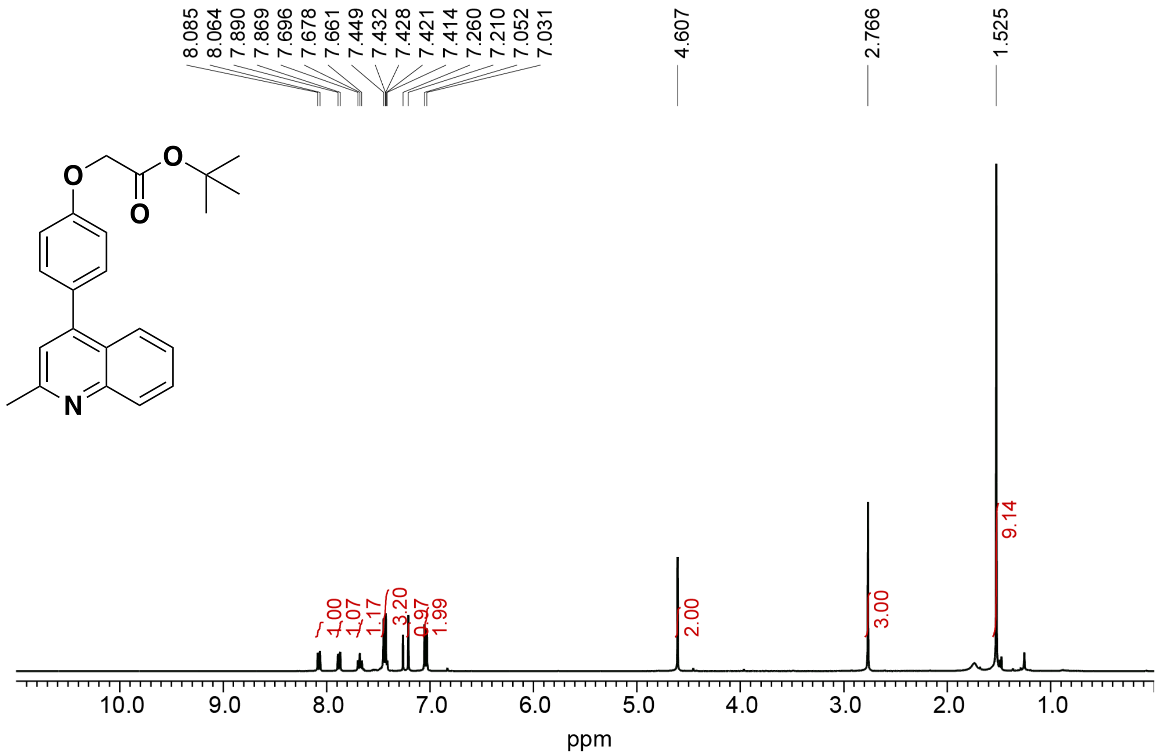


$^{1}$H NMR (500 MHz, $CDCl_3$, 298 K) of 4-(4-(phenoxy)*tert*-butyl acetate)-2-methylquinoline or 4-(4-(1,1-dimethylethyl)-phenoxyacetate)-2-methylquinoline (**1c**).

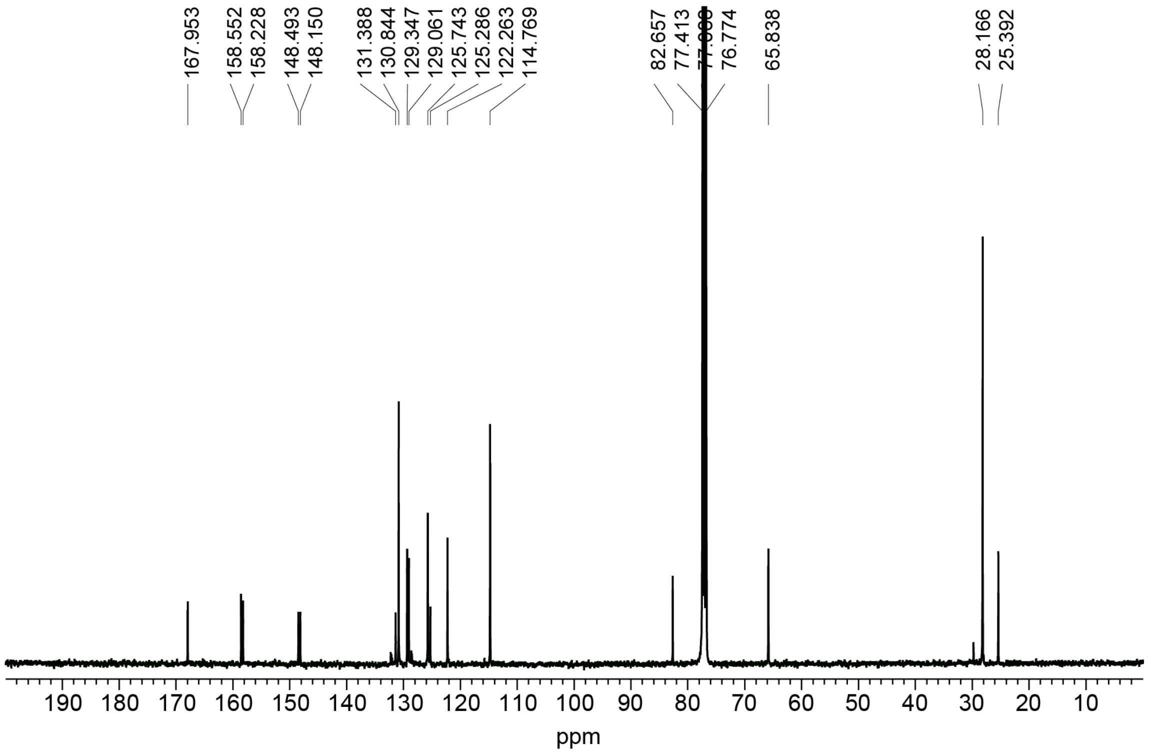


$^{13}$C NMR (126 MHz, $CDCl_3$, 298 K) of 4-(4-(phenoxy)*tert*-butyl acetate)-2-methylquinoline or 4-(4-(1,1-dimethylethyl)-phenoxyacetate)-2-methylquinoline (**1c**).

4-(4-(1,1-Dimethylethyl)-phenoxyacetate)-2-quinolinecarboxaldehyde (**2b**)

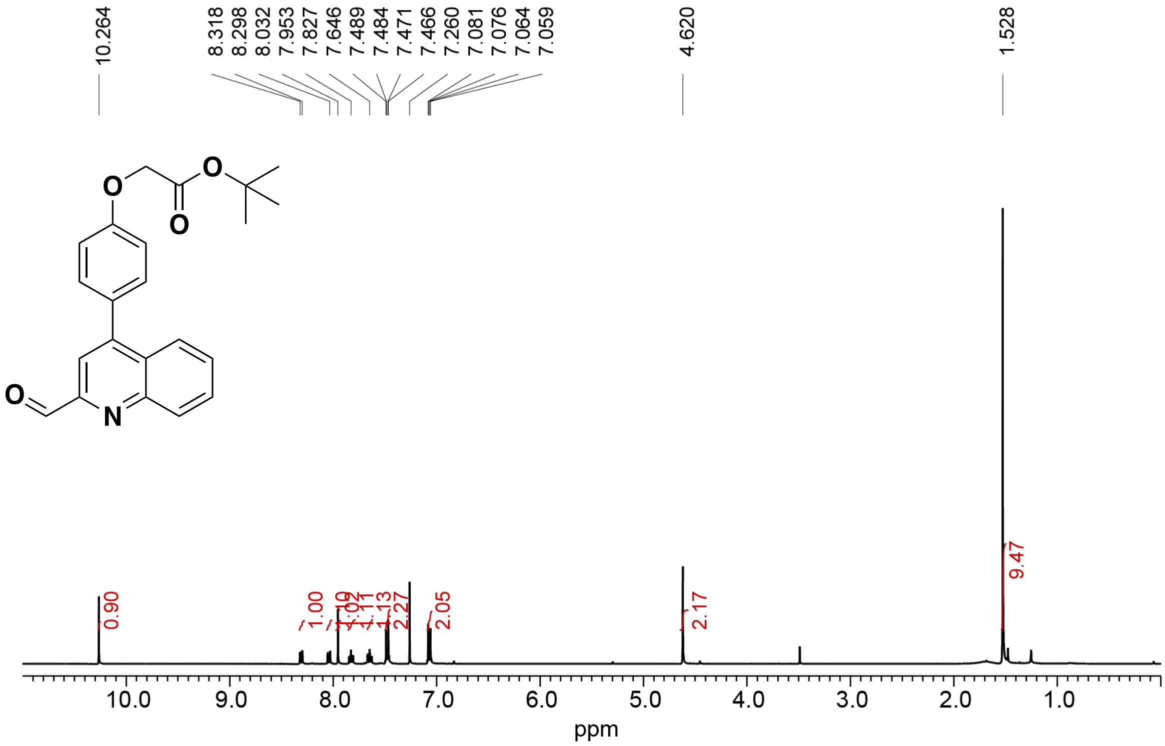


$^{1}$H NMR (500 MHz, $CDCl_3$, 298 K) of 4-(4-(1,1-dimethylethyl)-phenoxyacetate)-2-quinolinecarboxaldehyde (**2b**).

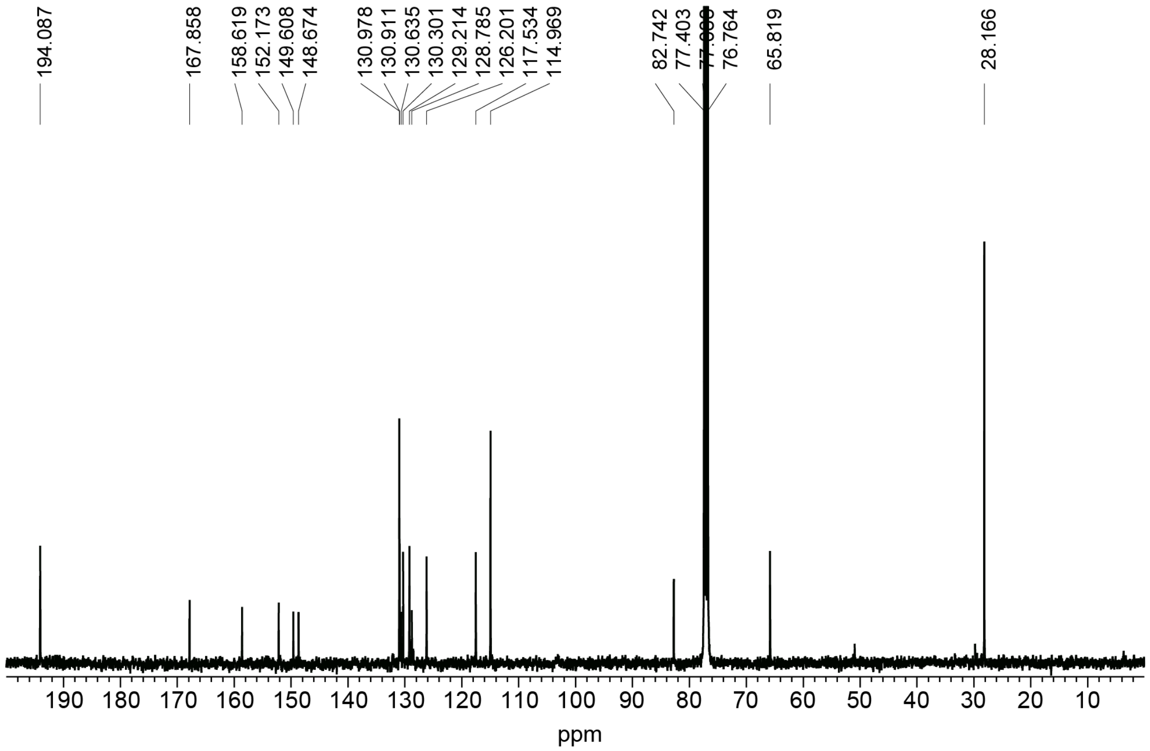


$^{13}$C NMR (126 MHz, $CDCl_3$, 298 K) of 4-(4-(1,1-dimethylethyl)-phenoxyacetate)-2-quinolinecarboxaldehyde (**2b**).

4-(4-(1,1-Dimethylethyl)-phenoxyacetate)-2-quinolinemethanol (**3b**)

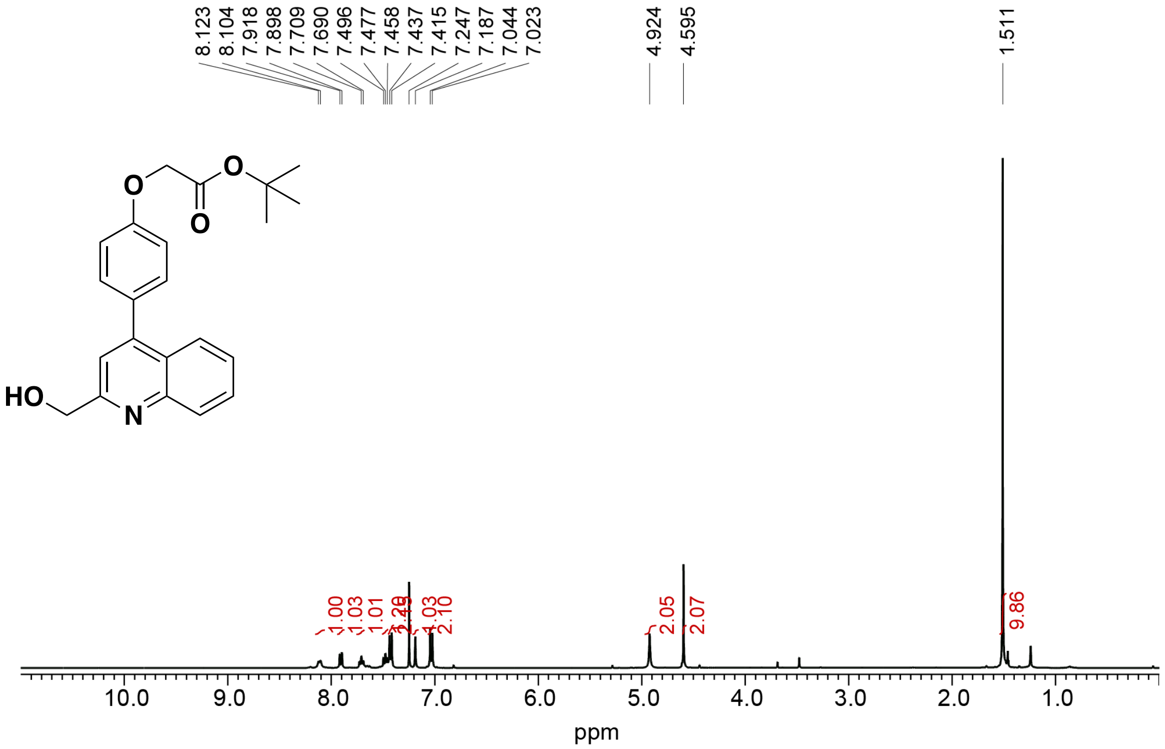


$^{1}$H NMR (500 MHz, $CDCl_3$, 298 K) of 4-(4-(1,1-dimethylethyl)-phenoxyacetate)-2-quinolinemethanol (**3b**).

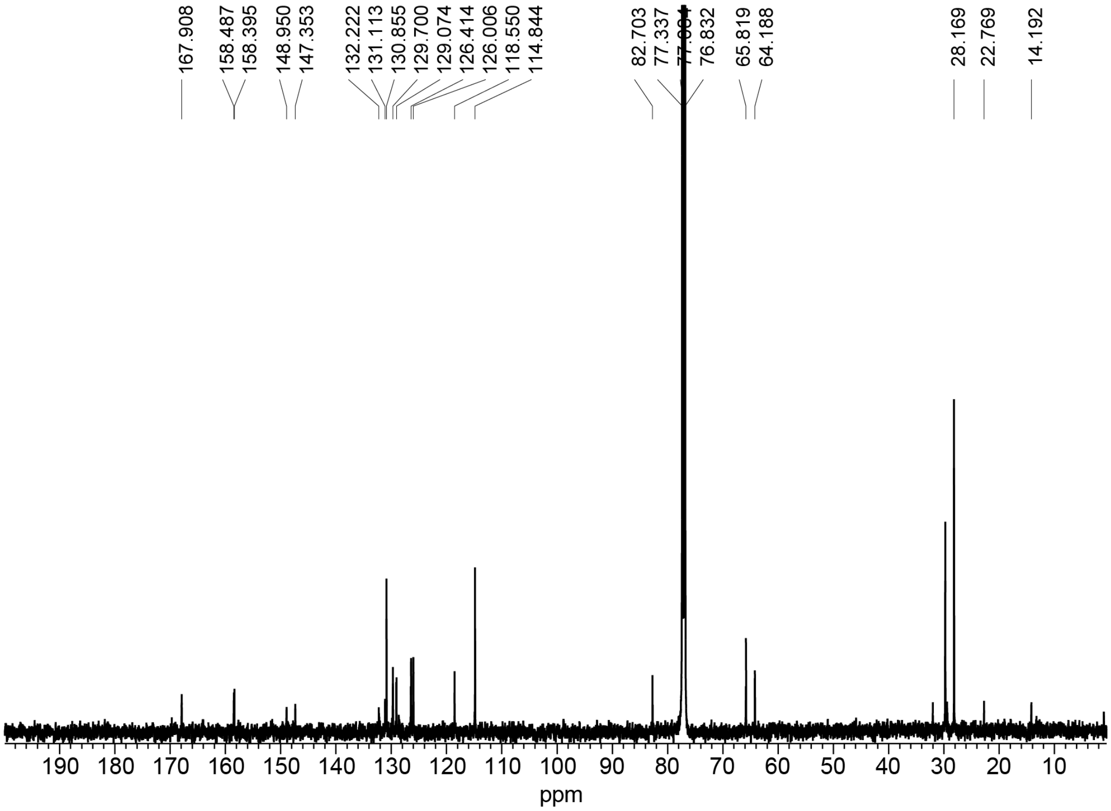


$^{13}$C NMR (126 MHz, $CDCl_3$, 298 K) of 4-(4-(1,1-dimethylethyl)-phenoxyacetate)-2-quinolinemethanol (**3b**).

4-(4-(1,1-Dimethylethyl)-phenoxyacetate)- 2-methanesulfonate-2-quinolinemethanol (**4b**)

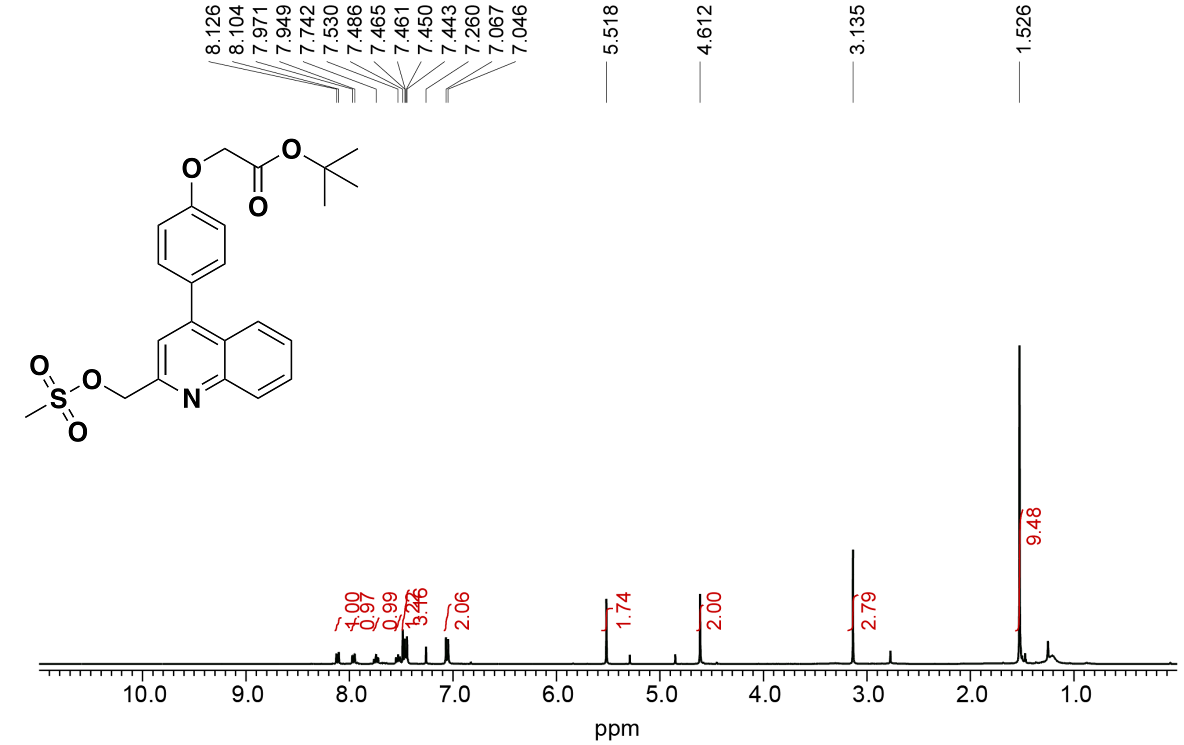


$^{1}$H NMR (500 MHz, $CDCl_3$, 298 K) of 4-(4-(1,1-dimethylethyl)-phenoxyacetate)- 2-methanesulfonate-2-quinolinemethanol (**4b**).

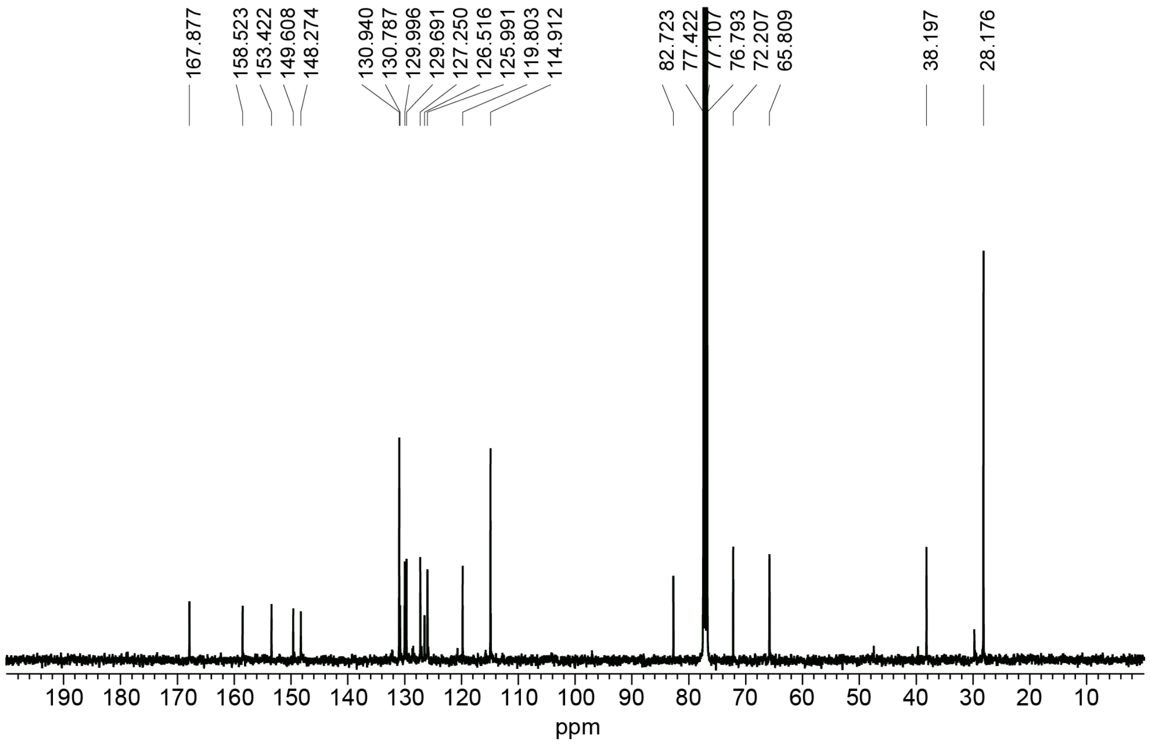


$^{13}$C NMR (126 MHz, $CDCl_3$, 298 K) of 4-(4-(1,1-dimethylethyl)-phenoxyacetate)- 2-methanesulfonate-2-quinolinemethanol (**4b**).

4,10-*Bis*((4-(4-(1,1-dimethylethyl)-phenoxyacetate)-quinolin-2-yl)-methyl)-1,4,7,10-tetraazacyclododecane-1,7-diyl)-diacetate (**5b**)

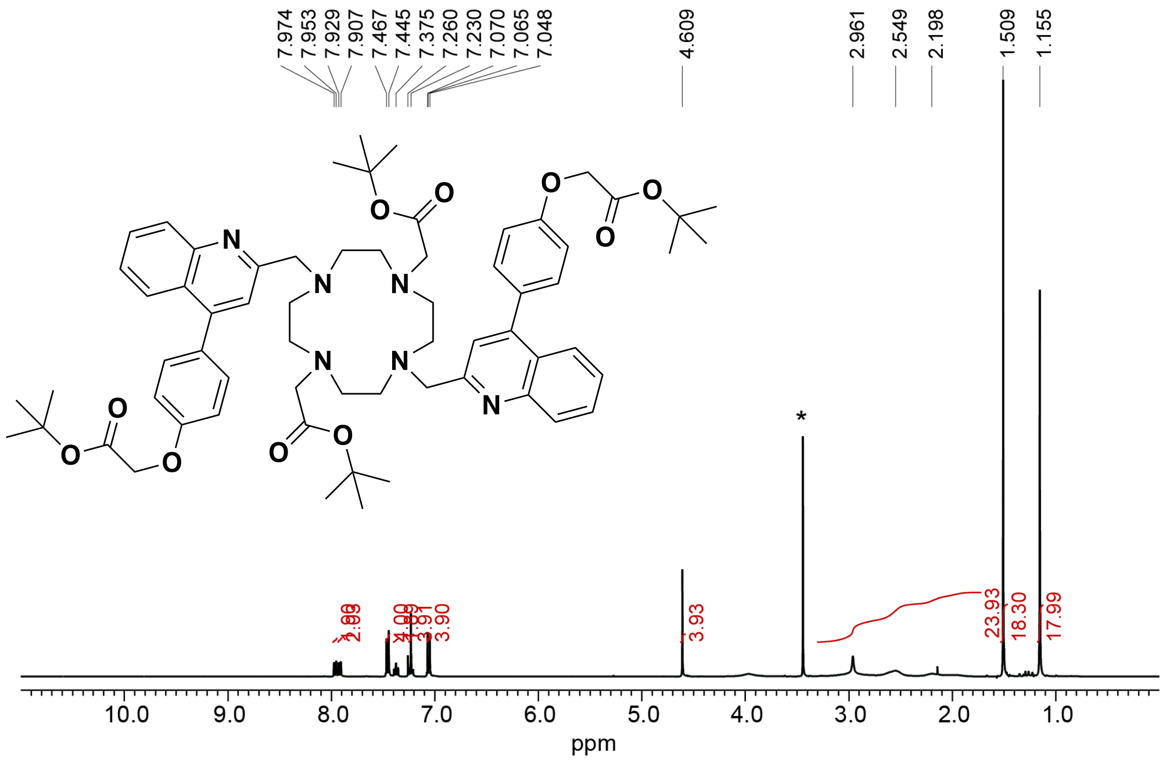

$^{1}$H NMR (400 MHz, $CDCl_3$, 298 K) of 4,10-*bis*((4-(4-(1,1-dimethylethyl)-phenoxyacetate)-quinolin-2-yl)-methyl)-1,4,7,10-tetraazacyclododecane-1,7-diyl)-diacetate (**5b**).

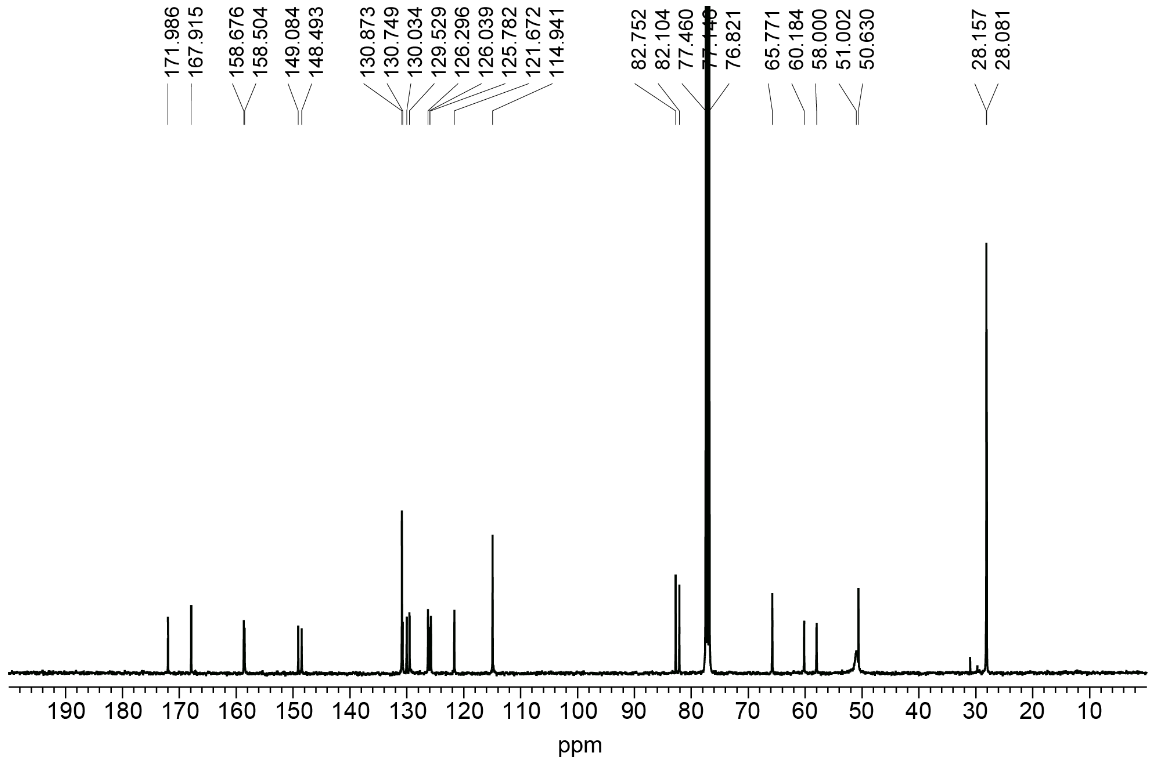

$^{13}$C NMR (101 MHz, $CDCl_3$, 298 K) of 4,10-*bis*((4-(4-(1,1-dimethylethyl)-phenoxyacetate)-quinolin-2-yl)-methyl)-1,4,7,10-tetraazacyclododecane-1,7-diyl)-diacetate (**5b**).

4,10-*Bis*((4-(4-(1,1-dimethylethyl)-phenoxyacetate)-quinolin-2-yl)-methyl)-1,4,7,10-tetraazacyclododecane-1,7-diyl)-diacetic acid (**6b**)

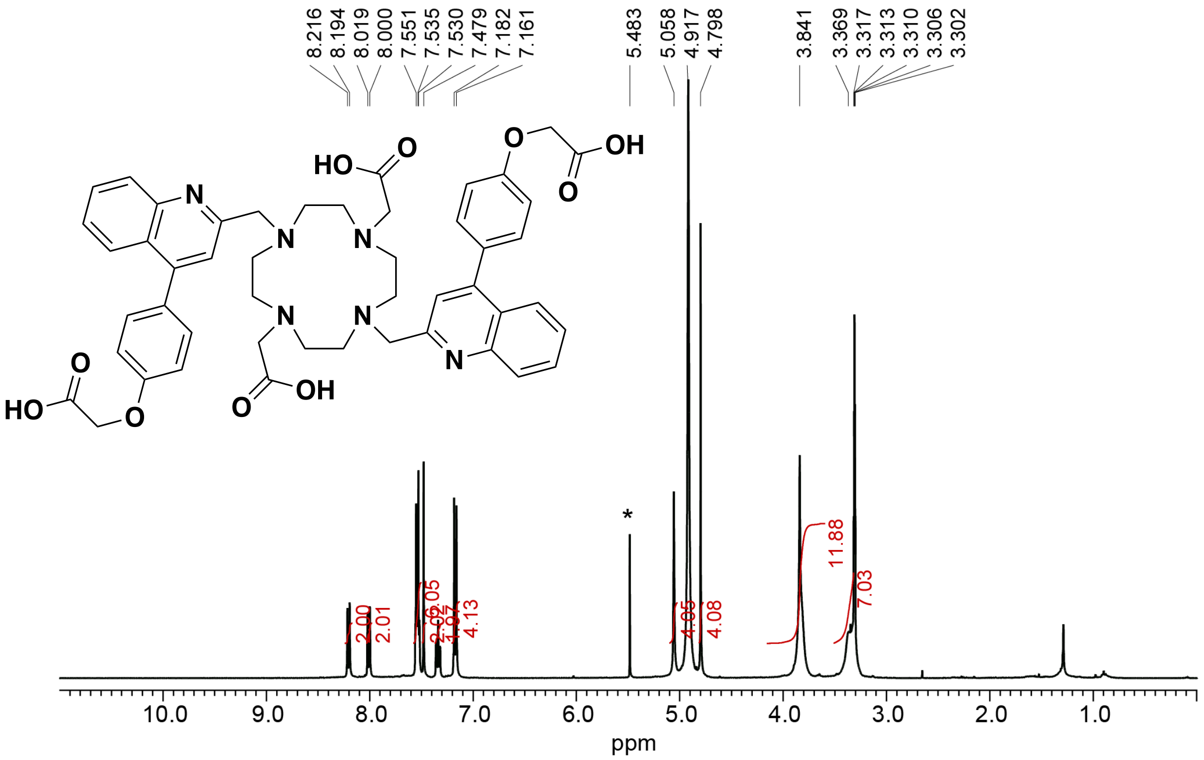


$^{1}$H NMR (500 MHz, $CDCl_3$, 298 K) of 4,10-*bis*((4-(4-(1,1-dimethylethyl)-phenoxyacetate)-quinolin-2-yl)-methyl)-1,4,7,10-tetraazacyclododecane-1,7-diyl)-diacetic acid (**6b**).

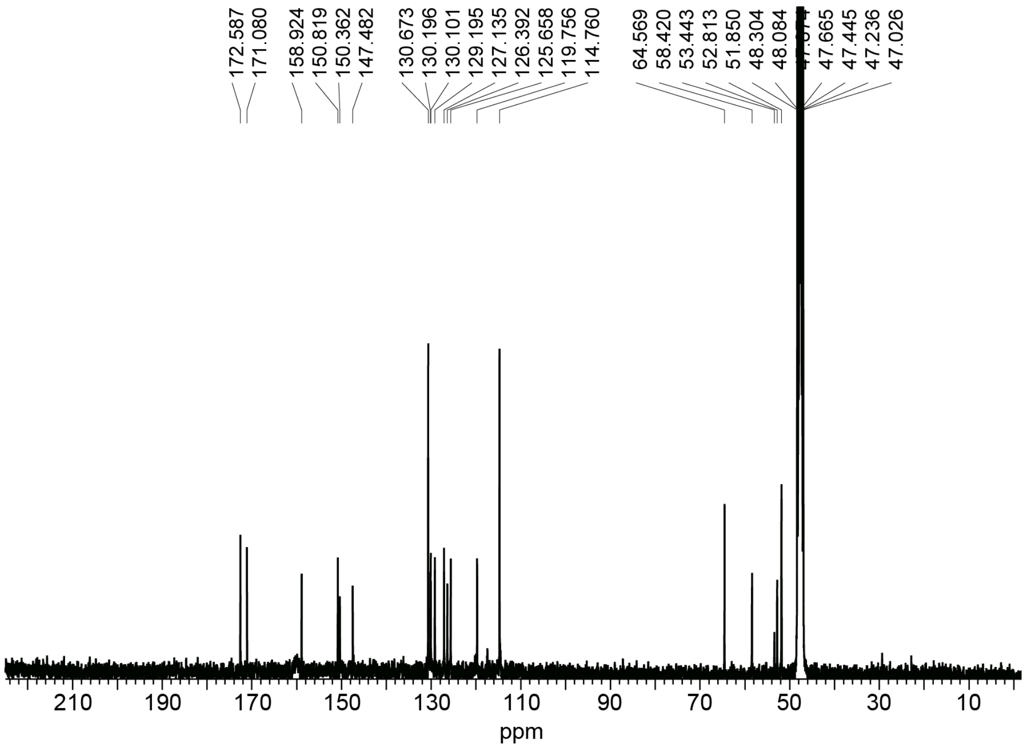


$^{13}$C NMR (126 MHz, $CDCl_3$, 298 K) of 4,10-*bis*((4-(4-(1,1-dimethylethyl)-phenoxyacetate)-quinolin-2-yl)-methyl)-1,4,7,10-tetraazacyclododecane-1,7-diyl)-diacetic acid (**6b**).

**[Eu.4PhOCH$_2$COO]$^-$**

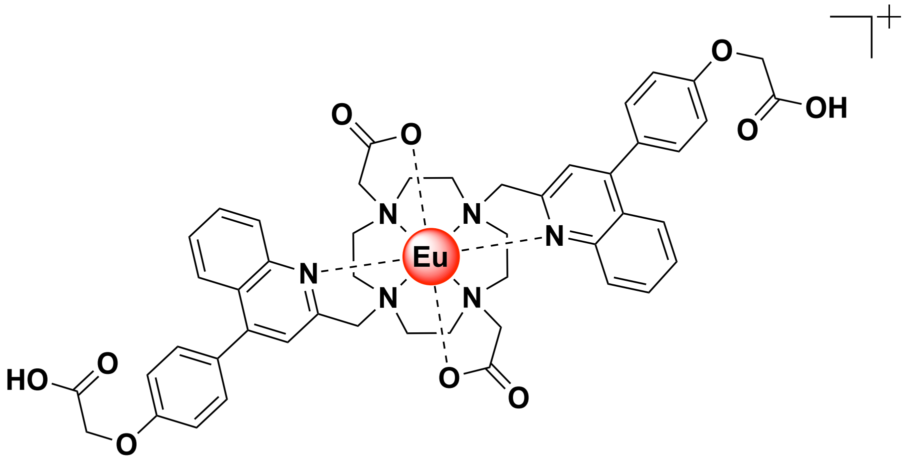


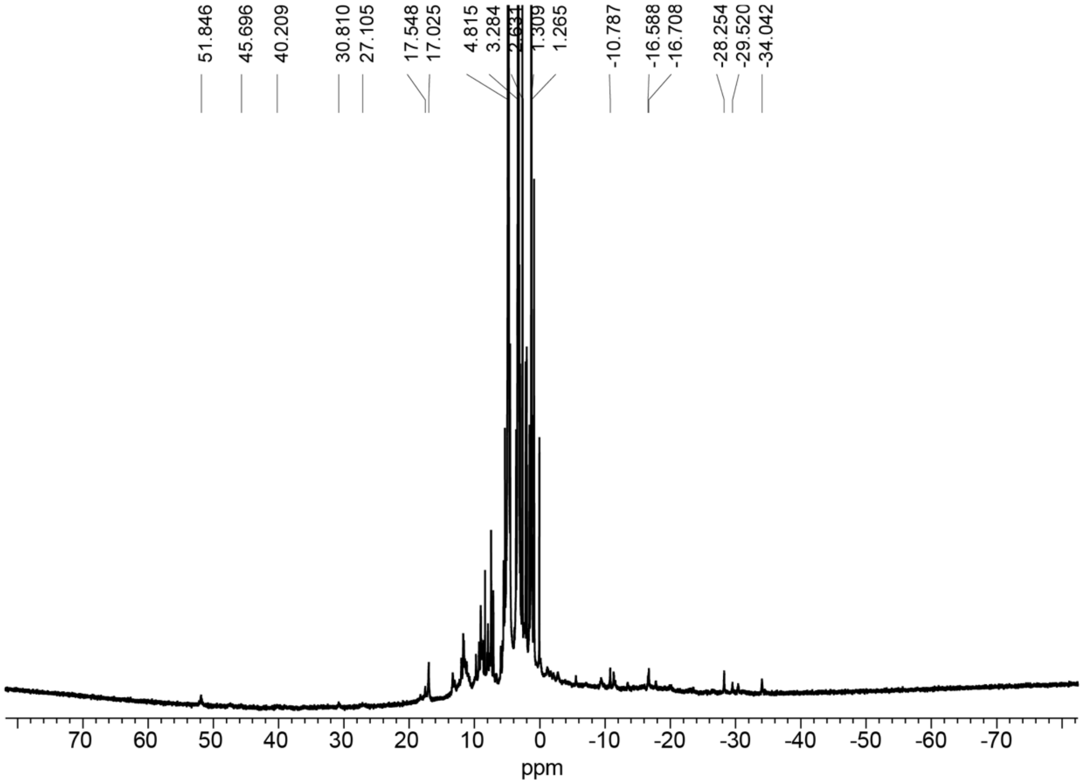


$^1$H NMR (500 MHz, CD$_3$OD, 298 K) of [**Eu.4PhOCH$_2$COO**]$^-$.

7-(4-Methoxyphenyl)-2-methylquinoline (**1d**)

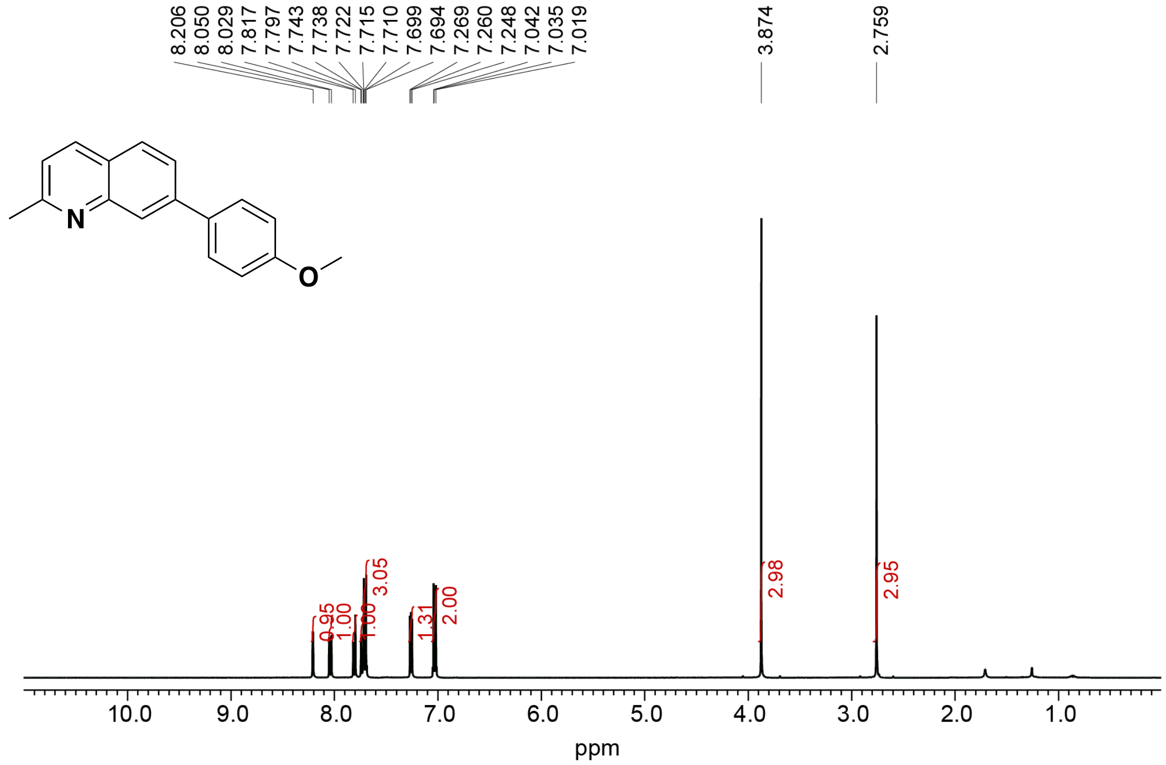


$^{1}$H NMR (400 MHz, $CDCl_3$, 298 K) of 7-(4-methoxyphenyl)-2-methylquinoline (**1d**).

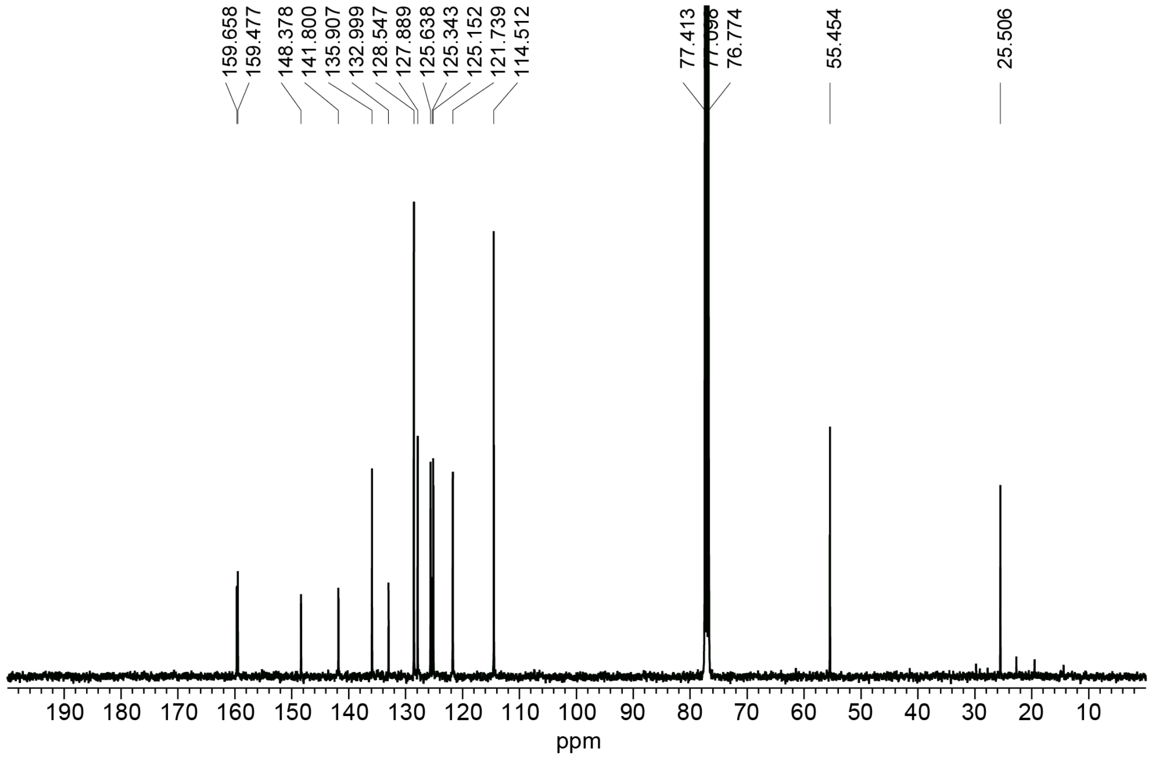


$^{13}$C NMR (101 MHz, $CDCl_3$, 298 K) of 7-(4-methoxyphenyl)-2-methylquinoline (**1d**).

7-(4-Methoxyphenyl)-2-quinolinecarboxaldehyde (**2c**)

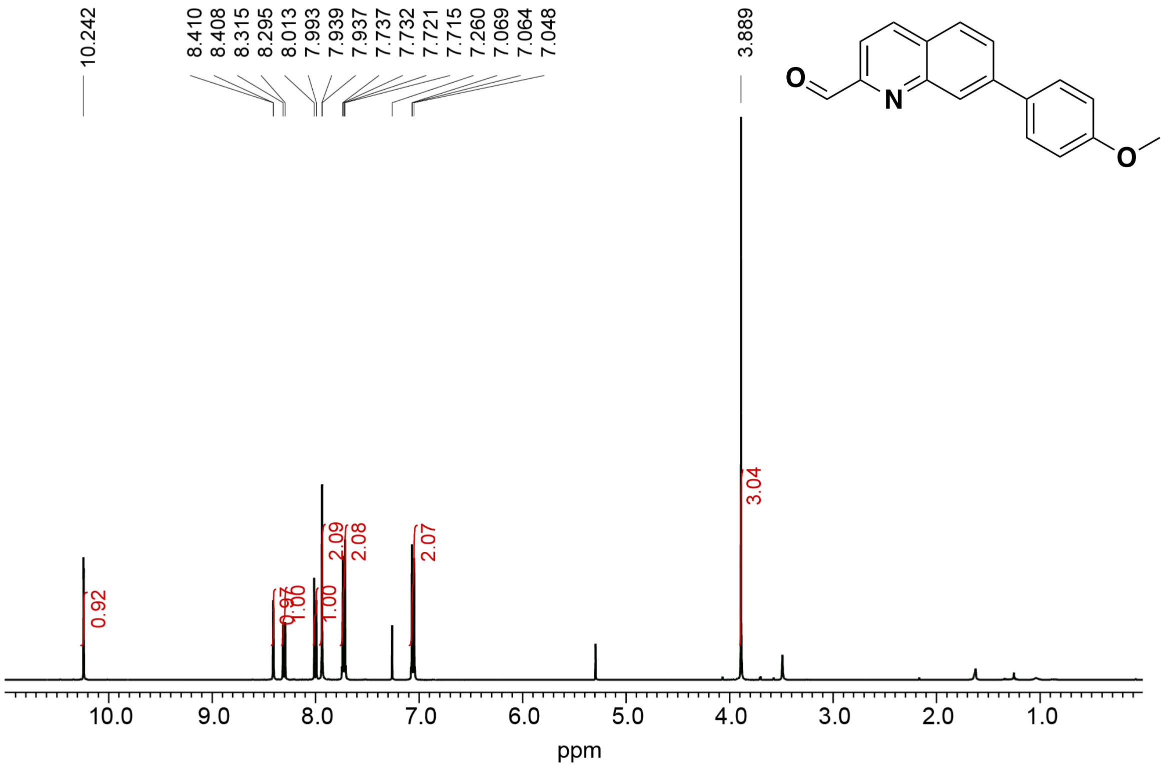


$^{1}$H NMR (400 MHz, $CDCl_3$, 298 K) of 7-(4-methoxyphenyl)-2-quinolinecarboxaldehyde (**2c**).

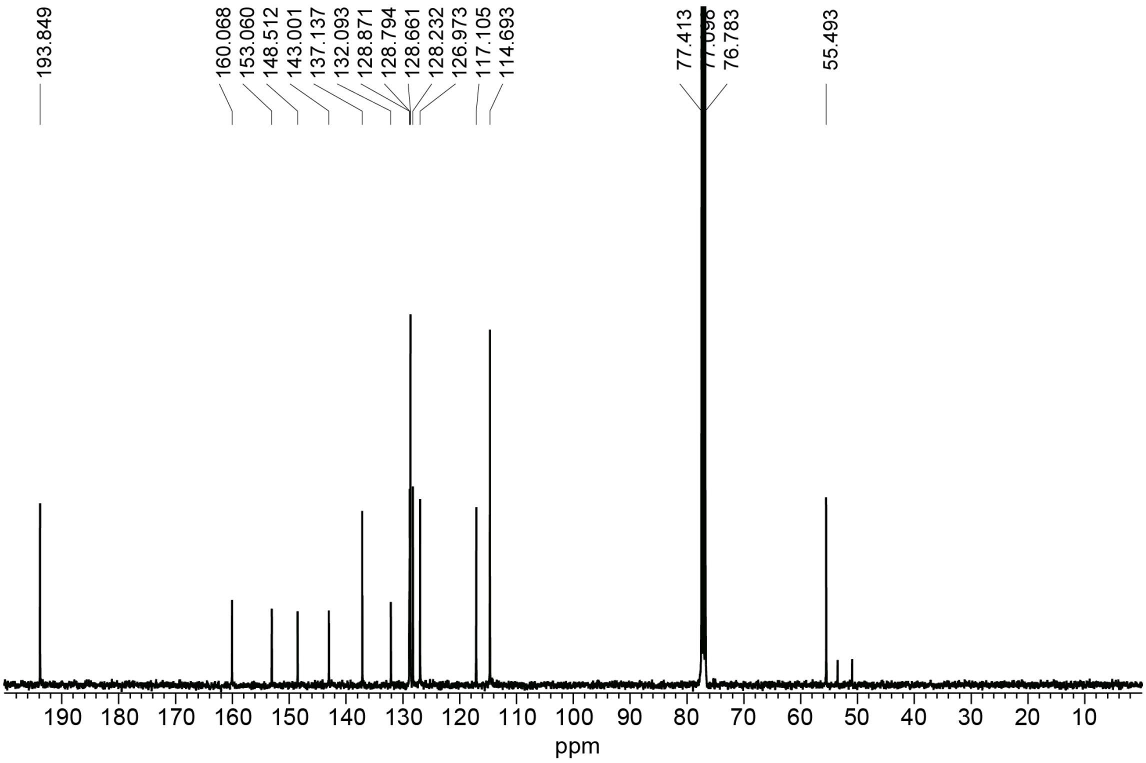


$^{13}$C NMR (101 MHz, $CDCl_3$, 298 K) of 7-(4-methoxyphenyl)-2-quinolinecarboxaldehyde (**2c**).

7-(4-Methoxyphenyl)-2-quinolinemethanol (**3c**)

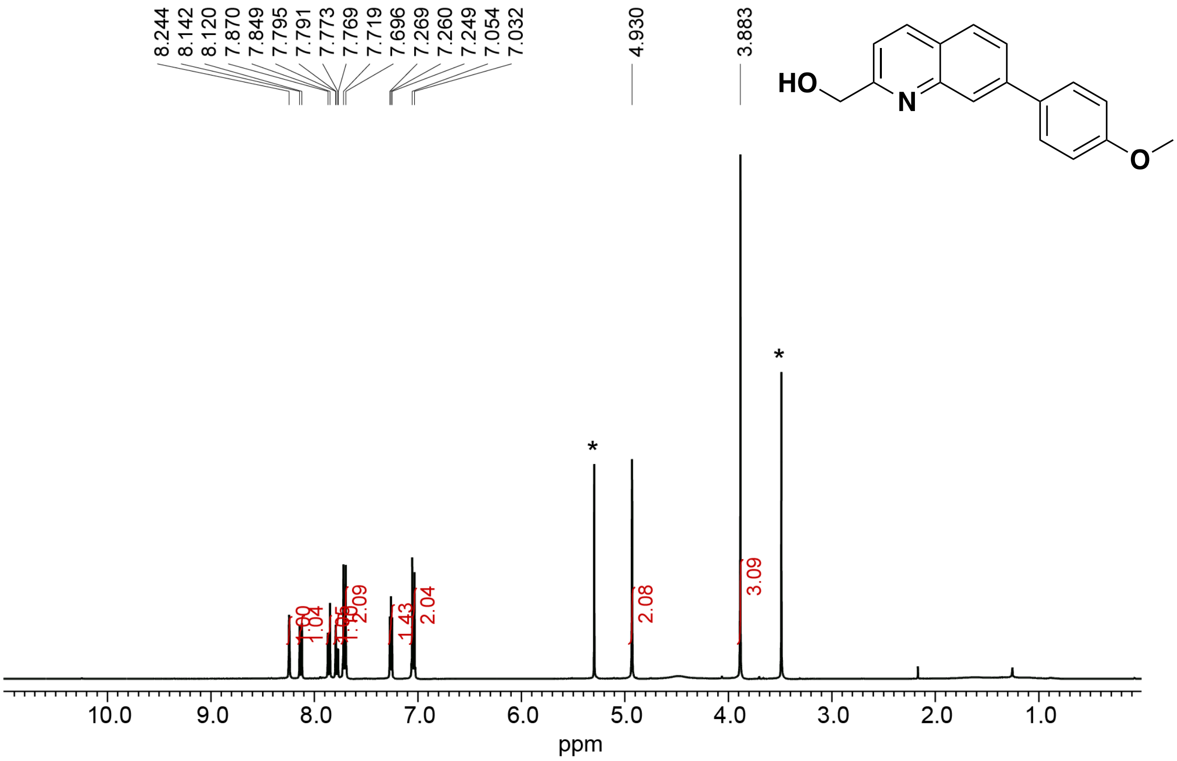


$^{1}$H NMR (500 MHz, $CDCl_3$, 298 K) of 7-(4-methoxyphenyl)-2-quinolinemethanol (**3c**).

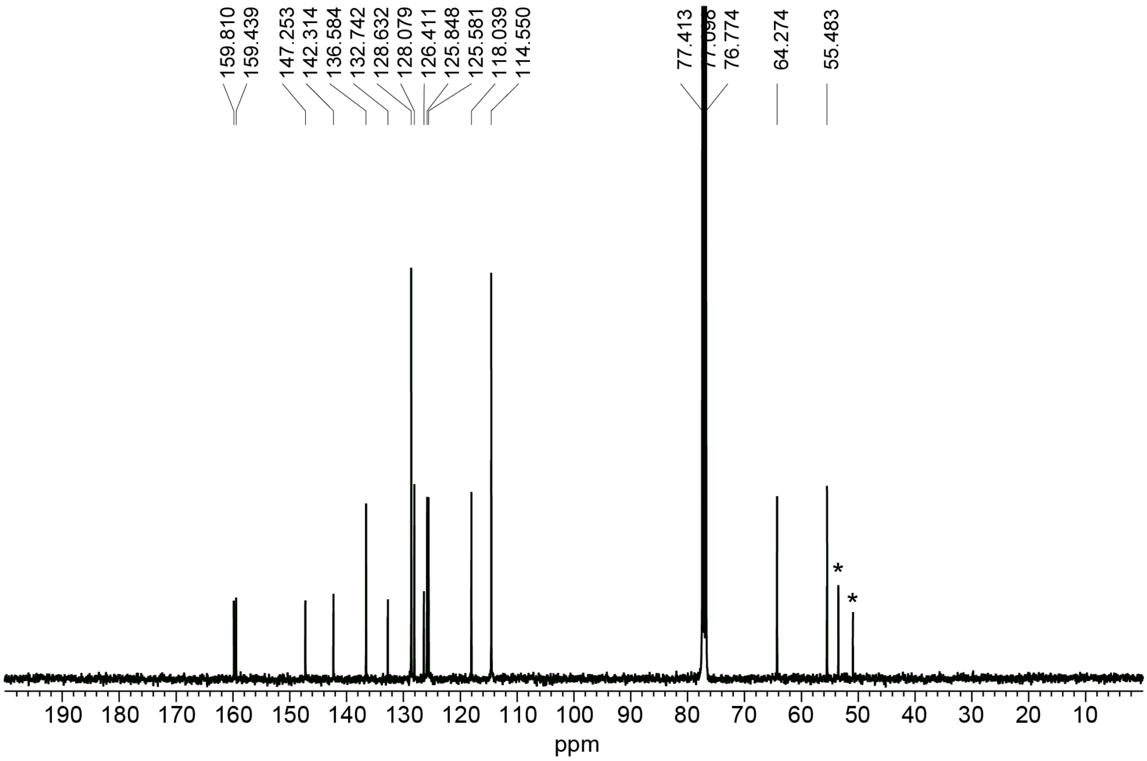


$^{13}$C NMR (126 MHz, $CDCl_3$, 298 K) of 7-(4-methoxyphenyl)-2-quinolinemethanol (**3c**).

7-(4-Methoxyphenyl)- 2-methanesulfonate-2-quinolinemethanol (**4c**)

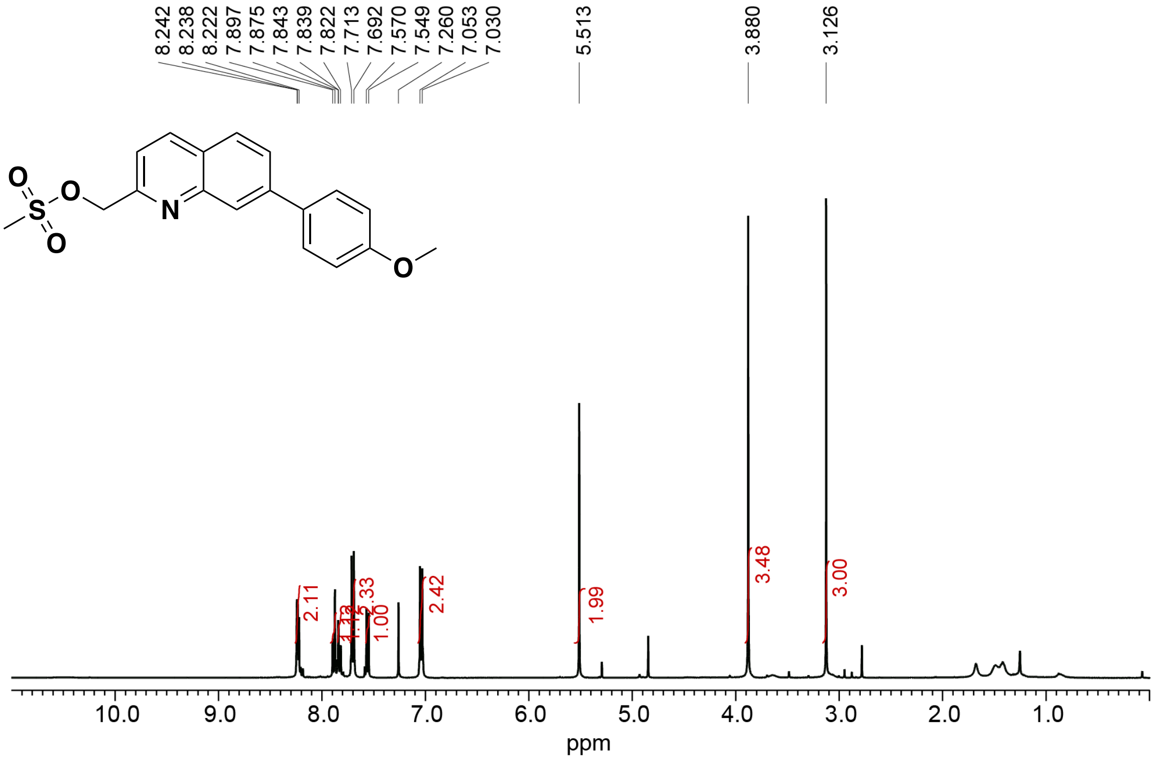


$^1$H NMR (400 MHz, $CDCl_3$, 298 K) of 7-(4-methoxyphenyl)- 2-methanesulfonate-2-quinolinemethanol (**4c**).

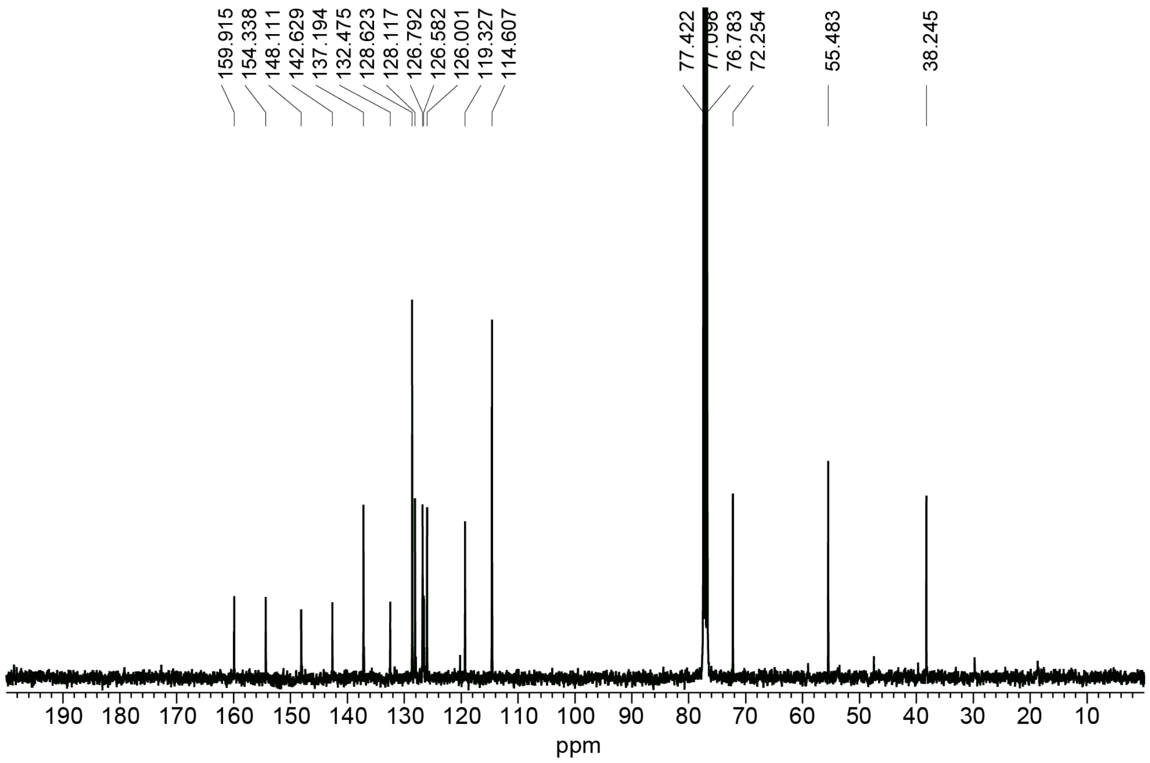


$^{13}$C NMR (101 MHz, $CDCl_3$, 298 K) of 7-(4-methoxyphenyl)- 2-methanesulfonate-2-quinolinemethanol (**4c**).

4,10-*Bis*((7-(4-methoxyphenyl)-quinolin-2-yl)-methyl)-1,4,7,10-tetraazacyclododecane-1,7-diyl)-diacetate (**5c**)

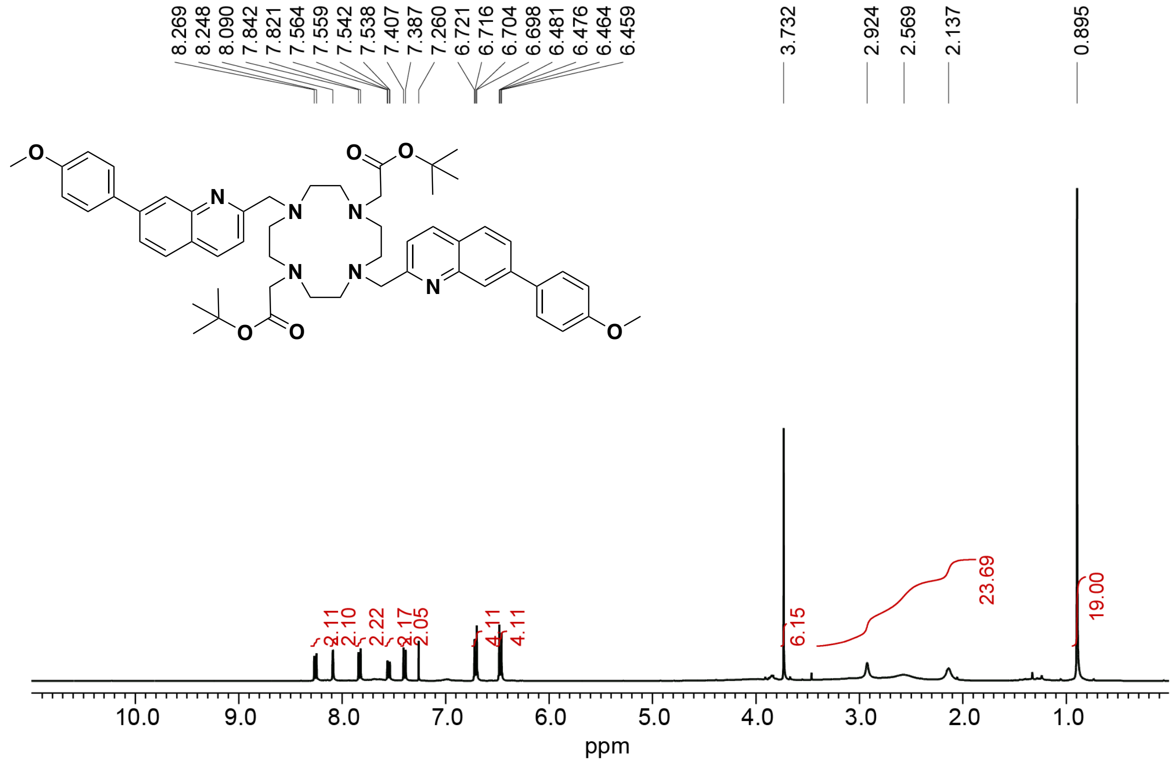


$^{1}$H NMR (400 MHz, $CDCl_3$, 298 K) of 4,10-*bis*((7-(4-methoxyphenyl)-quinolin-2-yl)-methyl)-1,4,7,10-tetraazacyclododecane-1,7-diyl)-diacetate (**5c**).

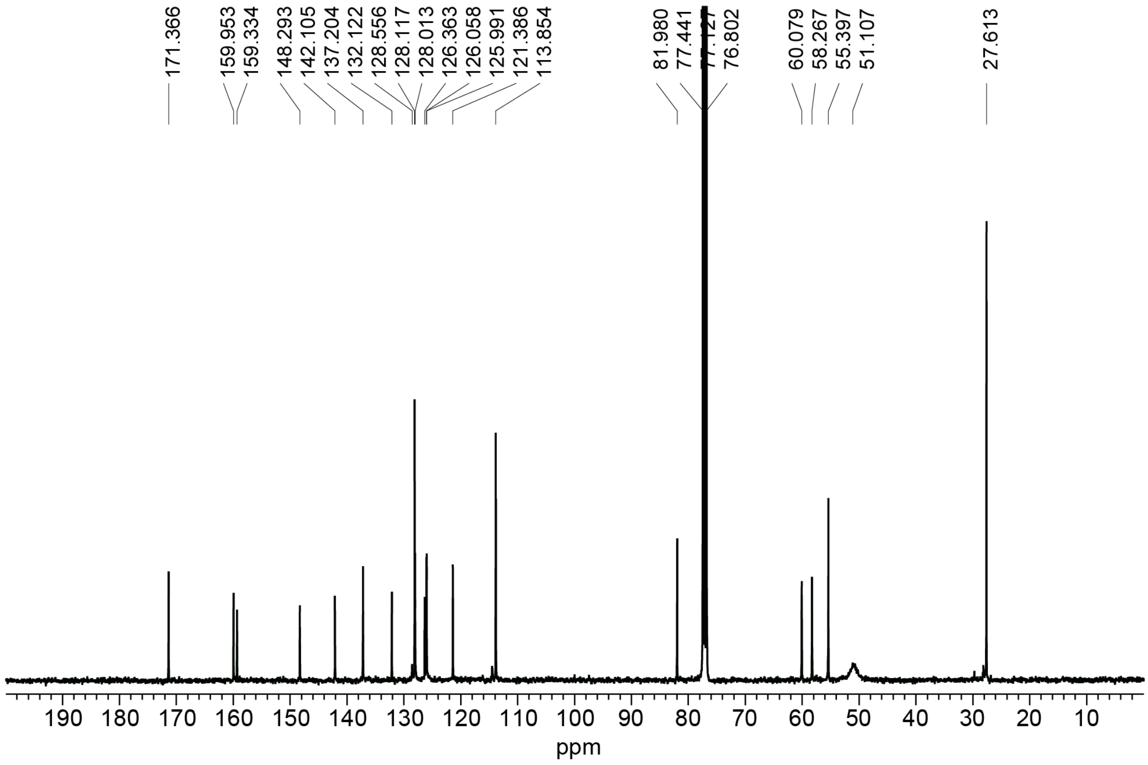


$^{13}$C NMR (101 MHz, $CDCl_3$, 298 K) of 4,10-*bis*((7-(4-methoxyphenyl)-quinolin-2-yl)-methyl)-1,4,7,10-tetraazacyclododecane-1,7-diyl)-diacetate (**5c**).

4,10-*Bis*((7-(4-methoxyphenyl)-quinolin-2-yl)-methyl)-1,4,7,10-tetraazacyclododecane-1,7-diyl)-diacetic acid (**6c**)

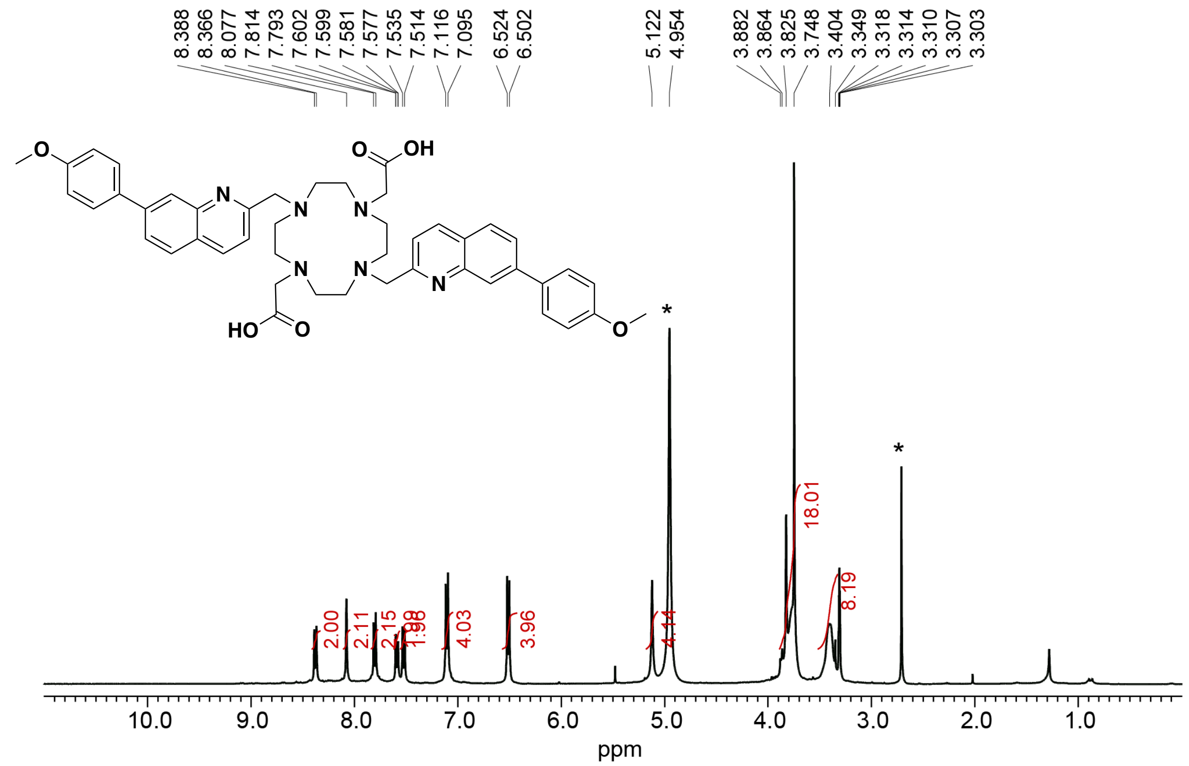


$^{1}$H NMR (500 MHz, $CD_3OD$, 298 K) of 4,10-*bis*((7-(4-methoxyphenyl)-quinolin-2-yl)-methyl)-1,4,7,10-tetraazacyclododecane-1,7-diyl)-diacetic acid (**6c**).

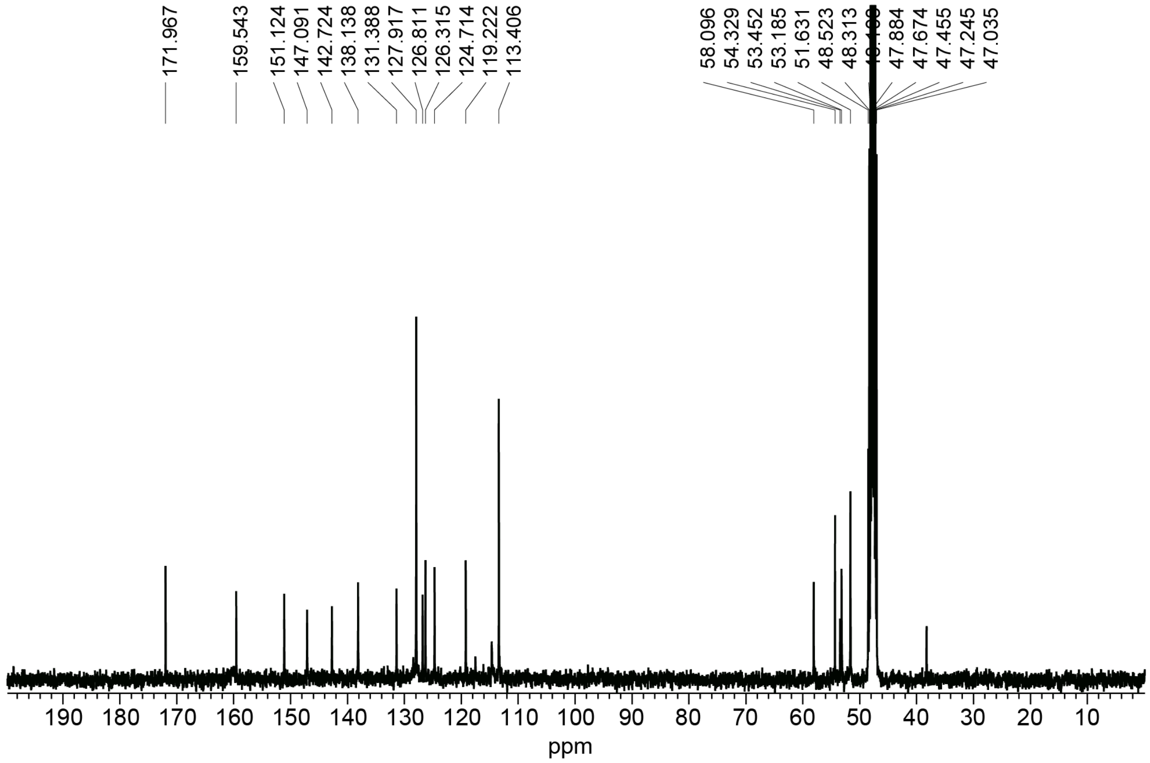


$^{13}$C NMR (126 MHz, $CD_3OD$, 298 K) of 4,10-*bis*((7-(4-methoxyphenyl)-quinolin-2-yl)-methyl)-1,4,7,10-tetraazacyclododecane-1,7-diyl)-diacetic acid (**6c**).

[Eu.7PhOMe]+

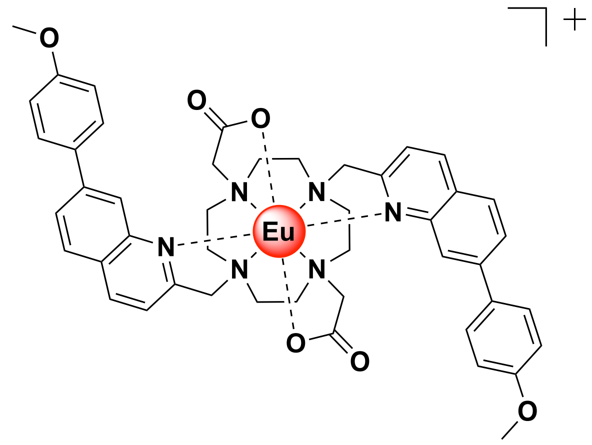

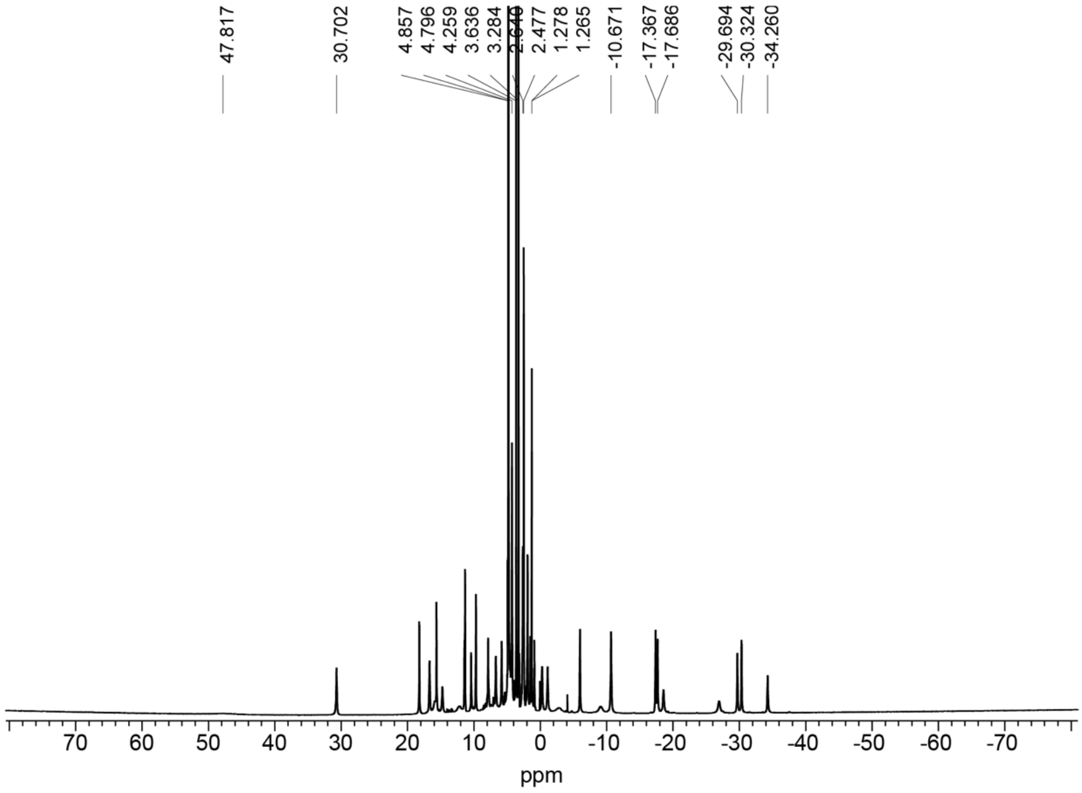


$^1$H NMR (500 MHz, $CD_3OD$, 298 K) of **[Eu.7PhOMe]**$^+$.

7-(2-Methyl-4-quinolinyl)-phenol (**1e**)

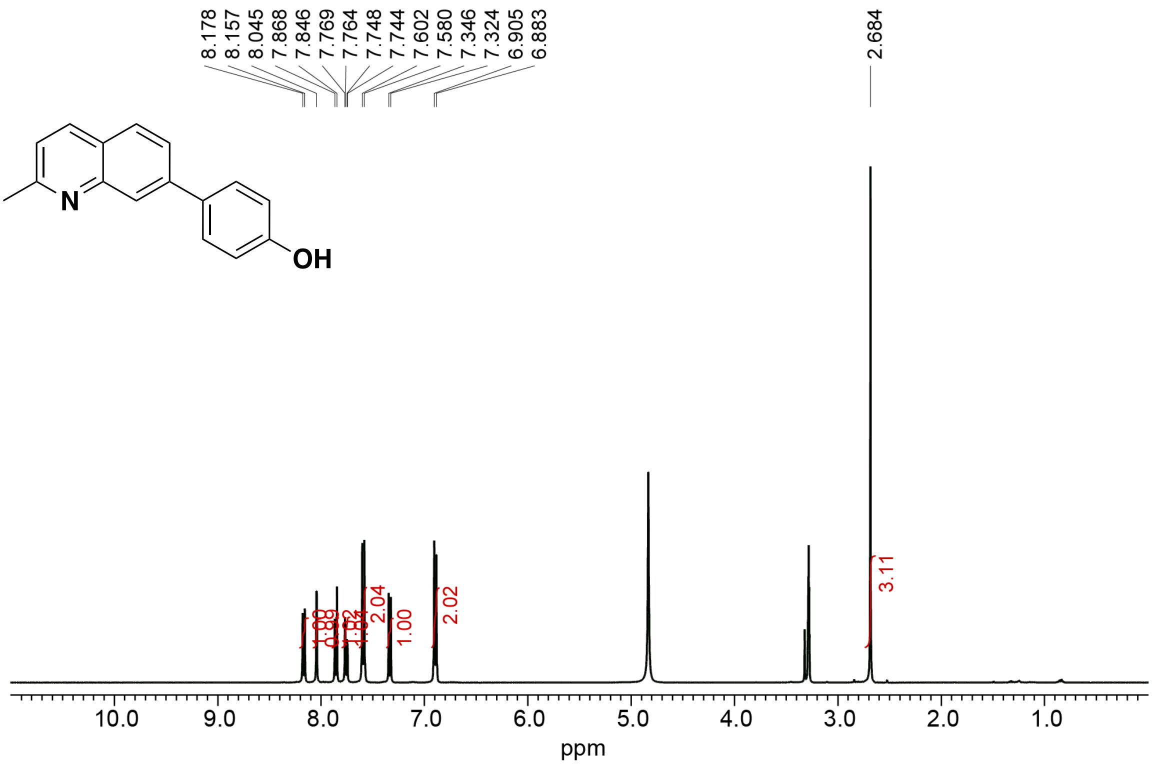


$^1$H NMR (400 MHz, DMSO-$d_6$, 298 K) of 7-(2-methyl-4-quinolinyl)-phenol (**1e**).

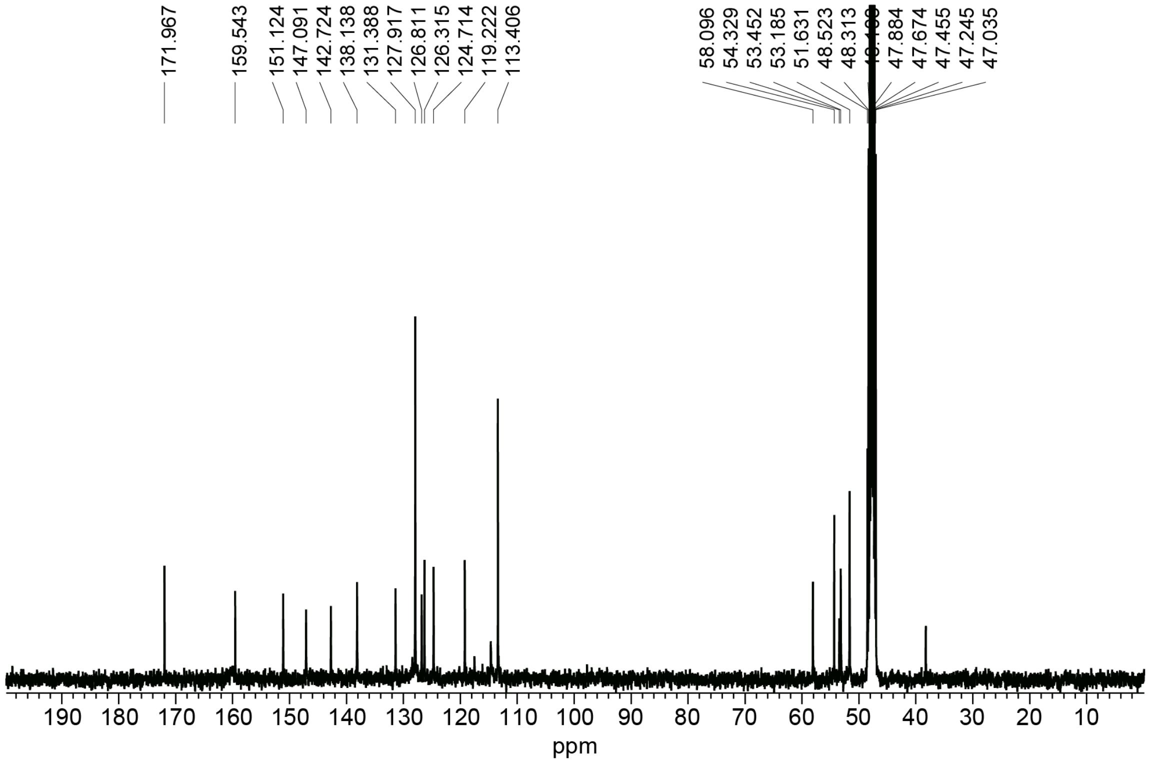


$^{13}$C NMR (101 MHz, DMSO-$d_6$, 298 K) of 7-(2-methyl-4-quinolinyl)-phenol (**1e**).

7-(4-(Phenoxy)*tert*-butyl acetate)-2-methylquinoline or 4-(4-(1,1-dimethylethyl)-phenoxyacetate)-2-methylquinoline (**1f**)

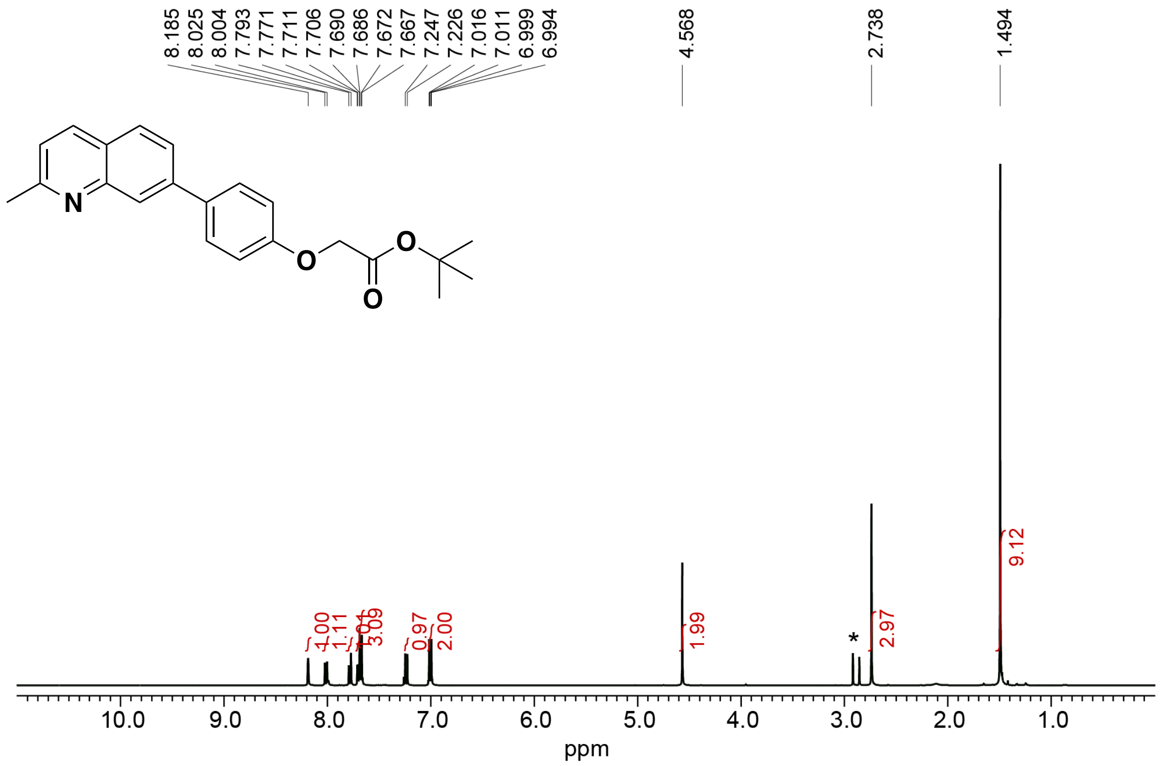


$^1$H NMR (400 MHz, $CDCl_3$, 298 K) of 7-(4-(phenoxy)*tert*-butyl acetate)-2-methylquinoline or 4-(4-(1,1-dimethylethyl)-phenoxyacetate)-2-methylquinoline (**1f**).

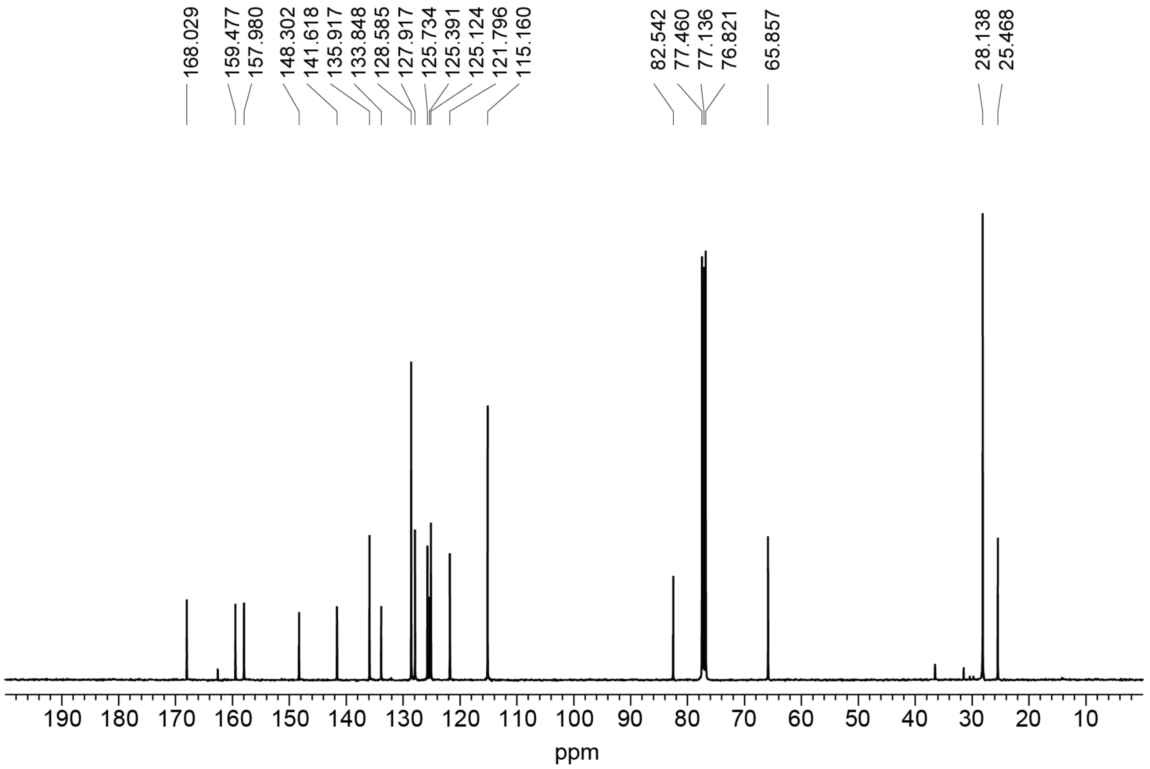


$^{13}$C NMR (101 MHz, $CDCl_3$, 298 K) of 7-(4-(phenoxy)*tert*-butyl acetate)-2-methylquinoline or 4-(4-(1,1-dimethylethyl)-phenoxyacetate)-2-methylquinoline (**1f**).

7-(4-(1,1-Dimethylethyl)-phenoxyacetate)-2-quinolinecarboxaldehyde (**2d**)

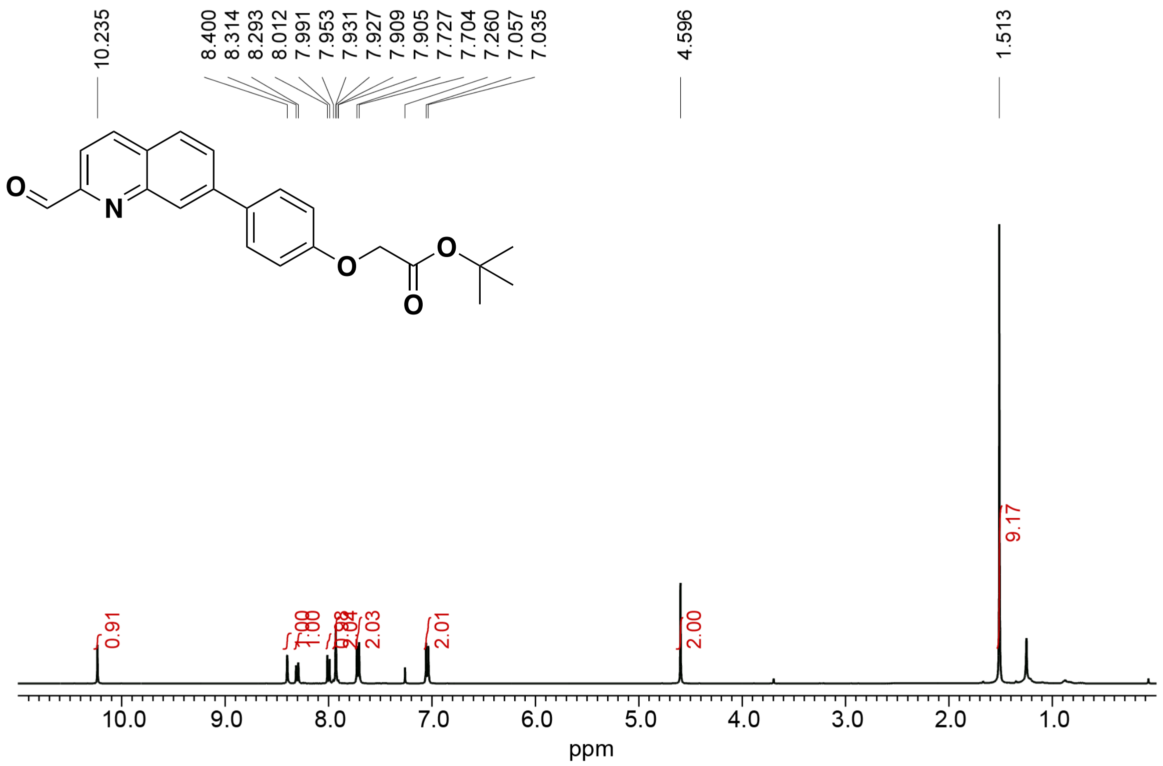


$^{1}$H NMR (400 MHz, $CDCl_3$, 298 K) of 7-(4-(1,1-dimethylethyl)-phenoxyacetate)-2-quinolinecarboxaldehyde (**2d**).

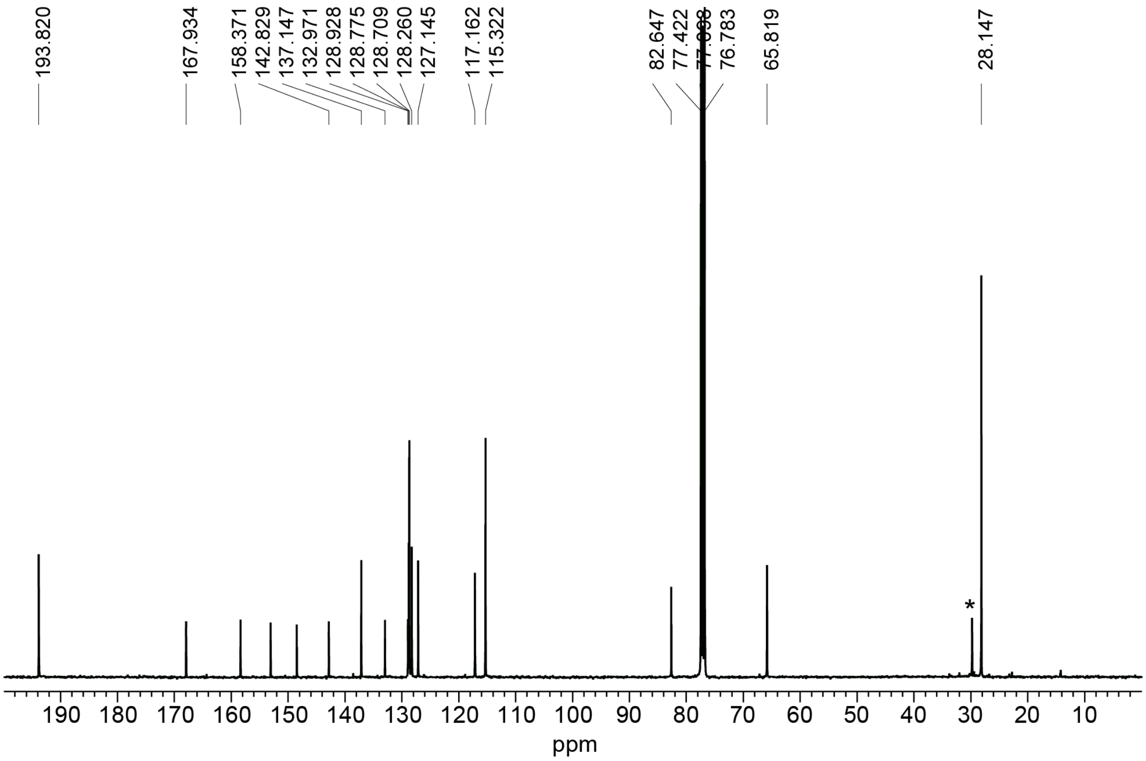


$^{13}$C NMR (101 MHz, $CDCl_3$, 298 K) of 7-(4-(1,1-dimethylethyl)-phenoxyacetate)-2-quinolinecarboxaldehyde (**2d**).

7-(4-(1,1-dimethylethyl)-phenoxyacetate)-2-quinolinemethanol (**3d**)

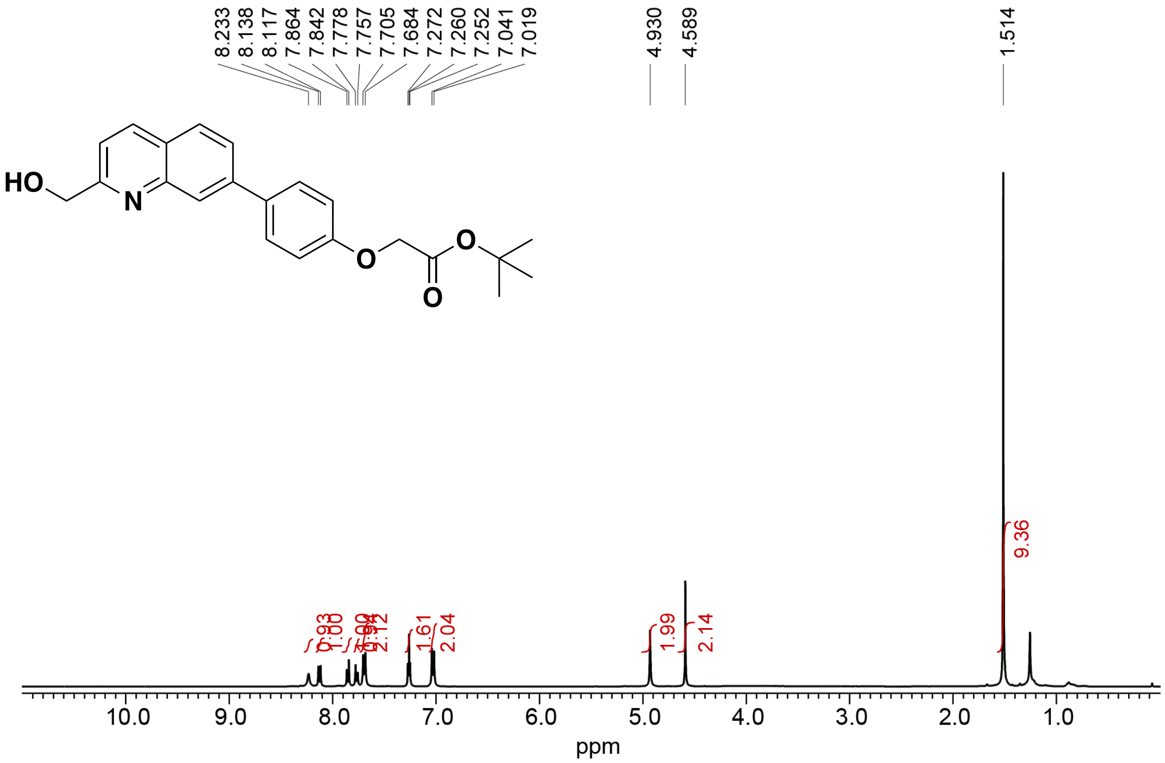


$^{1}H$ NMR (400 MHz, $CDCl_3$, 298 K) of 7-(4-(1,1-dimethylethyl)-phenoxyacetate)-2-quinolinemethanol (**3d**).

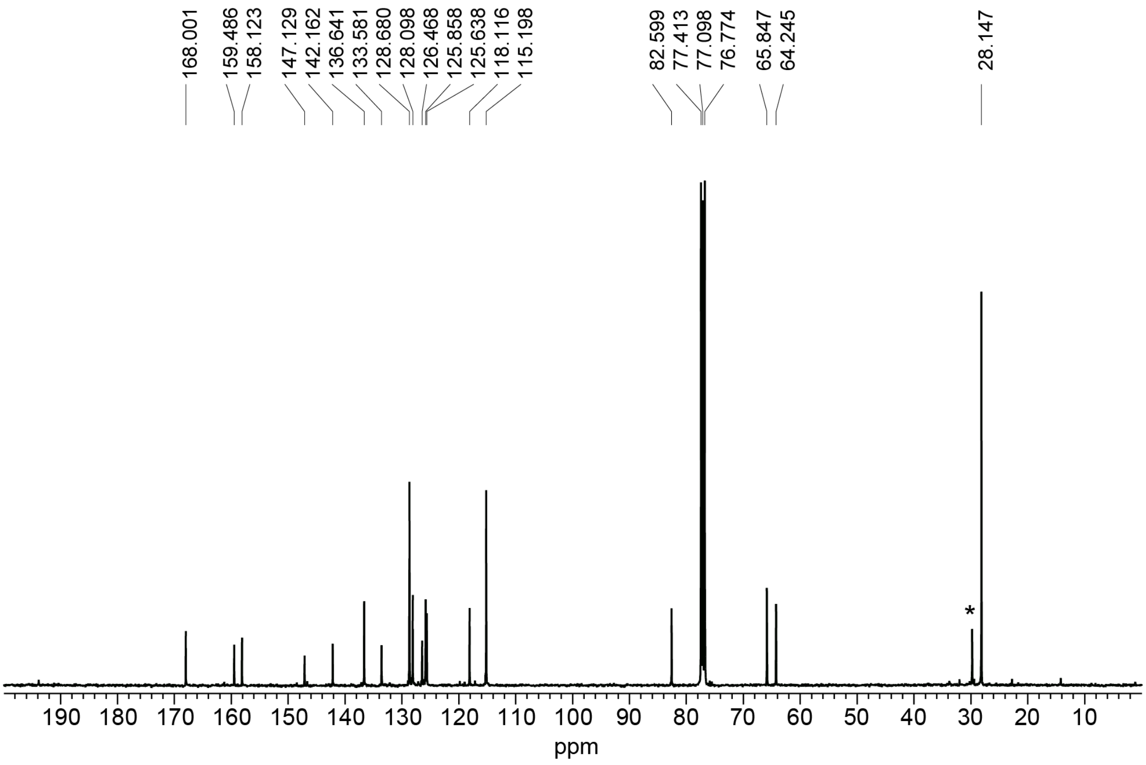


$^{13}C$ NMR (101 MHz, $CDCl_3$, 298 K) of 7-(4-(1,1-dimethylethyl)-phenoxyacetate)-2-quinolinemethanol (**3d**).

7-(4-(1,1-Dimethylethyl)-phenoxyacetate)-2-methanesulfonate-2-quinolinemethanol (**4d**)

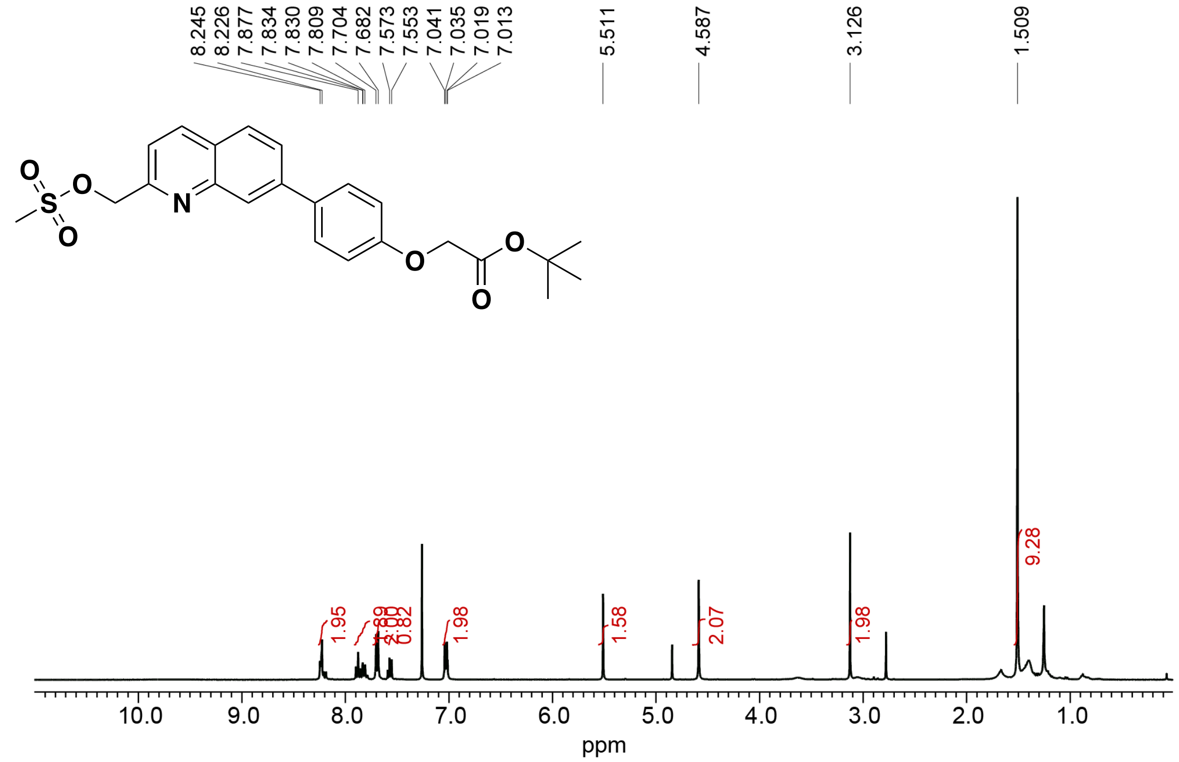


$^1$H NMR (400 MHz, $CDCl_3$, 298 K) of 7-(4-(1,1-dimethylethyl)-phenoxyacetate)-2-methanesulfonate-2-quinolinemethanol (**4d**).

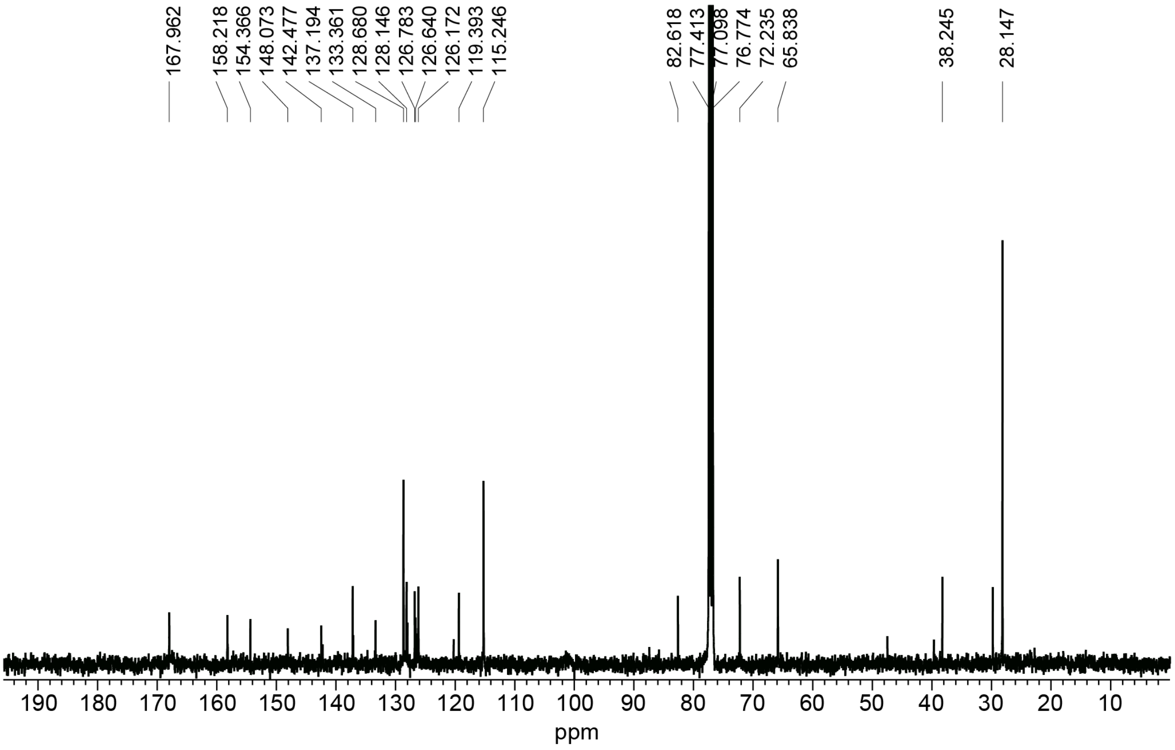


$^{13}$C NMR (101 MHz, $CDCl_3$, 298 K) of 7-(4-(1,1-dimethylethyl)-phenoxyacetate)-2-methanesulfonate-2-quinolinemethanol (**4d**).

4,10-*Bis*((7-(4-(1,1-dimethylethyl)-phenoxyacetate)-quinolin-2-yl)-methyl)-1,4,7,10-tetraazacyclododecane-1,7-diyl)-diacetate (**5d**)

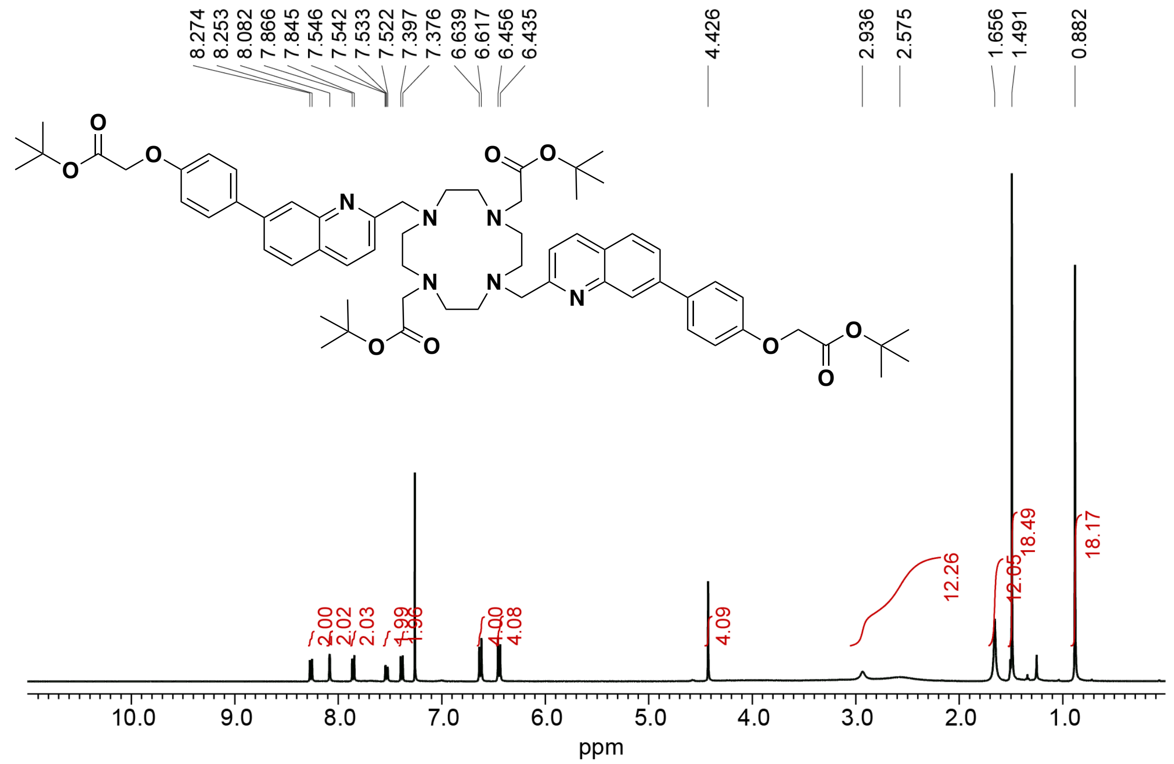


$^{1}$H NMR (500 MHz, $CDCl_3$, 298 K) of 4,10-*bis*((7-(4-(1,1-dimethylethyl)-phenoxyacetate)-quinolin-2-yl)-methyl)-1,4,7,10-tetraazacyclododecane-1,7-diyl)-diacetate (**5d**).

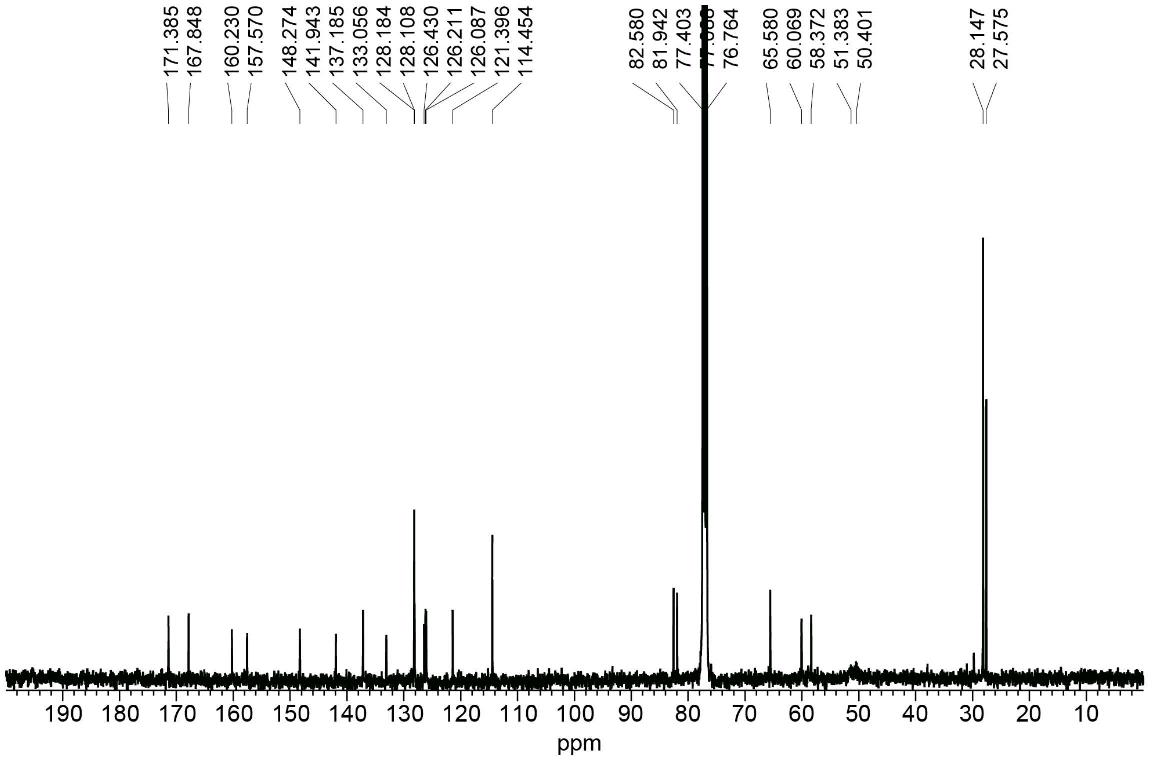


$^{13}$C NMR (126 MHz, $CDCl_3$, 298 K) of 4,10-*bis*((7-(4-(1,1-dimethylethyl)-phenoxyacetate)-quinolin-2-yl)-methyl)-1,4,7,10-tetraazacyclododecane-1,7-diyl)-diacetate (**5d**).

4,10-*Bis*((7-(4-(1,1-dimethylethyl)-phenoxyacetate)-quinolin-2-yl)-methyl)-1,4,7,10-tetraazacyclododecane-1,7-diyl)-diacetic acid (**6d**)

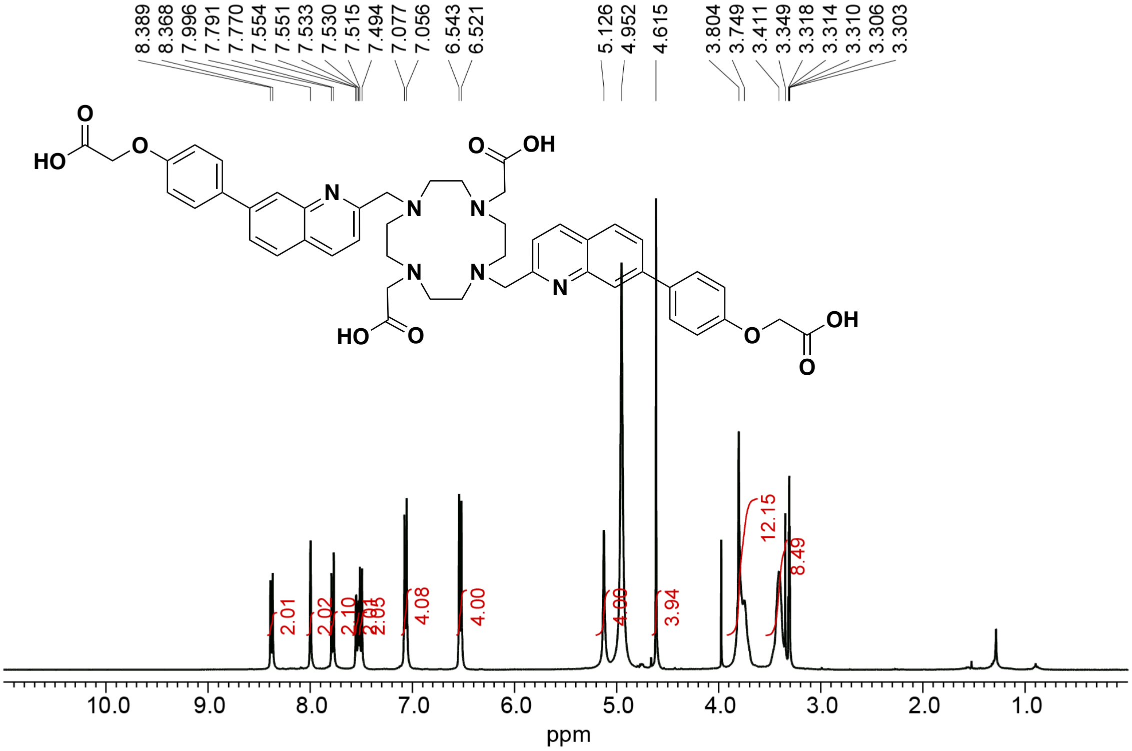


$^{1}$H NMR (500 MHz, $CD_3OD$, 298 K) of 4,10-*bis*((7-(4-(1,1-dimethylethyl)-phenoxyacetate)-quinolin-2-yl)-methyl)-1,4,7,10-tetraazacyclododecane-1,7-diyl)-diacetic acid (**6d**).

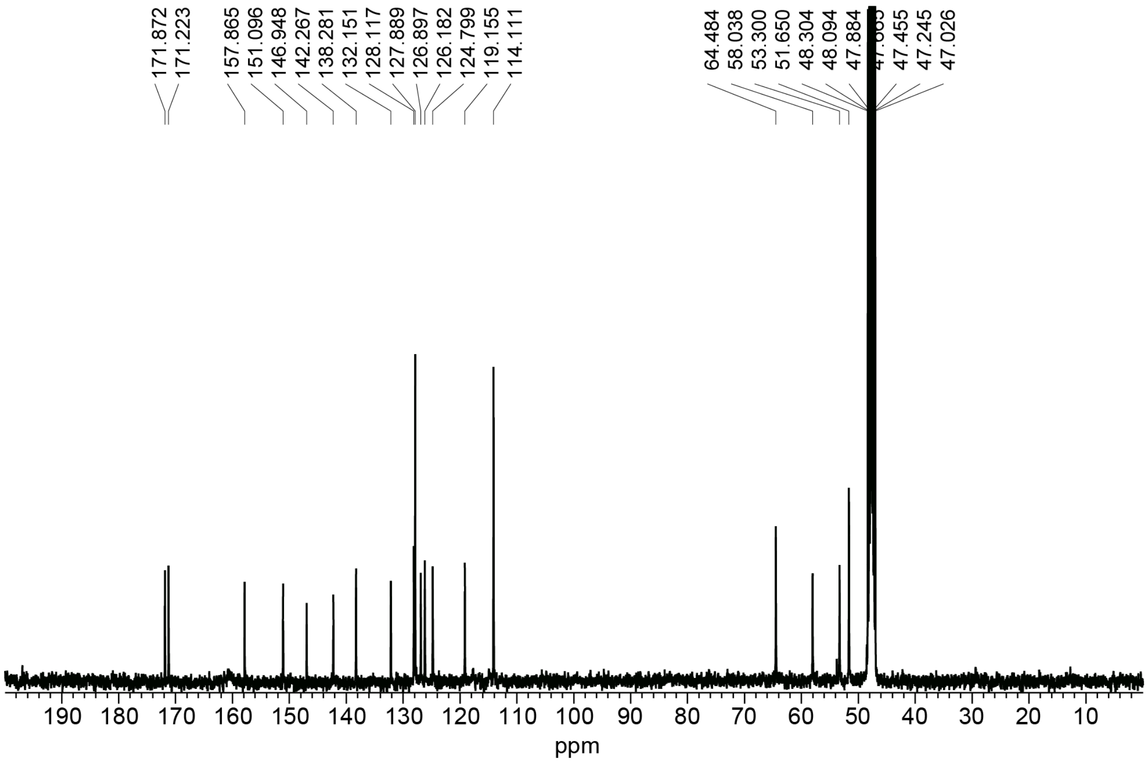


$^{13}$C NMR (126 MHz, $CD_3OD$, 298 K) of 4,10-*bis*((7-(4-(1,1-dimethylethyl)-phenoxyacetate)-quinolin-2-yl)-methyl)-1,4,7,10-tetraazacyclododecane-1,7-diyl)-diacetic acid (**6d**).

**[Eu.7PhOCH₂COO]⁻**

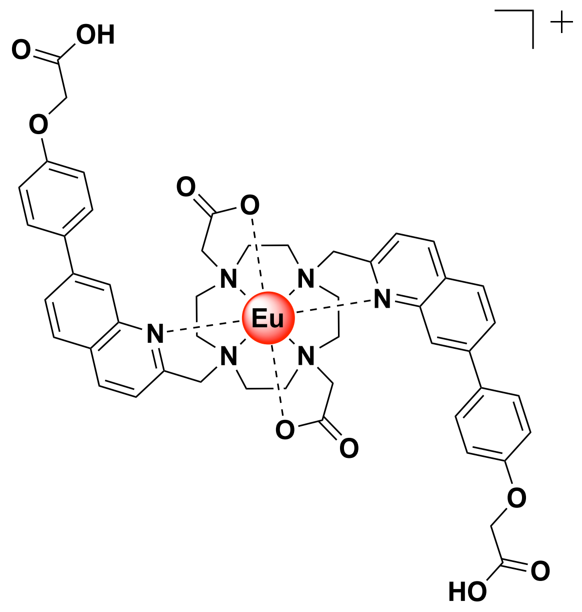

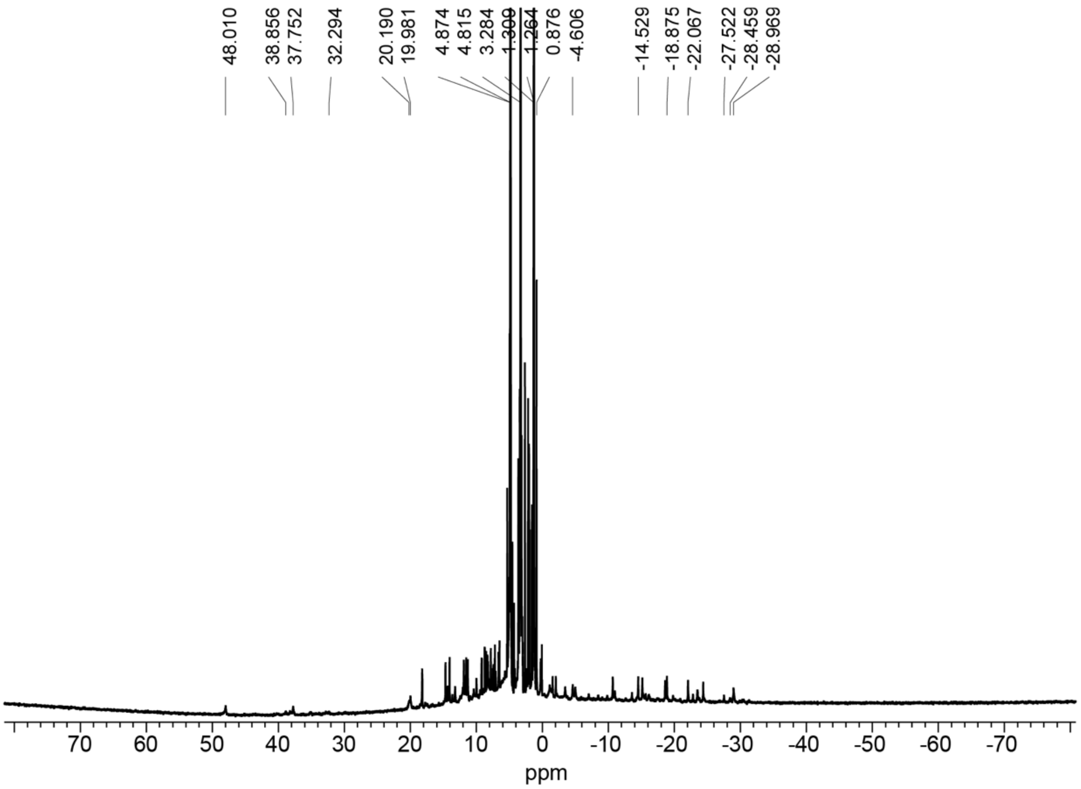


$^{1}$H NMR (500 MHz, $CD_3OD$, 298 K) of **[Eu.7PhOCH$_2$COO]**$^-$.

## Appendix 2: High-resolution and Low-resolution Mass Spectra

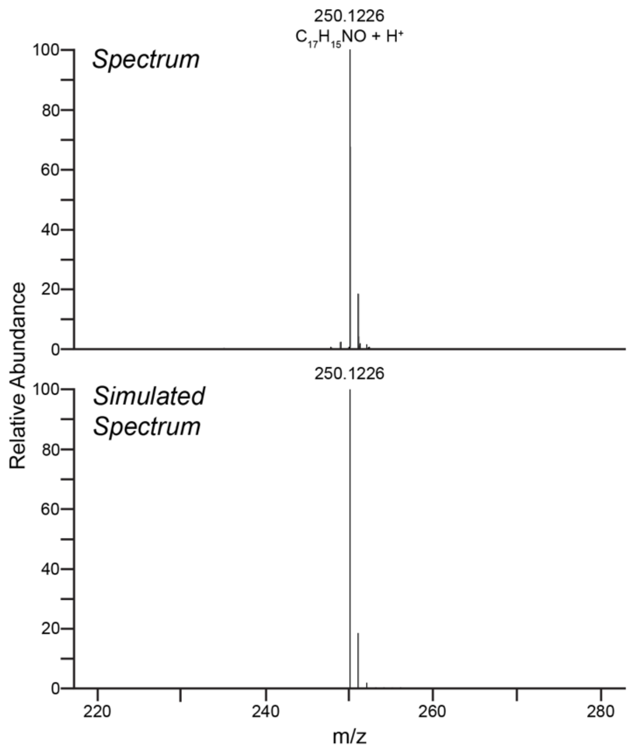


High-resolution mass spectra of 4-(4-methoxyphenyl)-2-methylquinoline (**1a**).

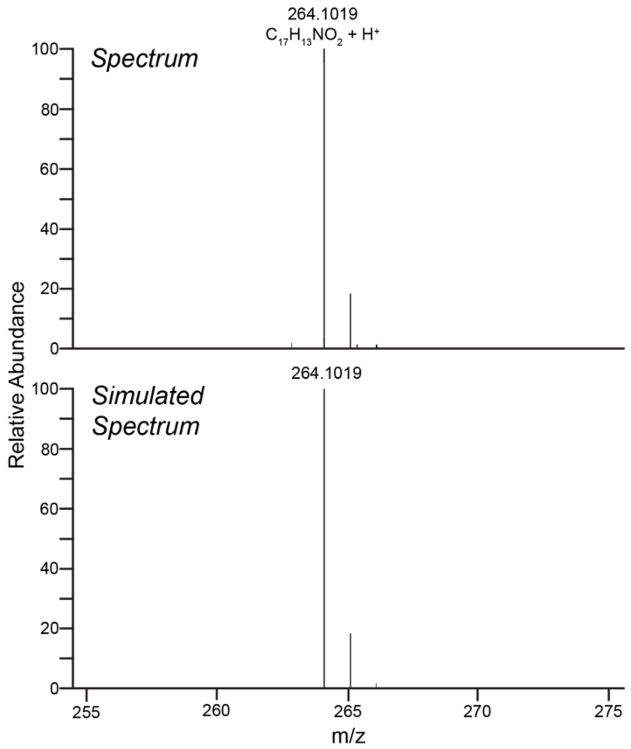


High-resolution mass spectra of 4-(4-methoxyphenyl)-2-quinolinecarboxaldehyde (**2a**).

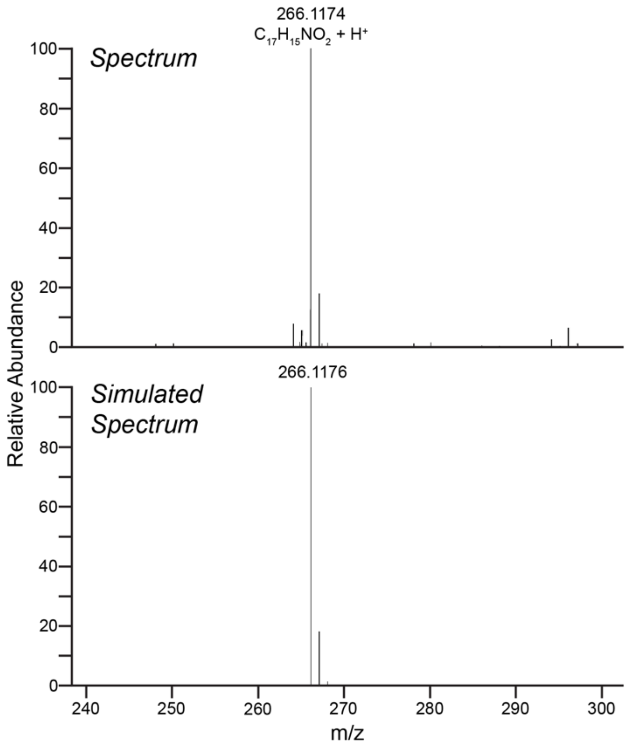


High-resolution mass spectra of 4-(4-methoxyphenyl)-2-quinolinemethanol (**3a**).

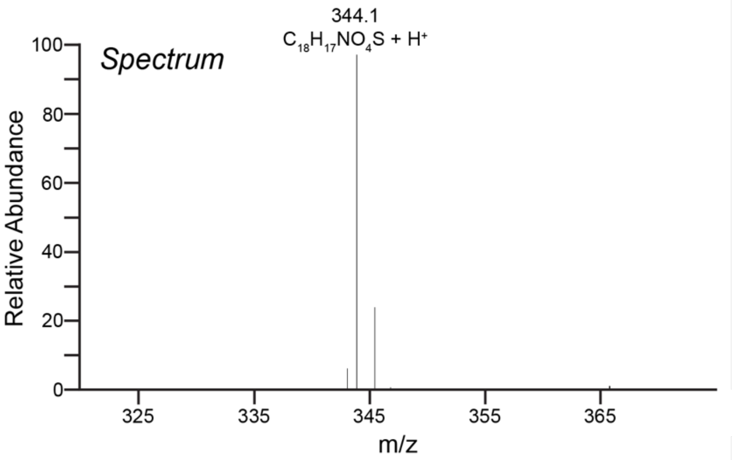


Low-resolution mass spectrum of 4-(4-methoxyphenyl)- 2-methanesulfonate-2-quinolinemethanol (**4a**).

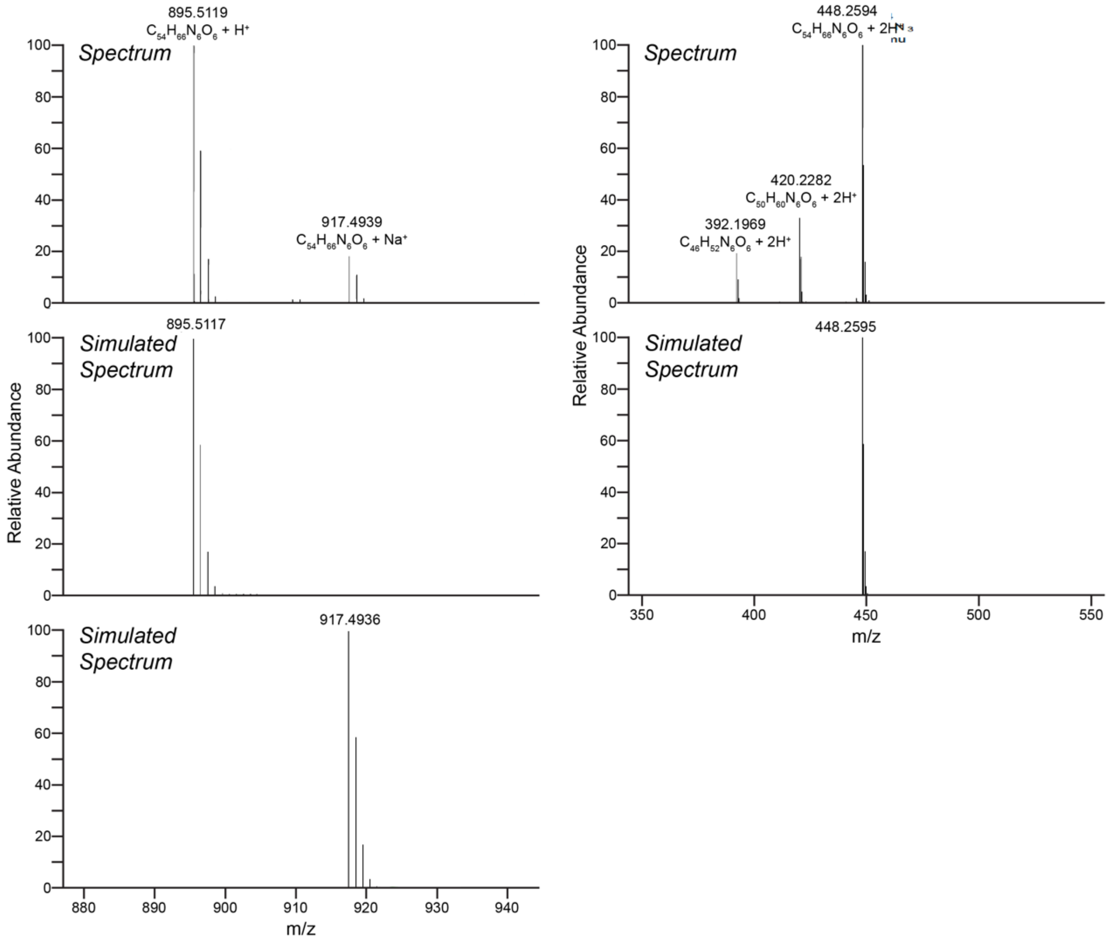


High-resolution mass spectra of 4,10-*bis*((4-(4-methoxyphenyl)-quinolin-2-yl)-methyl)-1,4,7,10-tetraazacyclododecane-1,7-diyl)-diacetate (**5a**).

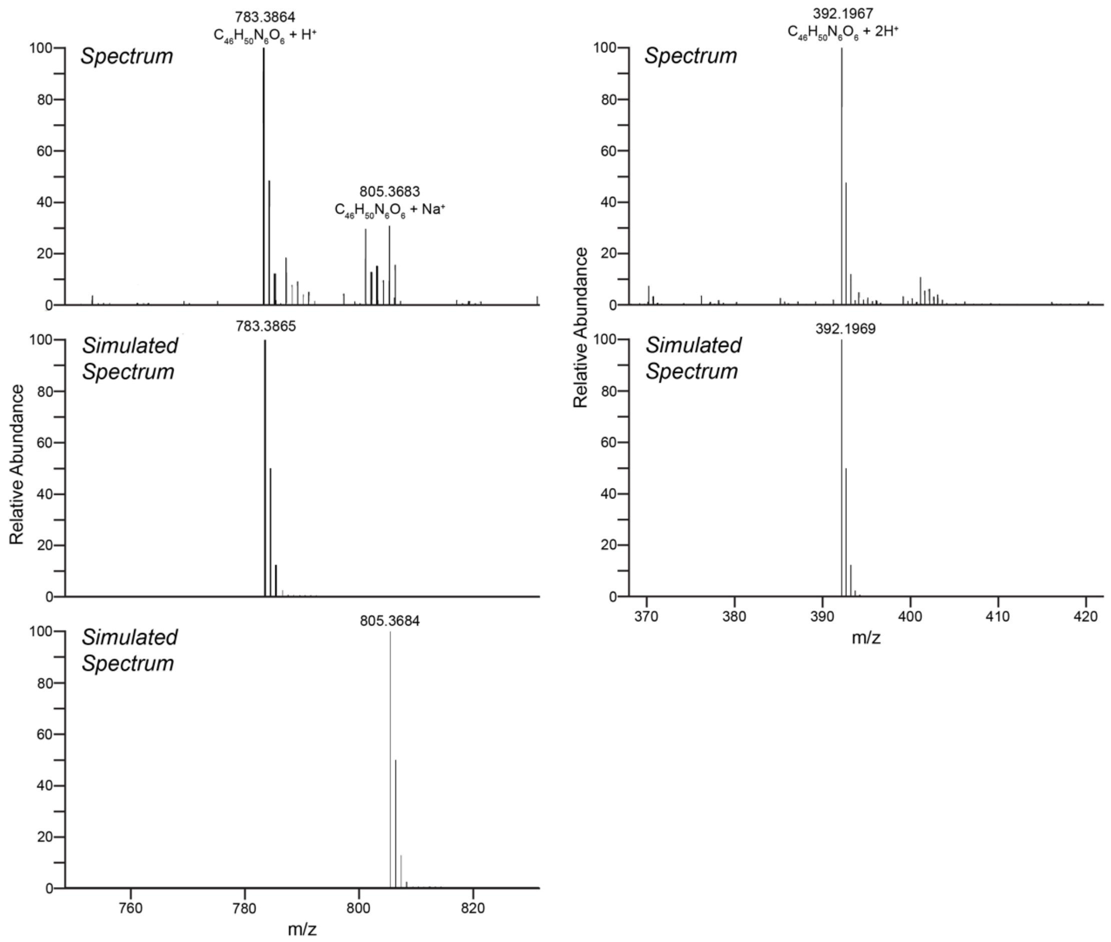


High-resolution mass spectra of 4,10-*bis*((4-(4-methoxyphenyl)-quinolin-2-yl)-methyl)-1,4,7,10-tetraazacyclododecane-1,7-diyl)-diacetic acid (**6a**).

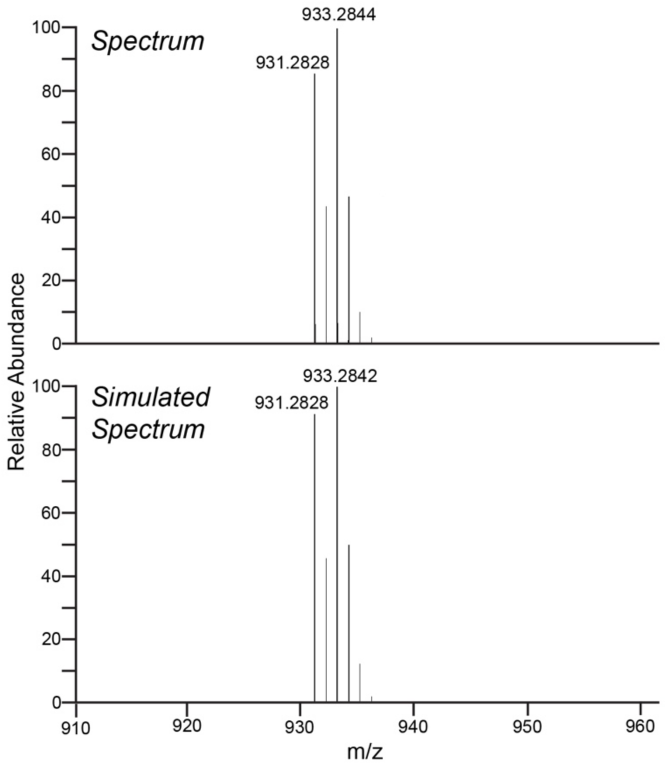


High-resolution mass spectra of [**Eu.4PhOMe**]$^{+}$.

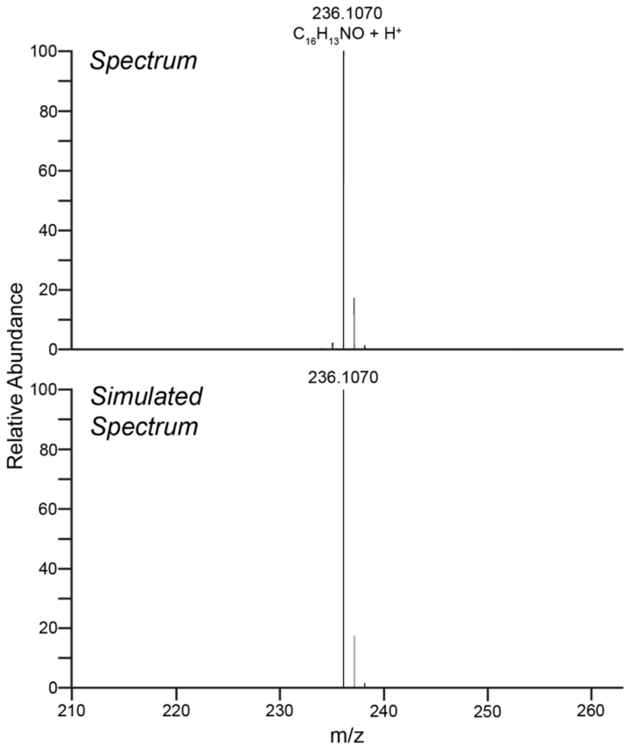


High-resolution mass spectra of 4-(2-methyl-4-quinolinyl)-phenol (**7a**).

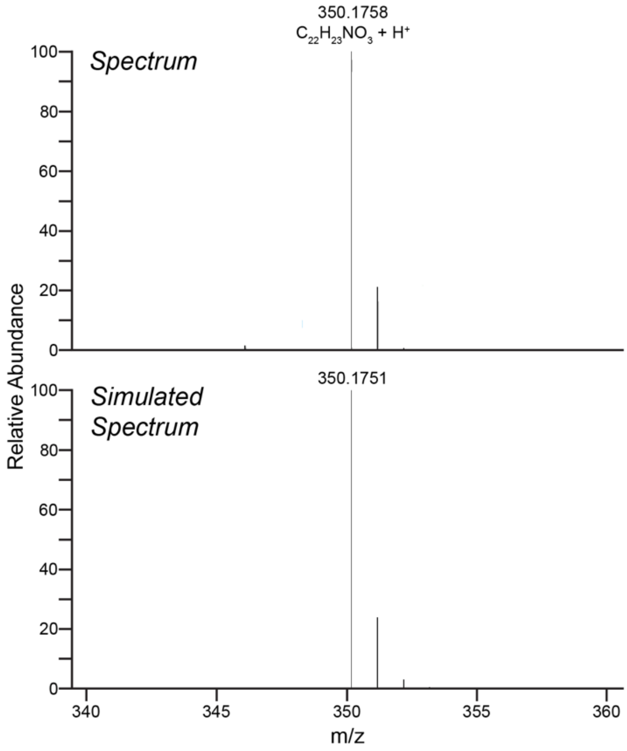


High-resolution mass spectra of 4-(4-(phenoxy)*tert*-butyl acetate)-2-methylquinoline or 4-(4-(1,1-dimethylethyl)-phenoxyacetate)-2-methylquinoline (**8a**).

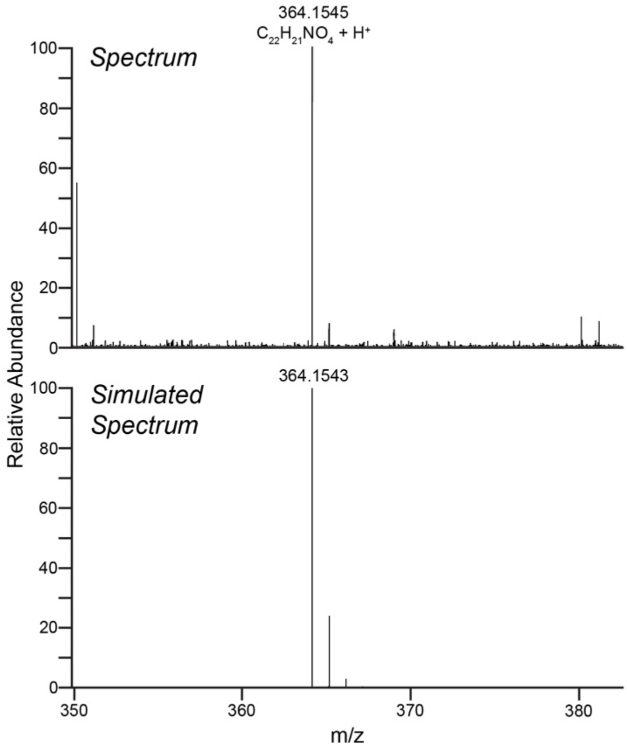


High-resolution mass spectra of 4-(4-(1,1-dimethylethyl)-phenoxyacetate)-2-quinolinecarboxaldehyde (**2b**).

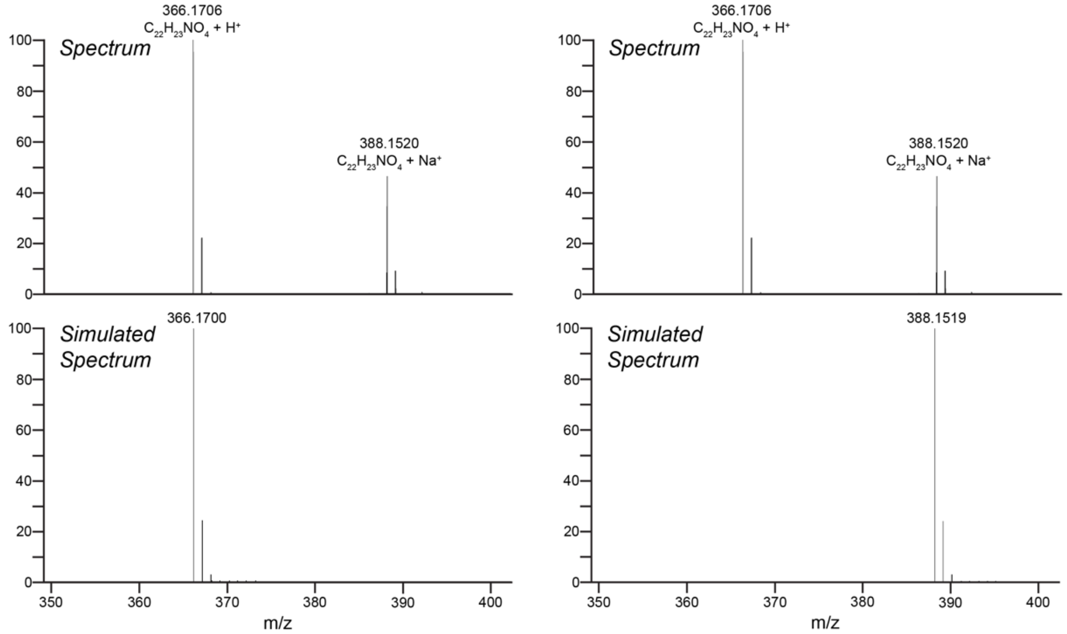


High-resolution mass spectra of 4-(4-(1,1-dimethylethyl)-phenoxyacetate)-2-quinolinemethanol (**3b**).

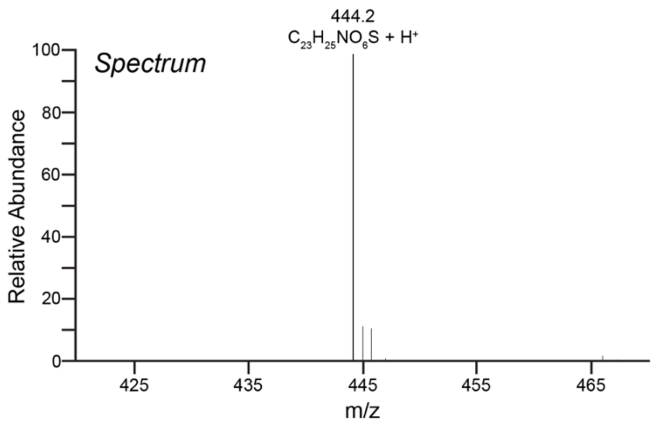


High-resolution mass spectra of 4-(4-(1,1-dimethylethyl)-phenoxyacetate)- 2-methanesulfonate-2-quinolinemethanol (**4b**).

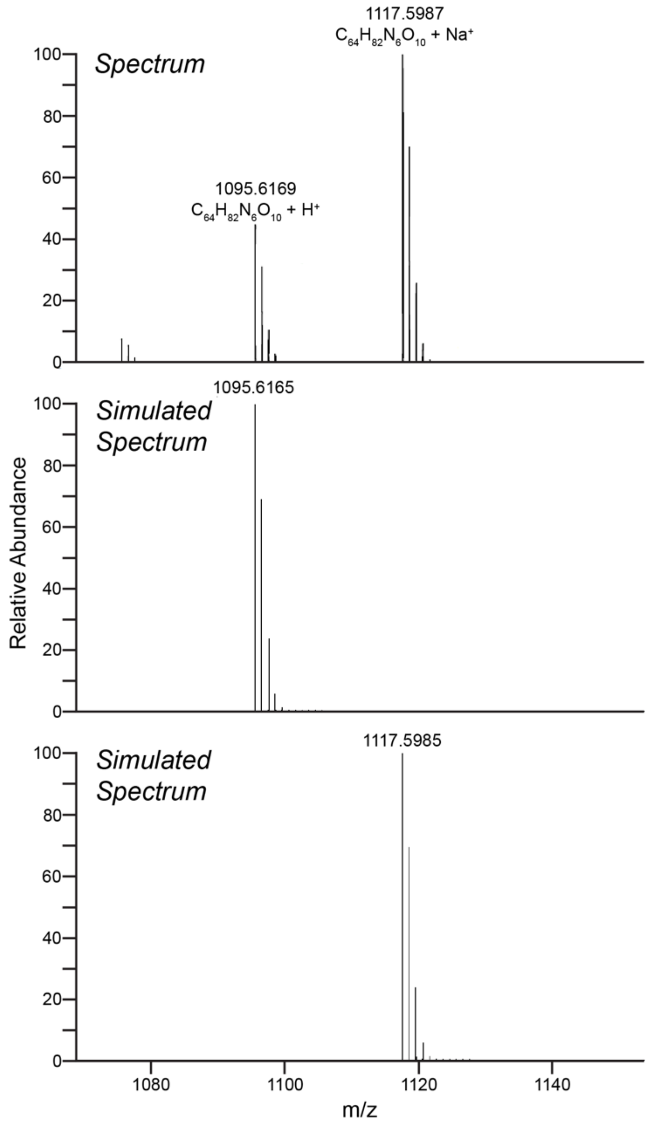


High-resolution mass spectra of 4,10-*bis*((4-(4-(1,1-dimethylethyl)-phenoxyacetate)-quinolin-2-yl)-methyl)-1,4,7,10-tetraazacyclododecane-1,7-diyl)-diacetate (**5b**).

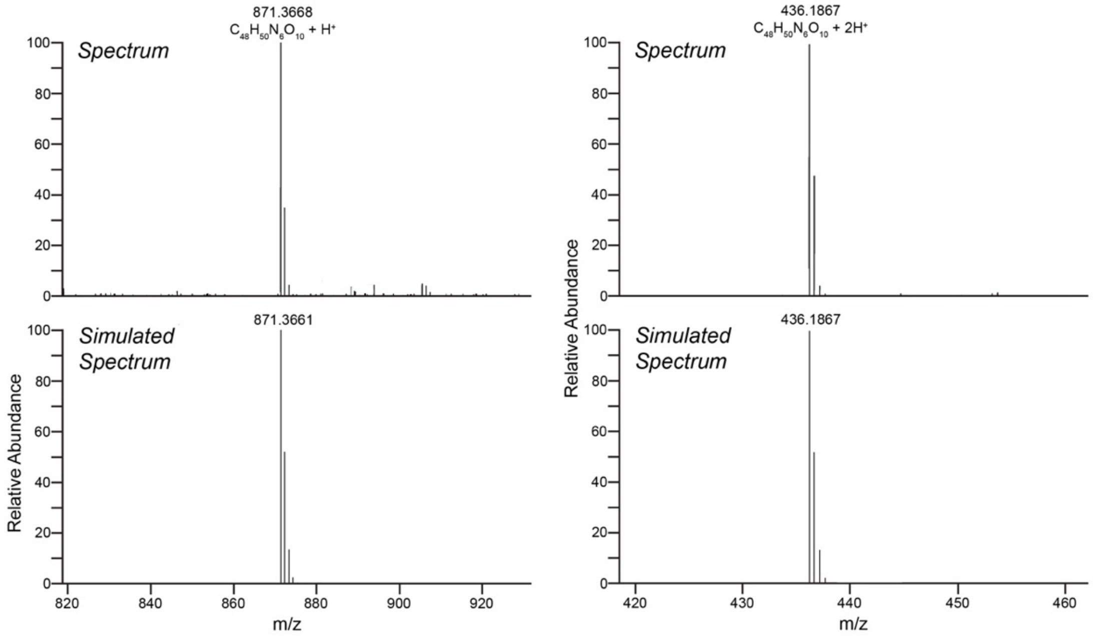


High-resolution mass spectra of 4,10-*bis*((4-(4-(1,1-dimethylethyl)-phenoxyacetate)-quinolin-2-yl)-methyl)-1,4,7,10-tetraazacyclododecane-1,7-diyl)-diacetic acid (**6b**).

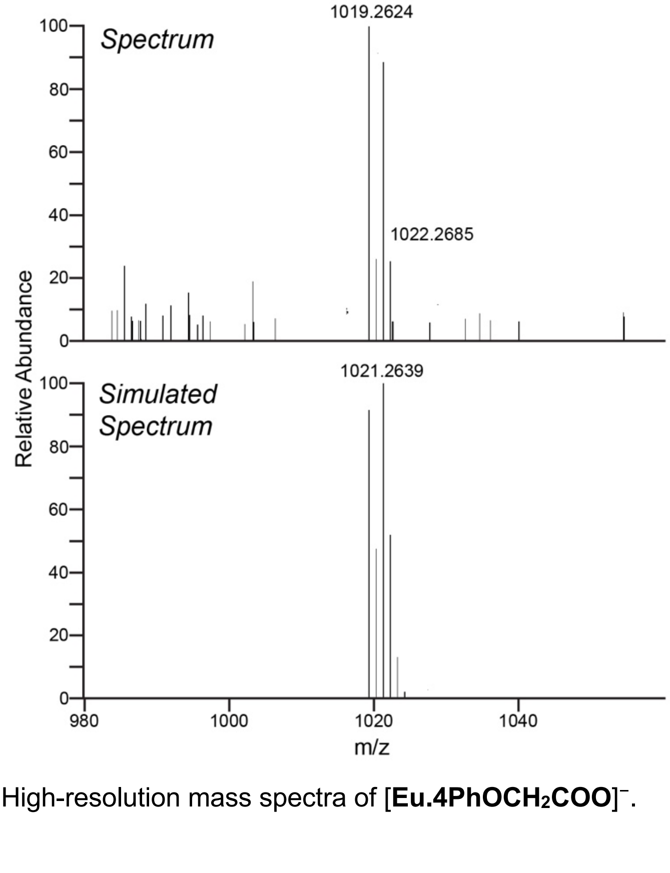


High-resolution mass spectra of [**Eu.4PhOCH$_2$COO**]$^-$.

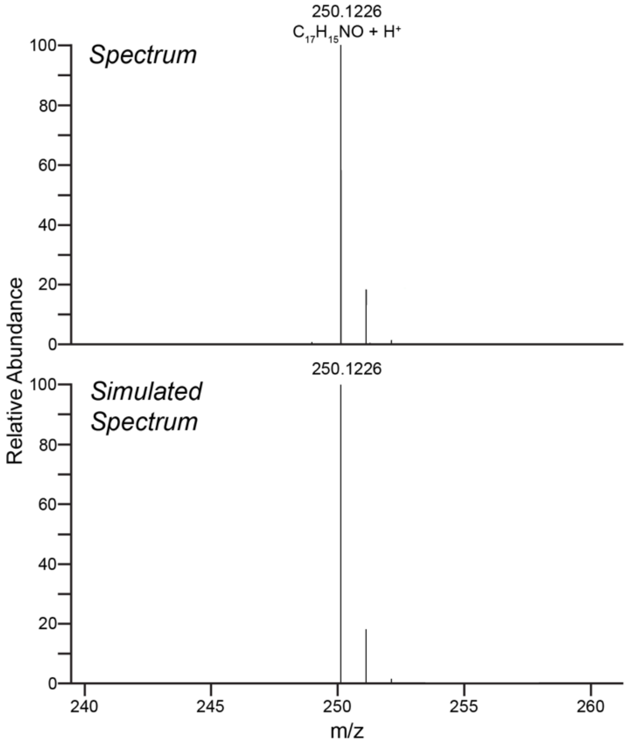


High-resolution mass spectra of 7-(4-methoxyphenyl)-2-methylquinoline (**1d**).

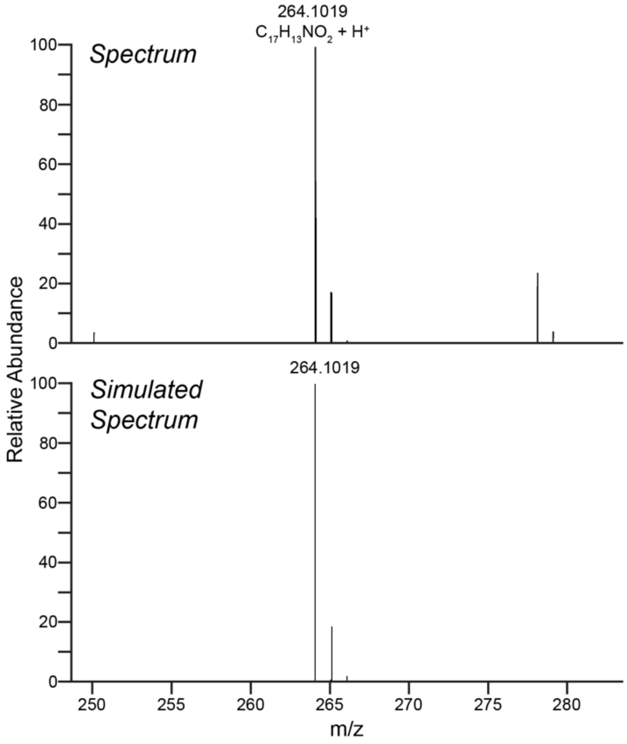


High-resolution mass spectra of 7-(4-methoxyphenyl)-2-quinolinecarboxaldehyde (**2c**).

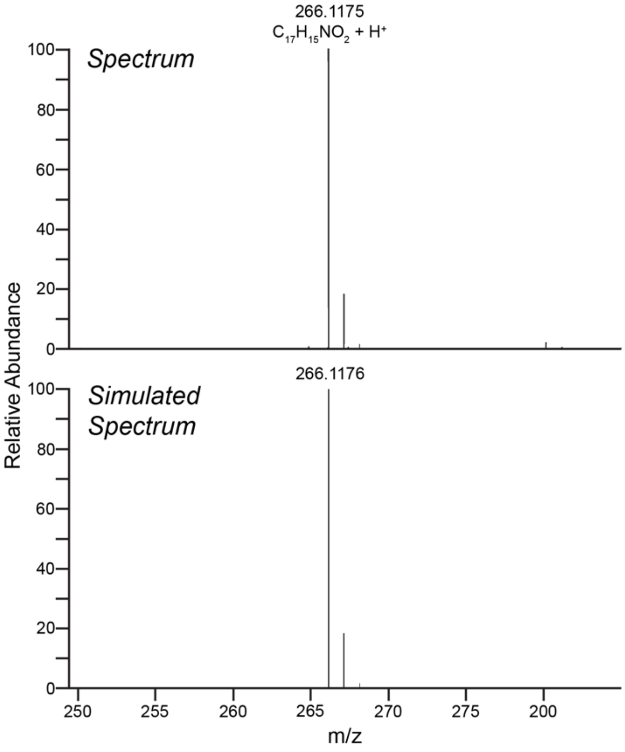


High-resolution mass spectra of 7-(4-methoxyphenyl)-2-quinolinemethanol (**3c**).

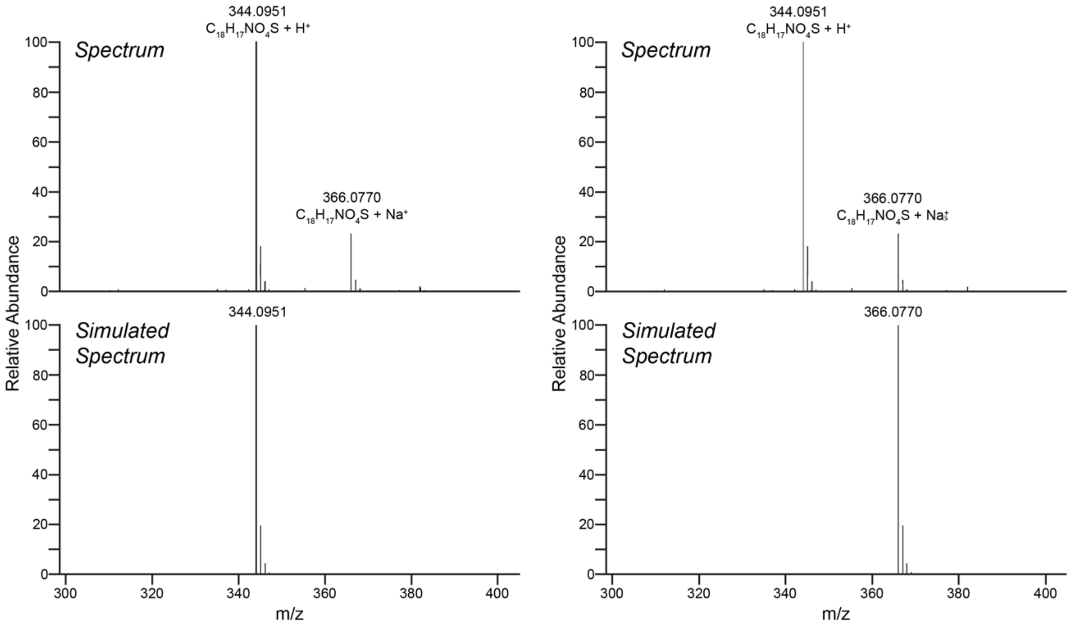


High-resolution mass spectra of 7-(4-methoxyphenyl)- 2-methanesulfonate-2-quinolinemethanol (**4c**).

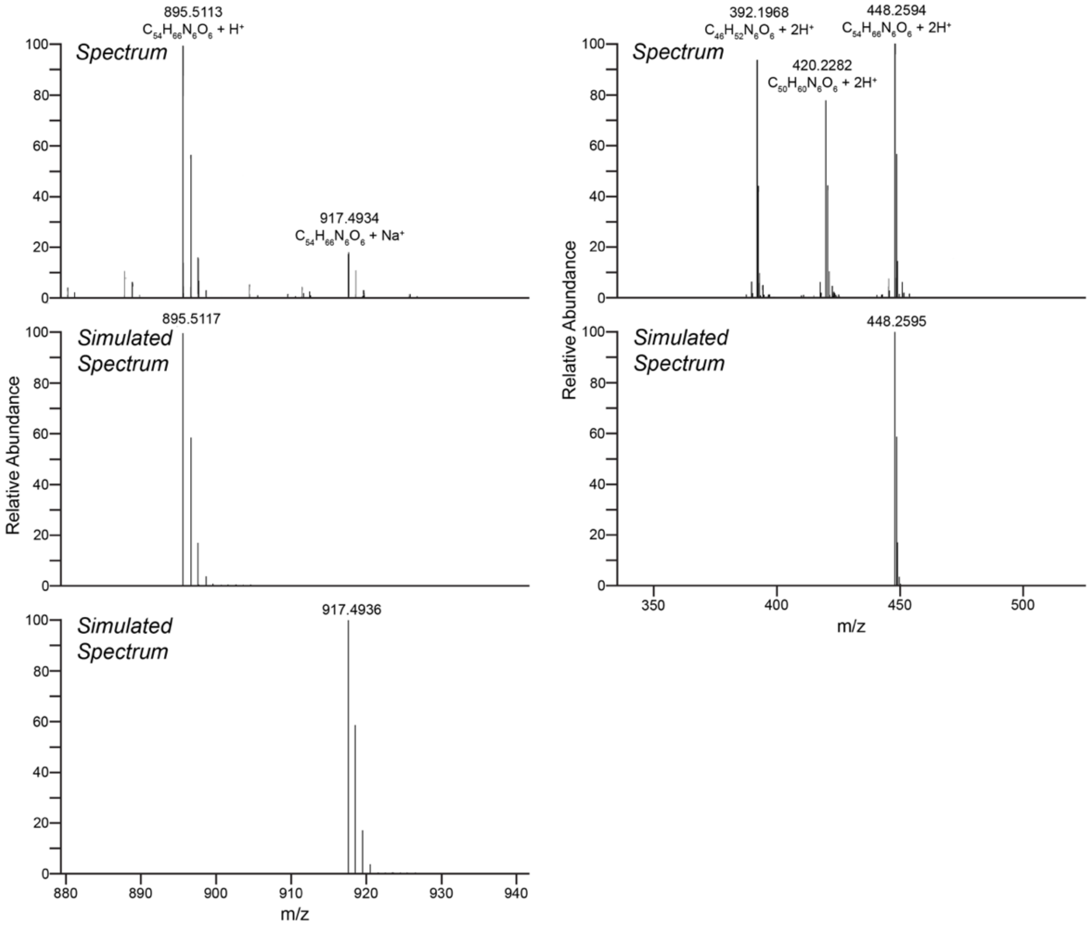


High-resolution mass spectra of 4,10-*bis*((7-(4-methoxyphenyl)-quinolin-2-yl)-methyl)-1,4,7,10-tetraazacyclododecane-1,7-diyl)-diacetate (**5c**).

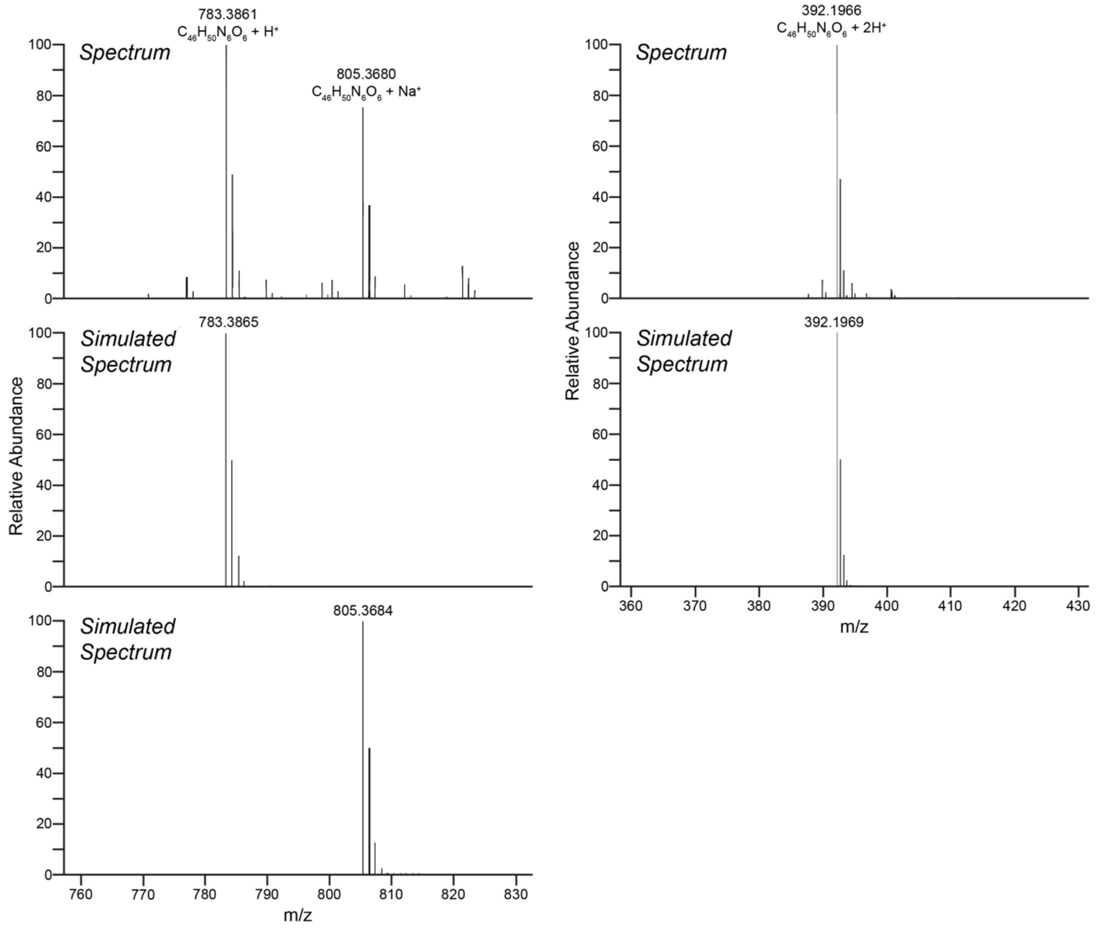


High-resolution mass spectra of 4,10-*bis*((7-(4-methoxyphenyl)-quinolin-2-yl)-methyl)-1,4,7,10-tetraazacyclododecane-1,7-diyl)-diacetic acid (**6c**).

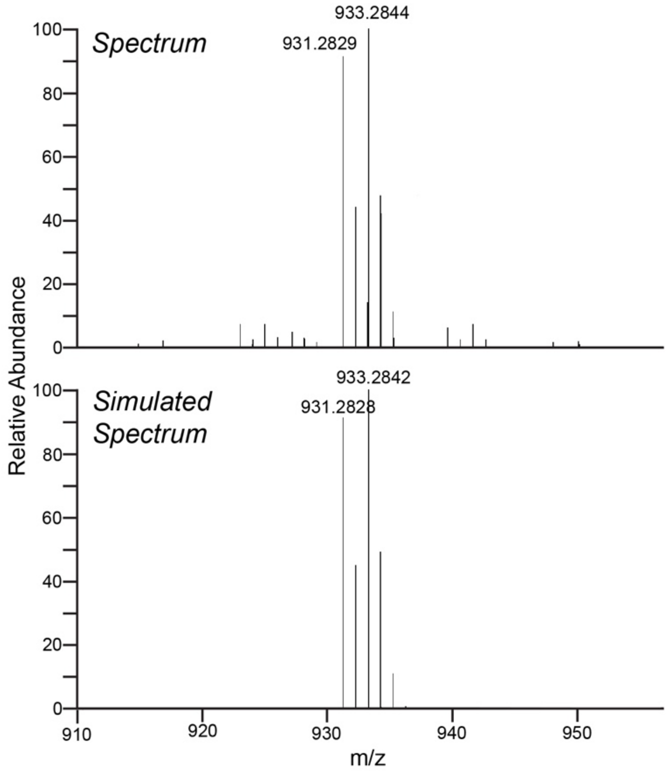


High-resolution mass spectra of [**Eu.7PhOMe**]$^{+}$.

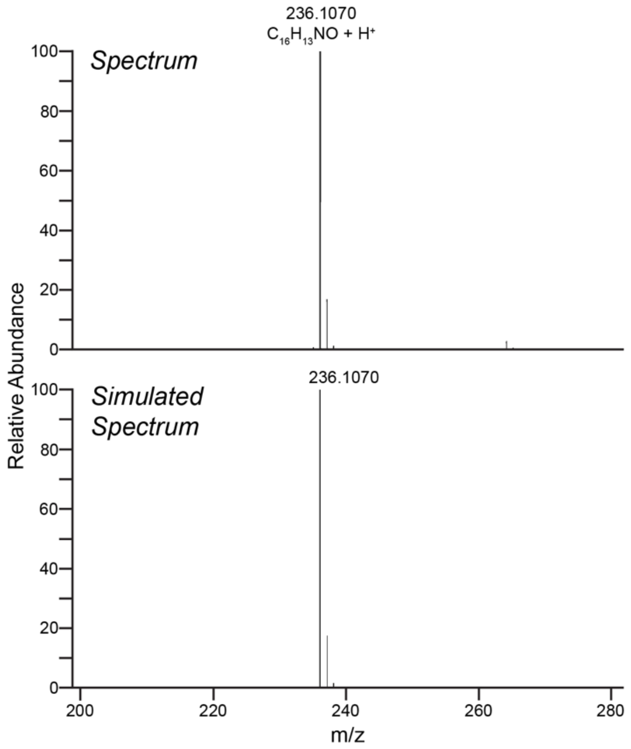


High-resolution mass spectra of 7-(2-methyl-4-quinolinyl)-phenol (**1e**).

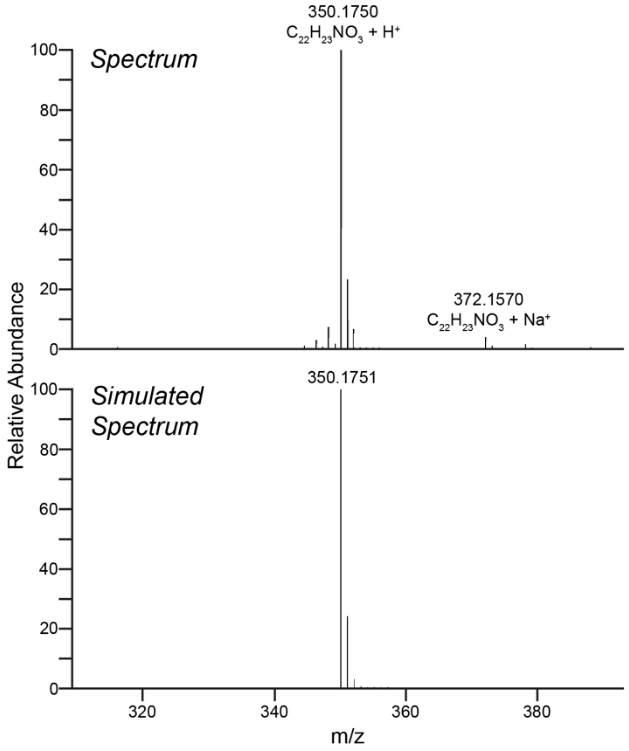


High-resolution mass spectra of 7-(4-(phenoxy)*tert*-butyl acetate)-2-methylquinoline or 4-(4-(1,1-dimethylethyl)-phenoxyacetate)-2-methylquinoline (**1f**).

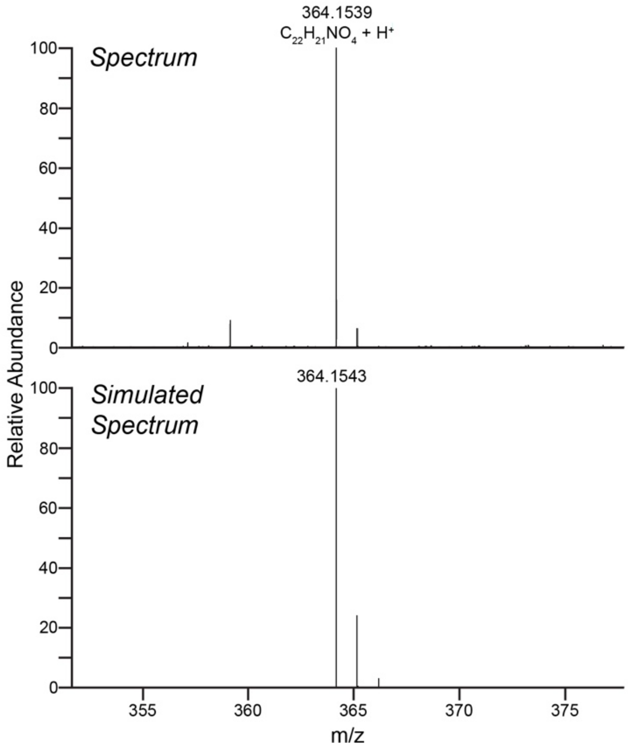


High-resolution mass spectra of 7-(4-(1,1-dimethylethyl)-phenoxyacetate)-2-quinolinecarboxaldehyde (**2d**).

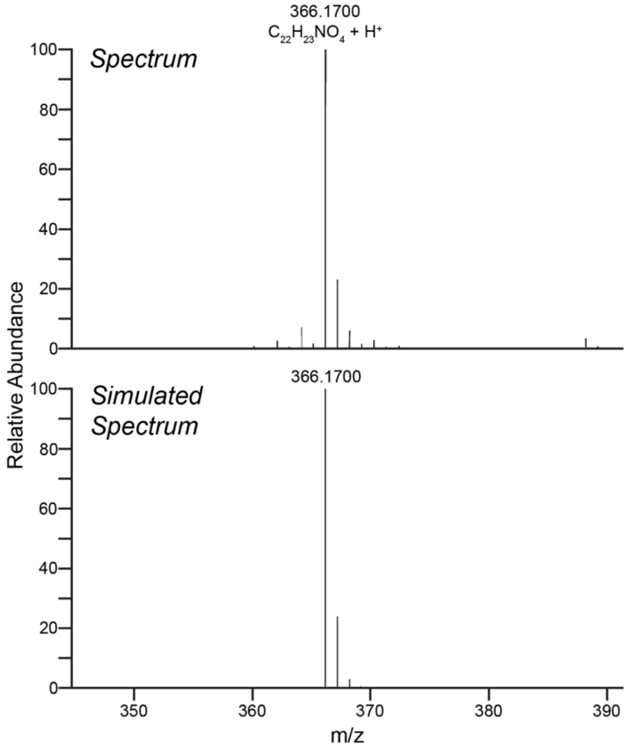


High-resolution mass spectra of 7-(4-(1,1-dimethylethyl)-phenoxyacetate)-2-quinolinemethanol (**3d**).

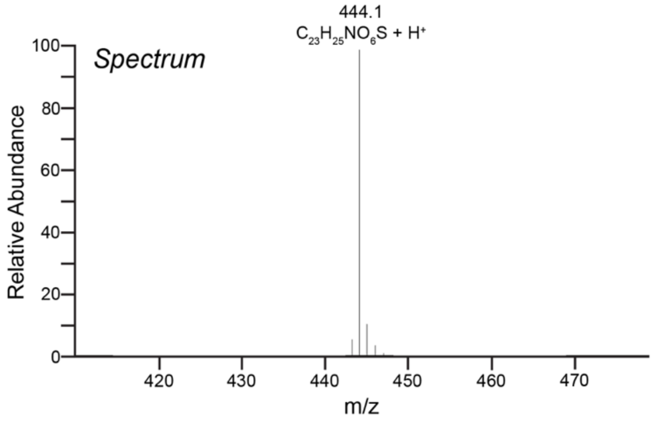


Low-resolution mass spectra of 7-(4-(1,1-dimethylethyl)-phenoxyacetate)-2-methanesulfonate-2-quinolinemethanol (**4d**).

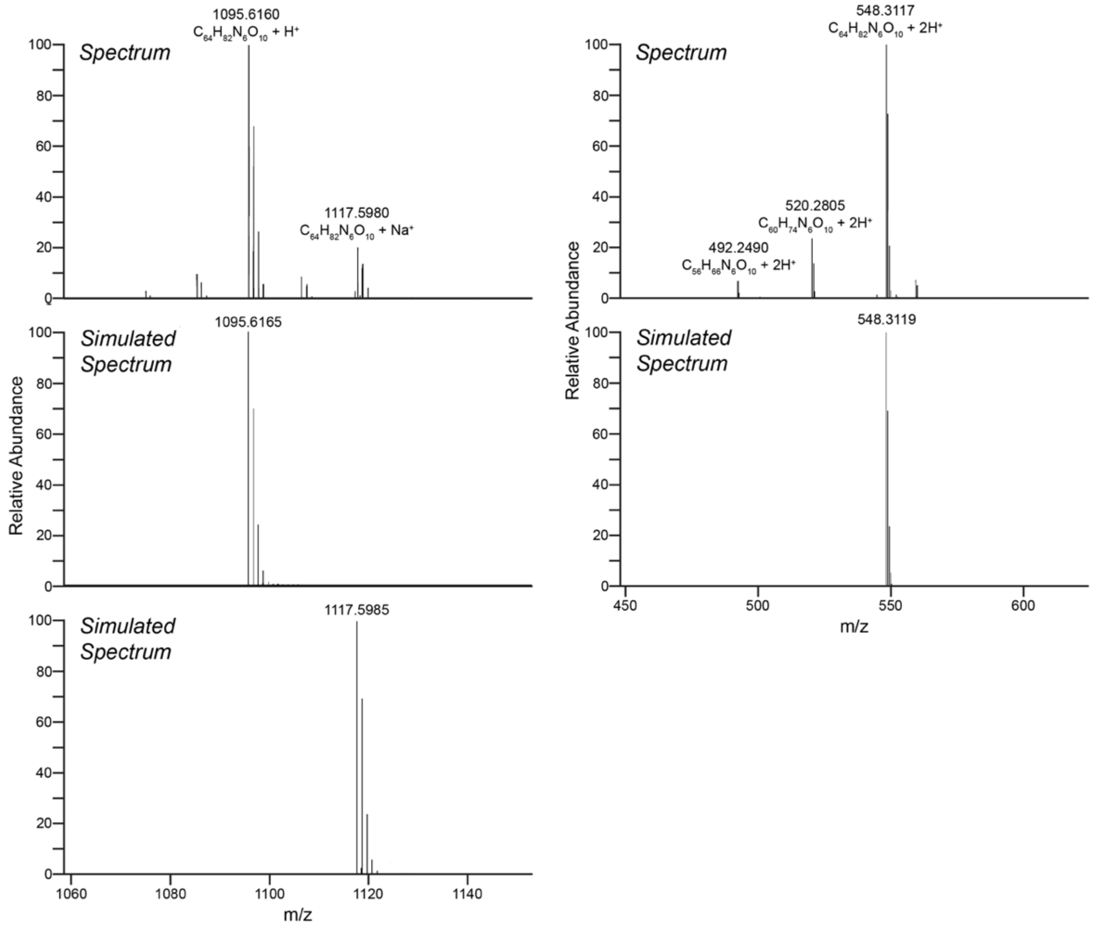


High-resolution mass spectra of 4,10-*bis*((7-(4-(1,1-dimethylethyl)-phenoxyacetate)-quinolin-2-yl)-methyl)-1,4,7,10-tetraazacyclododecane-1,7-diyl)-diacetate (**5d**).

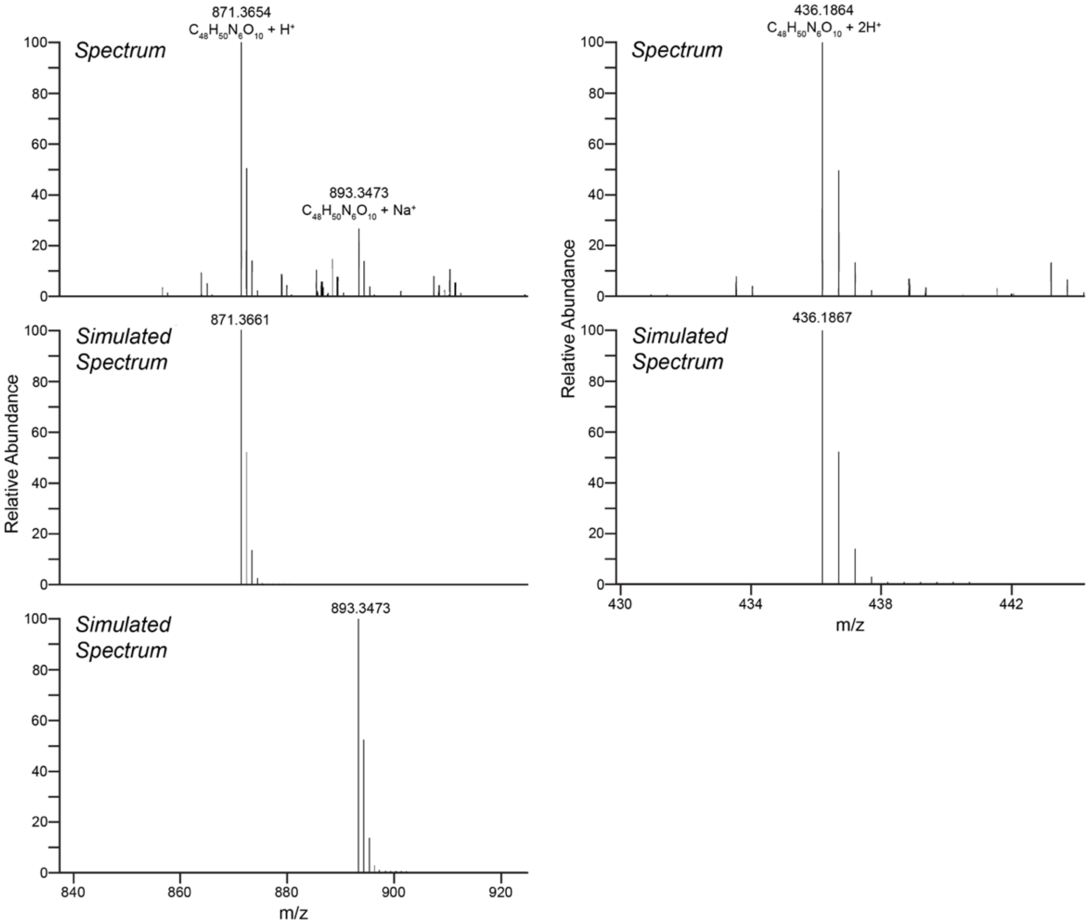


High-resolution mass spectra of 4,10-*bis*((7-(4-(1,1-dimethylethyl)-phenoxyacetate)-quinolin-2-yl)-methyl)-1,4,7,10-tetraazacyclododecane-1,7-diyl)-diacetic acid (**6d**).

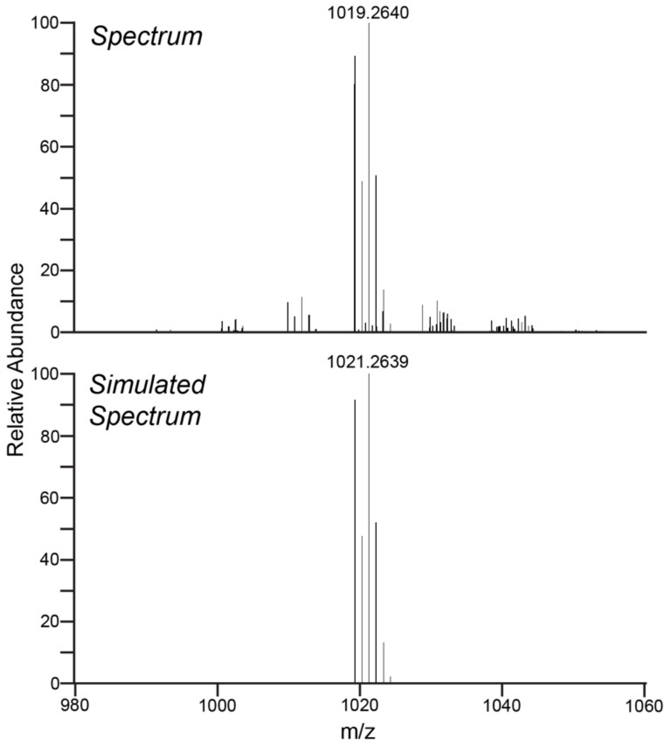


High-resolution mass spectra of [**Eu.7PhOCH₂COO**]$^{-}$.

## Appendix – X-ray Crystallography

## Complex [Eu.4PhOMe]$^+$

**Table S9.** Bond lengths for [**Eu.4PhOMe**]$^+$.

| Atom | Atom | Length/Å | Atom | Atom | Length/Å |
|---|---|---|---|---|---|
| Eu1 | O1 | 2.353(5) | C25 | C24 | 1.350(9) |
| Eu1 | O3 | 2.375(5) | C24 | C23 | 1.402(9) |
| Eu1 | N6 | 2.740(11) | C23 | C22 | 1.345(9) |
| Eu1 | N5 | 2.673(10) | C28 | C29 | 1.396(10) |
| Eu1 | N4 | 2.730(12) | C28 | C33 | 1.398(10) |
| Eu1 | N3 | 2.687(11) | C29 | C30 | 1.395(9) |
| Eu1 | N2 | 2.755(5) | C30 | C31 | 1.389(10) |
| Eu1 | N1 | 2.755(5) | C31 | C32 | 1.393(10) |
| Eu1 | C36 | 3.262(7) | C32 | C33 | 1.393(9) |
| Eu1 | O7 | 2.392(6) | C10 | C1 | 1.508(9) |
| O1 | C36 | 1.279(8) | C10 | N3A | 1.41(3) |
| O2 | C36 | 1.242(9) | C1 | C2 | 1.403(10) |
| O3 | C38 | 1.264(9) | C2 | C3 | 1.374(10) |
| O4 | C38 | 1.253(9) | C3 | C4 | 1.429(10) |
| O4 | Eu2 | 1.668(7) | C3 | C11 | 1.480(10) |
| O6 | C31 | 1.369(8) | C4 | C9 | 1.426(10) |
| O6 | C34 | 1.416(10) | C4 | C5 | 1.426(10) |
| O5 | C14 | 1.371(9) | C9 | C8 | 1.389(10) |
| O5 | C17 | 1.439(10) | C8 | C7 | 1.356(10) |
| N6 | C41 | 1.503(14) | C7 | C6 | 1.408(11) |
| N6 | C35 | 1.53(2) | C6 | C5 | 1.363(12) |
| N6 | C40 | 1.473(15) | C11 | C16 | 1.380(11) |
| N5 | C42 | 1.525(14) | C11 | C12 | 1.392(10) |
| N5 | C43 | 1.493(13) | C16 | C15 | 1.386(11) |
| N5 | C27 | 1.558(13) | C15 | C14 | 1.413(11) |
| N4 | C44 | 1.473(16) | C14 | C13 | 1.380(10) |
| N4 | C45 | 1.474(15) | C13 | C12 | 1.396(10) |
| N4 | C37 | 1.44(2) | C27 | N5A | 1.37(3) |
| N3 | C46 | 1.502(14) | C42A | C41A | 1.51(3) |
| N3 | C39 | 1.486(14) | C42A | N5A | 1.51(3) |
| N3 | C10 | 1.543(13) | C46A | C45A | 1.50(3) |
| N2 | C18 | 1.325(8) | C46A | N3A | 1.49(3) |
| N2 | C26 | 1.380(8) | C44A | C43A | 1.48(3) |
| N1 | C1 | 1.328(9) | C44A | N4A | 1.46(3) |
| N1 | C9 | 1.390(8) | C41A | N6A | 1.42(3) |
| C41 | C42 | 1.468(15) | C45A | N4A | 1.52(4) |
| C43 | C44 | 1.519(15) | C43A | N5A | 1.57(3) |
| C45 | C46 | 1.537(16) | N6A | C35A | 1.31(4) |
| C39 | C40 | 1.484(15) | N3A | C39A | 1.47(4) |
| C40A | N6A | 1.52(3) | N4A | C37A | 1.58(5) |
| C40A | C39A | 1.55(3) | C1B | F3 | 1.339(12) |
| C35 | C36 | 1.50(2) | C1B | F1 | 1.331(12) |

| Atom | Atom | Length/Å | Atom | Atom | Length/Å |
|---|---|---|---|---|---|
| C36 | C35A | 1.60(4) | C1B | F2 | 1.318(12) |
| C37 | C38 | 1.590(18) | C1B | S1 | 1.837(14) |
| C38 | Eu2 | 2.623(9) | S1 | O8 | 1.451(11) |
| C38 | C37A | 1.33(4) | S1 | O9 | 1.395(11) |
| C18 | C19 | 1.385(9) | S1 | O10 | 1.486(12) |
| C18 | C27 | 1.524(9) | C1A | F3A | 1.325(14) |
| C19 | C20 | 1.378(9) | C1A | F2A | 1.341(14) |
| C20 | C21 | 1.410(9) | C1A | F1A | 1.313(14) |
| C20 | C28 | 1.492(8) | C1A | S1A | 1.794(19) |
| C21 | C26 | 1.405(9) | S1A | O8A | 1.522(14) |
| C21 | C22 | 1.426(9) | S1A | O10A | 1.486(14) |
| C26 | C25 | 1.405(9) | S1A | O9A | 1.484(13) |

**Table S10.** Bond angles for [**Eu.4PhOMe**]$^{+}$.

| Atom | Atom | Atom | Angle/° | Atom | Atom | Atom | Angle/° |
|---|---|---|---|---|---|---|---|
| O1 | Eu1 | O3 | 147.12(19) | N2 | C18 | C19 | 124.4(6) |
| O1 | Eu1 | N6 | 62.9(2) | N2 | C18 | C27 | 118.7(6) |
| O1 | Eu1 | N5 | 121.2(2) | C19 | C18 | C27 | 116.9(6) |
| O1 | Eu1 | N4 | 145.7(3) | C20 | C19 | C18 | 120.4(6) |
| O1 | Eu1 | N3 | 80.5(2) | C19 | C20 | C21 | 117.0(6) |
| O1 | Eu1 | N2 | 88.34(16) | C19 | C20 | C28 | 119.9(6) |
| O1 | Eu1 | N1 | 80.70(16) | C21 | C20 | C28 | 123.1(6) |
| O1 | Eu1 | C36 | 18.69(18) | C20 | C21 | C22 | 122.9(6) |
| O1 | Eu1 | O7 | 74.09(17) | C26 | C21 | C20 | 119.1(6) |
| O3 | Eu1 | N6 | 145.6(3) | C26 | C21 | C22 | 117.9(6) |
| O3 | Eu1 | N5 | 81.4(3) | N2 | C26 | C21 | 122.5(6) |
| O3 | Eu1 | N4 | 62.4(3) | N2 | C26 | C25 | 118.5(5) |
| O3 | Eu1 | N3 | 121.5(2) | C21 | C26 | C25 | 119.0(6) |
| O3 | Eu1 | N2 | 82.29(17) | C24 | C25 | C26 | 121.1(6) |
| O3 | Eu1 | N1 | 88.85(17) | C25 | C24 | C23 | 120.7(6) |
| O3 | Eu1 | C36 | 154.53(17) | C22 | C23 | C24 | 119.5(6) |
| O3 | Eu1 | O7 | 73.04(18) | C23 | C22 | C21 | 121.7(6) |
| N6 | Eu1 | N2 | 83.3(2) | C29 | C28 | C20 | 119.3(6) |
| N6 | Eu1 | N1 | 119.8(2) | C29 | C28 | C33 | 119.0(6) |
| N6 | Eu1 | C36 | 46.9(3) | C33 | C28 | C20 | 121.8(6) |
| N5 | Eu1 | N6 | 64.2(3) | C30 | C29 | C28 | 120.6(7) |
| N5 | Eu1 | N4 | 66.3(3) | C31 | C30 | C29 | 119.1(7) |
| N5 | Eu1 | N3 | 100.0(3) | O6 | C31 | C30 | 115.4(7) |
| N5 | Eu1 | N2 | 61.7(2) | O6 | C31 | C32 | 122.9(7) |
| N5 | Eu1 | N1 | 150.5(2) | C30 | C31 | C32 | 121.6(6) |
| N5 | Eu1 | C36 | 102.5(3) | C33 | C32 | C31 | 118.3(7) |
| N4 | Eu1 | N6 | 99.4(4) | C32 | C33 | C28 | 121.4(7) |
| N4 | Eu1 | N2 | 120.2(3) | C1 | C10 | N4 | 110.6(7) |
| N4 | Eu1 | N1 | 84.5(2) | N3A | C10 | C1 | 120.6(13) |
| N4 | Eu1 | C36 | 142.4(3) | N1 | C1 | C10 | 119.3(6) |
| N3 | Eu1 | N6 | 66.0(3) | N1 | C1 | C2 | 124.6(6) |
| N3 | Eu1 | N5 | 65.2(3) | C2 | C1 | C10 | 116.1(6) |

| Atom | Atom | Atom | Angle/˚ | Atom | Atom | Atom | Angle/˚ |
|---|---|---|---|---|---|---|---|
| N3 | Eu1 | N2 | 149.1(2) | C3 | C2 | C1 | 120.5(6) |
| N3 | Eu1 | N1 | 61.9(2) | C2 | C3 | C4 | 117.0(6) |
| N3 | Eu1 | C36 | 83.0(2) | C2 | C3 | C11 | 120.6(7) |
| N2 | Eu1 | N1 | 144.50(16) | C4 | C3 | C11 | 122.3(6) |
| N2 | Eu1 | C36 | 77.82(17) | C9 | C4 | C3 | 119.4(6) |
| N1 | Eu1 | C36 | 98.15(18) | C9 | C4 | C5 | 118.7(7) |
| O7 | Eu1 | N6 | 130.6(2) | C5 | C4 | C3 | 121.8(7) |
| O7 | Eu1 | N5 | 129.2(3) | N1 | C9 | C4 | 121.6(6) |
| O7 | Eu1 | N4 | 130.0(3) | C8 | C9 | N1 | 119.4(6) |
| O7 | Eu1 | N3 | 130.8(3) | C8 | C9 | C4 | 119.0(6) |
| O7 | Eu1 | N2 | 71.78(16) | C7 | C8 | C9 | 121.7(7) |
| O7 | Eu1 | N1 | 72.75(16) | C8 | C7 | C6 | 119.7(7) |
| O7 | Eu1 | C36 | 85.64(17) | C5 | C6 | C7 | 121.2(7) |
| C36 | O1 | Eu1 | 125.2(5) | C6 | C5 | C4 | 119.5(8) |
| C38 | O3 | Eu1 | 125.3(5) | C16 | C11 | C3 | 121.2(7) |
| C38 | O4 | Eu2 | 127.2(6) | C16 | C11 | C12 | 118.6(7) |
| C31 | O6 | C34 | 118.3(6) | C12 | C11 | C3 | 120.1(7) |
| C14 | O5 | C17 | 116.4(6) | C11 | C16 | C15 | 121.5(7) |
| C41 | N6 | Eu1 | 116.4(7) | C16 | C15 | C14 | 118.9(7) |
| C41 | N6 | C35 | 111.1(12) | O5 | C14 | C15 | 114.9(7) |
| C35 | N6 | Eu1 | 103.0(10) | O5 | C14 | C13 | 124.5(7) |
| C40 | N6 | Eu1 | 111.0(7) | C13 | C14 | C15 | 120.6(7) |
| C40 | N6 | C41 | 108.4(9) | C14 | C13 | C12 | 118.8(7) |
| C40 | N6 | C35 | 106.5(11) | C11 | C12 | C13 | 121.6(7) |
| C42 | N5 | Eu1 | 112.6(7) | O4 | Eu2 | C38 | 22.4(3) |
| C42 | N5 | C27 | 111.7(8) | C18 | C27 | N5 | 108.8(6) |
| C43 | N5 | Eu1 | 113.4(6) | N5A | C27 | C18 | 118.1(12) |
| C43 | N5 | C42 | 108.2(9) | N5A | C42A | C41A | 115(2) |
| C43 | N5 | C27 | 107.4(8) | N3A | C46A | C45A | 116(2) |
| C27 | N5 | Eu1 | 103.4(5) | N4A | C44A | C43A | 115(2) |
| C44 | N4 | Eu1 | 111.3(7) | N6A | C41A | C42A | 111(2) |
| C44 | N4 | C45 | 108.9(9) | C46A | C45A | N4A | 111(2) |
| C45 | N4 | Eu1 | 115.9(8) | C44A | C43A | N5A | 111(2) |
| C37 | N4 | Eu1 | 102.7(10) | N6 | C40 | C39 | 111.5(10) |
| C37 | N4 | C44 | 110.4(14) | C27 | N5A | C42A | 97.6(18) |
| C37 | N4 | C45 | 107.5(12) | C27 | N5A | C43A | 109(2) |
| C46 | N3 | Eu1 | 111.9(7) | C42A | N5A | C43A | 109(2) |
| C46 | N3 | C10 | 110.8(8) | C41A | N6A | C40A | 107(2) |
| C39 | N3 | Eu1 | 111.9(6) | C35A | N6A | C40A | 114(3) |
| C39 | N3 | C46 | 109.3(9) | C35A | N6A | C41A | 107(3) |
| C39 | N3 | C10 | 109.5(8) | C10 | N3A | C46A | 100.4(18) |
| C10 | N3 | Eu1 | 103.3(5) | C10 | N3A | C39A | 110(2) |
| C18 | N2 | Eu1 | 113.5(4) | C39A | N3A | C46A | 109(2) |
| C18 | N2 | C26 | 116.2(5) | C44A | N4A | C45A | 109(2) |
| C26 | N2 | Eu1 | 129.8(4) | C44A | N4A | C37A | 105(3) |
| C1 | N1 | Eu1 | 112.6(4) | C45A | N4A | C37A | 118(3) |
| C1 | N1 | C9 | 116.8(6) | N3A | C39A | C40A | 110(2) |
| C9 | N1 | Eu1 | 128.5(4) | F3 | C1B | S1 | 107.8(10) |

| Atom | Atom | Atom | Angle/° | Atom | Atom | Atom | Angle/° |
|---|---|---|---|---|---|---|---|
| C42 | C41 | N6 | 109.3(8) | F1 | C1B | F3 | 110.9(15) |
| C41 | C42 | N5 | 112.8(9) | F1 | C1B | S1 | 104.1(13) |
| N5 | C43 | C44 | 112.2(9) | F2 | C1B | F3 | 110.0(14) |
| N4 | C44 | C43 | 111.7(9) | F2 | C1B | F1 | 109.5(16) |
| N4 | C45 | C46 | 110.5(9) | F2 | C1B | S1 | 114.4(13) |
| N3 | C46 | C45 | 111.6(9) | O8 | S1 | C1B | 99.2(9) |
| C40 | C39 | N3 | 113.2(9) | O8 | S1 | O10 | 113.8(12) |
| N6A | C40A | C39A | 109.8(19) | O9 | S1 | C1B | 103.7(9) |
| C36 | C35 | N7 | 107.1(15) | O9 | S1 | O8 | 123.6(13) |
| O1 | C36 | Eu1 | 36.1(3) | O9 | S1 | O10 | 113.7(13) |
| O1 | C36 | C35 | 118.7(9) | O10 | S1 | C1B | 96.7(10) |
| O1 | C36 | C35A | 110.1(15) | F3A | C1A | F2A | 114.1(19) |
| O2 | C36 | Eu1 | 159.8(5) | F3A | C1A | S1A | 97.7(16) |
| O2 | C36 | O1 | 124.8(7) | F2A | C1A | S1A | 97.9(18) |
| O2 | C36 | C35 | 116.5(9) | F1A | C1A | F3A | 118(2) |
| O2 | C36 | C35A | 124.7(15) | F1A | C1A | F2A | 117(2) |
| C35 | C36 | Eu1 | 83.1(7) | F1A | C1A | S1A | 107(2) |
| N4 | C37 | C38 | 105.8(16) | O8A | S1A | C1A | 101.4(16) |
| O3 | C38 | C37 | 115.1(10) | O10A | S1A | C1A | 98.8(15) |
| O3 | C38 | Eu2 | 152.7(5) | O10A | S1A | O8A | 115.3(18) |
| O3 | C38 | C37A | 121(2) | O9A | S1A | C1A | 107.9(16) |
| O4 | C38 | O3 | 124.5(7) | O9A | S1A | O8A | 111.8(17) |
| O4 | C38 | C37 | 120.3(10) | O9A | S1A | O10A | 118.8(18) |
| O4 | C38 | Eu2 | 30.4(4) | C38 | C37A | N4A | 116(3) |
| O4 | C38 | C37A | 114(2) | N6A | C35A | C36 | 119(3) |

**Table S11.** Torsion angles for [**Eu.4PhOMe**]$^{+}$.

| A | B | C | D | Angle/° | A | B | C | D | Angle/° |
|---|---|---|---|---|---|---|---|---|---|
| Eu1 | O1 | C36 | O2 | 170.7(6) | C29 | C28 | C33 | C32 | 0.8(9) |
| Eu1 | O1 | C36 | C35 | -10.4(13) | C29 | C30 | C31 | O6 | -177.9(6) |
| Eu1 | O3 | C38 | O4 | 175.8(6) | C29 | C30 | C31 | C32 | -0.5(10) |
| Eu1 | O3 | C38 | C37 | -5.5(12) | C30 | C31 | C32 | C33 | -0.3(10) |
| Eu1 | N6 | C41 | C42 | 34.2(10) | C31 | C32 | C33 | C28 | 0.1(10) |
| Eu1 | N6 | C35 | C36 | 46.9(13) | C33 | C28 | C29 | C30 | -1.6(10) |
| Eu1 | N6 | C40 | C39 | 44.6(11) | C34 | O6 | C31 | C30 | 177.1(7) |
| Eu1 | N5 | C42 | C41 | 50.7(10) | C34 | O6 | C31 | C32 | -0.3(10) |
| Eu1 | N5 | C43 | C44 | 40.5(11) | C10 | N3 | C46 | C45 | 165.1(8) |
| Eu1 | N5 | C27 | C18 | 60.4(7) | C10 | N3 | C39 | C40 | -70.7(11) |
| Eu1 | N4 | C44 | C43 | 45.0(11) | C10 | C1 | C2 | C3 | -178.5(7) |
| Eu1 | N4 | C45 | C46 | 33.6(10) | C10 | N3A | C39A | C40A | -167.9(18) |
| Eu1 | N4 | C37 | C38 | 53.7(14) | C1 | N1 | C9 | C4 | -0.4(9) |
| Eu1 | N3 | C46 | C45 | 50.4(10) | C1 | N1 | C9 | C8 | -179.7(6) |
| Eu1 | N3 | C39 | C40 | 43.2(11) | C1 | C10 | N3A | C46A | -89.1(19) |
| Eu1 | N3 | C10 | C1 | 58.0(7) | C1 | C10 | N3A | C39A | 156.3(16) |
| Eu1 | N2 | C18 | C19 | 170.1(5) | C1 | C2 | C3 | C4 | -1.7(11) |
| Eu1 | N2 | C18 | C27 | -11.6(8) | C1 | C2 | C3 | C11 | 175.0(7) |
| Eu1 | N2 | C26 | C21 | -174.8(4) | C2 | C3 | C4 | C9 | 1.9(10) |

| A | B | C | D | Angle/° | A | B | C | D | Angle/° |
|---|---|---|---|---|---|---|---|---|---|
| Eu1 | N2 | C26 | C25 | 5.7(8) | C2 | C3 | C4 | C5 | -175.6(7) |
| Eu1 | N1 | C1 | C10 | -15.2(8) | C2 | C3 | C11 | C16 | 55.5(10) |
| Eu1 | N1 | C1 | C2 | 165.8(5) | C2 | C3 | C11 | C12 | -120.7(8) |
| Eu1 | N1 | C9 | C4 | -162.8(5) | C3 | C4 | C9 | N1 | -0.9(10) |
| Eu1 | N1 | C9 | C8 | 17.9(8) | C3 | C4 | C9 | C8 | 178.4(6) |
| O1 | C36 | C35A | N6A | -27(4) | C3 | C4 | C5 | C6 | -179.9(8) |
| O2 | C36 | C35A | N6A | 160(3) | C3 | C11 | C16 | C15 | -177.0(7) |
| O3 | C38 | C37A | N4A | -19(4) | C3 | C11 | C12 | C13 | 175.2(7) |
| O4 | C38 | C37A | N4A | 162(2) | C4 | C3 | C11 | C16 | -127.9(8) |
| O6 | C31 | C32 | C33 | 176.9(6) | C4 | C3 | C11 | C12 | 55.8(10) |
| O5 | C14 | C13 | C12 | -178.4(7) | C4 | C9 | C8 | C7 | 1.9(10) |
| N6 | C41 | C42 | N6 | -55.9(12) | C9 | N1 | C1 | C10 | 179.6(6) |
| N6 | C35 | C36 | Eu1 | -37.0(10) | C9 | N1 | C1 | C2 | 0.6(10) |
| N6 | C35 | C36 | O1 | -30.8(17) | C9 | C4 | C5 | C6 | 2.6(12) |
| N6 | C35 | C36 | O2 | 148.1(10) | C9 | C8 | C7 | C6 | 1.6(11) |
| N5 | C43 | C44 | N4 | -58.8(13) | C8 | C7 | C6 | C5 | -3.1(12) |
| N4 | C45 | C46 | N3 | -56.4(12) | C7 | C6 | C5 | C4 | 0.9(13) |
| N4 | C37 | C38 | O3 | -38.6(18) | C5 | C4 | C9 | N1 | 176.7(7) |
| N4 | C37 | C38 | O4 | 140.1(12) | C5 | C4 | C9 | C8 | -4.0(10) |
| N3 | C39 | C40 | N6 | -60.9(13) | C11 | C3 | C4 | C9 | -174.8(7) |
| N3 | C10 | C1 | N1 | -29.9(9) | C11 | C3 | C4 | C5 | 7.7(11) |
| N3 | C10 | C1 | C2 | 149.2(7) | C11 | C16 | C15 | C14 | 2.0(11) |
| N2 | C18 | C19 | C20 | 5.3(11) | C16 | C11 | C12 | C13 | -1.1(11) |
| N2 | C18 | C27 | N5 | -33.7(9) | C16 | C15 | C14 | O5 | 176.9(7) |
| N2 | C18 | C27 | N5A | -12.6(16) | C16 | C15 | C14 | C13 | -1.6(11) |
| N2 | C26 | C25 | C24 | 178.4(6) | C15 | C14 | C13 | C12 | -0.2(11) |
| N1 | C1 | C2 | C3 | 0.5(11) | C14 | C13 | C12 | C11 | 1.5(11) |
| N1 | C9 | C8 | C7 | -178.8(6) | C12 | C11 | C16 | C15 | -0.7(11) |
| C41 | N6 | C35 | C36 | 172.2(10) | C17 | O5 | C14 | C15 | 173.8(7) |
| C41 | N6 | C40 | C39 | -84.3(12) | C17 | O5 | C14 | C13 | -7.9(11) |
| C42 | N5 | C43 | C44 | 166.2(9) | Eu2 | O4 | C38 | O3 | 163.5(6) |
| C42 | N5 | C27 | C18 | -60.9(10) | C27 | N5 | C42 | C41 | 166.5(8) |
| C43 | N5 | C42 | C41 | -75.4(11) | C27 | N5 | C43 | C44 | -73.1(11) |
| C43 | N5 | C27 | C18 | -179.4(7) | C27 | C18 | C19 | C20 | -173.0(7) |
| C44 | N4 | C45 | C46 | 160.0(9) | C42A | C41A | N6A | C40A | 76(3) |
| C44 | N4 | C37 | C38 | -65.0(16) | C42A | C41A | N6A | C35A | -161(3) |
| C45 | N4 | C44 | C43 | -84.0(11) | C46A | C45A | N4A | C44A | 75(3) |
| C45 | N4 | C37 | C38 | 176.4(12) | C46A | C45A | N4A | C37A | -165(2) |
| C46 | N3 | C39 | C40 | 167.8(10) | C46A | N3A | C39A | C40A | 83(3) |
| C46 | N3 | C10 | C1 | -62.0(10) | C44A | C43A | N5A | C27 | -167.3(19) |
| C39 | N3 | C46 | C45 | -74.2(11) | C44A | C43A | N5A | C42A | 87(3) |
| C39 | N3 | C10 | C1 | 177.4(7) | C44A | N4A | C37A | C38 | -87(3) |
| C40A | N6A | C35A | C36 | -82(4) | C41A | C42A | N5A | C27 | 92(2) |
| C35 | N6 | C41 | C42 | -83.2(13) | C41A | C42A | N5A | C43A | -155(2) |
| C35 | N6 | C40 | C39 | 156.0(13) | C41A | N6A | C35A | C36 | 160(3) |
| C37 | N4 | C44 | C43 | 158.3(12) | C45A | C46A | N3A | C10 | 86(2) |
| C37 | N4 | C45 | C46 | -80.5(15) | C45A | C46A | N3A | C39A | -158(2) |
| C18 | N2 | C26 | C21 | -3.5(9) | C45A | N4A | C37A | C38 | 151(3) |

| A | B | C | D | Angle/˚ | A | B | C | D | Angle/˚ |
|---|---|---|---|---|---|---|---|---|---|
| C18 | N2 | C26 | C25 | 177.0(6) | C43A | C44A | N4A | C45A | -168(2) |
| C18 | C19 | C20 | C21 | -1.6(10) | C43A | C44A | N4A | C37A | 65(3) |
| C18 | C19 | C20 | C28 | -179.9(6) | C40 | N6 | C41 | C42 | 160.1(9) |
| C18 | C27 | N5A | C42A | -87.9(18) | C40 | N6 | C35 | C36 | -70.0(14) |
| C18 | C27 | N5A | C43A | 158.7(14) | N5A | C42A | C41A | N6A | 50(3) |
| C19 | C18 | C27 | N6 | 144.8(7) | N6A | C40A | C39A | N3A | 64(3) |
| C19 | C18 | C27 | N5A | 165.9(13) | N3A | C10 | C1 | N1 | -9.5(15) |
| C19 | C20 | C21 | C26 | -4.2(9) | N3A | C10 | C1 | C2 | 169.5(13) |
| C19 | C20 | C21 | C22 | 179.8(6) | N3A | C46A | C45A | N4A | 53(3) |
| C19 | C20 | C28 | C29 | 56.3(9) | N4A | C44A | C43A | N5A | 60(3) |
| C19 | C20 | C28 | C33 | -123.2(7) | C39A | C40A | N6A | C41A | -174(2) |
| C20 | C21 | C26 | N2 | 7.0(9) | C39A | C40A | N6A | C35A | 68(3) |
| C20 | C21 | C26 | C25 | -173.5(6) | F3 | C1B | S1 | O8 | -70.9(14) |
| C20 | C21 | C22 | C23 | 173.7(7) | F3 | C1B | S1 | O9 | 57.2(16) |
| C20 | C28 | C29 | C30 | 178.9(6) | F3 | C1B | S1 | O10 | 173.6(14) |
| C20 | C28 | C33 | C32 | -179.7(6) | F1 | C1B | S1 | O8 | 171.3(15) |
| C21 | C20 | C28 | C29 | -121.9(7) | F1 | C1B | S1 | O9 | -60.6(17) |
| C21 | C20 | C28 | C33 | 58.6(9) | F1 | C1B | S1 | O10 | 55.8(16) |
| C21 | C26 | C25 | C24 | -1.1(9) | F2 | C1B | S1 | O8 | 51.8(17) |
| C26 | N2 | C18 | C19 | -2.7(10) | F2 | C1B | S1 | O9 | 179.9(17) |
| C26 | N2 | C18 | C27 | 175.6(6) | F2 | C1B | S1 | O10 | -63.6(17) |
| C26 | C21 | C22 | C23 | -2.4(10) | F3A | C1A | S1A | O8A | -59(2) |
| C26 | C25 | C24 | C23 | -0.9(10) | F3A | C1A | S1A | O10A | 59.6(19) |
| C25 | C24 | C23 | C22 | 1.3(11) | F3A | C1A | S1A | O9A | -176.2(19) |
| C24 | C23 | C22 | C21 | 0.4(11) | F2A | C1A | S1A | O8A | -174(2) |
| C22 | C21 | C26 | N2 | -176.7(6) | F2A | C1A | S1A | O10A | -56(2) |
| C22 | C21 | C26 | C25 | 2.7(9) | F2A | C1A | S1A | O9A | 68(2) |
| C28 | C20 | C21 | C26 | 174.1(6) | F1A | C1A | S1A | O8A | 64(2) |
| C28 | C20 | C21 | C22 | -2.0(10) | F1A | C1A | S1A | O10A | -178(2) |
| C28 | C29 | C30 | C31 | 1.4(10) | F1A | C1A | S1A | O9A | -53(2) |

**Table S12.** Fractional Atomic Coordinates and Equivalent Isotropic Displacement Parameters (Å$^2$) for [**Eu.4PhOMe**]$^+$.

| Atom | *x* | *y* | *z* | U(eq) |
|---|---|---|---|---|
| Eu1 | 4278.7(2) | 3154.6(2) | 4229.1(2) | 31.03(15) |
| O1 | 3530(3) | 3400.6(19) | 2812(3) | 44.9(11) |
| O2 | 2240(4) | 3420(2) | 1746(4) | 63.2(15) |
| O3 | 5043(4) | 3430(2) | 5641(3) | 50.3(12) |
| O4 | 6288(4) | 3439(3) | 6744(4) | 70.0(17) |
| O6 | -1654(4) | 4502(2) | 8127(3) | 53.5(13) |
| O5 | 10038(4) | 4356(2) | 32(4) | 61.6(14) |
| N6 | 3008(8) | 2453(4) | 3327(7) | 36(2) |
| N5 | 3387(7) | 2493(4) | 5228(7) | 33(2) |
| N4 | 5552(8) | 2478(4) | 5174(9) | 39(3) |
| N3 | 5169(7) | 2474(4) | 3246(7) | 33(2) |
| N2 | 2590(4) | 3483(2) | 4891(3) | 34.8(11) |
| N1 | 5940(4) | 3480(2) | 3519(4) | 36.9(12) |

| Atom | *x* | *y* | *z* | U(eq) |
|---|---|---|---|---|
| C41 | 2589(7) | 2041(4) | 3873(7) | 39(2) |
| C42 | 2441(8) | 2260(4) | 4736(8) | 36(2) |
| C43 | 4040(7) | 2055(4) | 5590(7) | 40(2) |
| C44 | 5093(8) | 2235(4) | 5897(8) | 39(2) |
| C45 | 5968(8) | 2065(4) | 4657(7) | 42(2) |
| C46 | 6118(8) | 2267(4) | 3734(8) | 38(2) |
| C39 | 4507(7) | 2032(4) | 2942(7) | 38(2) |
| C40A | 3277(16) | 2081(9) | 3207(16) | 48(2) |
| C35 | 2188(17) | 2800(9) | 2864(10) | 35(3) |
| C36 | 2685(5) | 3241(3) | 2444(5) | 45.0(16) |
| C37 | 6357(18) | 2810(6) | 5524(16) | 35(3) |
| C38 | 5868(5) | 3269(3) | 6021(5) | 41.4(15) |
| C18 | 2489(5) | 3282(2) | 5677(4) | 37.5(14) |
| C19 | 1789(5) | 3443(3) | 6204(4) | 37.8(14) |
| C20 | 1081(5) | 3807(2) | 5896(4) | 37.6(14) |
| C21 | 1127(5) | 4010(2) | 5043(4) | 35.6(13) |
| C26 | 1914(5) | 3862(2) | 4582(4) | 33.7(13) |
| C25 | 2016(5) | 4105(2) | 3773(4) | 34.2(13) |
| C24 | 1362(5) | 4470(2) | 3428(4) | 38.1(14) |
| C23 | 553(5) | 4611(3) | 3863(5) | 44.8(16) |
| C22 | 440(5) | 4387(3) | 4641(4) | 42.0(15) |
| C28 | 332(5) | 3978(2) | 6471(4) | 35.0(14) |
| C29 | 654(5) | 4185(3) | 7305(4) | 41.7(15) |
| C30 | -28(5) | 4357(3) | 7852(5) | 46.2(16) |
| C31 | -1035(5) | 4308(3) | 7564(5) | 45.9(16) |
| C32 | -1380(5) | 4096(3) | 6741(4) | 40.9(15) |
| C33 | -687(5) | 3933(3) | 6201(5) | 43.1(15) |
| C34 | -2690(6) | 4487(4) | 7855(6) | 64(2) |
| C10 | 5376(6) | 2802(3) | 2441(5) | 50.3(18) |
| C1 | 6007(5) | 3269(2) | 2733(5) | 37.9(14) |
| C2 | 6646(5) | 3444(3) | 2142(5) | 43.3(15) |
| C3 | 7268(5) | 3860(3) | 2357(5) | 45.2(16) |
| C4 | 7209(5) | 4108(3) | 3188(5) | 45.5(16) |
| C9 | 6544(5) | 3908(2) | 3757(5) | 38.6(14) |
| C8 | 6509(5) | 4143(2) | 4573(5) | 41.9(15) |
| C7 | 7069(5) | 4567(3) | 4830(5) | 49.7(17) |
| C6 | 7687(6) | 4784(3) | 4252(6) | 60(2) |
| C5 | 7769(6) | 4564(3) | 3451(6) | 62(2) |
| C11 | 7999(6) | 4019(3) | 1766(5) | 48.4(17) |
| C16 | 7707(6) | 4136(3) | 888(5) | 50.4(17) |
| C15 | 8392(6) | 4261(3) | 321(5) | 53.3(18) |
| C14 | 9413(6) | 4252(3) | 649(5) | 48.9(17) |
| C13 | 9721(6) | 4133(3) | 1523(5) | 48.6(17) |
| C12 | 9007(5) | 4022(3) | 2079(5) | 47.2(17) |
| C17 | 11075(6) | 4403(3) | 359(6) | 58(2) |
| Eu2 | 7210(3) | 3159.2(14) | 7406(4) | 40.0(11) |
| C27 | 3166(6) | 2835(3) | 6024(5) | 49.8(18) |
| O7 | 4262(3) | 4087(2) | 4224(4) | 59.4(15) |

| Atom | *x* | *y* | *z* | U(eq) |
|---|---|---|---|---|
| C42A | 2940(20) | 2194(10) | 5098(16) | 48(2) |
| C46A | 5584(19) | 2157(10) | 3421(15) | 48(2) |
| C44A | 5337(17) | 2142(10) | 5389(16) | 48(2) |
| C41A | 2278(18) | 2361(9) | 4280(17) | 48(2) |
| C45A | 6299(18) | 2341(9) | 4193(17) | 48(2) |
| C43A | 4612(19) | 2307(9) | 5982(16) | 48(2) |
| C40 | 3477(9) | 2195(4) | 2623(9) | 43(3) |
| N5A | 3708(17) | 2592(10) | 5448(18) | 50(4) |
| N6A | 2839(19) | 2552(10) | 3622(18) | 50(4) |
| N3A | 4835(18) | 2544(11) | 3037(19) | 50(4) |
| N4A | 5750(20) | 2566(11) | 4916(19) | 50(4) |
| C39A | 3990(20) | 2263(10) | 2551(16) | 48(2) |
| C1B | -876(10) | 2757(6) | 3859(9) | 108(5) |
| F3 | -1454(11) | 3142(5) | 4092(11) | 152(5) |
| F1 | -1380(18) | 2444(9) | 3266(12) | 221(8) |
| F2 | -106(13) | 2953(9) | 3526(14) | 214(8) |
| S1 | -553(5) | 2339(2) | 4831(4) | 108.5(16) |
| O8 | 115(14) | 2688(7) | 5366(12) | 180(8) |
| O9 | -1472(11) | 2183(9) | 5057(14) | 192(8) |
| O10 | -38(17) | 1928(7) | 4376(16) | 213(9) |
| C1A | 9006(16) | 2341(9) | 3583(17) | 129(8) |
| F3A | 9856(16) | 2297(11) | 3250(15) | 140(7) |
| F2A | 8450(20) | 2747(12) | 3270(20) | 167(10) |
| F1A | 8540(20) | 1913(11) | 3750(30) | 209(11) |
| S1A | 9493(16) | 2621(9) | 4628(17) | 274(10) |
| O8A | 10230(20) | 2203(15) | 5000(20) | 182(12) |
| O10A | 9940(20) | 3099(10) | 4300(20) | 138(9) |
| O9A | 8662(19) | 2676(13) | 5166(17) | 137(9) |
| C37A | 6370(40) | 2904(12) | 5660(30) | 35(3) |
| C35A | 2230(30) | 2830(20) | 3070(20) | 35(3) |

**Table S13.** Atomic Occupancy [**Eu.4PhOMe**]$^{+}$.

| Atom | *Occupancy* | Atom | *Occupancy* | Atom | *Occupancy* |
|---|---|---|---|---|---|
| Eu1 | 0.93 | N6 | 0.65 | N5 | 0.65 |
| N4 | 0.65 | N3 | 0.65 | C41 | 0.65 |
| H41A | 0.65 | H41B | 0.65 | C42 | 0.65 |
| H42A | 0.65 | H42B | 0.65 | C43 | 0.65 |
| H43A | 0.65 | H43B | 0.65 | C44 | 0.65 |
| H44A | 0.65 | H44B | 0.65 | C45 | 0.65 |
| H45A | 0.65 | H45B | 0.65 | C46 | 0.65 |
| H46A | 0.65 | H46B | 0.65 | C39 | 0.65 |
| H39A | 0.65 | H39B | 0.65 | C40A | 0.35 |
| H40A | 0.35 | H40B | 0.35 | C35 | 0.65 |
| H35A | 0.65 | H35B | 0.65 | C37 | 0.65 |
| H37A | 0.65 | H37B | 0.65 | H10A | 0.65 |
| H10B | 0.65 | H10C | 0.35 | H10D | 0.35 |
| Eu2 | 0.07 | H27C | 0.35 | H27D | 0.35 |

| Atom | *Occupancy* | Atom | *Occupancy* | Atom | *Occupancy* |
|---|---|---|---|---|---|
| H27A | 0.65 | H27B | 0.65 | C42A | 0.35 |
| H42C | 0.35 | H42D | 0.35 | C46A | 0.35 |
| H46C | 0.35 | H46D | 0.35 | C44A | 0.35 |
| H44C | 0.35 | H44D | 0.35 | C41A | 0.35 |
| H41C | 0.35 | H41D | 0.35 | C45A | 0.35 |
| H45C | 0.35 | H45D | 0.35 | C43A | 0.35 |
| H43C | 0.35 | H43D | 0.35 | C40 | 0.65 |
| H40C | 0.65 | H40D | 0.65 | N5A | 0.35 |
| N6A | 0.35 | N3A | 0.35 | N4A | 0.35 |
| C39A | 0.35 | H39C | 0.35 | H39D | 0.35 |
| C1B | 0.65 | F3 | 0.65 | F1 | 0.65 |
| F2 | 0.65 | S1 | 0.65 | O8 | 0.65 |
| O9 | 0.65 | O10 | 0.65 | C1A | 0.35 |
| F3A | 0.35 | F2A | 0.35 | F1A | 0.35 |
| S1A | 0.35 | O8A | 0.35 | O10A | 0.35 |
| O9A | 0.35 | C37A | 0.35 | H37C | 0.35 |
| H37D | 0.35 | C35A | 0.35 | H35C | 0.35 |
| H35D | 0.35 | | | | |

**Table S14.** Torsion angles determining the square antiprismatic geometry of [**Eu.4PhOMe**]$^{+}$.

| **Complex 1** | **torsion angle / °** | **Complex 1** | **torsion angle / °** |
|---|---|---|---|
| O6-Eu1-N1-O1 | 43.7(2) | O6-Eu1-N1-N5 | 40.8(2) |
| O6-Eu1-N1-O3 | 43.8(2) | O6-Eu1-N3-N6 | 37.8(5) |
| | | O6-Eu1-N3-N6A | 41.6(7) |

**Compound 3c**

**Table S15.** Bond lengths for compound **3c**.

| Atom | Atom | Length/Å | Atom | Atom | Length/Å |
|---|---|---|---|---|---|
| O2 | C14 | 1.3697(11) | C11 | C12 | 1.3986(11) |
| O2 | C17 | 1.4266(11) | C11 | C16 | 1.4063(12) |
| O1 | C10 | 1.4194(12) | C7 | C6 | 1.4258(12) |
| N1 | C9 | 1.3725(11) | C13 | C12 | 1.3901(13) |
| N1 | C1 | 1.3267(10) | C13 | C14 | 1.3942(13) |
| C9 | C8 | 1.4142(11) | C14 | C15 | 1.3995(12) |
| C9 | C4 | 1.4213(11) | C15 | C16 | 1.3822(12) |
| C8 | C7 | 1.3817(11) | C2 | C3 | 1.3623(15) |
| C1 | C2 | 1.4208(12) | C4 | C5 | 1.4144(14) |
| C1 | C10 | 1.5096(13) | C4 | C3 | 1.4154(13) |
| C11 | C7 | 1.4830(12) | C6 | C5 | 1.3677(14) |

**Table S16.** Bond angles for compound **3c**.

| Atom | Atom | Atom | Angle/˚ | Atom | Atom | Atom | Angle/˚ |
|---|---|---|---|---|---|---|---|
| C14 | O2 | C17 | 117.02(7) | C12 | C13 | C14 | 119.53(8) |
| C1 | N1 | C9 | 118.57(7) | C13 | C12 | C11 | 121.81(8) |
| N1 | C9 | C8 | 118.53(7) | O2 | C14 | C13 | 124.46(7) |
| N1 | C9 | C4 | 122.01(7) | O2 | C14 | C15 | 115.81(8) |
| C8 | C9 | C4 | 119.46(8) | C13 | C14 | C15 | 119.72(8) |
| C7 | C8 | C9 | 121.36(7) | C16 | C15 | C14 | 120.00(8) |
| N1 | C1 | C2 | 122.75(8) | C3 | C2 | C1 | 119.27(8) |
| N1 | C1 | C10 | 116.81(7) | C15 | C16 | C11 | 121.39(7) |
| C2 | C1 | C10 | 120.40(8) | C5 | C4 | C9 | 118.55(8) |
| C12 | C11 | C7 | 121.49(7) | C5 | C4 | C3 | 123.87(8) |
| C12 | C11 | C16 | 117.51(8) | C3 | C4 | C9 | 117.58(8) |
| C16 | C11 | C7 | 120.95(7) | C5 | C6 | C7 | 121.15(8) |
| C8 | C7 | C11 | 120.58(7) | C6 | C5 | C4 | 120.96(8) |
| C8 | C7 | C6 | 118.50(8) | O1 | C10 | C1 | 110.51(7) |
| C6 | C7 | C11 | 120.91(7) | C2 | C3 | C4 | 119.82(8) |

**Table S17.** Hydrogen bonds for compound **3c**.

| D | H | A | d(D-H)/Å | d(H-A)/Å | d(D-A)/Å | D-H-A/° |
|---|---|---|---|---|---|---|
| O1 | H1 | N11 | 0.84 | 1.99 | 2.8041(9) | 163.2 |

**Table S18.** Torsion angles for compound **3c**.

| A | B | C | D | Angle/˚ | A | B | C | D | Angle/˚ |
|---|---|---|---|---|---|---|---|---|---|
| O2 | C14 | C15 | C16 | 179.98(7) | C7 | C11 | C16 | C15 | -175.52(7) |
| N1 | C9 | C8 | C7 | 179.46(7) | C7 | C6 | C5 | C4 | 0.02(13) |
| N1 | C9 | C4 | C5 | -179.88(7) | C13 | C14 | C15 | C16 | -1.20(13) |

| A | B | C | D | Angle/˚ | A | B | C | D | Angle/˚ |
|---|---|---|---|---|---|---|---|---|---|
| N1 | C9 | C4 | C3 | -0.14(12) | C12 | C11 | C7 | C8 | -147.19(8) |
| N1 | C1 | C2 | C3 | 0.03(13) | C12 | C11 | C7 | C6 | 31.88(12) |
| N1 | C1 | C10 | O1 | 86.28(9) | C12 | C11 | C16 | C15 | 1.83(12) |
| C9 | N1 | C1 | C2 | 0.42(12) | C12 | C13 | C14 | O2 | -179.77(8) |
| C9 | N1 | C1 | C10 | -177.37(7) | C12 | C13 | C14 | C15 | 1.51(13) |
| C9 | C8 | C7 | C11 | 179.71(7) | C14 | C13 | C12 | C11 | -0.13(13) |
| C9 | C8 | C7 | C6 | 0.61(12) | C14 | C15 | C16 | C11 | -0.51(13) |
| C9 | C4 | C5 | C6 | 0.21(13) | C2 | C1 | C10 | O1 | -91.56(9) |
| C9 | C4 | C3 | C2 | 0.59(12) | C16 | C11 | C7 | C8 | 30.05(11) |
| C8 | C9 | C4 | C5 | -0.03(12) | C16 | C11 | C7 | C6 | -150.87(8) |
| C8 | C9 | C4 | C3 | 179.71(7) | C16 | C11 | C12 | C13 | -1.51(12) |
| C8 | C7 | C6 | C5 | -0.43(12) | C4 | C9 | C8 | C7 | -0.39(12) |
| C1 | N1 | C9 | C8 | 179.80(7) | C5 | C4 | C3 | C2 | -179.69(8) |
| C1 | N1 | C9 | C4 | -0.36(11) | C10 | C1 | C2 | C3 | 177.74(8) |
| C1 | C2 | C3 | C4 | -0.54(13) | C3 | C4 | C5 | C6 | -179.51(8) |
| C11 | C7 | C6 | C5 | -179.53(8) | C17 | O2 | C14 | C13 | -0.34(12) |
| C7 | C11 | C12 | C13 | 175.83(8) | C17 | O2 | C14 | C15 | 178.42(8) |

**Table S19.** Fractional Atomic Coordinates and Equivalent Isotropic Displacement Parameters (Å$^2$) for compound **3c**.

| Atom | *x* | *y* | *z* | U(eq) |
|---|---|---|---|---|
| O2 | 5261.5(6) | 5088.6(9) | 8897.4(4) | 31.66(15) |
| O1 | 10135.8(6) | -468.0(9) | 3702.1(4) | 31.97(16) |
| N1 | 9403.8(6) | 2594.8(9) | 4809.2(4) | 24.34(15) |
| C9 | 8550.0(7) | 3840.4(10) | 4870.0(5) | 23.82(16) |
| C8 | 8055.8(7) | 3938.5(10) | 5649.5(5) | 23.77(16) |
| C1 | 9865.2(8) | 2498.3(11) | 4077.2(5) | 26.95(17) |
| C11 | 6688.5(7) | 5248.8(10) | 6575.2(6) | 25.25(16) |
| C7 | 7200.6(7) | 5171.5(10) | 5754.6(5) | 25.19(16) |
| C13 | 4977.7(8) | 5747.7(11) | 7326.9(6) | 29.70(18) |
| C12 | 5488.7(7) | 5776.4(11) | 6570.3(6) | 28.83(18) |
| C14 | 5672.4(8) | 5176.2(11) | 8115.1(6) | 27.00(17) |
| C15 | 6883.5(8) | 4677.0(11) | 8140.8(6) | 28.25(18) |
| C2 | 9518.2(9) | 3637.2(13) | 3356.4(5) | 31.53(19) |
| C16 | 7379.4(7) | 4721.5(11) | 7383.4(6) | 27.00(17) |
| C4 | 8155.2(8) | 5026.2(11) | 4177.1(5) | 28.16(18) |
| C6 | 6810.6(8) | 6347.6(11) | 5052.3(6) | 30.83(19) |
| C5 | 7273.5(9) | 6274.2(12) | 4291.6(6) | 32.6(2) |
| C10 | 10759.4(8) | 1060.7(13) | 4022.3(5) | 31.07(19) |
| C3 | 8677.9(9) | 4886.9(12) | 3410.6(6) | 32.6(2) |
| C17 | 4039.7(9) | 5632.4(14) | 8895.5(7) | 38.9(2) |

**Complex** [**Eu.7PhOMe**]$^{+}$

**Table S20.** Bond lengths for [**Eu.7PhOMe**]$^{+}$.

| Atom | Atom | Length/Å | Atom | Atom | Length/Å |
|---|---|---|---|---|---|
| Eu2 | O5 | 2.367(3) | F3 | C93 | 1.372(10) |
| Eu2 | O7 | 2.327(3) | C48 | C47 | 1.505(8) |
| Eu2 | N11 | 2.597(4) | C83 | C82 | 1.409(11) |
| Eu2 | N8 | 2.596(4) | C83 | C78 | 1.404(11) |
| Eu2 | N12 | 2.565(15) | C29 | C28 | 1.807(18) |
| Eu2 | N7 | 2.609(4) | C29 | N6A | 1.351(14) |
| Eu2 | N9 | 2.568(5) | C29 | C30A | 1.396(11) |
| Eu2 | N10 | 2.624(4) | C50 | C49 | 1.499(10) |
| Eu2 | C92 | 3.182(5) | C13 | C12 | 1.398(8) |
| Eu2 | C73 | 3.242(4) | C82 | C81 | 1.397(13) |
| Eu2 | N12A | 2.76(2) | F1 | C93 | 1.322(9) |
| Eu1 | O6 | 2.388(3) | C58 | C57 | 1.348(8) |
| Eu1 | O3 | 2.356(3) | C35A | C38A | 1.440(16) |
| Eu1 | O1 | 2.329(4) | C35A | C34A | 1.388(10) |
| Eu1 | N5 | 2.706(4) | C91 | C92 | 1.518(9) |
| Eu1 | N6 | 2.732(17) | C75 | C76 | 1.392(11) |
| Eu1 | N4 | 2.693(4) | C75 | C74 | 1.262(17) |
| Eu1 | N1 | 2.676(5) | C54 | C53 | 1.499(9) |
| Eu1 | N3 | 2.666(5) | C20 | C98 | 1.382(9) |
| Eu1 | N2 | 2.700(5) | C6 | C5 | 1.539(10) |
| Eu1 | N6A | 2.82(2) | C10 | C9 | 1.472(9) |
| S1 | O15 | 1.428(6) | C10 | C11 | 1.425(9) |
| S1 | O14 | 1.440(6) | O8A | C92 | 1.243(8) |
| S1 | O13 | 1.430(5) | C84B | C85B | 1.37(2) |
| S1 | C93 | 1.749(10) | C84B | C89B | 1.38(2) |
| O5 | C73 | 1.277(5) | C84B | C81A | 1.452(15) |
| O7 | C92 | 1.290(7) | C81 | C80 | 1.406(11) |
| O6 | C73 | 1.247(5) | C81 | C84A | 1.47(2) |
| O3 | C46 | 1.285(7) | C12 | C11 | 1.335(9) |
| O1 | C27A | 1.399(14) | C8 | C7 | 1.480(10) |
| O1 | C27B | 1.221(18) | C45 | C46 | 1.476(9) |
| N11 | C64 | 1.370(6) | C38B | C39B | 1.30(2) |
| N11 | C56 | 1.319(6) | C38B | C43B | 1.51(2) |
| O9 | C22 | 1.368(6) | C38B | C35B | 1.463(19) |
| O9 | C25 | 1.417(7) | C28 | C29A | 1.297(15) |
| N8 | C72 | 1.463(6) | C31A | C30 | 1.378(10) |
| N8 | C48 | 1.483(8) | C31A | C32A | 1.410(11) |
| N8 | C49 | 1.489(7) | C30 | C29A | 1.389(10) |
| O11 | C68 | 1.381(7) | C41A | O10A | 1.241(17) |
| O11 | C71 | 1.409(8) | C41A | C42A | 1.40(2) |
| N12 | C83 | 1.343(15) | C41A | C40A | 1.50(2) |
| N12 | C75 | 1.317(15) | C52 | C51 | 1.511(9) |
| N5 | C18 | 1.409(7) | C76 | C77 | 1.387(11) |
| N5 | C10 | 1.333(7) | C78 | C79 | 1.403(11) |
| N6 | C37 | 1.367(12) | C78 | C77 | 1.382(13) |
| N6 | C29A | 1.347(12) | C79 | C80 | 1.379(12) |
| C63 | C64 | 1.397(7) | C92 | O8B | 1.333(18) |
| C63 | C62 | 1.392(6) | C43A | C42A | 1.395(19) |
| N4 | C6 | 1.486(8) | C43A | C38A | 1.445(19) |
| N4 | C45 | 1.473(7) | C3 | C4 | 1.455(12) |
| N4 | C7 | 1.489(7) | C74 | C75A | 1.739(16) |

| Atom | Atom | Length/Å | Atom | Atom | Length/Å |
|---|---|---|---|---|---|
| C64 | C59 | 1.410(7) | C1 | C2 | 1.501(12) |
| O4 | C46 | 1.232(7) | C26 | C27A | 1.320(16) |
| C23 | C24 | 1.388(7) | C26 | C27B | 1.81(2) |
| C23 | C22 | 1.408(7) | O10A | C21 | 1.62(2) |
| N7 | C55 | 1.483(7) | C39A | C40A | 1.35(2) |
| N7 | C47 | 1.480(7) | C39A | C38A | 1.436(18) |
| N7 | C54 | 1.482(7) | C40B | C41B | 1.45(2) |
| C24 | C19 | 1.376(7) | C40B | C39B | 1.33(3) |
| C15 | C16 | 1.413(7) | C87B | C88B | 1.34(2) |
| C15 | C14 | 1.345(8) | C87B | O12B | 1.420(19) |
| C65 | C66 | 1.392(7) | C87B | C86B | 1.38(2) |
| C65 | C62 | 1.471(7) | C85B | C86B | 1.365(19) |
| C65 | C70 | 1.417(7) | C88B | C89B | 1.40(2) |
| C56 | C55 | 1.507(7) | O12B | C90B | 1.381(19) |
| C56 | C57 | 1.408(7) | C86A | C85A | 1.38(2) |
| N1 | C8 | 1.504(8) | C86A | C87A | 1.39(2) |
| N1 | C9 | 1.506(10) | C85A | C84A | 1.42(2) |
| N1 | C1 | 1.483(9) | C87A | C88A | 1.45(3) |
| C22 | C98 | 1.384(8) | C87A | O12A | 1.33(2) |
| N9 | C50 | 1.471(7) | C88A | C89A | 1.38(3) |
| N9 | C74 | 1.514(10) | C89A | C84A | 1.31(2) |
| N9 | C51 | 1.474(8) | O12A | C90A | 1.54(3) |
| C68 | C69 | 1.379(8) | C41B | O10B | 1.30(2) |
| C68 | C67 | 1.407(7) | C41B | C42B | 1.39(3) |
| C36 | C37 | 1.402(10) | O10B | C27 | 1.414(17) |
| C36 | C35A | 1.377(11) | C42B | C43B | 1.35(3) |
| C16 | C17 | 1.382(7) | O2A | C27A | 1.229(14) |
| C16 | C19 | 1.483(7) | C34A | C33A | 1.387(11) |
| C59 | C58 | 1.407(8) | C33A | C32A | 1.395(10) |
| C59 | C60 | 1.426(8) | C34B | C33B | 1.349(12) |
| N10 | C91 | 1.495(7) | C34B | C35B | 1.379(11) |
| N10 | C53 | 1.463(8) | C33B | C32B | 1.395(11) |
| N10 | C52 | 1.477(7) | C35B | C36A | 1.374(13) |
| C37 | C32A | 1.393(10) | C31B | C32B | 1.381(13) |
| C14 | C13 | 1.408(8) | C31B | C30A | 1.375(11) |
| N3 | C28 | 1.454(9) | C32B | C37A | 1.391(11) |
| N3 | C5 | 1.487(8) | O2B | C27B | 1.21(2) |
| N3 | C4 | 1.523(9) | C36A | C37A | 1.426(14) |
| C66 | C67 | 1.373(7) | C37A | N6A | 1.371(14) |
| C69 | C70 | 1.363(8) | C76A | C77A | 1.369(10) |
| F2 | C93 | 1.333(8) | C76A | C75A | 1.393(11) |
| C62 | C61 | 1.417(7) | C77A | C78A | 1.395(11) |
| N2 | C3 | 1.458(10) | C78A | C79A | 1.399(10) |
| N2 | C2 | 1.447(11) | C78A | C83A | 1.410(10) |
| N2 | C26 | 1.479(9) | C79A | C80A | 1.397(10) |
| C17 | C18 | 1.401(7) | C80A | C81A | 1.409(10) |
| C61 | C60 | 1.344(8) | C81A | C82A | 1.391(12) |
| C72 | C73 | 1.519(6) | C82A | C83A | 1.412(10) |
| C18 | C13 | 1.417(7) | C83A | N12A | 1.345(14) |
| C19 | C20 | 1.408(7) | N12A | C75A | 1.325(17) |

**Table S21.** Bond angles for [**Eu.7PhOMe**]$^+$.

| Atom | Atom | Atom | Angle/° | Atom | Atom | Atom | Angle/° |
|---|---|---|---|---|---|---|---|
| O5 | Eu2 | N11 | 75.43(10) | C3 | N2 | Eu1 | 108.3(4) |
| O5 | Eu2 | N8 | 65.31(10) | C3 | N2 | C26 | 108.6(6) |
| O5 | Eu2 | N12 | 87.1(3) | C2 | N2 | Eu1 | 114.3(5) |
| O5 | Eu2 | N7 | 88.19(11) | C2 | N2 | C3 | 106.9(6) |
| O5 | Eu2 | N9 | 123.17(12) | C2 | N2 | C26 | 110.2(6) |
| O5 | Eu2 | N10 | 154.39(13) | C26 | N2 | Eu1 | 108.4(4) |
| O5 | Eu2 | C92 | 139.84(14) | C16 | C17 | C18 | 121.5(4) |
| O5 | Eu2 | C73 | 19.34(10) | C60 | C61 | C62 | 121.5(5) |
| O5 | Eu2 | N12A | 82.6(3) | N8 | C72 | C73 | 111.4(4) |
| O7 | Eu2 | O5 | 132.81(11) | N5 | C18 | C13 | 121.3(4) |
| O7 | Eu2 | N11 | 83.20(11) | C17 | C18 | N5 | 120.5(4) |
| O7 | Eu2 | N8 | 155.04(13) | C17 | C18 | C13 | 118.2(4) |
| O7 | Eu2 | N12 | 74.1(4) | C24 | C19 | C16 | 123.2(4) |
| O7 | Eu2 | N7 | 121.25(12) | C24 | C19 | C20 | 117.4(5) |
| O7 | Eu2 | N9 | 86.03(14) | C20 | C19 | C16 | 119.4(4) |
| O7 | Eu2 | N10 | 65.10(13) | N8 | C48 | C47 | 112.3(4) |
| O7 | Eu2 | C92 | 20.46(15) | N12 | C83 | C82 | 119.8(14) |
| O7 | Eu2 | C73 | 143.74(11) | N12 | C83 | C78 | 125.6(15) |
| O7 | Eu2 | N12A | 72.6(4) | C78 | C83 | C82 | 114.7(15) |
| N11 | Eu2 | N7 | 67.05(12) | N6A | C29 | C28 | 113.9(14) |
| N11 | Eu2 | N10 | 91.46(13) | N6A | C29 | C30A | 126.7(18) |
| N11 | Eu2 | C92 | 72.53(15) | C30A | C29 | C28 | 119.4(14) |
| N11 | Eu2 | C73 | 93.79(11) | N9 | C50 | C49 | 112.1(5) |
| N11 | Eu2 | N12A | 120.1(3) | C69 | C70 | C65 | 121.3(5) |
| N8 | Eu2 | N11 | 121.07(13) | C14 | C13 | C18 | 119.3(5) |
| N8 | Eu2 | N7 | 69.48(14) | C12 | C13 | C14 | 122.5(5) |
| N8 | Eu2 | N10 | 105.45(13) | C12 | C13 | C18 | 118.2(5) |
| N8 | Eu2 | C92 | 154.25(15) | C81 | C82 | C83 | 123.0(16) |
| N8 | Eu2 | C73 | 48.40(11) | C57 | C58 | C59 | 120.6(5) |
| N8 | Eu2 | N12A | 97.0(4) | C66 | C67 | C68 | 118.4(5) |
| N12 | Eu2 | N11 | 128.4(3) | N7 | C55 | C56 | 111.0(4) |
| N12 | Eu2 | N8 | 92.4(4) | C36 | C35A | C38A | 124.0(11) |
| N12 | Eu2 | N7 | 161.6(4) | C36 | C35A | C34A | 121.1(10) |
| N12 | Eu2 | N9 | 62.7(3) | C34A | C35A | C38A | 114.8(10) |
| N12 | Eu2 | N10 | 117.9(3) | C61 | C60 | C59 | 121.4(5) |
| N12 | Eu2 | C92 | 94.2(4) | N10 | C91 | C92 | 112.0(5) |
| N12 | Eu2 | C73 | 80.0(4) | N12 | C75 | C76 | 128.1(14) |
| N7 | Eu2 | N10 | 66.32(13) | C74 | C75 | N12 | 122.4(12) |
| N7 | Eu2 | C92 | 100.80(15) | C74 | C75 | C76 | 109.5(11) |
| N7 | Eu2 | C73 | 89.77(11) | N7 | C47 | C48 | 113.0(4) |
| N7 | Eu2 | N12A | 166.0(4) | N7 | C54 | C53 | 111.3(5) |
| N9 | Eu2 | N11 | 160.74(13) | C98 | C20 | C19 | 120.6(5) |
| N9 | Eu2 | N8 | 69.05(14) | N4 | C6 | C5 | 111.2(5) |
| N9 | Eu2 | N7 | 105.83(16) | C22 | C98 | C20 | 121.3(5) |
| N9 | Eu2 | N10 | 69.49(15) | N8 | C49 | C50 | 112.1(5) |
| N9 | Eu2 | C92 | 92.14(16) | N5 | C10 | C9 | 120.2(5) |
| N9 | Eu2 | C73 | 104.28(12) | N5 | C10 | C11 | 121.7(5) |

| Atom | Atom | Atom | Angle/° | Atom | Atom | Atom | Angle/° |
|---|---|---|---|---|---|---|---|
| N9 | Eu2 | N12A | 71.1(3) | C11 | C10 | C9 | 118.0(5) |
| N10 | Eu2 | C92 | 49.85(15) | C85B | C84B | C89B | 119.0(14) |
| N10 | Eu2 | C73 | 151.12(12) | C85B | C84B | C81A | 120.4(15) |
| N10 | Eu2 | N12A | 122.9(3) | C89B | C84B | C81A | 120.7(14) |
| C92 | Eu2 | C73 | 157.35(14) | C82 | C81 | C80 | 119.5(14) |
| N12A | Eu2 | C92 | 93.1(4) | C82 | C81 | C84A | 120.7(12) |
| N12A | Eu2 | C73 | 78.0(4) | C80 | C81 | C84A | 119.8(13) |
| O6 | Eu1 | N5 | 73.96(11) | C11 | C12 | C13 | 120.1(5) |
| O6 | Eu1 | N6 | 74.7(4) | N10 | C53 | C54 | 111.8(4) |
| O6 | Eu1 | N4 | 129.10(12) | C7 | C8 | N1 | 110.2(5) |
| O6 | Eu1 | N1 | 128.98(15) | C58 | C57 | C56 | 118.7(5) |
| O6 | Eu1 | N3 | 128.24(14) | N4 | C45 | C46 | 112.2(5) |
| O6 | Eu1 | N2 | 129.98(12) | O3 | C46 | C45 | 115.3(5) |
| O6 | Eu1 | N6A | 71.5(6) | O4 | C46 | O3 | 123.3(6) |
| O3 | Eu1 | O6 | 76.51(10) | O4 | C46 | C45 | 121.4(5) |
| O3 | Eu1 | N5 | 85.56(13) | C39B | C38B | C43B | 114.5(18) |
| O3 | Eu1 | N6 | 91.6(3) | C39B | C38B | C35B | 125.3(19) |
| O3 | Eu1 | N4 | 64.47(13) | C35B | C38B | C43B | 120.2(15) |
| O3 | Eu1 | N1 | 70.77(16) | N3 | C28 | C29 | 117.2(7) |
| O3 | Eu1 | N3 | 129.29(14) | C29A | C28 | N3 | 117.8(6) |
| O3 | Eu1 | N2 | 134.96(17) | C30 | C31A | C32A | 120.5(10) |
| O3 | Eu1 | N6A | 85.0(4) | C31A | C30 | C29A | 120.7(10) |
| O1 | Eu1 | O6 | 76.56(11) | O10A | C41A | C42A | 137.4(18) |
| O1 | Eu1 | O3 | 153.03(11) | O10A | C41A | C40A | 109.6(16) |
| O1 | Eu1 | N5 | 85.66(14) | C42A | C41A | C40A | 112.6(12) |
| O1 | Eu1 | N6 | 82.7(4) | N10 | C52 | C51 | 111.1(5) |
| O1 | Eu1 | N4 | 137.50(13) | C77 | C76 | C75 | 115.2(14) |
| O1 | Eu1 | N1 | 127.36(16) | C8 | C7 | N4 | 113.9(5) |
| O1 | Eu1 | N3 | 70.75(13) | C83 | C78 | C79 | 123.8(14) |
| O1 | Eu1 | N2 | 65.15(16) | C77 | C78 | C83 | 116.8(14) |
| O1 | Eu1 | N6A | 87.9(5) | C77 | C78 | C79 | 119.4(14) |
| N5 | Eu1 | N6 | 148.3(4) | C80 | C79 | C78 | 119.2(13) |
| N5 | Eu1 | N6A | 145.4(6) | O7 | C92 | Eu2 | 39.1(2) |
| N4 | Eu1 | N5 | 130.22(14) | O7 | C92 | C91 | 116.2(5) |
| N4 | Eu1 | N6 | 75.1(5) | O7 | C92 | O8B | 114.6(12) |
| N4 | Eu1 | N2 | 100.90(14) | C91 | C92 | Eu2 | 81.1(3) |
| N4 | Eu1 | N6A | 73.7(7) | O8A | C92 | Eu2 | 147.3(5) |
| N1 | Eu1 | N5 | 65.60(15) | O8A | C92 | O7 | 124.7(6) |
| N1 | Eu1 | N6 | 142.3(5) | O8A | C92 | C91 | 118.2(6) |
| N1 | Eu1 | N4 | 67.27(15) | O8B | C92 | Eu2 | 150.5(12) |
| N1 | Eu1 | N2 | 64.44(19) | O8B | C92 | C91 | 112.7(10) |
| N1 | Eu1 | N6A | 140.0(7) | C79 | C80 | C81 | 119.6(13) |
| N3 | Eu1 | N5 | 139.26(16) | C76 | C77 | C78 | 120.4(14) |
| N3 | Eu1 | N6 | 62.4(3) | N3 | C5 | C6 | 114.3(5) |
| N3 | Eu1 | N4 | 66.89(14) | C42A | C43A | C38A | 126.5(14) |
| N3 | Eu1 | N1 | 102.78(18) | C10 | C9 | N1 | 114.1(6) |
| N3 | Eu1 | N2 | 67.77(19) | C4 | C3 | N2 | 112.4(6) |
| N3 | Eu1 | N6A | 68.4(4) | C3 | C4 | N3 | 113.0(6) |

| Atom | Atom | Atom | Angle/˚ | Atom | Atom | Atom | Angle/˚ |
|---|---|---|---|---|---|---|---|
| N2 | Eu1 | N5 | 72.30(16) | N9 | C74 | C75A | 117.4(6) |
| N2 | Eu1 | N6 | 127.2(3) | C75 | C74 | N9 | 115.0(8) |
| N2 | Eu1 | N6A | 134.0(4) | N1 | C1 | C2 | 113.0(5) |
| O15 | S1 | O14 | 112.1(4) | N9 | C51 | C52 | 112.1(5) |
| O15 | S1 | C93 | 105.4(4) | N2 | C2 | C1 | 112.1(6) |
| O14 | S1 | C93 | 102.4(4) | N2 | C26 | C27B | 111.1(8) |
| O13 | S1 | O15 | 114.7(4) | C27A | C26 | N2 | 116.3(8) |
| O13 | S1 | O14 | 116.3(4) | C12 | C11 | C10 | 120.8(5) |
| O13 | S1 | C93 | 104.2(3) | F2 | C93 | S1 | 114.8(6) |
| C73 | O5 | Eu2 | 122.8(3) | F2 | C93 | F3 | 103.5(7) |
| C92 | O7 | Eu2 | 120.5(3) | F3 | C93 | S1 | 110.9(6) |
| C73 | O6 | Eu1 | 150.0(3) | F1 | C93 | S1 | 114.9(7) |
| C46 | O3 | Eu1 | 128.3(3) | F1 | C93 | F2 | 106.9(7) |
| C27A | O1 | Eu1 | 121.7(6) | F1 | C93 | F3 | 104.8(7) |
| C27B | O1 | Eu1 | 140.5(9) | C41A | O10A | C21 | 103.3(16) |
| C64 | N11 | Eu2 | 124.9(3) | C40A | C39A | C38A | 123.3(15) |
| C56 | N11 | Eu2 | 114.8(3) | C39B | C40B | C41B | 121.0(18) |
| C56 | N11 | C64 | 119.3(4) | C43A | C42A | C41A | 121.5(14) |
| C22 | O9 | C25 | 118.9(4) | O5 | C73 | Eu2 | 37.84(19) |
| C72 | N8 | Eu2 | 105.2(3) | O5 | C73 | C72 | 115.9(4) |
| C72 | N8 | C48 | 107.7(4) | O6 | C73 | Eu2 | 160.3(3) |
| C72 | N8 | C49 | 110.1(4) | O6 | C73 | O5 | 124.3(4) |
| C48 | N8 | Eu2 | 109.9(3) | O6 | C73 | C72 | 119.8(4) |
| C48 | N8 | C49 | 112.1(4) | C72 | C73 | Eu2 | 78.6(2) |
| C49 | N8 | Eu2 | 111.5(3) | C88B | C87B | O12B | 116.2(14) |
| C68 | O11 | C71 | 117.6(5) | C88B | C87B | C86B | 118.4(14) |
| C83 | N12 | Eu2 | 127.6(10) | C86B | C87B | O12B | 125.4(14) |
| C75 | N12 | Eu2 | 115.8(10) | C86B | C85B | C84B | 119.1(14) |
| C75 | N12 | C83 | 113.3(14) | C87B | C88B | C89B | 120.3(14) |
| C18 | N5 | Eu1 | 129.0(3) | C84B | C89B | C88B | 120.4(14) |
| C10 | N5 | Eu1 | 113.0(3) | C90B | O12B | C87B | 117.9(12) |
| C10 | N5 | C18 | 117.7(4) | C87A | C86A | C85A | 123.6(14) |
| C37 | N6 | Eu1 | 131.2(10) | C86A | C85A | C84A | 122.1(15) |
| C29A | N6 | Eu1 | 111.2(9) | C86A | C87A | C88A | 112.3(16) |
| C29A | N6 | C37 | 117.4(14) | O12A | C87A | C86A | 119.6(18) |
| C62 | C63 | C64 | 122.5(4) | O12A | C87A | C88A | 128(2) |
| C6 | N4 | Eu1 | 111.5(3) | C89A | C88A | C87A | 121.7(18) |
| C45 | N4 | Eu1 | 107.8(3) | C84A | C89A | C88A | 125(2) |
| C45 | N4 | C6 | 110.6(5) | C87A | O12A | C90A | 114.6(18) |
| C45 | N4 | C7 | 107.1(4) | C85B | C86B | C87B | 122.7(16) |
| C7 | N4 | Eu1 | 109.0(3) | C81 | C84A | C85A | 119.4(15) |
| C7 | N4 | C6 | 110.6(4) | C89A | C84A | C81 | 125.7(17) |
| N11 | C64 | C63 | 120.3(4) | C89A | C84A | C85A | 115(2) |
| N11 | C64 | C59 | 120.8(4) | C39A | C40A | C41A | 124.6(14) |
| C63 | C64 | C59 | 118.9(4) | O10B | C41B | C40B | 104.6(17) |
| C24 | C23 | C22 | 118.9(5) | O10B | C41B | C42B | 137(2) |
| C55 | N7 | Eu2 | 105.3(3) | C42B | C41B | C40B | 118.3(17) |
| C47 | N7 | Eu2 | 110.8(3) | C41B | O10B | C27 | 103.0(17) |

| Atom | Atom | Atom | Angle/˚ | Atom | Atom | Atom | Angle/˚ |
|---|---|---|---|---|---|---|---|
| C47 | N7 | C55 | 109.7(4) | C40B | C39B | C38B | 124.9(19) |
| C47 | N7 | C54 | 108.9(4) | C43B | C42B | C41B | 118(2) |
| C54 | N7 | Eu2 | 114.1(3) | C43A | C38A | C35A | 123.9(12) |
| C54 | N7 | C55 | 108.0(4) | C39A | C38A | C35A | 124.5(13) |
| C19 | C24 | C23 | 123.0(5) | C39A | C38A | C43A | 111.4(14) |
| C14 | C15 | C16 | 120.6(5) | C42B | C43B | C38B | 124(2) |
| C66 | C65 | C62 | 122.6(4) | C35A | C34A | C33A | 117.6(10) |
| C66 | C65 | C70 | 116.7(5) | C32A | C33A | C34A | 120.2(10) |
| C70 | C65 | C62 | 120.7(4) | C33B | C34B | C35B | 118.1(16) |
| N11 | C56 | C55 | 118.0(4) | C34B | C33B | C32B | 120.4(15) |
| N11 | C56 | C57 | 122.6(5) | C34B | C35B | C38B | 115.1(14) |
| C57 | C56 | C55 | 119.4(5) | C36A | C35B | C38B | 121.4(13) |
| C8 | N1 | Eu1 | 111.9(3) | C36A | C35B | C34B | 123.4(15) |
| C8 | N1 | C9 | 107.8(6) | C37 | C32A | C31A | 114.5(10) |
| C9 | N1 | Eu1 | 103.6(3) | C37 | C32A | C33A | 123.4(10) |
| C1 | N1 | Eu1 | 116.5(5) | C33A | C32A | C31A | 122.1(9) |
| C1 | N1 | C8 | 106.4(5) | C30A | C31B | C32B | 123.8(17) |
| C1 | N1 | C9 | 110.4(6) | C31B | C32B | C33B | 121.4(14) |
| O9 | C22 | C23 | 124.9(5) | C31B | C32B | C37A | 115.7(15) |
| O9 | C22 | C98 | 116.4(5) | C37A | C32B | C33B | 122.7(14) |
| C98 | C22 | C23 | 118.8(5) | C26 | C27A | O1 | 122.1(9) |
| C50 | N9 | Eu2 | 110.9(3) | O2A | C27A | O1 | 120.5(12) |
| C50 | N9 | C74 | 107.1(5) | O2A | C27A | C26 | 116.9(11) |
| C50 | N9 | C51 | 109.8(5) | O1 | C27B | C26 | 101.8(12) |
| C74 | N9 | Eu2 | 107.8(4) | O2B | C27B | O1 | 132.5(17) |
| C51 | N9 | Eu2 | 110.9(4) | O2B | C27B | C26 | 124.6(15) |
| C51 | N9 | C74 | 110.2(5) | C35B | C36A | C37A | 119.0(15) |
| O11 | C68 | C69 | 116.5(5) | C32B | C37A | C36A | 115.9(15) |
| O11 | C68 | C67 | 123.4(5) | N6A | C37A | C32B | 125.0(17) |
| C69 | C68 | C67 | 120.1(5) | N6A | C37A | C36A | 119.1(14) |
| C35A | C36 | C37 | 123.2(11) | C29 | N6A | Eu1 | 113.9(13) |
| C15 | C16 | C19 | 119.4(4) | C29 | N6A | C37A | 114(2) |
| C17 | C16 | C15 | 119.0(5) | C37A | N6A | Eu1 | 131.2(14) |
| C17 | C16 | C19 | 121.6(4) | N6 | C29A | C30 | 120.9(12) |
| C64 | C59 | C60 | 118.3(5) | C28 | C29A | N6 | 122.8(10) |
| C58 | C59 | C64 | 117.8(5) | C28 | C29A | C30 | 116.1(9) |
| C58 | C59 | C60 | 123.8(5) | C31B | C30A | C29 | 114.5(16) |
| C91 | N10 | Eu2 | 103.3(3) | C77A | C76A | C75A | 116.4(11) |
| C53 | N10 | Eu2 | 114.7(3) | C76A | C77A | C78A | 118.6(11) |
| C53 | N10 | C91 | 110.2(5) | C77A | C78A | C79A | 121.8(10) |
| C53 | N10 | C52 | 110.1(5) | C77A | C78A | C83A | 120.8(11) |
| C52 | N10 | Eu2 | 108.6(3) | C83A | C78A | C79A | 117.3(10) |
| C52 | N10 | C91 | 109.6(4) | C80A | C79A | C78A | 122.3(10) |
| N6 | C37 | C36 | 119.5(11) | C79A | C80A | C81A | 120.9(10) |
| N6 | C37 | C32A | 125.9(12) | C80A | C81A | C84B | 120.9(11) |
| C32A | C37 | C36 | 114.3(11) | C82A | C81A | C84B | 122.2(11) |
| C15 | C14 | C13 | 121.3(5) | C82A | C81A | C80A | 116.9(12) |
| C28 | N3 | Eu1 | 106.0(3) | C81A | C82A | C83A | 122.7(13) |

| Atom | Atom | Atom | Angle/˚ | Atom | Atom | Atom | Angle/˚ |
|---|---|---|---|---|---|---|---|
| C28 | N3 | C5 | 110.5(6) | C78A | C83A | C82A | 119.9(12) |
| C28 | N3 | C4 | 110.0(6) | N12A | C83A | C78A | 120.2(13) |
| C5 | N3 | Eu1 | 114.5(4) | N12A | C83A | C82A | 119.9(12) |
| C5 | N3 | C4 | 106.6(5) | C83A | N12A | Eu2 | 129.7(11) |
| C4 | N3 | Eu1 | 109.3(4) | C75A | N12A | Eu2 | 112.0(10) |
| C67 | C66 | C65 | 123.0(4) | C75A | N12A | C83A | 117.1(16) |
| C70 | C69 | C68 | 120.6(5) | C76A | C75A | C74 | 114.8(10) |
| C63 | C62 | C65 | 120.2(4) | N12A | C75A | C74 | 118.2(11) |
| C63 | C62 | C61 | 117.5(5) | N12A | C75A | C76A | 126.8(13) |
| C61 | C62 | C65 | 122.3(4) | | | | |

**Table S22.** Torsion angles for [**Eu.7PhOMe**]$^+$.

| A | B | C | D | Angle/˚ | A | B | C | D | Angle/˚ |
|---|---|---|---|---|---|---|---|---|---|
| Eu2 | O5 | C73 | O6 | -168.2(3) | C60 | C59 | C58 | C57 | 175.6(5) |
| Eu2 | O5 | C73 | C72 | 10.6(5) | C91 | N10 | C53 | C54 | 77.3(6) |
| Eu2 | O7 | C92 | C91 | 28.4(7) | C91 | N10 | C52 | C51 | -158.1(6) |
| Eu2 | O7 | C92 | O8A | -140.4(6) | C75 | N12 | C83 | C82 | -178.5(19) |
| Eu2 | O7 | C92 | O8B | 162.6(11) | C75 | N12 | C83 | C78 | 1(3) |
| Eu2 | N11 | C64 | C63 | 20.7(5) | C75 | C76 | C77 | C78 | -7(3) |
| Eu2 | N11 | C64 | C59 | -161.7(3) | C47 | N7 | C55 | C56 | -171.8(4) |
| Eu2 | N11 | C56 | C55 | -16.8(5) | C47 | N7 | C54 | C53 | 82.3(5) |
| Eu2 | N11 | C56 | C57 | 163.2(4) | C54 | N7 | C55 | C56 | 69.7(5) |
| Eu2 | N8 | C72 | C73 | -44.5(5) | C54 | N7 | C47 | C48 | -165.1(5) |
| Eu2 | N8 | C48 | C47 | -45.1(5) | C6 | N4 | C45 | C46 | 158.7(4) |
| Eu2 | N8 | C49 | C50 | -37.2(5) | C6 | N4 | C7 | C8 | -170.3(5) |
| Eu2 | N12 | C83 | C82 | -20(3) | C49 | N8 | C72 | C73 | -164.8(4) |
| Eu2 | N12 | C83 | C78 | 159.8(16) | C49 | N8 | C48 | C47 | 79.5(5) |
| Eu2 | N12 | C75 | C76 | -169.3(16) | C10 | N5 | C18 | C17 | -179.2(5) |
| Eu2 | N12 | C75 | C74 | 9(2) | C10 | N5 | C18 | C13 | -0.4(7) |
| Eu2 | N7 | C55 | C56 | -52.6(4) | C84B | C85B | C86B | C87B | 1(2) |
| Eu2 | N7 | C47 | C48 | -38.8(6) | C84B | C81A | C82A | C83A | -178.5(16) |
| Eu2 | N7 | C54 | C53 | -42.0(5) | O15 | S1 | C93 | F2 | -56.5(7) |
| Eu2 | N9 | C50 | C49 | -46.3(6) | O15 | S1 | C93 | F3 | 60.2(6) |
| Eu2 | N9 | C74 | C75 | -43.4(10) | O15 | S1 | C93 | F1 | 178.9(6) |
| Eu2 | N9 | C74 | C75A | -39.2(9) | O14 | S1 | C93 | F2 | 60.9(7) |
| Eu2 | N9 | C51 | C52 | -42.8(7) | O14 | S1 | C93 | F3 | 177.6(5) |
| Eu2 | N10 | C91 | C92 | -40.0(5) | O14 | S1 | C93 | F1 | -63.7(6) |
| Eu2 | N10 | C53 | C54 | -38.9(6) | C53 | N10 | C91 | C92 | -163.0(5) |
| Eu2 | N10 | C52 | C51 | -45.9(7) | C53 | N10 | C52 | C51 | 80.5(7) |
| Eu2 | N12A | C75A | C74 | 8.3(19) | C8 | N1 | C9 | C10 | 172.5(5) |
| Eu2 | N12A | C75A | C76A | -166.4(15) | C8 | N1 | C1 | C2 | -156.3(7) |
| Eu1 | O6 | C73 | Eu2 | -163.9(5) | C57 | C56 | C55 | N7 | -130.1(5) |
| Eu1 | O6 | C73 | O5 | -142.1(4) | C45 | N4 | C6 | C5 | -166.9(5) |
| Eu1 | O6 | C73 | C72 | 39.2(8) | C45 | N4 | C7 | C8 | 69.1(6) |
| Eu1 | O3 | C46 | O4 | -165.4(4) | C38B | C35B | C36A | C37A | -172.1(19) |
| Eu1 | O3 | C46 | C45 | 16.5(7) | O13 | S1 | C93 | F2 | -177.6(6) |
| Eu1 | O1 | C27A | C26 | -17.5(14) | O13 | S1 | C93 | F3 | -60.8(6) |

| A | B | C | D | Angle/° | A | B | C | D | Angle/° |
|---|---|---|---|---|---|---|---|---|---|
| Eu1 | O1 | C27A | O2A | 170.9(9) | O13 | S1 | C93 | F1 | 57.8(6) |
| Eu1 | O1 | C27B | C26 | 23.5(18) | C71 | O11 | C68 | C69 | -168.6(5) |
| Eu1 | O1 | C27B | O2B | -168.7(15) | C71 | O11 | C68 | C67 | 10.6(7) |
| Eu1 | N5 | C18 | C17 | -5.3(7) | C28 | N3 | C5 | C6 | 89.0(7) |
| Eu1 | N5 | C18 | C13 | 173.5(4) | C28 | N3 | C4 | C3 | -157.1(5) |
| Eu1 | N5 | C10 | C9 | 4.8(8) | C28 | C29 | N6A | Eu1 | -9(3) |
| Eu1 | N5 | C10 | C11 | -178.1(5) | C28 | C29 | N6A | C37A | 179(2) |
| Eu1 | N6 | C37 | C36 | 16(3) | C28 | C29 | C30A | C31B | 176.9(16) |
| Eu1 | N6 | C37 | C32A | -170.4(13) | C31A | C30 | C29A | N6 | 0(2) |
| Eu1 | N6 | C29A | C28 | -3.5(19) | C31A | C30 | C29A | C28 | 175.7(10) |
| Eu1 | N6 | C29A | C30 | 171.8(9) | C30 | C31A | C32A | C37 | -2.6(18) |
| Eu1 | N4 | C6 | C5 | -46.8(5) | C30 | C31A | C32A | C33A | 176.3(11) |
| Eu1 | N4 | C45 | C46 | 36.4(5) | C52 | N10 | C91 | C92 | 75.6(6) |
| Eu1 | N4 | C7 | C8 | -47.3(6) | C52 | N10 | C53 | C54 | -161.7(5) |
| Eu1 | N1 | C8 | C7 | -42.2(6) | C76 | C75 | C74 | N9 | -157.1(12) |
| Eu1 | N1 | C9 | C10 | 53.8(6) | C7 | N4 | C6 | C5 | 74.6(6) |
| Eu1 | N1 | C1 | C2 | -30.8(9) | C7 | N4 | C45 | C46 | -80.7(5) |
| Eu1 | N3 | C28 | C29 | 49.2(9) | C78 | C83 | C82 | C81 | -4(3) |
| Eu1 | N3 | C28 | C29A | 50.5(8) | C78 | C79 | C80 | C81 | -4(2) |
| Eu1 | N3 | C5 | C6 | -30.5(8) | C79 | C78 | C77 | C76 | -179.3(17) |
| Eu1 | N3 | C4 | C3 | -41.1(6) | C80 | C81 | C84A | C85A | -44(2) |
| Eu1 | N2 | C3 | C4 | -49.7(7) | C80 | C81 | C84A | C89A | 137.8(18) |
| Eu1 | N2 | C2 | C1 | -45.0(7) | C77 | C78 | C79 | C80 | -177.5(16) |
| Eu1 | N2 | C26 | C27A | 21.0(10) | C5 | N3 | C28 | C29 | -75.3(9) |
| Eu1 | N2 | C26 | C27B | 36.0(8) | C5 | N3 | C28 | C29A | -74.1(8) |
| N11 | C64 | C59 | C58 | -2.7(7) | C5 | N3 | C4 | C3 | 83.1(7) |
| N11 | C64 | C59 | C60 | -179.9(4) | C9 | N1 | C8 | C7 | -155.5(5) |
| N11 | C56 | C55 | N7 | 49.9(6) | C9 | N1 | C1 | C2 | 86.9(8) |
| N11 | C56 | C57 | C58 | 2.0(8) | C9 | C10 | C11 | C12 | -178.3(7) |
| O9 | C22 | C98 | C20 | -178.8(6) | C3 | N2 | C2 | C1 | 74.8(8) |
| N8 | C72 | C73 | Eu2 | 33.5(4) | C3 | N2 | C26 | C27A | -96.5(9) |
| N8 | C72 | C73 | O5 | 26.9(6) | C3 | N2 | C26 | C27B | -81.5(8) |
| N8 | C72 | C73 | O6 | -154.2(4) | C4 | N3 | C28 | C29 | 167.3(9) |
| N8 | C48 | C47 | N7 | 58.6(6) | C4 | N3 | C28 | C29A | 168.5(7) |
| O11 | C68 | C69 | C70 | 179.7(4) | C4 | N3 | C5 | C6 | -151.5(7) |
| O11 | C68 | C67 | C66 | -179.4(4) | C74 | N9 | C50 | C49 | -163.7(5) |
| N12 | C83 | C82 | C81 | 175(2) | C74 | N9 | C51 | C52 | 76.6(7) |
| N12 | C83 | C78 | C79 | -178(2) | C74 | C75 | C76 | C77 | -167.4(15) |
| N12 | C83 | C78 | C77 | 2(3) | C1 | N1 | C8 | C7 | 86.1(7) |
| N12 | C75 | C76 | C77 | 11(3) | C1 | N1 | C9 | C10 | -71.6(6) |
| N12 | C75 | C74 | N9 | 25(2) | C51 | N9 | C50 | C49 | 76.6(6) |
| N5 | C18 | C13 | C14 | -175.7(5) | C51 | N9 | C74 | C75 | -164.6(10) |
| N5 | C18 | C13 | C12 | 2.9(7) | C51 | N9 | C74 | C75A | -160.4(8) |
| N5 | C10 | C9 | N1 | -42.7(8) | C2 | N2 | C3 | C4 | -173.3(6) |
| N5 | C10 | C11 | C12 | 4.5(10) | C2 | N2 | C26 | C27A | 146.7(9) |
| N6 | C37 | C32A | C31A | 0(3) | C2 | N2 | C26 | C27B | 161.7(8) |
| N6 | C37 | C32A | C33A | -178.9(18) | C26 | N2 | C3 | C4 | 67.8(8) |
| C63 | C64 | C59 | C58 | 175.0(5) | C26 | N2 | C2 | C1 | -167.3(6) |

| A | B | C | D | Angle/˚ | A | B | C | D | Angle/˚ |
|---|---|---|---|---|---|---|---|---|---|
| C63 | C64 | C59 | C60 | -2.2(7) | C11 | C10 | C9 | N1 | 140.0(6) |
| C63 | C62 | C61 | C60 | -1.6(7) | O10A | C41A | C42A | C43A | -174.6(17) |
| N4 | C6 | C5 | N3 | 52.9(8) | O10A | C41A | C40A | C39A | 175.6(12) |
| N4 | C45 | C46 | O3 | -36.9(6) | C40B | C41B | O10B | C27 | 170.1(14) |
| N4 | C45 | C46 | O4 | 144.9(6) | C40B | C41B | C42B | C43B | 0(2) |
| C64 | N11 | C56 | C55 | 173.9(4) | C42A | C41A | O10A | C21 | -16(2) |
| C64 | N11 | C56 | C57 | -6.1(7) | C42A | C41A | C40A | C39A | 1.6(19) |
| C64 | C63 | C62 | C65 | -177.5(4) | C42A | C43A | C38A | C35A | 178.2(12) |
| C64 | C63 | C62 | C61 | 0.3(6) | C42A | C43A | C38A | C39A | 2.8(17) |
| C64 | C59 | C58 | C57 | -1.4(8) | C87B | C88B | C89B | C84B | 2(2) |
| C64 | C59 | C60 | C61 | 0.9(8) | C85B | C84B | C89B | C88B | -3(2) |
| C23 | C24 | C19 | C16 | -179.2(4) | C85B | C84B | C81A | C80A | -43(2) |
| C23 | C24 | C19 | C20 | -0.7(7) | C85B | C84B | C81A | C82A | 136.6(17) |
| C23 | C22 | C98 | C20 | 2.0(9) | C88B | C87B | O12B | C90B | -171.1(12) |
| N7 | C54 | C53 | N10 | 53.8(6) | C88B | C87B | C86B | C85B | -3(2) |
| C24 | C23 | C22 | O9 | 179.7(5) | C89B | C84B | C85B | C86B | 2(2) |
| C24 | C23 | C22 | C98 | -1.2(7) | C89B | C84B | C81A | C80A | 136.7(15) |
| C24 | C19 | C20 | C98 | 1.5(8) | C89B | C84B | C81A | C82A | -44(2) |
| C15 | C16 | C17 | C18 | 2.4(7) | O12B | C87B | C88B | C89B | -177.9(12) |
| C15 | C16 | C19 | C24 | 141.8(5) | O12B | C87B | C86B | C85B | 176.4(11) |
| C15 | C16 | C19 | C20 | -36.7(7) | C86A | C85A | C84A | C81 | -175.9(13) |
| C15 | C14 | C13 | C18 | -0.2(7) | C86A | C85A | C84A | C89A | 3(2) |
| C15 | C14 | C13 | C12 | -178.7(5) | C86A | C87A | C88A | C89A | 1(2) |
| C65 | C66 | C67 | C68 | 0.8(7) | C86A | C87A | O12A | C90A | -157.1(19) |
| C65 | C62 | C61 | C60 | 176.1(5) | C85A | C86A | C87A | C88A | 3(2) |
| C56 | N11 | C64 | C63 | -171.2(4) | C85A | C86A | C87A | O12A | -179.8(12) |
| C56 | N11 | C64 | C59 | 6.4(6) | C87A | C86A | C85A | C84A | -5(2) |
| N1 | C8 | C7 | N4 | 62.4(7) | C87A | C88A | C89A | C84A | -4(3) |
| N1 | C1 | C2 | N2 | 50.5(10) | C88A | C87A | O12A | C90A | 20(2) |
| C22 | C23 | C24 | C19 | 0.6(7) | C88A | C89A | C84A | C81 | -179.6(16) |
| N9 | C50 | C49 | N8 | 57.5(6) | C88A | C89A | C84A | C85A | 2(3) |
| N9 | C74 | C75A | C76A | -163.2(11) | O12A | C87A | C88A | C89A | -175.5(16) |
| N9 | C74 | C75A | N12A | 21(2) | C86B | C87B | C88B | C89B | 1.4(19) |
| C68 | C69 | C70 | C65 | -1.1(7) | C86B | C87B | O12B | C90B | 9.6(19) |
| C36 | C37 | C32A | C31A | 173.7(14) | C84A | C81 | C80 | C79 | 179.9(15) |
| C36 | C37 | C32A | C33A | -5(2) | C40A | C41A | O10A | C21 | 172.0(12) |
| C36 | C35A | C38A | C43A | 23(2) | C40A | C41A | C42A | C43A | -3.0(17) |
| C36 | C35A | C38A | C39A | -162.2(13) | C40A | C39A | C38A | C35A | -179.5(12) |
| C36 | C35A | C34A | C33A | -1(2) | C40A | C39A | C38A | C43A | -4.2(17) |
| C16 | C15 | C14 | C13 | -1.7(7) | C41B | C40B | C39B | C38B | 0(3) |
| C16 | C17 | C18 | N5 | 174.6(4) | C41B | C42B | C43B | C38B | 1(3) |
| C16 | C17 | C18 | C13 | -4.2(7) | O10B | C41B | C42B | C43B | 177.4(18) |
| C16 | C19 | C20 | C98 | -179.9(5) | C39B | C38B | C43B | C42B | -2(3) |
| C59 | C58 | C57 | C56 | 1.8(8) | C39B | C38B | C35B | C34B | 20(2) |
| N10 | C91 | C92 | Eu2 | 31.5(4) | C39B | C38B | C35B | C36A | -160.8(18) |
| N10 | C91 | C92 | O7 | 13.8(7) | C39B | C40B | C41B | O10B | -178.7(14) |
| N10 | C91 | C92 | O8A | -176.6(6) | C39B | C40B | C41B | C42B | -1(2) |
| N10 | C91 | C92 | O8B | -121.2(13) | C42B | C41B | O10B | C27 | -7(3) |

| A | B | C | D | Angle/˚ | A | B | C | D | Angle/˚ |
|---|---|---|---|---|---|---|---|---|---|
| N10 | C52 | C51 | N9 | 62.2(8) | C38A | C35A | C34A | C33A | 177.4(12) |
| C37 | N6 | C29A | C28 | -178.0(15) | C38A | C43A | C42A | C41A | 0.8(19) |
| C37 | N6 | C29A | C30 | -3(3) | C38A | C39A | C40A | C41A | 2.2(19) |
| C37 | C36 | C35A | C38A | -178.8(15) | C43B | C38B | C39B | C40B | 1(2) |
| C37 | C36 | C35A | C34A | -1(2) | C43B | C38B | C35B | C34B | -160.3(16) |
| C14 | C15 | C16 | C17 | 0.7(7) | C43B | C38B | C35B | C36A | 19(2) |
| C14 | C15 | C16 | C19 | -178.8(4) | C34A | C35A | C38A | C43A | -155.4(12) |
| C14 | C13 | C12 | C11 | 176.8(6) | C34A | C35A | C38A | C39A | 19.4(19) |
| N3 | C28 | C29A | N6 | -33.6(17) | C34A | C33A | C32A | C37 | 3.9(19) |
| N3 | C28 | C29A | C30 | 150.9(8) | C34A | C33A | C32A | C31A | -174.9(12) |
| C66 | C65 | C62 | C63 | -25.9(6) | C34B | C33B | C32B | C31B | -176(2) |
| C66 | C65 | C62 | C61 | 156.4(4) | C34B | C33B | C32B | C37A | 0(3) |
| C66 | C65 | C70 | C69 | 1.5(6) | C34B | C35B | C36A | C37A | 7(3) |
| C69 | C68 | C67 | C66 | -0.4(7) | C33B | C34B | C35B | C38B | 176.8(17) |
| C62 | C63 | C64 | N11 | 179.3(4) | C33B | C34B | C35B | C36A | -3(3) |
| C62 | C63 | C64 | C59 | 1.6(6) | C33B | C32B | C37A | C36A | 5(3) |
| C62 | C65 | C66 | C67 | 176.9(4) | C33B | C32B | C37A | N6A | -178(3) |
| C62 | C65 | C70 | C69 | -176.8(4) | C35B | C38B | C39B | C40B | -178.7(16) |
| C62 | C61 | C60 | C59 | 1.0(8) | C35B | C38B | C43B | C42B | 178.3(16) |
| N2 | C3 | C4 | N3 | 64.5(8) | C35B | C34B | C33B | C32B | -1(3) |
| N2 | C26 | C27A | O1 | -5.8(15) | C35B | C36A | C37A | C32B | -8(3) |
| N2 | C26 | C27A | O2A | 166.1(10) | C35B | C36A | C37A | N6A | 175(2) |
| N2 | C26 | C27B | O1 | -37.6(13) | C32A | C31A | C30 | C29A | 2.7(17) |
| N2 | C26 | C27B | O2B | 153.2(16) | C31B | C32B | C37A | C36A | -179(2) |
| C17 | C16 | C19 | C24 | -37.7(7) | C31B | C32B | C37A | N6A | -2(4) |
| C17 | C16 | C19 | C20 | 143.8(5) | C32B | C31B | C30A | C29 | 3(3) |
| C17 | C18 | C13 | C14 | 3.1(7) | C32B | C37A | N6A | Eu1 | -165.3(19) |
| C17 | C18 | C13 | C12 | -178.3(5) | C32B | C37A | N6A | C29 | 4(4) |
| C72 | N8 | C48 | C47 | -159.2(4) | C36A | C37A | N6A | Eu1 | 12(4) |
| C72 | N8 | C49 | C50 | 79.2(5) | C36A | C37A | N6A | C29 | -179(2) |
| C18 | N5 | C10 | C9 | 179.6(6) | N6A | C29 | C28 | N3 | -29(2) |
| C18 | N5 | C10 | C11 | -3.2(9) | N6A | C29 | C30A | C31B | -1(4) |
| C18 | C13 | C12 | C11 | -1.7(9) | C29A | N6 | C37 | C36 | -170.7(17) |
| C19 | C16 | C17 | C18 | -178.1(4) | C29A | N6 | C37 | C32A | 3(3) |
| C19 | C20 | C98 | C22 | -2.2(10) | C30A | C29 | C28 | N3 | 153.2(15) |
| C48 | N8 | C72 | C73 | 72.7(5) | C30A | C29 | N6A | Eu1 | 168.9(18) |
| C48 | N8 | C49 | C50 | -161.0(5) | C30A | C29 | N6A | C37A | -2(4) |
| C83 | N12 | C75 | C76 | -8(3) | C30A | C31B | C32B | C33B | 174.3(19) |
| C83 | N12 | C75 | C74 | 170.0(18) | C30A | C31B | C32B | C37A | -2(3) |
| C83 | C82 | C81 | C80 | 3(3) | C76A | C77A | C78A | C79A | 179.3(13) |
| C83 | C82 | C81 | C84A | -175.6(19) | C76A | C77A | C78A | C83A | 1(2) |
| C83 | C78 | C79 | C80 | 2(3) | C77A | C76A | C75A | C74 | -173.9(12) |
| C83 | C78 | C77 | C76 | 1(3) | C77A | C76A | C75A | N12A | 1(3) |
| C50 | N9 | C74 | C75 | 76.0(11) | C77A | C78A | C79A | C80A | -178.4(13) |
| C50 | N9 | C74 | C75A | 80.2(9) | C77A | C78A | C83A | C82A | 179.3(16) |
| C50 | N9 | C51 | C52 | -165.7(6) | C77A | C78A | C83A | N12A | 2(2) |
| C70 | C65 | C66 | C67 | -1.4(6) | C78A | C79A | C80A | C81A | -1(2) |
| C70 | C65 | C62 | C63 | 152.3(4) | C78A | C83A | N12A | Eu2 | 162.5(12) |

| A | B | C | D | Angle/˚ | A | B | C | D | Angle/˚ |
|---|---|---|---|---|---|---|---|---|---|
| C70 | C65 | C62 | C61 | -25.4(6) | C78A | C83A | N12A | C75A | -3(3) |
| C13 | C12 | C11 | C10 | -1.9(10) | C79A | C78A | C83A | C82A | 1(2) |
| C82 | C83 | C78 | C79 | 2(3) | C79A | C78A | C83A | N12A | -176.3(15) |
| C82 | C83 | C78 | C77 | -178.4(19) | C79A | C80A | C81A | C84B | 179.4(13) |
| C82 | C81 | C80 | C79 | 2(2) | C79A | C80A | C81A | C82A | 0(2) |
| C82 | C81 | C84A | C85A | 134.7(18) | C80A | C81A | C82A | C83A | 1(3) |
| C82 | C81 | C84A | C89A | -44(3) | C81A | C84B | C85B | C86B | -178.9(12) |
| C58 | C59 | C60 | C61 | -176.0(5) | C81A | C84B | C89B | C88B | 177.5(13) |
| C67 | C68 | C69 | C70 | 0.5(7) | C81A | C82A | C83A | C78A | -1(3) |
| C55 | N7 | C47 | C48 | 76.9(6) | C81A | C82A | C83A | N12A | 175.9(18) |
| C55 | N7 | C54 | C53 | -158.6(4) | C82A | C83A | N12A | Eu2 | -15(3) |
| C55 | C56 | C57 | C58 | -178.0(5) | C82A | C83A | N12A | C75A | 179.4(18) |
| C25 | O9 | C22 | C23 | 3.9(8) | C83A | C78A | C79A | C80A | 0(2) |
| C25 | O9 | C22 | C98 | -175.2(5) | C83A | N12A | C75A | C74 | 176.7(15) |
| C35A | C36 | C37 | N6 | 177.7(18) | C83A | N12A | C75A | C76A | 2(3) |
| C35A | C36 | C37 | C32A | 4(2) | C75A | C76A | C77A | C78A | -2(2) |
| C35A | C34A | C33A | C32A | -0.6(18) | | | | | |

**Table S23.** Fractional Atomic Coordinates and Equivalent Isotropic Displacement Parameters (Å$^2$) for [**Eu.7PhOMe**]$^+$.

| Atom | *x* | *y* | *z* | U(eq) |
|---|---|---|---|---|
| Eu2 | 8077.3(2) | 2343.7(2) | 3410.0(2) | 27.77(9) |
| Eu1 | 6833.8(2) | 6124.7(2) | 3926.6(2) | 32.44(9) |
| S1 | 11181.8(10) | 738.9(12) | 4803.4(5) | 77.2(5) |
| O5 | 7313.1(15) | 3447.3(16) | 3556.3(8) | 27.7(6) |
| O7 | 8071.6(17) | 909(2) | 3536.7(10) | 41.9(8) |
| O6 | 7135.2(15) | 4687.0(17) | 3834.3(8) | 29.8(6) |
| O3 | 8052.8(18) | 6131.0(19) | 3987.4(11) | 42.4(8) |
| O1 | 5767.2(19) | 5472(2) | 3807.9(12) | 54.5(10) |
| N11 | 6938.9(18) | 2000(2) | 3016.0(10) | 29.8(8) |
| O9 | 3948(2) | 1780(2) | 3244.7(12) | 57.9(10) |
| N8 | 8620(2) | 3834(2) | 3396.0(13) | 40.6(10) |
| O11 | 5919(2) | -626(2) | 5125.1(11) | 59.2(10) |
| N12 | 8334(6) | 2197(11) | 4136(5) | 24(3) |
| N5 | 7002(2) | 5943(3) | 3164.6(12) | 46.3(10) |
| N6 | 6741(7) | 5389(13) | 4631(6) | 27(3) |
| C63 | 6481(2) | 1053(3) | 3476.3(14) | 32.2(10) |
| N4 | 7394(2) | 7247(2) | 4429.0(13) | 46.3(11) |
| C64 | 6484(2) | 1377(3) | 3103.8(13) | 31.6(10) |
| O4 | 9074(2) | 6657(3) | 4176.7(16) | 78.1(14) |
| C23 | 5051(3) | 2521(3) | 3375.7(14) | 38.3(11) |
| N7 | 8070(2) | 2913(2) | 2707.4(12) | 38.9(9) |
| C24 | 5504(2) | 3128(3) | 3250.5(13) | 34.4(10) |
| C15 | 5955(3) | 4310(3) | 2385.1(14) | 41.7(12) |
| C65 | 6025(2) | 132(3) | 3980.8(15) | 35.6(11) |
| C56 | 6873(3) | 2372(3) | 2678.1(14) | 36.9(11) |
| N1 | 7313(3) | 7494(3) | 3582.2(16) | 64.8(15) |

| Atom | *x* | *y* | *z* | U(eq) |
|---|---|---|---|---|
| C22 | 4435(3) | 2362(3) | 3155.3(15) | 42.5(12) |
| N9 | 9355(2) | 2309(3) | 3647.7(16) | 57.6(13) |
| C68 | 5985(3) | -375(3) | 4749.1(16) | 45.0(13) |
| C36 | 7853(7) | 4734(9) | 4713(4) | 23(3) |
| C16 | 5863(2) | 4232(3) | 2783.2(13) | 35.4(10) |
| C59 | 5993(3) | 1074(3) | 2825.0(16) | 42.7(12) |
| N10 | 8806(2) | 1430(3) | 2949.6(14) | 45.9(10) |
| C37 | 7172(7) | 4840(11) | 4827(4) | 31(3) |
| C14 | 6402(3) | 4877(3) | 2253.1(15) | 45.4(12) |
| N3 | 5919(2) | 6758(3) | 4396.8(17) | 61.5(14) |
| C66 | 6606(2) | 183(3) | 4230.8(15) | 36.5(11) |
| C69 | 5400(3) | -439(3) | 4508.3(17) | 46.1(13) |
| F2 | 11075(2) | 1666(4) | 4186.9(16) | 112.6(17) |
| C62 | 6024(2) | 427(3) | 3580.6(15) | 36.0(11) |
| N2 | 5868(3) | 7081(3) | 3550.1(18) | 71.2(17) |
| C17 | 6232(3) | 4752(3) | 3035.4(14) | 37.4(11) |
| C61 | 5551(2) | 116(3) | 3291.7(17) | 43.4(12) |
| C72 | 8298(2) | 4299(3) | 3703.0(16) | 42.6(12) |
| C18 | 6676(3) | 5374(3) | 2904.5(14) | 40.0(11) |
| C19 | 5377(2) | 3590(3) | 2920.7(14) | 35.9(10) |
| F3 | 11947(3) | 1935(4) | 4560.2(17) | 121.3(18) |
| C48 | 8422(3) | 4254(3) | 3027.1(17) | 48.9(14) |
| C83 | 7876(9) | 2154(12) | 4413(5) | 36(4) |
| C29 | 6315(10) | 5361(13) | 4837(5) | 54(6) |
| C50 | 9598(3) | 3152(4) | 3761(2) | 63.7(18) |
| C70 | 5415(3) | -201(3) | 4133.0(16) | 42.4(12) |
| C13 | 6776(3) | 5422(3) | 2504.8(15) | 44.1(12) |
| C82 | 7194(9) | 2446(15) | 4340(6) | 34(4) |
| F1 | 11942(3) | 836(5) | 4209.1(16) | 131(2) |
| C58 | 5969(3) | 1458(4) | 2460.9(16) | 51.5(14) |
| C67 | 6603(3) | -55(3) | 4609.3(15) | 40.6(11) |
| C55 | 7335(3) | 3113(3) | 2605.1(14) | 40.9(11) |
| C25 | 4035(3) | 1330(4) | 3594.6(17) | 57.5(15) |
| C35A | 8326(6) | 4217(8) | 4903(3) | 33(3) |
| C60 | 5531(3) | 427(3) | 2932.6(17) | 48.5(13) |
| C91 | 8376(3) | 655(3) | 2890.9(18) | 54.2(15) |
| C75 | 8945(8) | 1884(11) | 4240(4) | 35(4) |
| C47 | 8494(3) | 3684(3) | 2686.9(17) | 52.1(14) |
| C54 | 8310(3) | 2297(3) | 2423.8(18) | 53.2(15) |
| C20 | 4760(3) | 3421(3) | 2703.2(17) | 57.1(15) |
| C6 | 6974(3) | 7332(4) | 4773.4(19) | 61.6(17) |
| C98 | 4298(3) | 2827(4) | 2824.9(18) | 61.3(16) |
| C49 | 9385(3) | 3805(4) | 3469(2) | 57.6(16) |
| C10 | 7412(3) | 6527(3) | 3022.2(16) | 55.6(15) |
| O8A | 7713(4) | -288(3) | 3251.3(18) | 64.1(15) |
| C84B | 5740(8) | 2791(11) | 4441(5) | 24(3) |
| C81 | 6676(8) | 2369(10) | 4605(4) | 33(4) |
| O15 | 10780(3) | 1343(4) | 4998.9(19) | 104.8(19) |

| Atom | *x* | *y* | *z* | U(eq) |
|---|---|---|---|---|
| C12 | 7233(3) | 6029(4) | 2375.3(17) | 54.3(14) |
| O14 | 10754(3) | 121(4) | 4605.0(19) | 110(2) |
| C53 | 8931(3) | 1822(4) | 2579.8(19) | 60.3(17) |
| C8 | 7765(4) | 8016(4) | 3852.3(18) | 63.3(17) |
| C57 | 6393(3) | 2105(3) | 2387.3(16) | 49.5(13) |
| C45 | 8101(3) | 6964(3) | 4539.3(18) | 52.2(14) |
| C46 | 8447(3) | 6574(3) | 4215.2(19) | 51.2(14) |
| C38B | 9274(9) | 4243(12) | 4696(5) | 37(4) |
| O13 | 11771(3) | 426(4) | 5022.0(15) | 91.7(16) |
| C71 | 6463(4) | -425(4) | 5392.7(19) | 70.4(18) |
| C28 | 5671(3) | 6052(4) | 4616(2) | 69(2) |
| C31A | 6365(6) | 4588(7) | 5300(3) | 39(3) |
| C30 | 5934(6) | 5165(6) | 5116(3) | 36(3) |
| C41A | 10498(7) | 3968(11) | 4572(5) | 43(3) |
| C52 | 9470(3) | 1211(4) | 3151(2) | 66.0(18) |
| C76 | 9181(9) | 1617(11) | 4603(4) | 63(5) |
| C7 | 7457(4) | 8072(3) | 4232(2) | 61.5(17) |
| C78 | 8009(9) | 1830(14) | 4784(5) | 60(5) |
| C79 | 7509(9) | 1769(9) | 5062(5) | 48(4) |
| C92 | 8055(4) | 373(3) | 3258.6(19) | 59.4(16) |
| C80 | 6838(9) | 2015(9) | 4968(3) | 39(4) |
| C77 | 8676(9) | 1555(10) | 4872(5) | 51(4) |
| C5 | 6199(4) | 7426(4) | 4659(2) | 70(2) |
| C43A | 9401(7) | 4721(10) | 4576(4) | 38(3) |
| C9 | 7763(4) | 7142(4) | 3279.6(19) | 71(2) |
| C3 | 5545(4) | 7618(5) | 3829(3) | 83(2) |
| C4 | 5320(3) | 7150(5) | 4160(3) | 88(3) |
| C74 | 9410(4) | 1764(4) | 4004(2) | 75(2) |
| C1 | 6787(5) | 8072(4) | 3406(2) | 88(3) |
| C51 | 9793(3) | 1968(4) | 3350(2) | 71(2) |
| C2 | 6141(5) | 7629(4) | 3262(3) | 94(3) |
| C26 | 5334(4) | 6520(4) | 3373(3) | 84(2) |
| C11 | 7549(4) | 6556(4) | 2624.3(19) | 66.1(18) |
| C93 | 11537(4) | 1287(6) | 4425(2) | 86(2) |
| O10A | 11110(6) | 3750(8) | 4547(4) | 100(4) |
| C39A | 9471(7) | 3426(7) | 4916(4) | 51(3) |
| C40B | 10382(10) | 3668(12) | 4666(5) | 43(4) |
| C42A | 10087(6) | 4664(8) | 4471(4) | 49(3) |
| C73 | 7522(2) | 4147(3) | 3698.3(12) | 28.8(9) |
| C87B | 4453(7) | 3383(10) | 4178(4) | 42(3) |
| C85B | 5155(8) | 2352(9) | 4519(4) | 34(3) |
| C88B | 5022(7) | 3826(9) | 4109(4) | 34(3) |
| C89B | 5675(8) | 3541(9) | 4244(5) | 22(3) |
| O12B | 3818(6) | 3706(8) | 4019(3) | 44(3) |
| C86A | 4729(11) | 2286(10) | 4486(4) | 49(4) |
| C85A | 5406(9) | 2100(9) | 4595(4) | 44(3) |
| C87A | 4526(9) | 3032(14) | 4307(5) | 47(5) |
| C88A | 5110(11) | 3568(10) | 4230(5) | 51(4) |

| Atom | *x* | *y* | *z* | U(eq) |
|---|---|---|---|---|
| C89A | 5780(11) | 3326(15) | 4322(6) | 42(5) |
| O12A | 3859(5) | 3161(7) | 4213(3) | 74(4) |
| C86B | 4526(8) | 2656(12) | 4391(4) | 34(3) |
| C90B | 3211(7) | 3335(10) | 4128(4) | 75(4) |
| C90A | 3721(19) | 3800(20) | 3887(9) | 134(13) |
| C84A | 5963(11) | 2631(11) | 4503(5) | 33(4) |
| C40A | 10130(8) | 3346(9) | 4812(4) | 60(3) |
| C41B | 10626(8) | 4331(11) | 4420(5) | 56(4) |
| O10B | 11260(7) | 4122(11) | 4356(5) | 73(4) |
| C39B | 9737(9) | 3670(10) | 4784(5) | 51(4) |
| C42B | 10165(9) | 4966(12) | 4307(6) | 65(5) |
| C38A | 9030(9) | 4112(9) | 4795(4) | 39(3) |
| C43B | 9517(10) | 4926(13) | 4435(6) | 56(5) |
| O2A | 4773(4) | 5348(5) | 3432(3) | 65(2) |
| O8B | 8241(12) | -404(11) | 3361(7) | 82(6) |
| C34A | 8152(7) | 3769(7) | 5226(3) | 37(3) |
| C33A | 7489(6) | 3871(7) | 5354(3) | 44(3) |
| C34B | 8465(9) | 3784(11) | 5154(4) | 57(5) |
| C33B | 7835(8) | 3805(10) | 5304(5) | 52(5) |
| C35B | 8568(8) | 4260(10) | 4831(4) | 32(4) |
| C32A | 7024(6) | 4415(6) | 5163(3) | 32(2) |
| C31B | 6674(9) | 4376(11) | 5300(6) | 65(5) |
| C32B | 7310(8) | 4306(11) | 5138(5) | 52(5) |
| O2B | 4647(6) | 5239(9) | 3679(5) | 79(4) |
| C27A | 5293(6) | 5760(8) | 3522(4) | 44(3) |
| C27B | 5183(9) | 5625(11) | 3677(5) | 41(4) |
| C36A | 8055(8) | 4725(13) | 4646(5) | 29(4) |
| C37A | 7402(9) | 4804(14) | 4817(5) | 33(4) |
| N6A | 6909(10) | 5321(19) | 4649(8) | 40(5) |
| C29A | 6129(6) | 5560(8) | 4782(3) | 28(3) |
| C30A | 6160(9) | 4914(12) | 5167(5) | 53(5) |
| C21 | 11400(11) | 4396(14) | 4232(6) | 82(6) |
| C27 | 11439(11) | 4691(13) | 4065(6) | 78(6) |
| C76A | 8916(6) | 1519(8) | 4690(3) | 30(3) |
| C77A | 8390(6) | 1499(8) | 4940(4) | 35(3) |
| C78A | 7747(6) | 1828(9) | 4820(3) | 29(3) |
| C79A | 7184(6) | 1821(7) | 5058(3) | 24(3) |
| C80A | 6533(6) | 2131(8) | 4937(3) | 27(3) |
| C81A | 6418(7) | 2476(8) | 4567(3) | 16(3) |
| C82A | 6979(7) | 2488(13) | 4332(5) | 22(3) |
| C83A | 7636(7) | 2166(9) | 4448(4) | 18(3) |
| N12A | 8146(7) | 2148(12) | 4199(6) | 32(4) |
| C75A | 8753(7) | 1846(12) | 4326(4) | 38(4) |

**Table S24.** Atomic Occupancy [**Eu.7PhOMe**]$^{+}$.

| Atom | *Occupancy* | Atom | *Occupancy* | Atom | *Occupancy* |
|---|---|---|---|---|---|
| N12 | 0.496(12) | N6 | 0.55 | C36 | 0.55 |

| Atom | *Occupancy* | Atom | *Occupancy* | Atom | *Occupancy* |
|---|---|---|---|---|---|
| H36 | 0.55 | C37 | 0.55 | C83 | 0.496(12) |
| C29 | 0.45 | C82 | 0.496(12) | H82 | 0.496(12) |
| C35A | 0.55 | C75 | 0.496(12) | O8A | 0.75 |
| C84B | 0.504(12) | C81 | 0.496(12) | C38B | 0.45 |
| H28C | 0.45 | H28D | 0.45 | H28A | 0.55 |
| H28B | 0.55 | C31A | 0.55 | H31A | 0.55 |
| C30 | 0.55 | H30 | 0.55 | C41A | 0.55 |
| C76 | 0.496(12) | H76 | 0.496(12) | C78 | 0.496(12) |
| C79 | 0.496(12) | H79 | 0.496(12) | C80 | 0.496(12) |
| H80 | 0.496(12) | C77 | 0.496(12) | H77 | 0.496(12) |
| C43A | 0.55 | H43A | 0.55 | H74C | 0.504(12) |
| H74D | 0.504(12) | H74A | 0.496(12) | H74B | 0.496(12) |
| H26A | 0.4(3) | H26B | 0.4(3) | H26C | 0.6(3) |
| H26D | 0.6(3) | O10A | 0.55 | C39A | 0.55 |
| H39A | 0.55 | C40B | 0.45 | H40B | 0.45 |
| C42A | 0.55 | H42A | 0.55 | C87B | 0.504(12) |
| C85B | 0.504(12) | H85B | 0.504(12) | C88B | 0.504(12) |
| H88B | 0.504(12) | C89B | 0.504(12) | H89B | 0.504(12) |
| O12B | 0.504(12) | C86A | 0.496(12) | H86A | 0.496(12) |
| C85A | 0.496(12) | H85A | 0.496(12) | C87A | 0.496(12) |
| C88A | 0.496(12) | H88A | 0.496(12) | C89A | 0.496(12) |
| H89A | 0.496(12) | O12A | 0.496(12) | C86B | 0.504(12) |
| H86B | 0.504(12) | C90B | 0.504(12) | H90A | 0.504(12) |
| H90B | 0.504(12) | H90C | 0.504(12) | C90A | 0.496(12) |
| H90D | 0.496(12) | H90E | 0.496(12) | H90F | 0.496(12) |
| C84A | 0.496(12) | C40A | 0.55 | H40A | 0.55 |
| C41B | 0.45 | O10B | 0.45 | C39B | 0.45 |
| H39B | 0.45 | C42B | 0.45 | H42B | 0.45 |
| C38A | 0.55 | C43B | 0.45 | H43B | 0.45 |
| O2A | 0.59 | O8B | 0.25 | C34A | 0.55 |
| H34A | 0.55 | C33A | 0.55 | H33A | 0.55 |
| C34B | 0.45 | H34B | 0.45 | C33B | 0.45 |
| H33B | 0.45 | C35B | 0.45 | C32A | 0.55 |
| C31B | 0.45 | H31B | 0.45 | C32B | 0.45 |
| O2B | 0.41 | C27A | 0.59 | C27B | 0.41 |
| C36A | 0.45 | H36A | 0.45 | C37A | 0.45 |
| N6A | 0.45 | C29A | 0.55 | C30A | 0.45 |
| H30A | 0.45 | C21 | 0.55 | H21A | 0.55 |
| H21B | 0.55 | H21C | 0.55 | C27 | 0.45 |
| H27A | 0.45 | H27B | 0.45 | H27C | 0.45 |
| C76A | 0.504(12) | H76A | 0.504(12) | C77A | 0.504(12) |
| H77A | 0.504(12) | C78A | 0.504(12) | C79A | 0.504(12) |
| H79A | 0.504(12) | C80A | 0.504(12) | H80A | 0.504(12) |
| C81A | 0.504(12) | C82A | 0.504(12) | H82A | 0.504(12) |
| C83A | 0.504(12) | N12A | 0.504(12) | C75A | 0.504(12) |

**Table S25.** Face-to-face π-π interactions observed in the packing of [**Eu.7PhOMe**]$^{+}$.

| Interaction | centroid-to-centroid / Å | plane-to-plane shift / Å | twist angle / ° |
|---|---|---|---|
| *Intramolecular* | | | |
| phenyl ••• quinoline | 3.754(3) | 1.586(9) | 23.7(7) |
| quinoline$_{major}$ ••• quinoline$_{major}$* | 3.785(8) | 0.21(3) | 48(7) |
| quinoline$_{major}$ ••• quinoline$_{minor}$* | 3.751(9) | 0.32(3) | 146(5) |
| quinoline$_{major}$ ••• quinoline$_{minor}$* | 3.883(10) | 0.75(3) | 135(2) |
| quinoline$_{minor}$ ••• quinoline$_{minor}$* | 3.778(11) | 0.40(3) | 41(5) |
| *Intermolecular* | | | |
| phenyl$_{major}$ ••• phenyl$_{major}$* | 3.824(10) | 1.57(2) | 180(3) |
| phenyl$_{major}$ ••• phenyl$_{minor}$* | 3.758(6) | 1.11(2) | 174(2) |
| phenyl$_{minor}$ ••• phenyl$_{minor}$* | 3.836(13) | 0.97(3) | 180(3) |

*Disordered components (major and minor = part 1 and part 2, respectively) of the aromatic rings in the complex structure.

**Table S26.** Torsion angles determining the square antiprismatic geometry of [**Eu.7PhOMe**]$^{+}$.

| Complex 1 | torsion angle / ° | Complex 1 | torsion angle / ° |
|---|---|---|---|
| O6-Eu1-N1-O1 | 43.7(2) | O6-Eu1-N1-N5 | 40.8(2) |
| O6-Eu1-N1-O3 | 43.8(2) | O6-Eu1-N3-N6 | 37.8(5) |
| | | O6-Eu1-N3-N6A | 41.6(7) |
